# Supplementary material for: Genome-wide rare variant analysis for thousands of phenotypes in over 70,000 exomes from two cohorts
Source: Nat Commun. 2020 Jan 28;11:542. doi: 10.1038/s41467-020-14288-y (PMC6987107; doi:10.1038/s41467-020-14288-y)
Supplement: Supplementary file 1 — Supplementary Information [file 41467_2020_14288_MOESM1_ESM.pdf]

Genome-wide rare variant analysis for thousands of phenotypes in over 70,000 exomes from two cohorts

Cirulli et al.

Supplementary note

Additional analysis methods

Gene-based collapsing analysis utilizing CADD scores

In addition to our LoF and coding models, we also tried two different collapsing models, both of which restricted to rare (MAF<0.1%) variants with a CADD score above the 95% mutation significance cutoff (MSC) for its gene[1–3]. The inclusive CADD model collapsed all such variants that were within 5kb of a gene, and the restrictive CADD model only collapsed coding variants (stop_lost, missense_variant, start_lost, splice_donor_variant, inframe_deletion, frameshift_variant, splice_acceptor_variant, stop_gained, or inframe_insertion) that met these criteria.

In our initial analysis performing BOLT-LMM analysis on European ancestry individuals in the UKB cohort, we identified 17 significant associations ($p<3.4x10^{-10}$) in the inclusive CADD model and 26 in the restrictive CADD model. All of these associations were also statistically significant in the European ancestry BOLT-LMM meta-analysis coding or LoF models used in the main paper. In contrast, nearly half of the significant results from the main UKB European ancestry BOLT-LMM coding and LoF models were not found among the significant CADD model associations. Because these additional models did not provide new insights, we did not include these analysis results in the main manuscript.

SKAT analysis

We also performed a gene-based SKAT analysis of the unrelated European ancestry individuals [4]. We performed this analysis in Hail, with accuracy set at $1x10^{-11}$ and $1x10^{9}$ iterations and using age, sex, and 10 European-specific principal components as covariates [5]. We used two models: a basic SKAT model with weights based on beta (1,25) of the MAF and using the same set of coding variants from our main gene-based collapsing analysis (MAF model); and a SKAT model with weights of beta (1,25) x Phred-scaled CADD score that used all coding variants with MAF<0.1% (MAF-CADD model). The MAF-CADD model included 2,000,908 variants for the UKB cohort and 1,427,500 for the HNP, approximately twice as many as the MAF model.

We started with the set of unrelated European ancestry individuals from the UKB cohort as a discovery set. We ran the full set of 4,264 phenotypes and restricted to results where at least five individuals were variant carriers for quantitative traits, or five expected case carriers based on the overall prevalence for binary traits. We ran linear regression for quantitative traits and logistic regression for binary traits. We identified 464 statistically significant results ($p<3.4x10^{-10}$) for the MAF model and 1,331 for the MAF-CADD model. Of these, 235 and 778 respectively had data available for replication in the HNP cohort. When we attempted to replicate the findings, we found that respectively only 8.1% of the 8.6% associations generated a statistically significant result (p<0.0002, Bonferroni correction for 235 tests, and $p<6.4x10^{-5}$, correction for 788 tests). This is in contrast to 46% formal replication in our main gene-based collapsing

analysis, as described in the main text. Furthermore, if we chose a more lenient replication p-value cutoff that would confirm at least the suggestion of replication (p<0.05), we found that respectively only 24.2% and 24.4% of associations replicated, compared to 82% in our main gene-based collapsing analysis as described in the main manuscript. In our main gene-based collapsing analysis, 100% of signals showed at least the same direction of effect in the replication cohort, but this statistic cannot be analyzed in the context of a SKAT analysis as there is no main direction of effect provided.

Even when we were more stringent about which SKAT analyses to include as significant, the replication rate was very low. For example, when we required the significant binary associations to have at least 10 expected case carriers based on the overall frequency, for the MAF model, only 25% of the 32 eligible associations replicated (p<0.0015, correction for 32 tests), and only 34% had uncorrected p<0.05. For the MAF-CADD model, only 7.3% of the 82 eligible associations replicated (p<0.0006, correction for 82 tests), and only 24.4% had uncorrected p<0.05.

Our results coincide with a number of previous studies documenting that the results of SKAT analyses in real-world datasets can often be noisy and difficult to interpret[6,7]. Given these difficulties, we did not include these analysis results in our main manuscript.

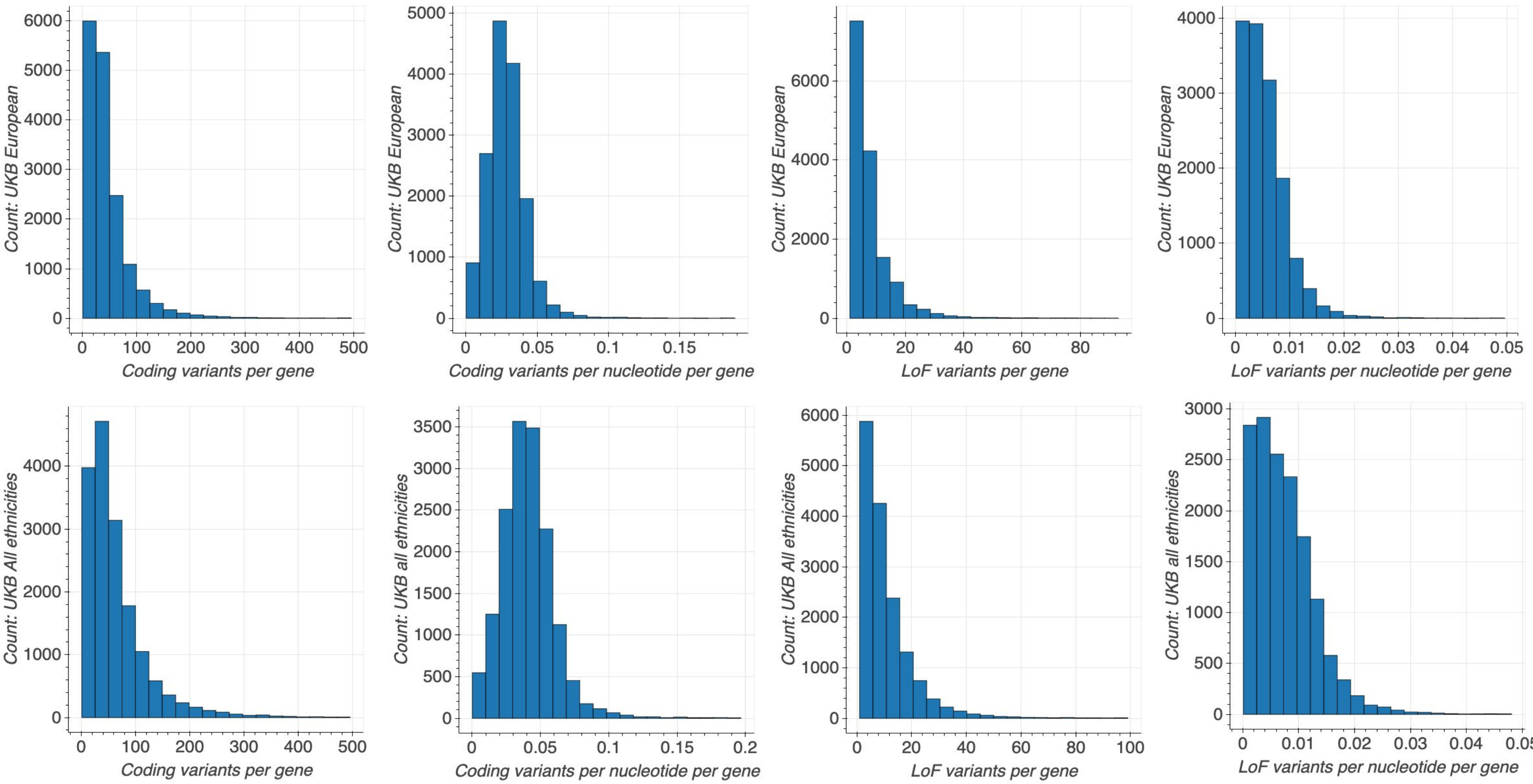
Count: UKB European
Coding variants per gene
0
100
200
300
400
500
0
1000
2000
3000
4000
5000
6000
Count: UKB European
Coding variants per nucleotide per gene
0
0.05
0.1
0.15
0
1000
2000
3000
4000
5000
Count: UKB European
LoF variants per gene
0
20
40
60
80
0
2000
4000
6000
Count: UKB European
LoF variants per nucleotide per gene
0
0.01
0.02
0.03
0.04
0.05
0
1000
2000
3000
4000
Count: UKB All ethnicities
Coding variants per gene
0
100
200
300
400
500
0
1000
2000
3000
4000
Count: UKB all ethnicities
Coding variants per nucleotide per gene
0
0.05
0.1
0.15
0.2
0
500
1000
1500
2000
2500
3000
3500
Count: UKB All ethnicities
LoF variants per gene
0
20
40
60
80
100
0
1000
2000
3000
4000
5000
6000
Count: UKB all ethnicities
LoF variants per nucleotide per gene
0
0.01
0.02
0.03
0.04
0.05
0
500
1000
1500
2000
2500
3000

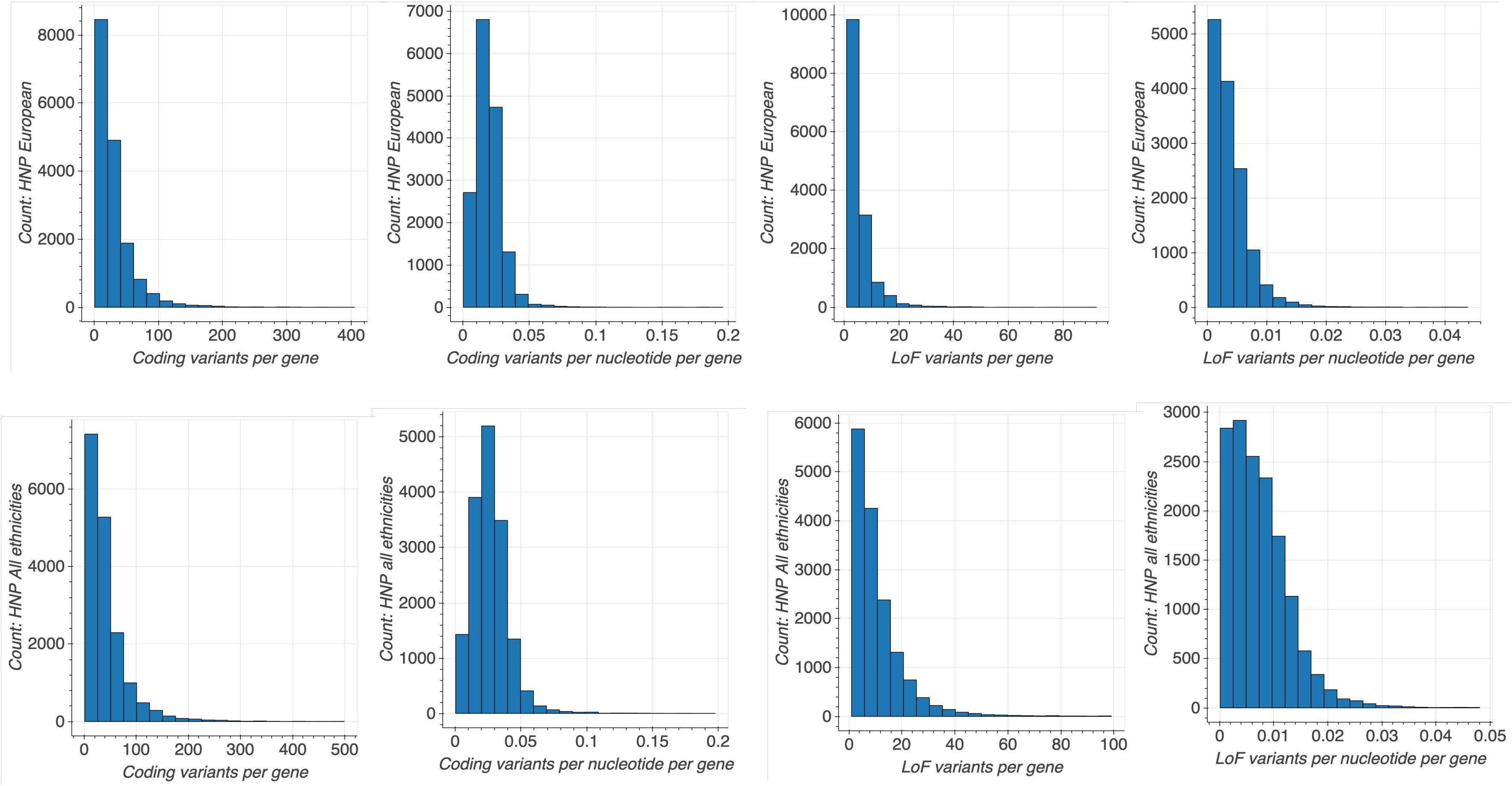

Count: HNP European
8000
6000
4000
2000
0
0 100 200 300 400
Coding variants per gene
Count: HNP European
7000
6000
5000
4000
3000
2000
1000
0
0 0.05 0.1 0.15 0.2
Coding variants per nucleotide per gene
Count: HNP European
10000
8000
6000
4000
2000
0
0 20 40 60 80
LoF variants per gene
Count: HNP European
5000
4000
3000
2000
1000
0
0 0.01 0.02 0.03 0.04
LoF variants per nucleotide per gene
Count: HNP All ethnicities
6000
4000
2000
0
0 100 200 300 400 500
Coding variants per gene
Count: HNP all ethnicities
5000
4000
3000
2000
1000
0
0 0.05 0.1 0.15 0.2
Coding variants per nucleotide per gene
Count: HNP All ethnicities
6000
5000
4000
3000
2000
1000
0
0 20 40 60 80 100
LoF variants per gene
Count: HNP all ethnicities
3000
2500
2000
1500
1000
500
0
0 0.01 0.02 0.03 0.04 0.05
LoF variants per nucleotide per gene

Supplementary Figure 1. Histograms of number of qualifying variants per gene. Each histogram is labeled to indicate whether it is UKB or HNP cohort, European ancestry only or all ancestries, coding or LoF model, and number of qualifying variants per gene or number of qualifying variants per coding nucleotide of each gene. Between 1 and 42 genes were excluded from each plot as they were out of range.

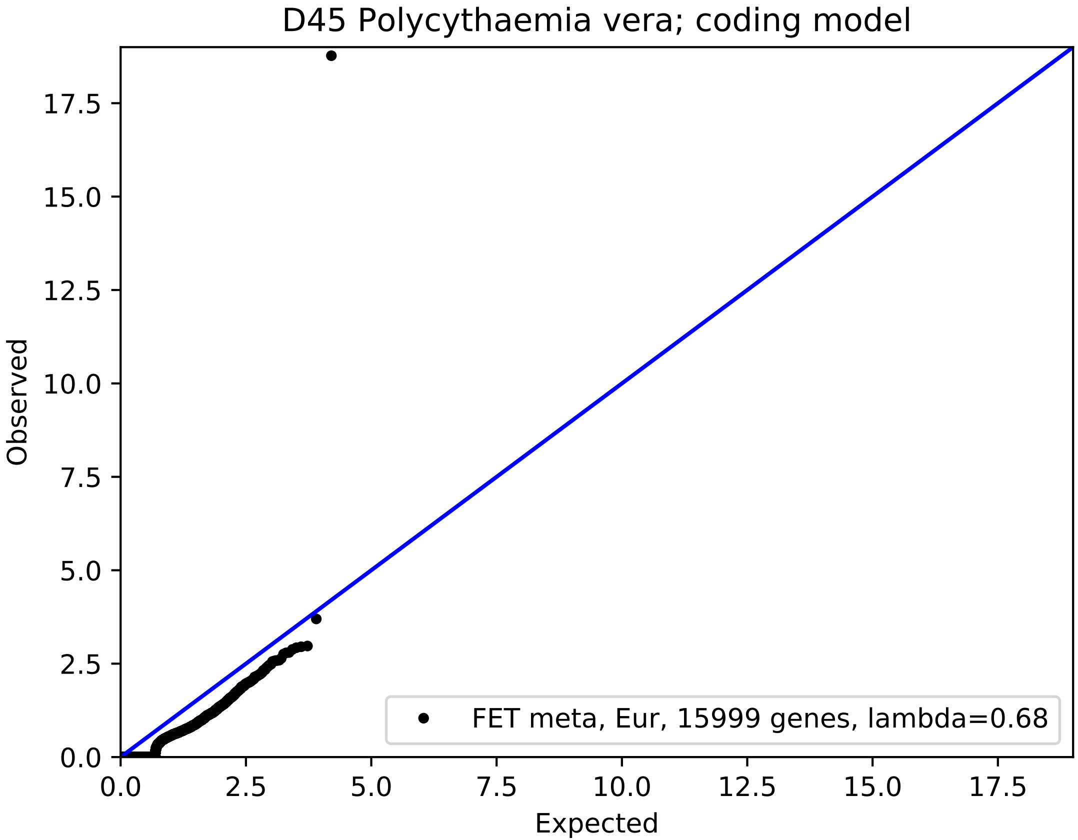

D45 Polycythaemia vera; coding model
Observed
Expected
0.0
2.5
5.0
7.5
10.0
12.5
15.0
17.5
FET meta, Eur, 15999 genes, lambda=0.68

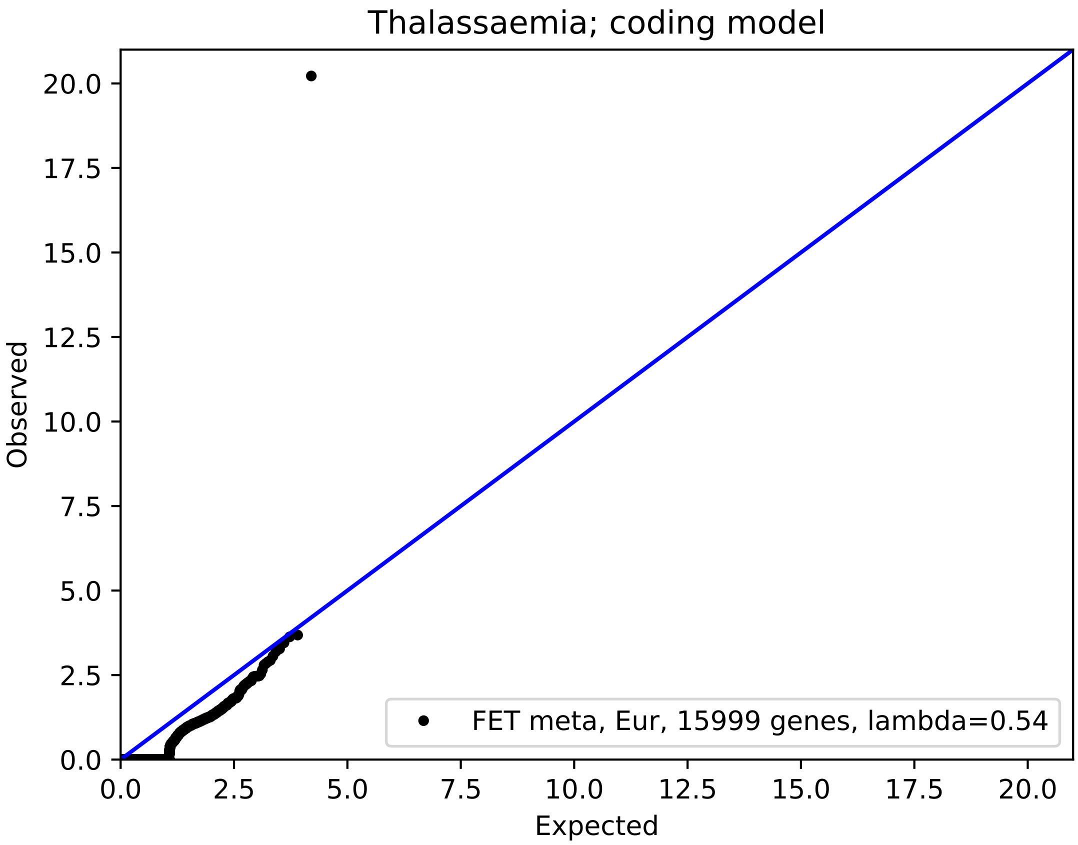

Thalassaemia; coding model
Observed
Expected
0.0
2.5
5.0
7.5
10.0
12.5
15.0
17.5
20.0
FET meta, Eur, 15999 genes, lambda=0.54

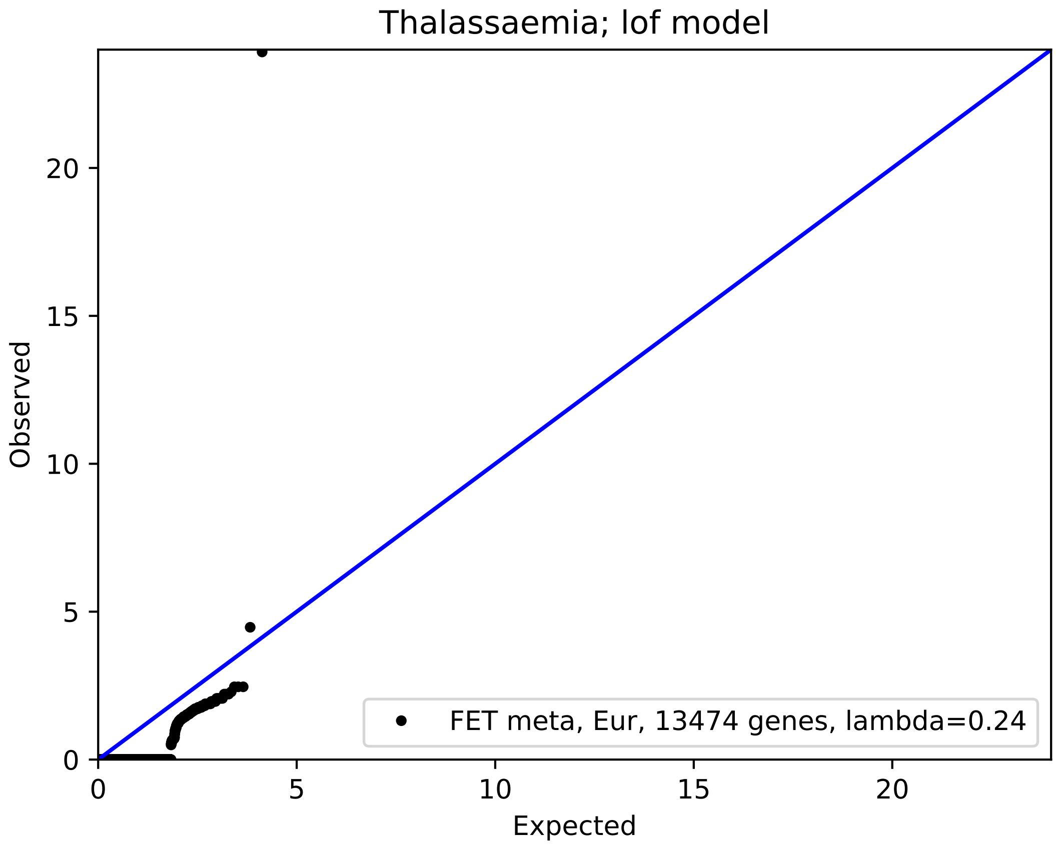
Thalassaemia; lof model
Observed
Expected
0
5
10
15
20
FET meta, Eur, 13474 genes, lambda=0.24

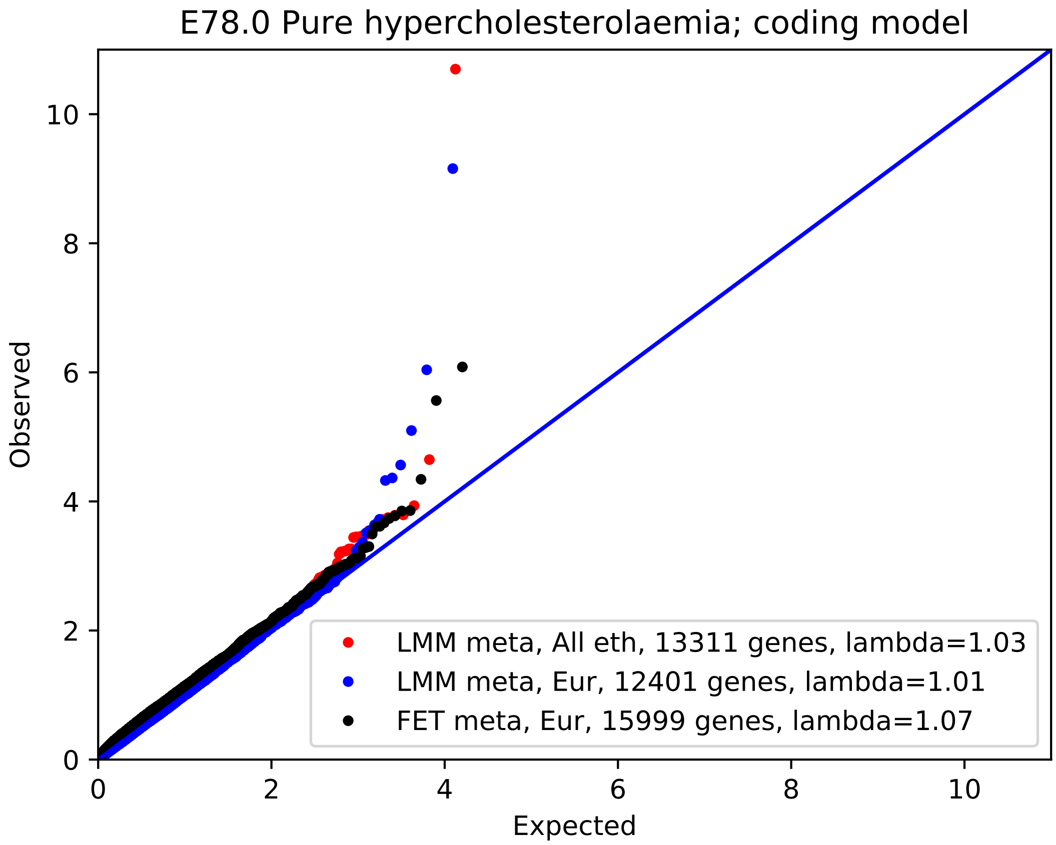
E78.0 Pure hypercholesterolaemia; coding model
Observed
Expected
0
2
4
6
8
10
LMM meta, All eth, 13311 genes, lambda=1.03
LMM meta, Eur, 12401 genes, lambda=1.01
FET meta, Eur, 15999 genes, lambda=1.07

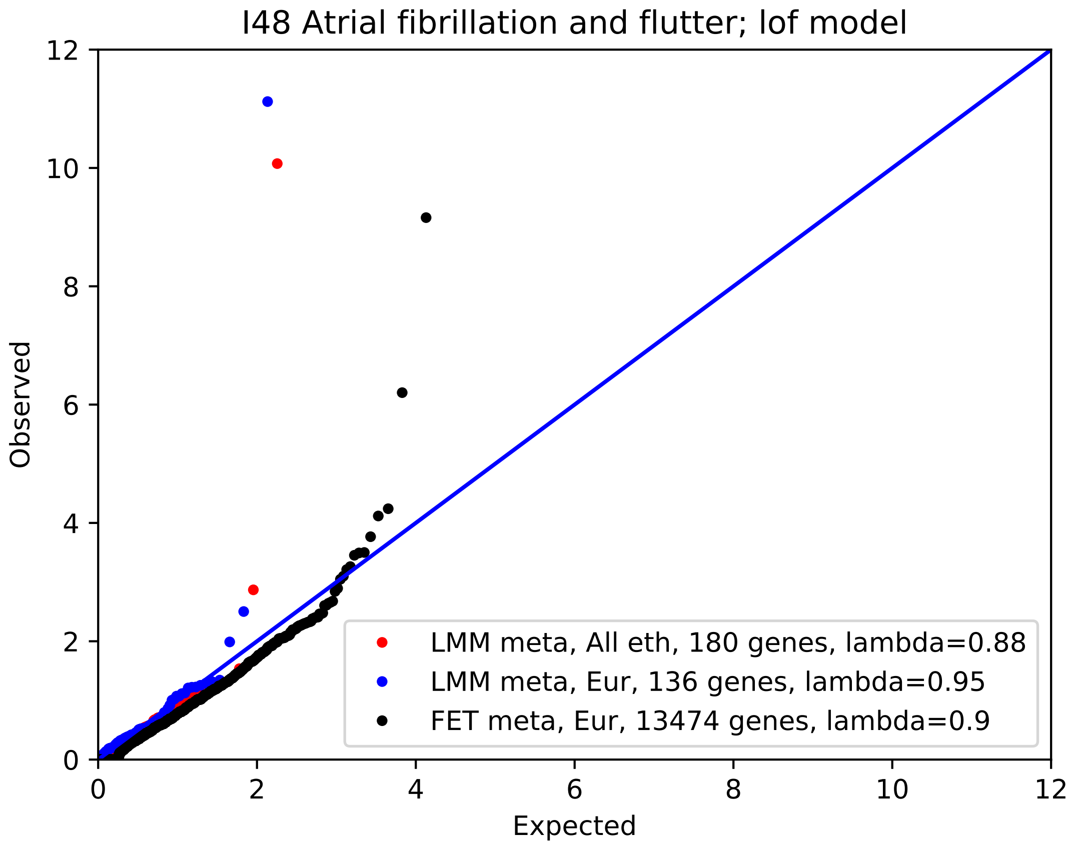
I48 Atrial fibrillation and flutter; lof model
Observed
Expected
0
2
4
6
8
10
12
LMM meta, All eth, 180 genes, lambda=0.88
LMM meta, Eur, 136 genes, lambda=0.95
FET meta, Eur, 13474 genes, lambda=0.9

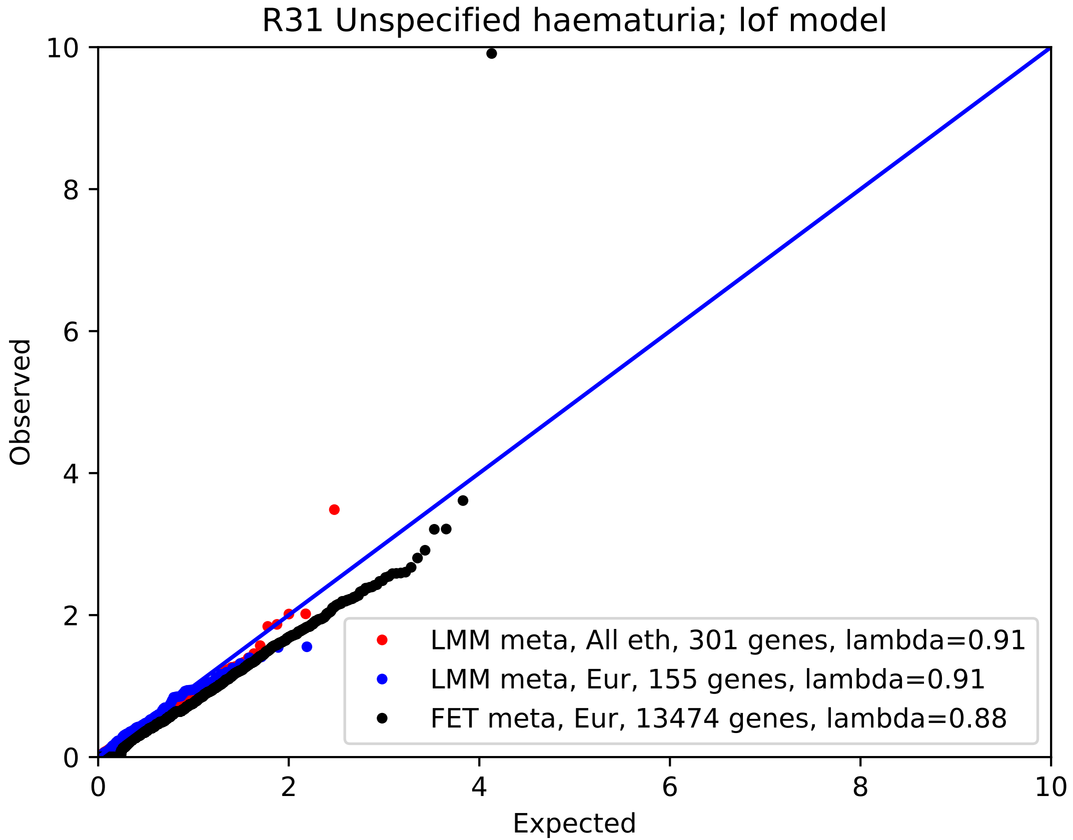
R31 Unspecified haematuria; lof model
Observed
Expected
0
2
4
6
8
10
LMM meta, All eth, 301 genes, lambda=0.91
LMM meta, Eur, 155 genes, lambda=0.91
FET meta, Eur, 13474 genes, lambda=0.88

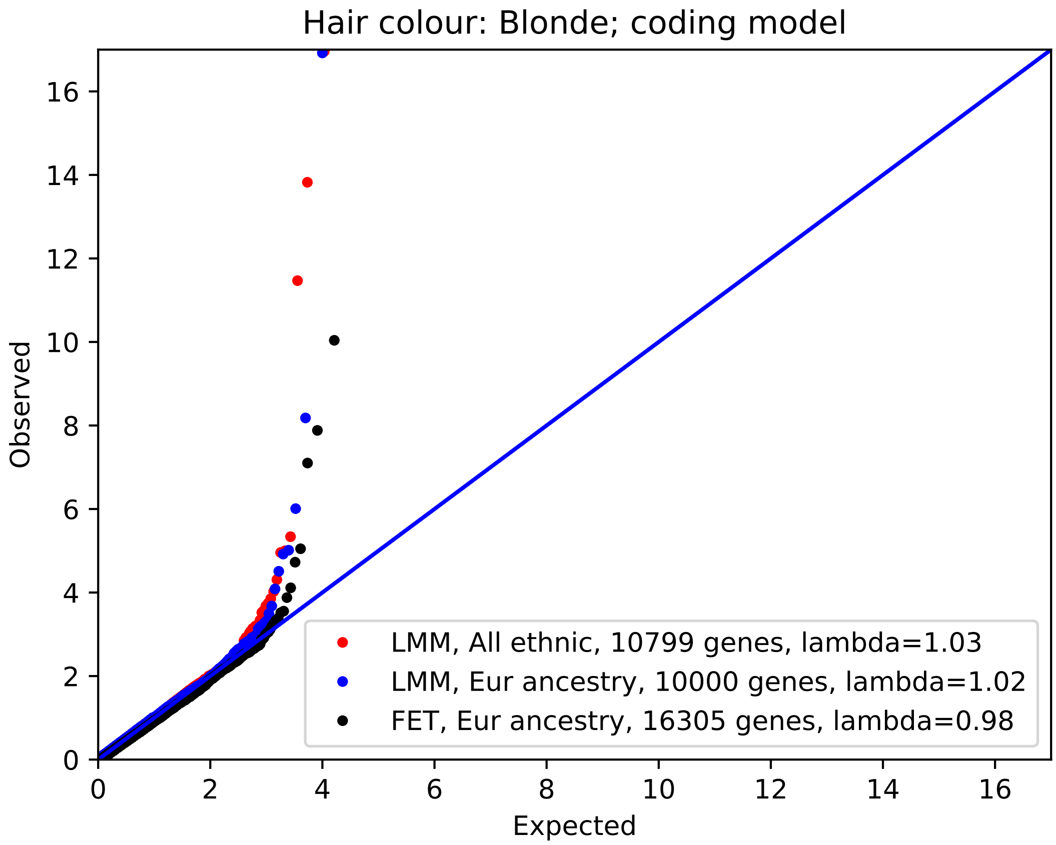
Hair colour: Blonde; coding model
Observed
Expected
0
2
4
6
8
10
12
14
16
LMM, All ethnic, 10799 genes, lambda=1.03
LMM, Eur ancestry, 10000 genes, lambda=1.02
FET, Eur ancestry, 16305 genes, lambda=0.98

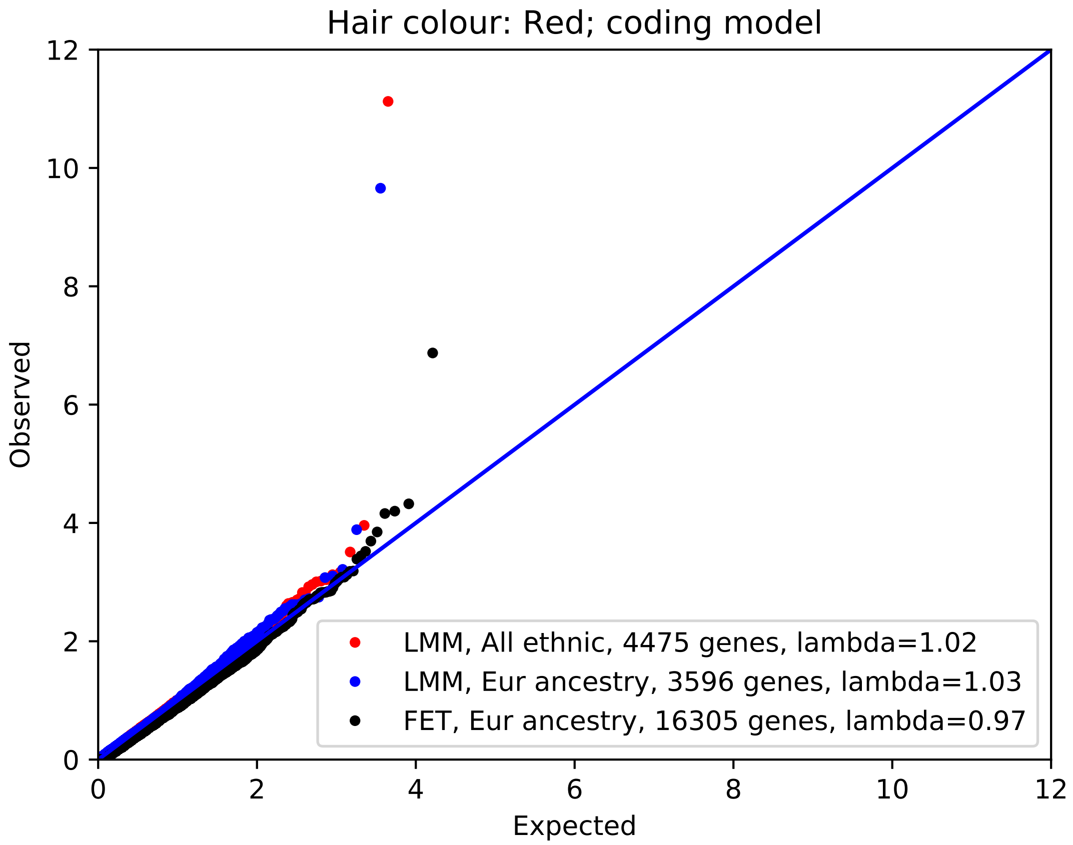

Hair colour: Red; coding model
Observed
Expected
0
2
4
6
8
10
12
LMM, All ethnic, 4475 genes, lambda=1.02
LMM, Eur ancestry, 3596 genes, lambda=1.03
FET, Eur ancestry, 16305 genes, lambda=0.97

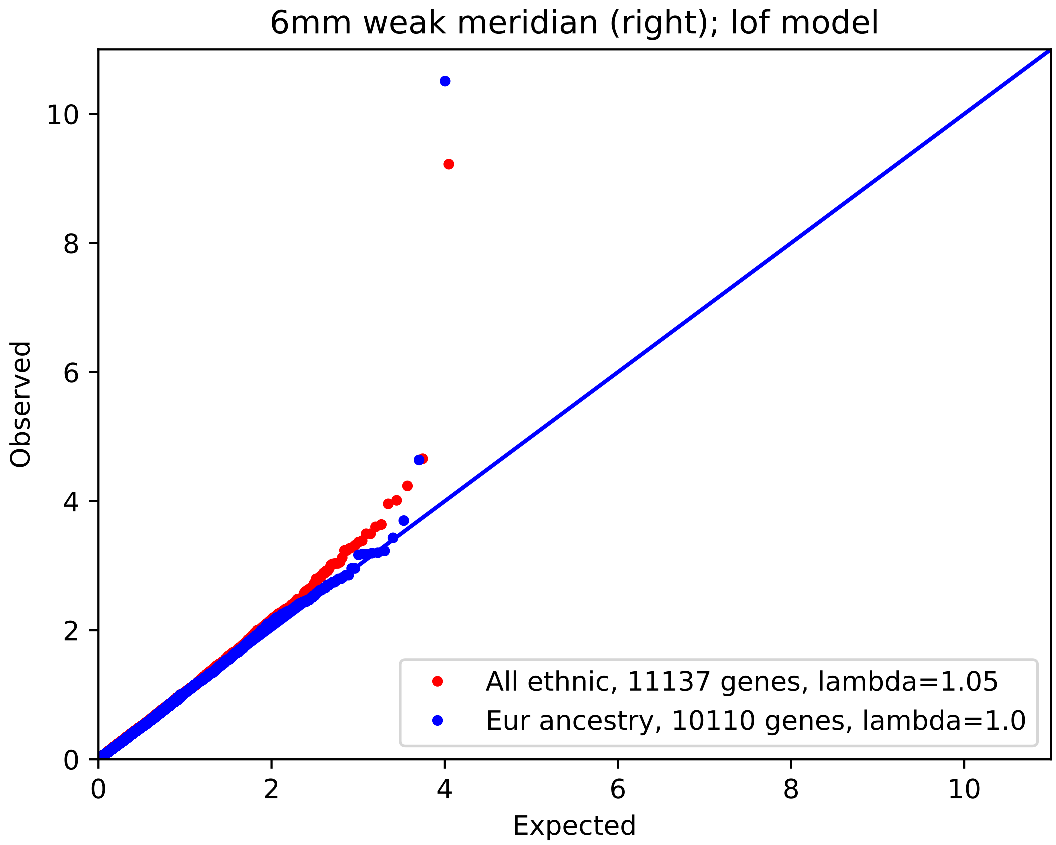

6mm weak meridian (right); lof model
Observed
Expected
0
2
4
6
8
10
0
2
4
6
8
10
All ethnic, 11137 genes, lambda=1.05
Eur ancestry, 10110 genes, lambda=1.0

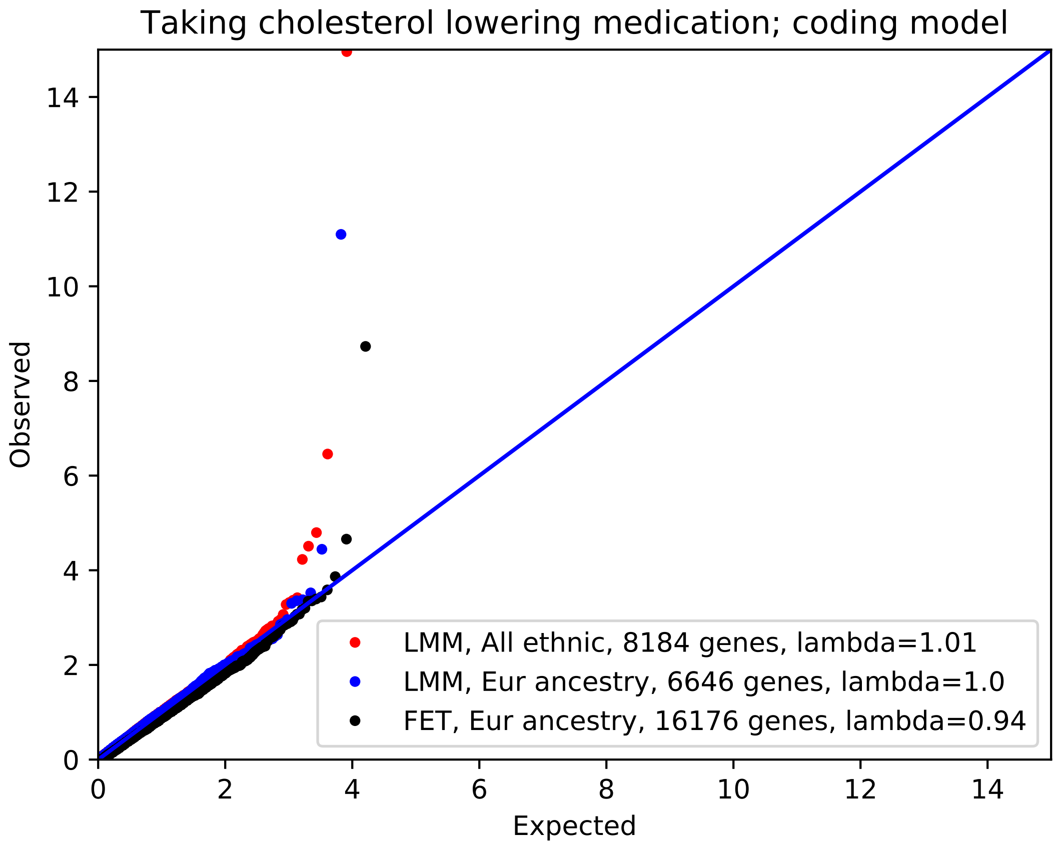

Taking cholesterol lowering medication; coding model
Observed
Expected
0
2
4
6
8
10
12
14
LMM, All ethnic, 8184 genes, lambda=1.01
LMM, Eur ancestry, 6646 genes, lambda=1.0
FET, Eur ancestry, 16176 genes, lambda=0.94

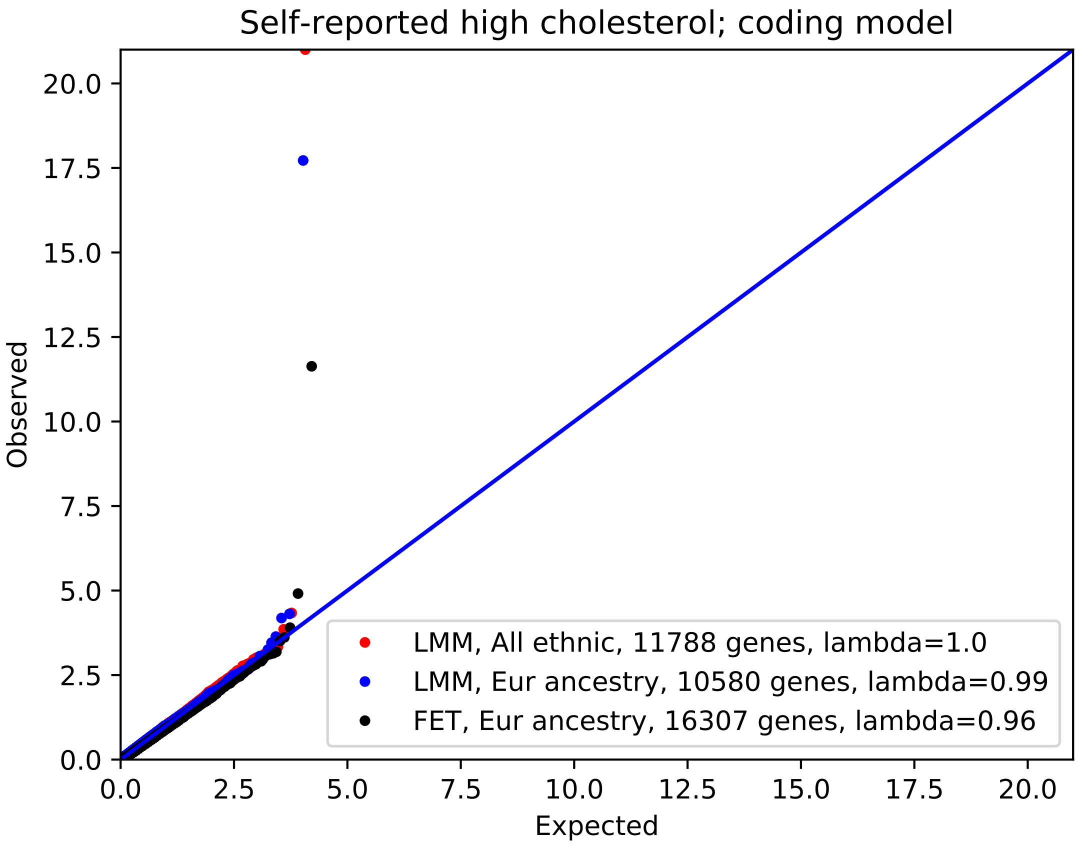

Self-reported high cholesterol; coding model
Observed
Expected
0.0
2.5
5.0
7.5
10.0
12.5
15.0
17.5
20.0
LMM, All ethnic, 11788 genes, lambda=1.0
LMM, Eur ancestry, 10580 genes, lambda=0.99
FET, Eur ancestry, 16307 genes, lambda=0.96

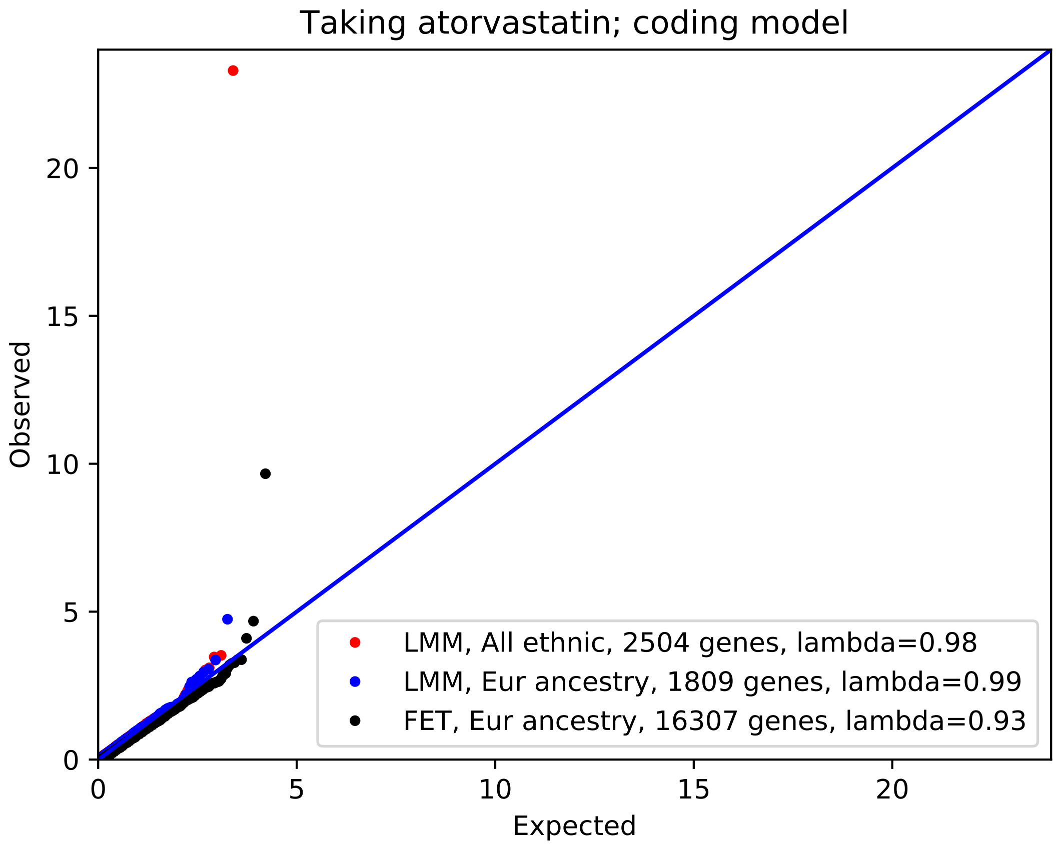

Taking atorvastatin; coding model
Observed
Expected
0
5
10
15
20
LMM, All ethnic, 2504 genes, lambda=0.98
LMM, Eur ancestry, 1809 genes, lambda=0.99
FET, Eur ancestry, 16307 genes, lambda=0.93

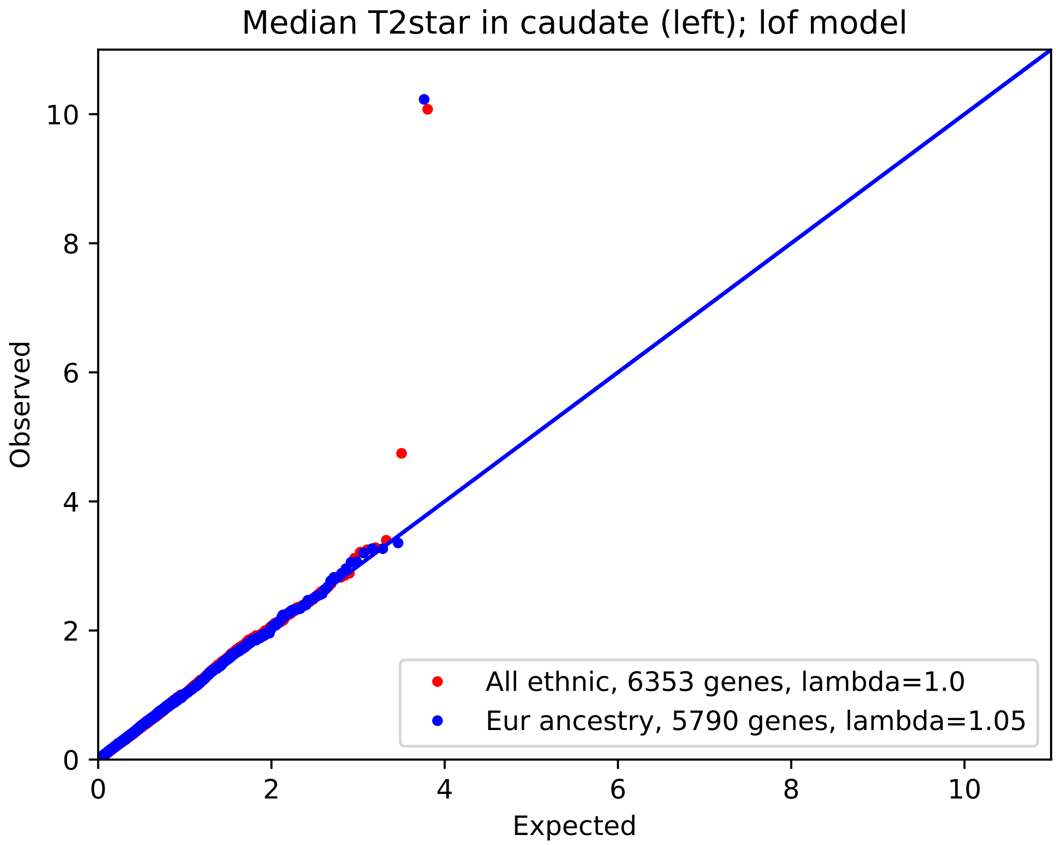

Median T2star in caudate (left); lof model
Observed
Expected
0
2
4
6
8
10
All ethnic, 6353 genes, lambda=1.0
Eur ancestry, 5790 genes, lambda=1.05

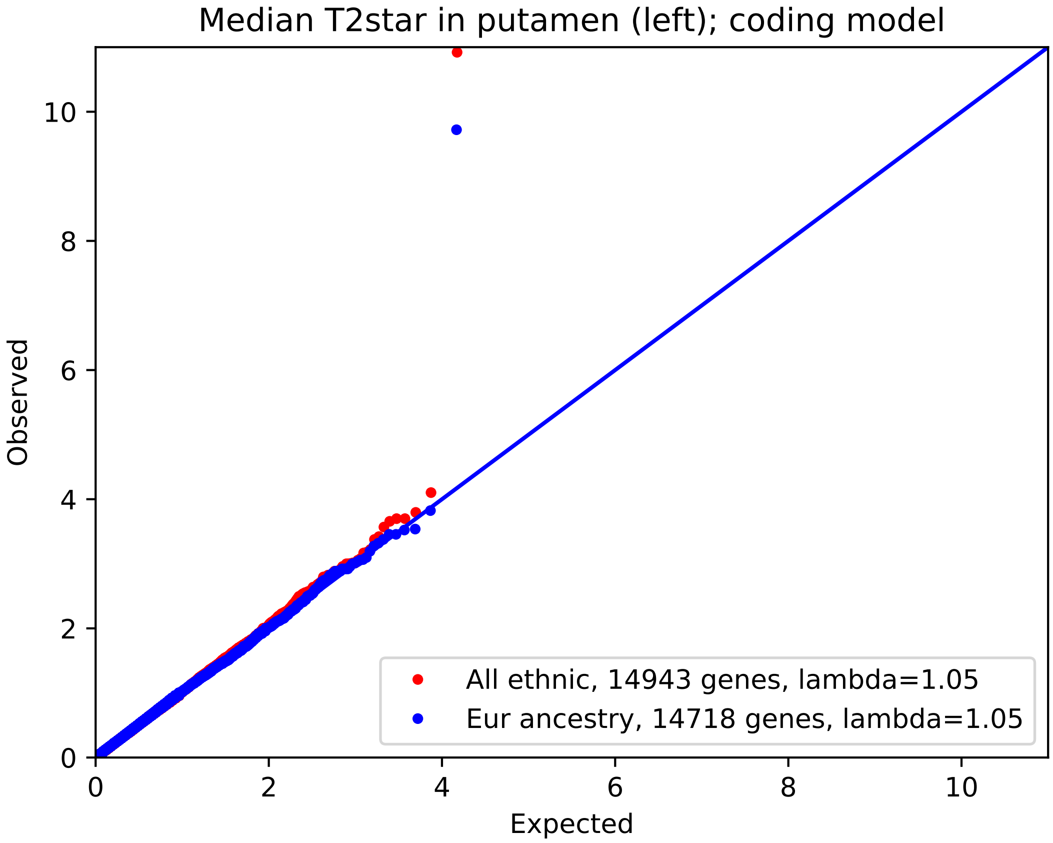
Median T2star in putamen (left); coding model
Observed
Expected
0
2
4
6
8
10
All ethnic, 14943 genes, lambda=1.05
Eur ancestry, 14718 genes, lambda=1.05

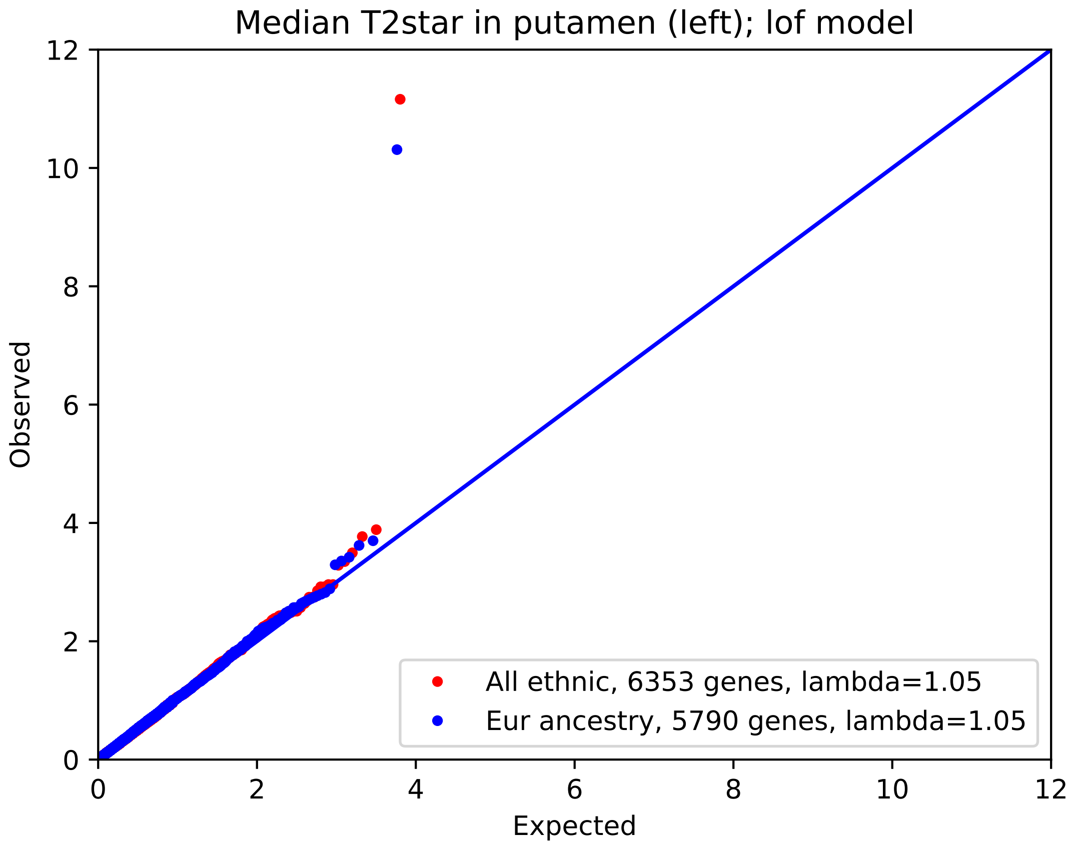

Median T2star in putamen (left); lof model
Observed
Expected
0
2
4
6
8
10
12
All ethnic, 6353 genes, lambda=1.05
Eur ancestry, 5790 genes, lambda=1.05

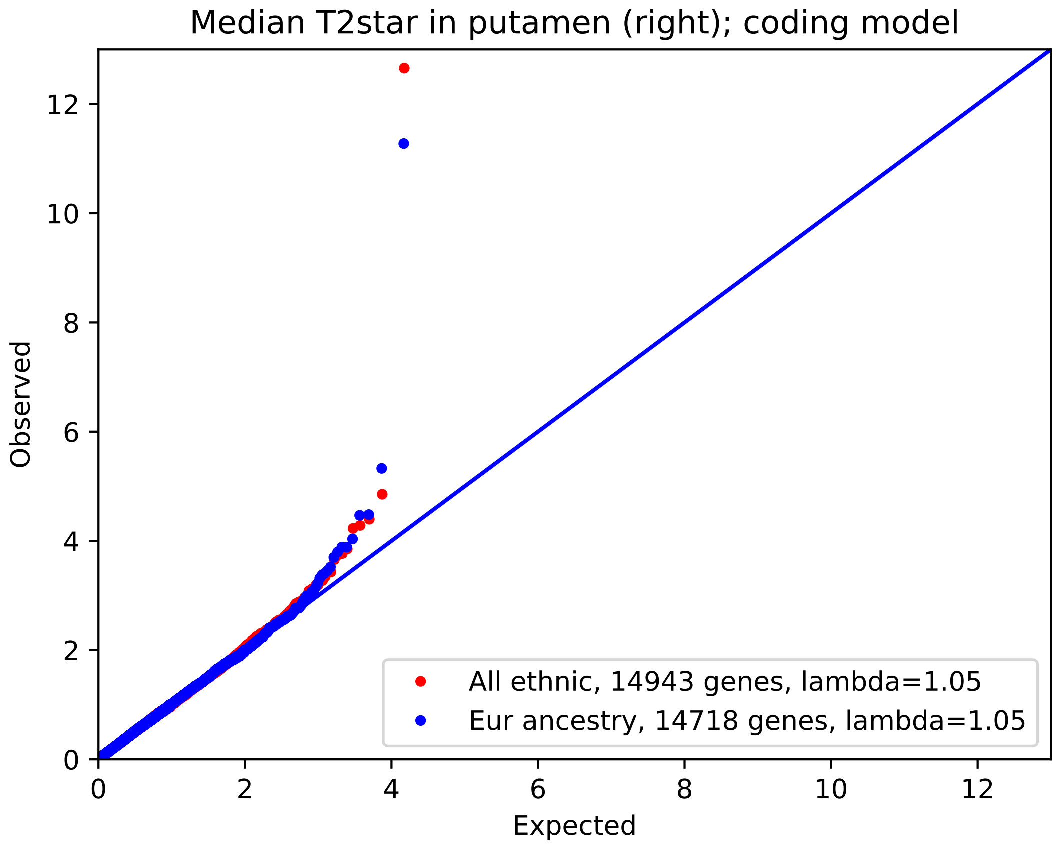
Median T2star in putamen (right); coding model
Observed
Expected
0
2
4
6
8
10
12
All ethnic, 14943 genes, lambda=1.05
Eur ancestry, 14718 genes, lambda=1.05

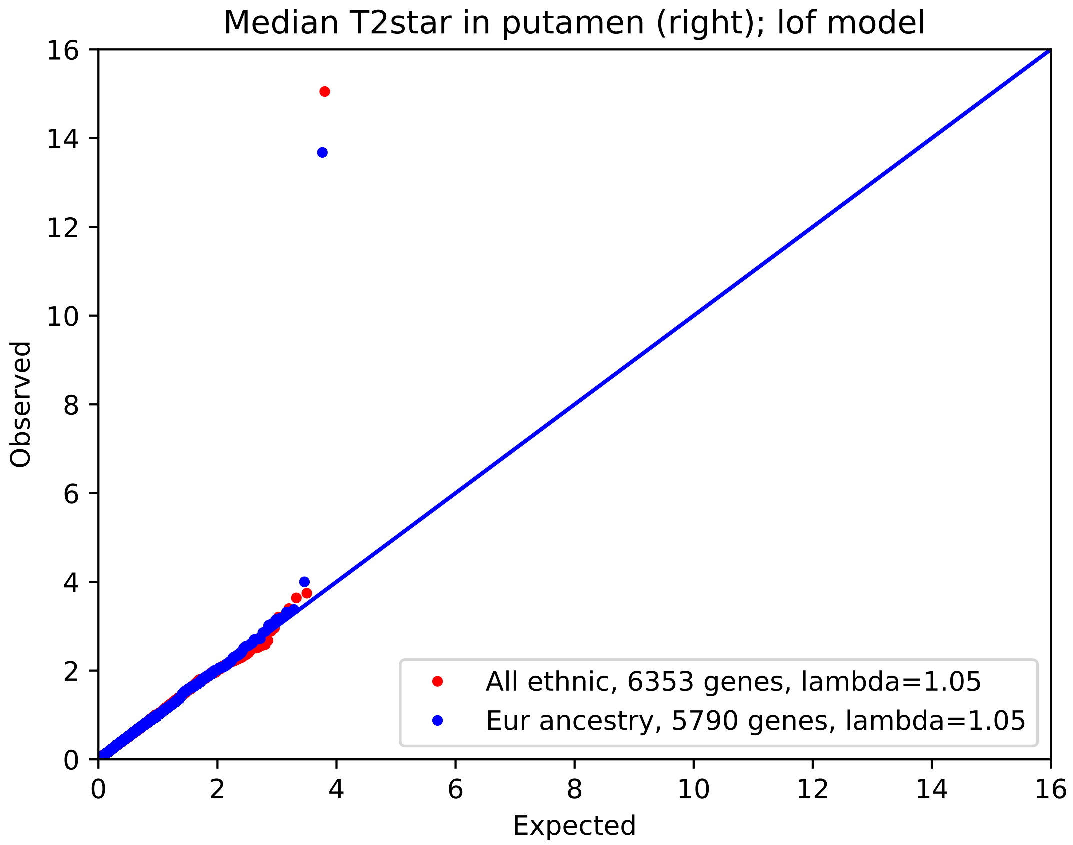
Median T2star in putamen (right); lof model
Observed
Expected
0
2
4
6
8
10
12
14
16
All ethnic, 6353 genes, lambda=1.05
Eur ancestry, 5790 genes, lambda=1.05

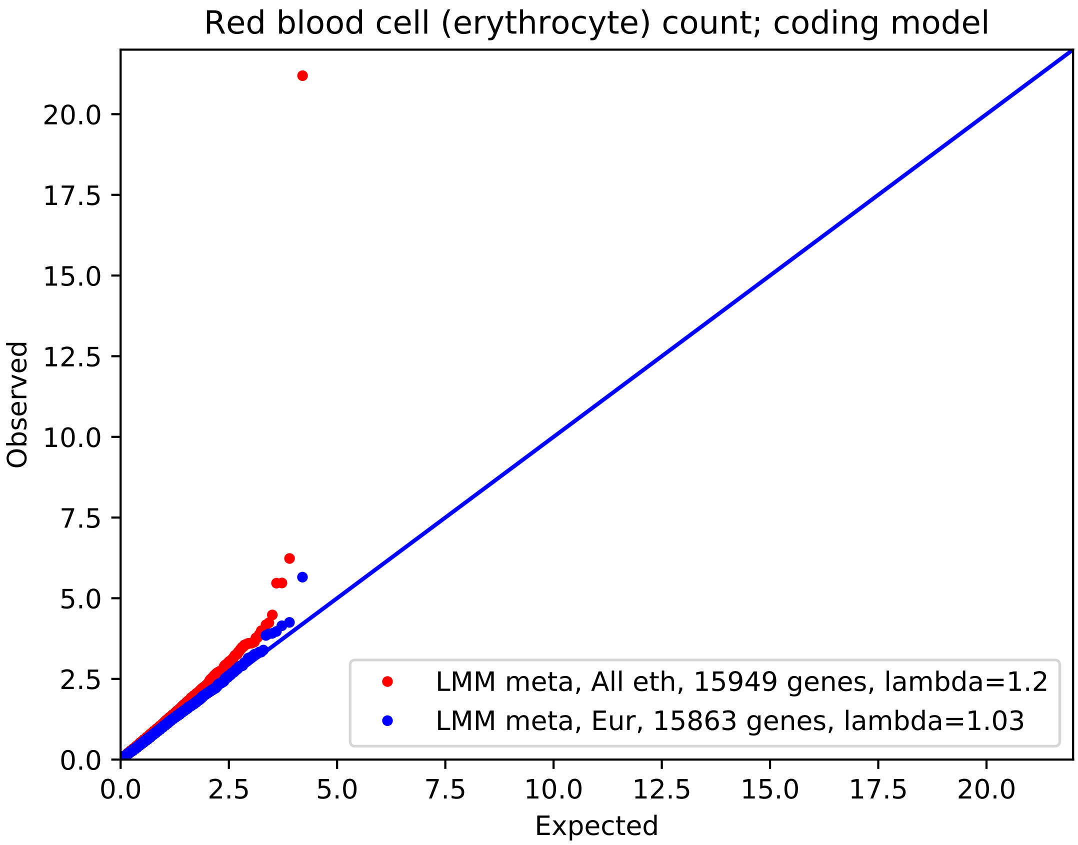

Red blood cell (erythrocyte) count; coding model
Observed
Expected
0.0
2.5
5.0
7.5
10.0
12.5
15.0
17.5
20.0
LMM meta, All eth, 15949 genes, lambda=1.2
LMM meta, Eur, 15863 genes, lambda=1.03

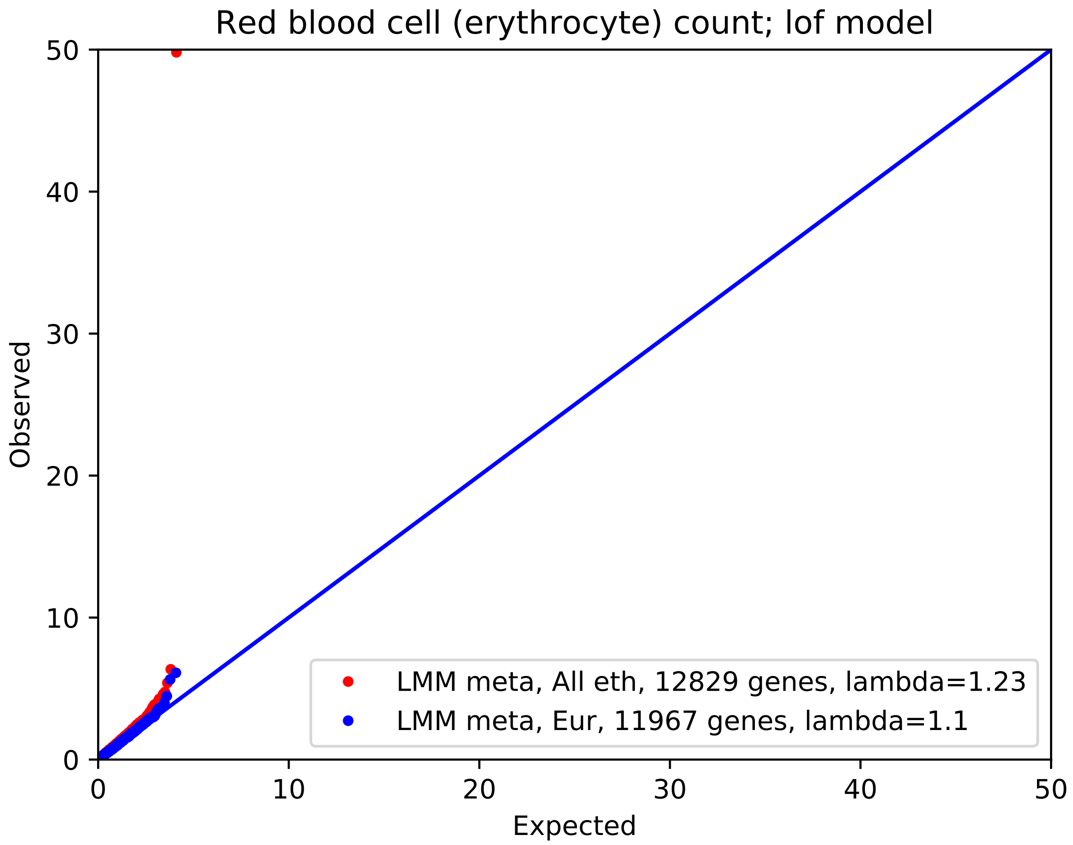

Red blood cell (erythrocyte) count; lof model
Observed
Expected
0
10
20
30
40
50
LMM meta, All eth, 12829 genes, lambda=1.23
LMM meta, Eur, 11967 genes, lambda=1.1

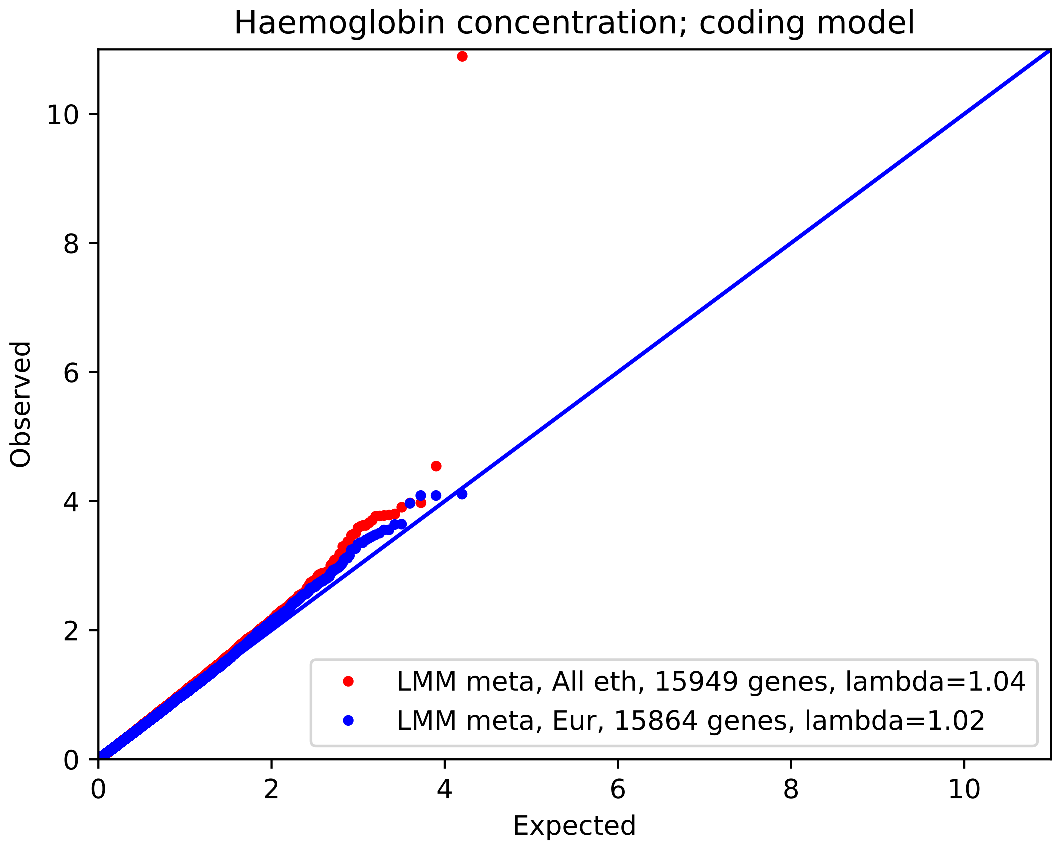
Haemoglobin concentration; coding model
Observed
Expected
0
2
4
6
8
10
LMM meta, All eth, 15949 genes, lambda=1.04
LMM meta, Eur, 15864 genes, lambda=1.02

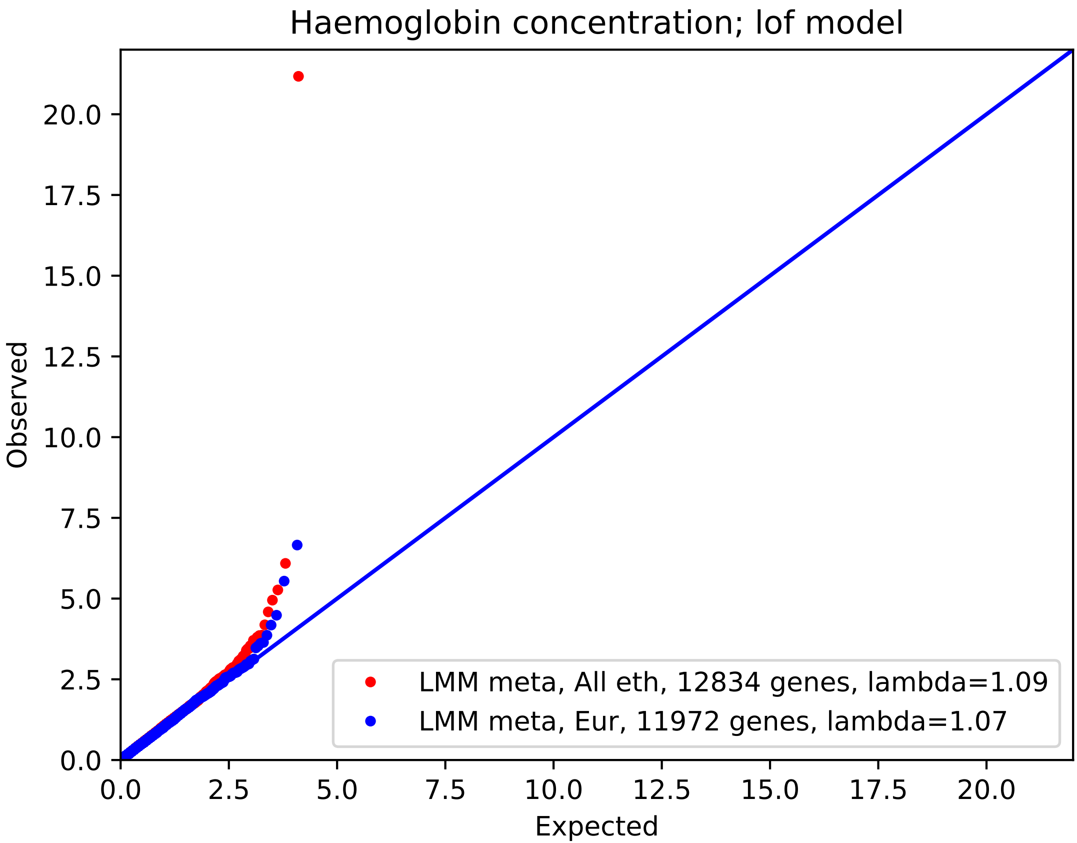

Haemoglobin concentration; lof model
Observed
Expected
0.0
2.5
5.0
7.5
10.0
12.5
15.0
17.5
20.0
LMM meta, All eth, 12834 genes, lambda=1.09
LMM meta, Eur, 11972 genes, lambda=1.07

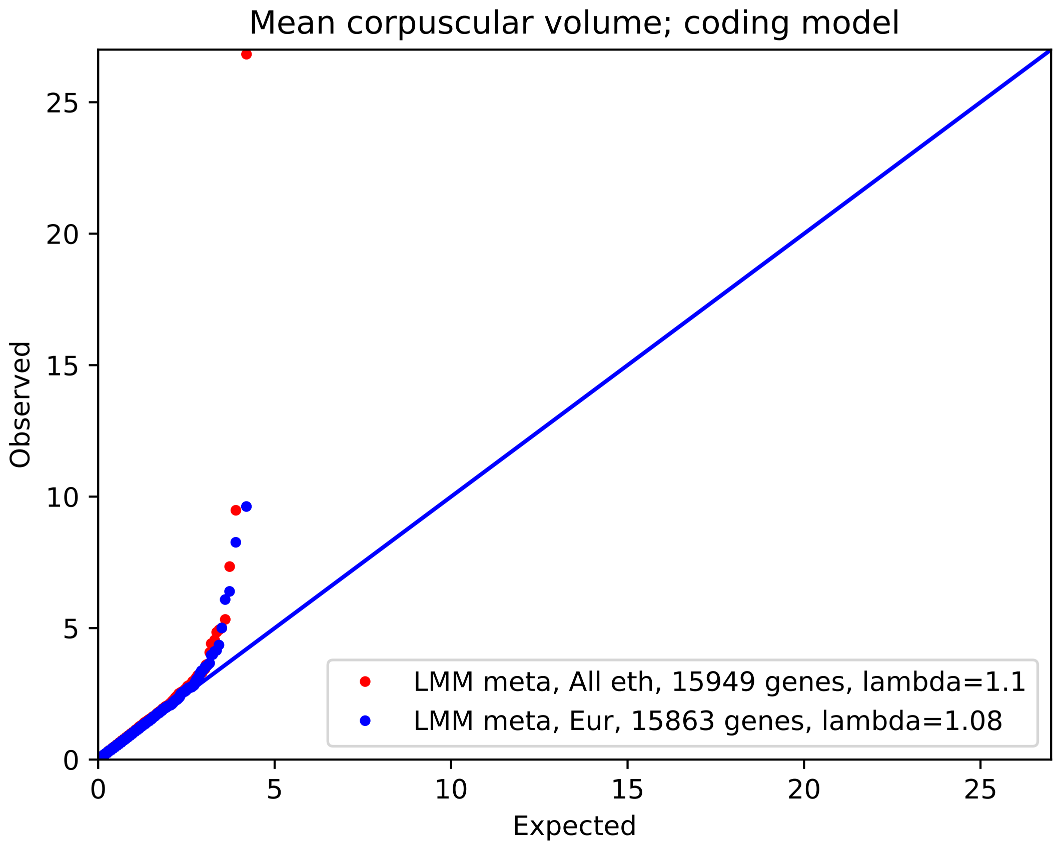

Mean corpuscular volume; coding model
Observed
Expected
0
5
10
15
20
25
LMM meta, All eth, 15949 genes, lambda=1.1
LMM meta, Eur, 15863 genes, lambda=1.08

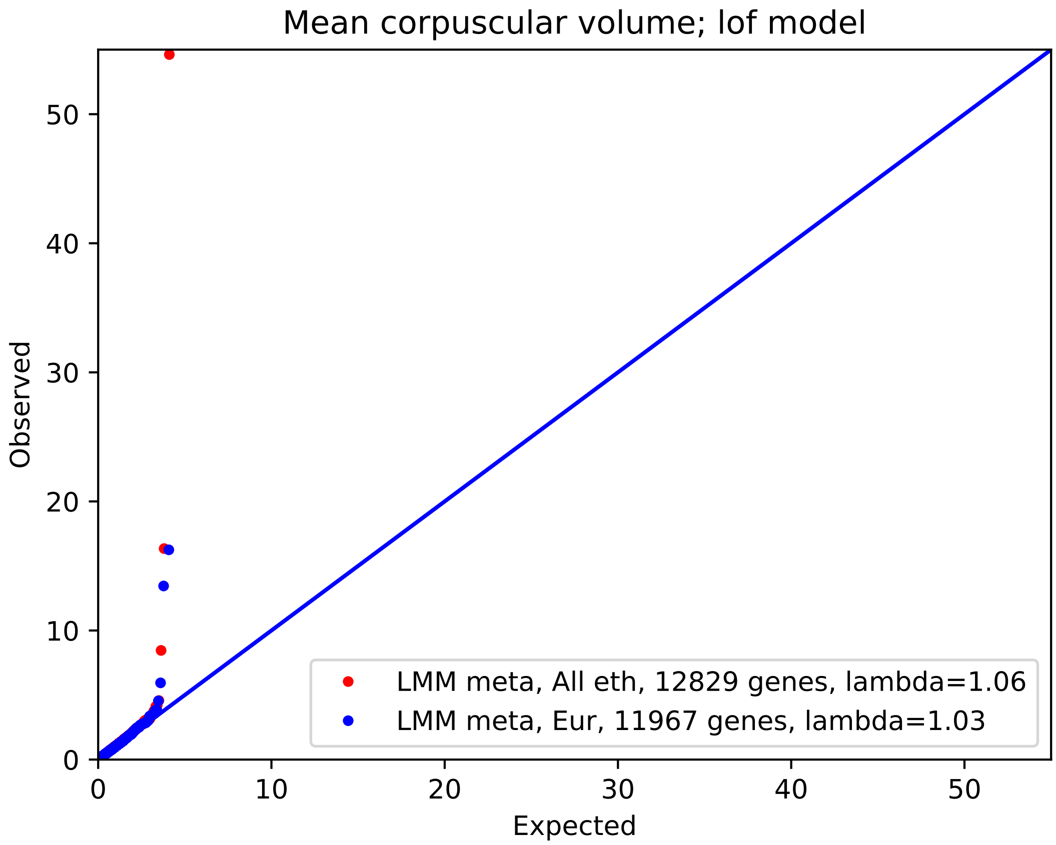

Mean corpuscular volume; lof model
Observed
Expected
0
10
20
30
40
50
LMM meta, All eth, 12829 genes, lambda=1.06
LMM meta, Eur, 11967 genes, lambda=1.03

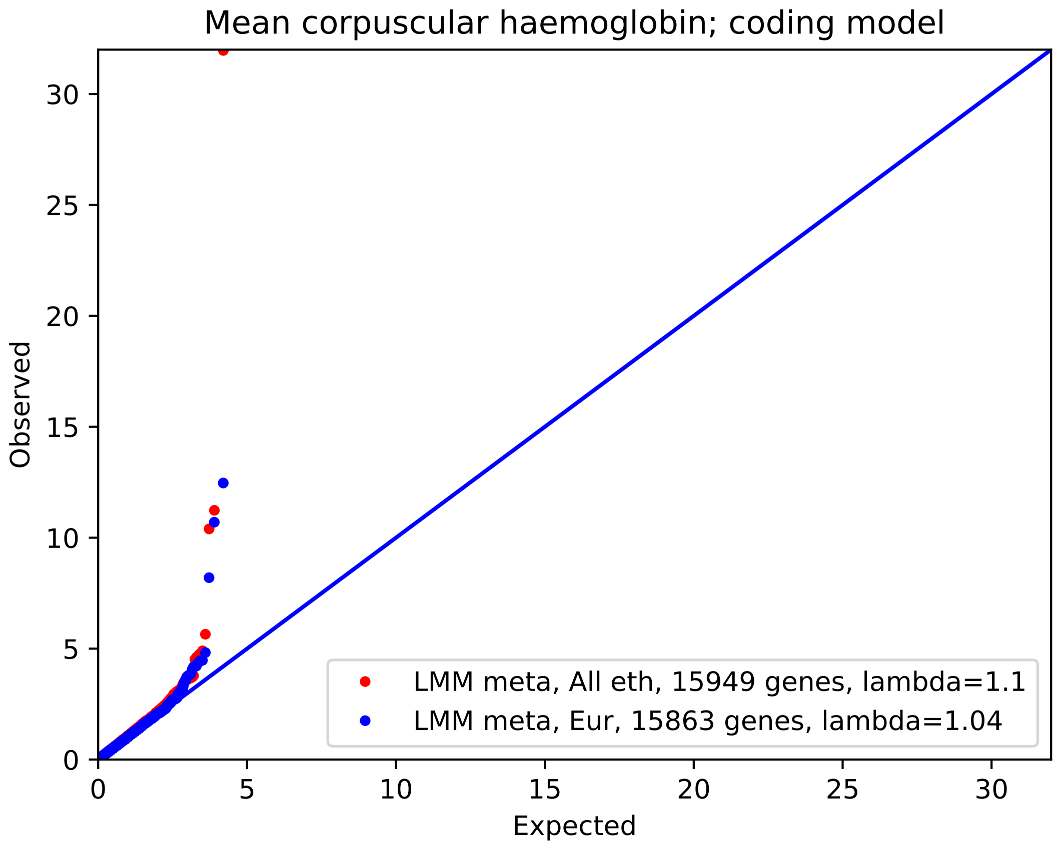

Mean corpuscular haemoglobin; coding model
Observed
Expected
0
5
10
15
20
25
30
LMM meta, All eth, 15949 genes, lambda=1.1
LMM meta, Eur, 15863 genes, lambda=1.04

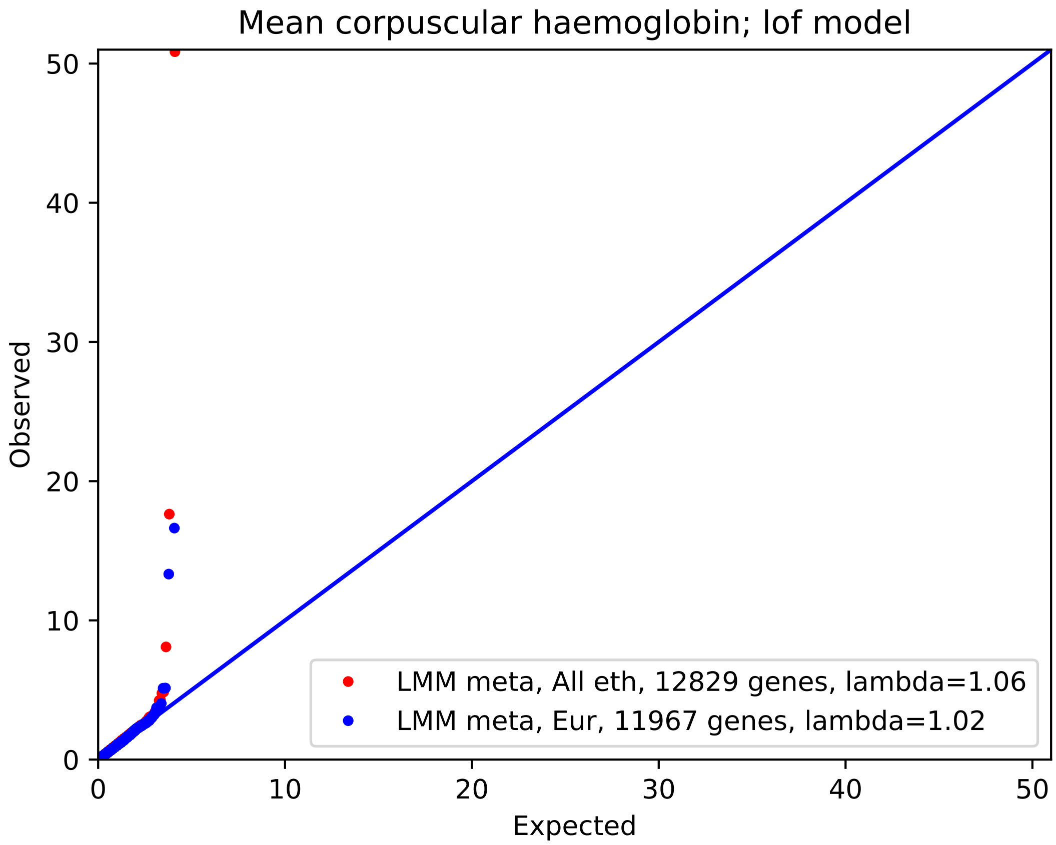
Mean corpuscular haemoglobin; lof model
Observed
Expected
0
10
20
30
40
50
0
10
20
30
40
50
LMM meta, All eth, 12829 genes, lambda=1.06
LMM meta, Eur, 11967 genes, lambda=1.02

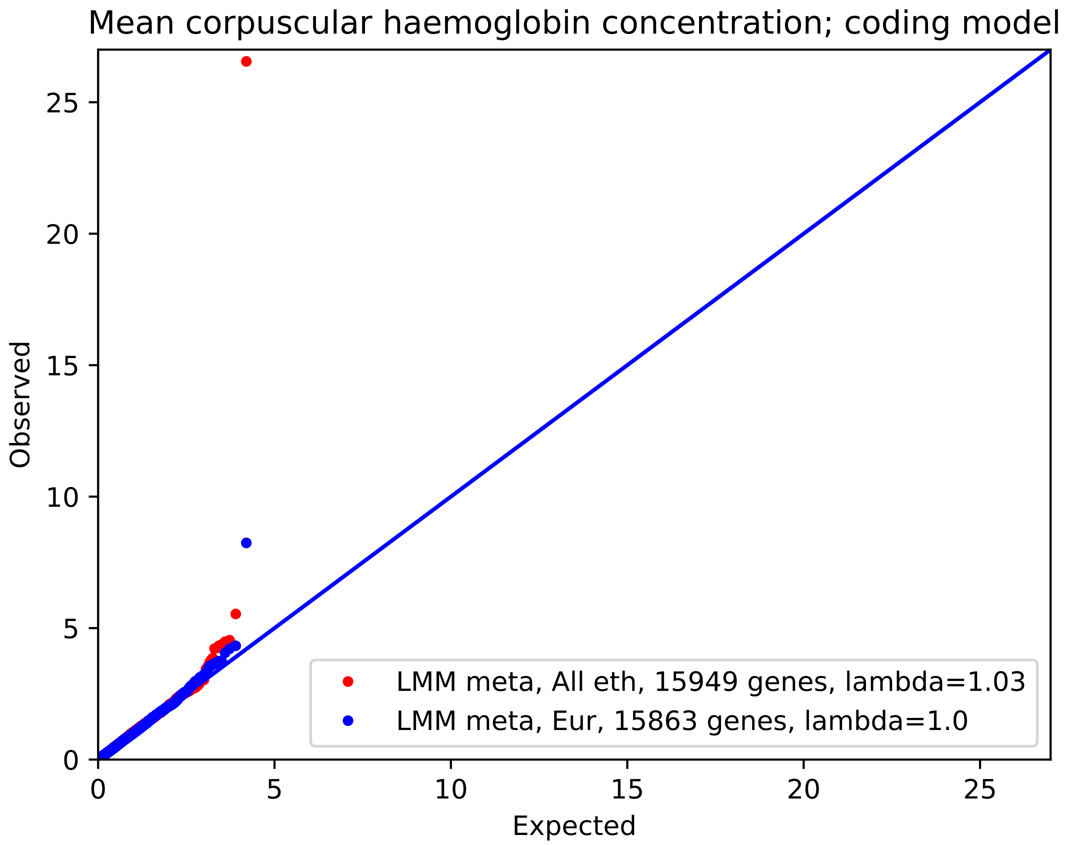
Mean corpuscular haemoglobin concentration; coding model
Observed
Expected
0
5
10
15
20
25
LMM meta, All eth, 15949 genes, lambda=1.03
LMM meta, Eur, 15863 genes, lambda=1.0

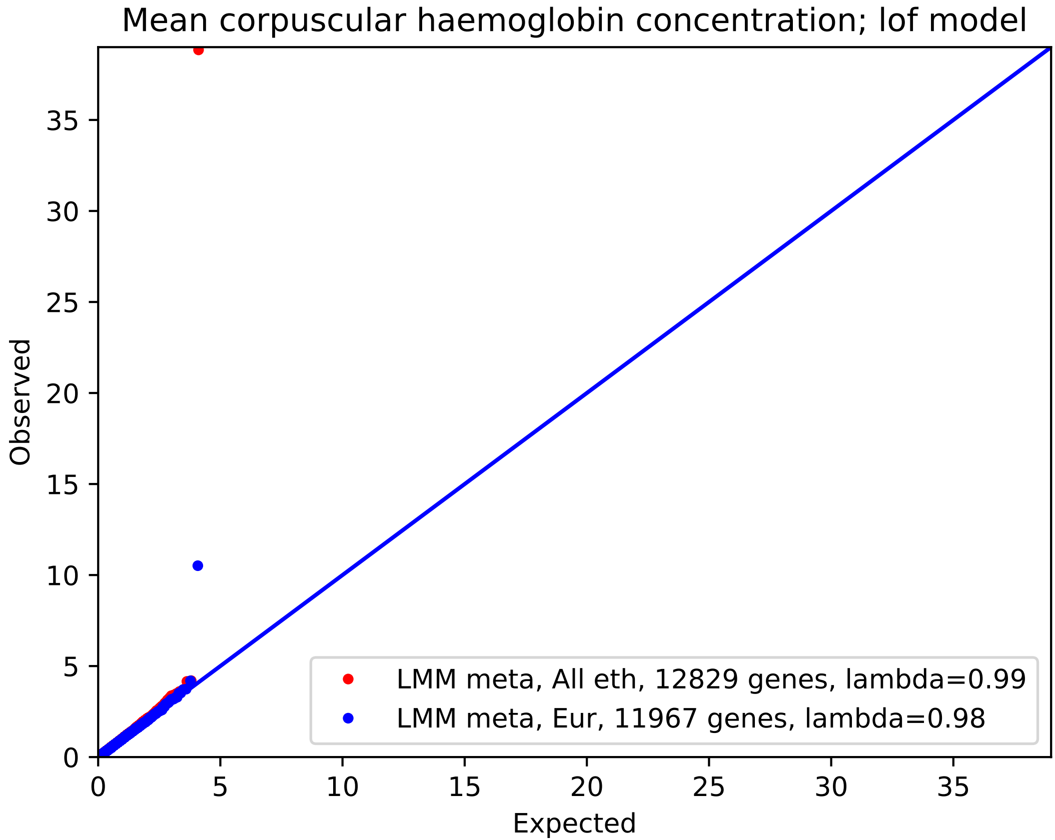

Mean corpuscular haemoglobin concentration; lof model
Observed
Expected
0
5
10
15
20
25
30
35
LMM meta, All eth, 12829 genes, lambda=0.99
LMM meta, Eur, 11967 genes, lambda=0.98

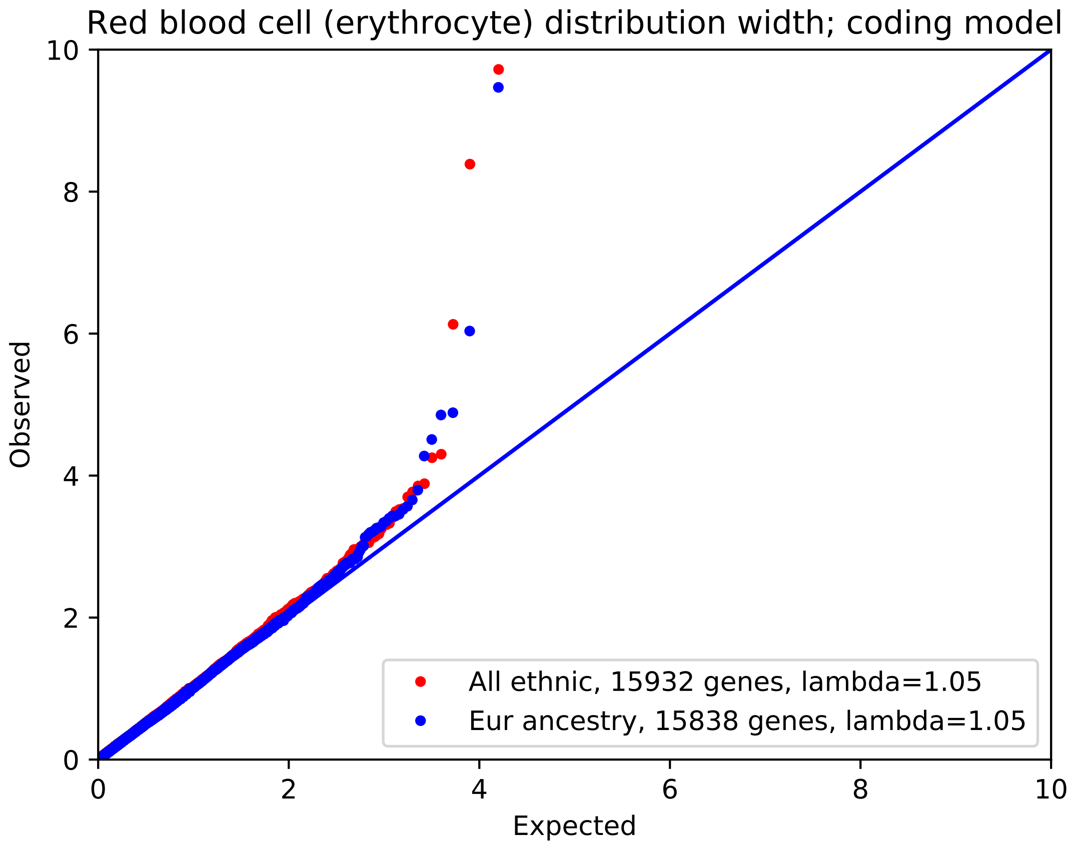

Red blood cell (erythrocyte) distribution width; coding model
Observed
Expected
0
2
4
6
8
10
All ethnic, 15932 genes, lambda=1.05
Eur ancestry, 15838 genes, lambda=1.05

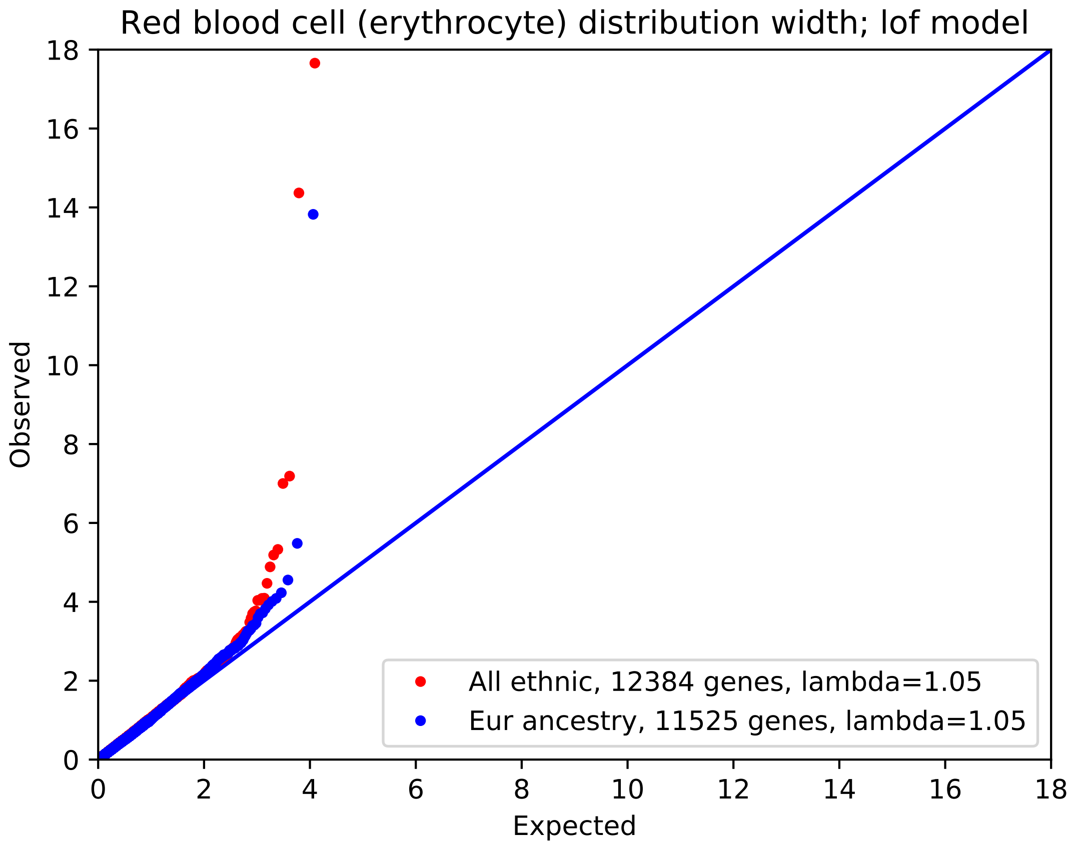

Red blood cell (erythrocyte) distribution width; lof model
Observed
Expected
0
2
4
6
8
10
12
14
16
18
All ethnic, 12384 genes, lambda=1.05
Eur ancestry, 11525 genes, lambda=1.05

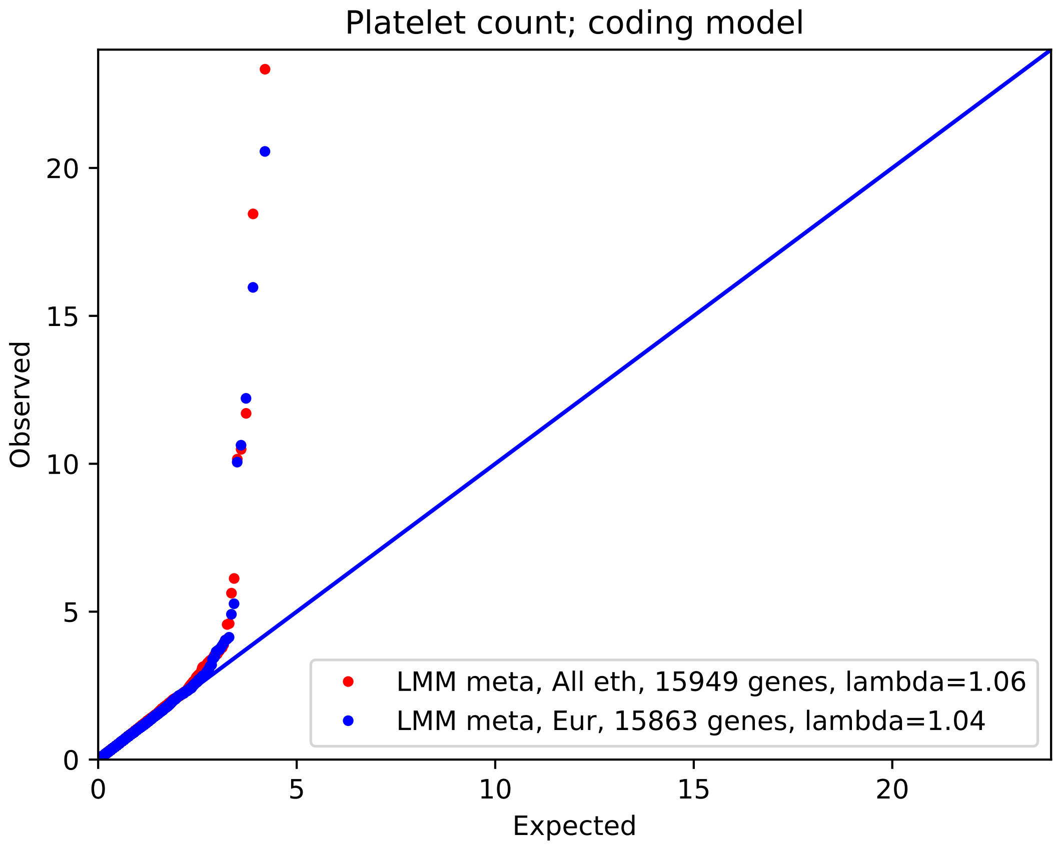

Platelet count; coding model
Observed
Expected
0
5
10
15
20
LMM meta, All eth, 15949 genes, lambda=1.06
LMM meta, Eur, 15863 genes, lambda=1.04

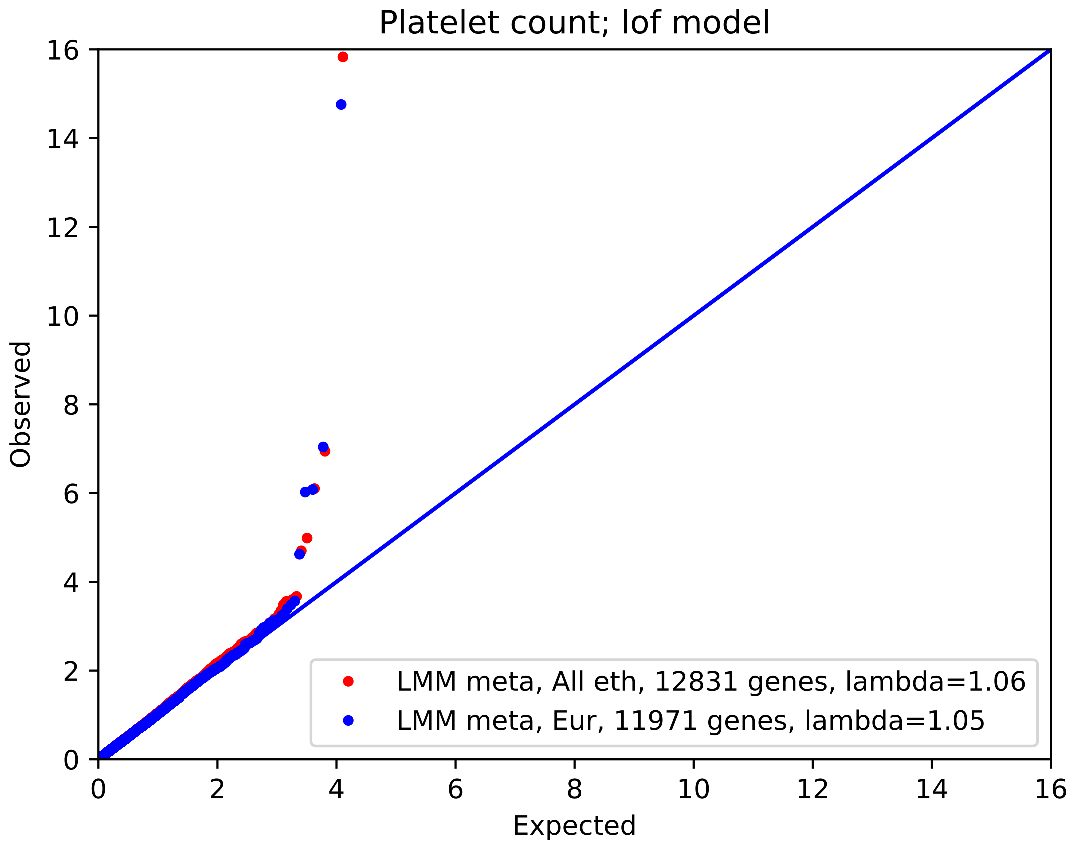
Platelet count; lof model
Observed
Expected
0
2
4
6
8
10
12
14
16
LMM meta, All eth, 12831 genes, lambda=1.06
LMM meta, Eur, 11971 genes, lambda=1.05

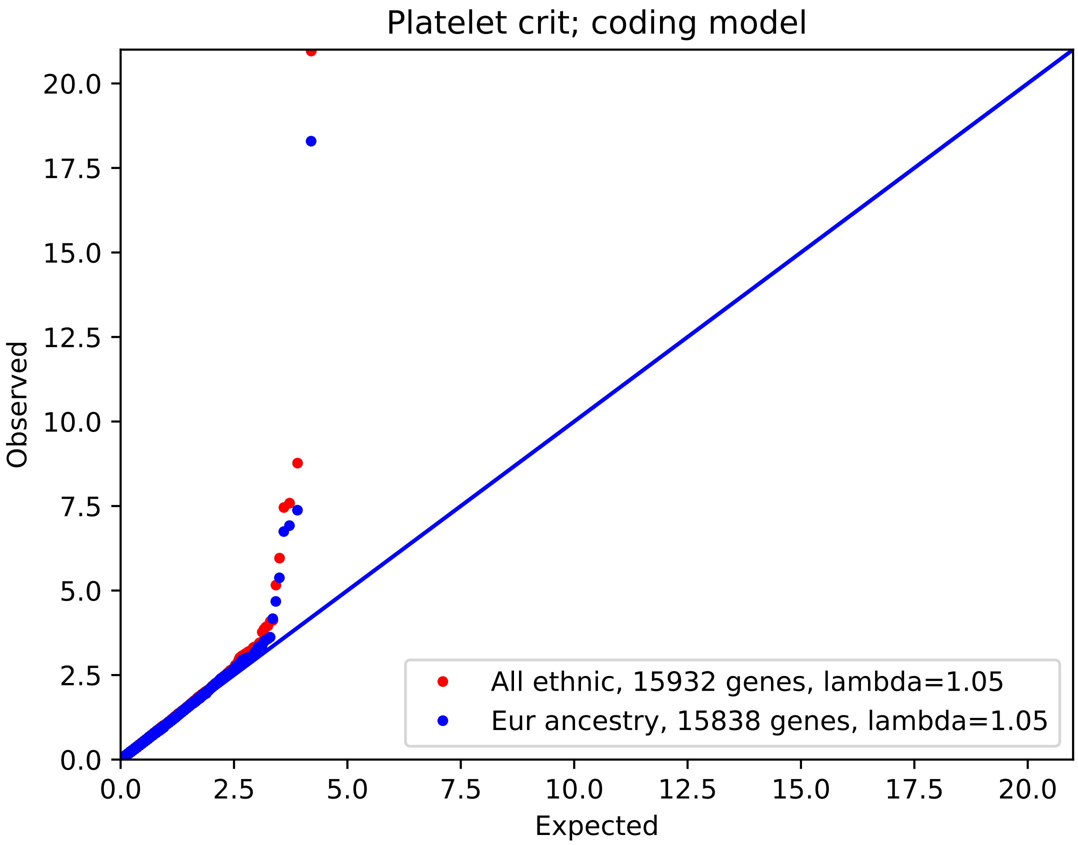
Platelet crit; coding model
Observed
Expected
0.0
2.5
5.0
7.5
10.0
12.5
15.0
17.5
20.0
All ethnic, 15932 genes, lambda=1.05
Eur ancestry, 15838 genes, lambda=1.05

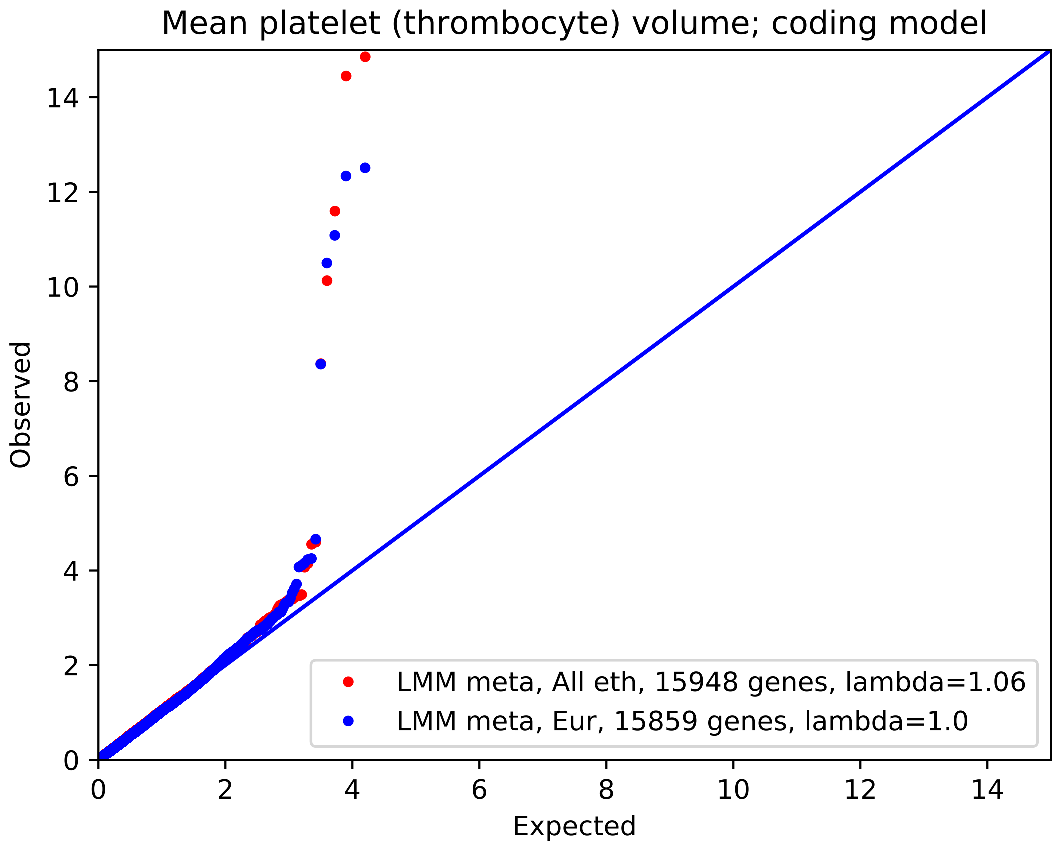

Mean platelet (thrombocyte) volume; coding model
Observed
Expected
0
2
4
6
8
10
12
14
LMM meta, All eth, 15948 genes, lambda=1.06
LMM meta, Eur, 15859 genes, lambda=1.0

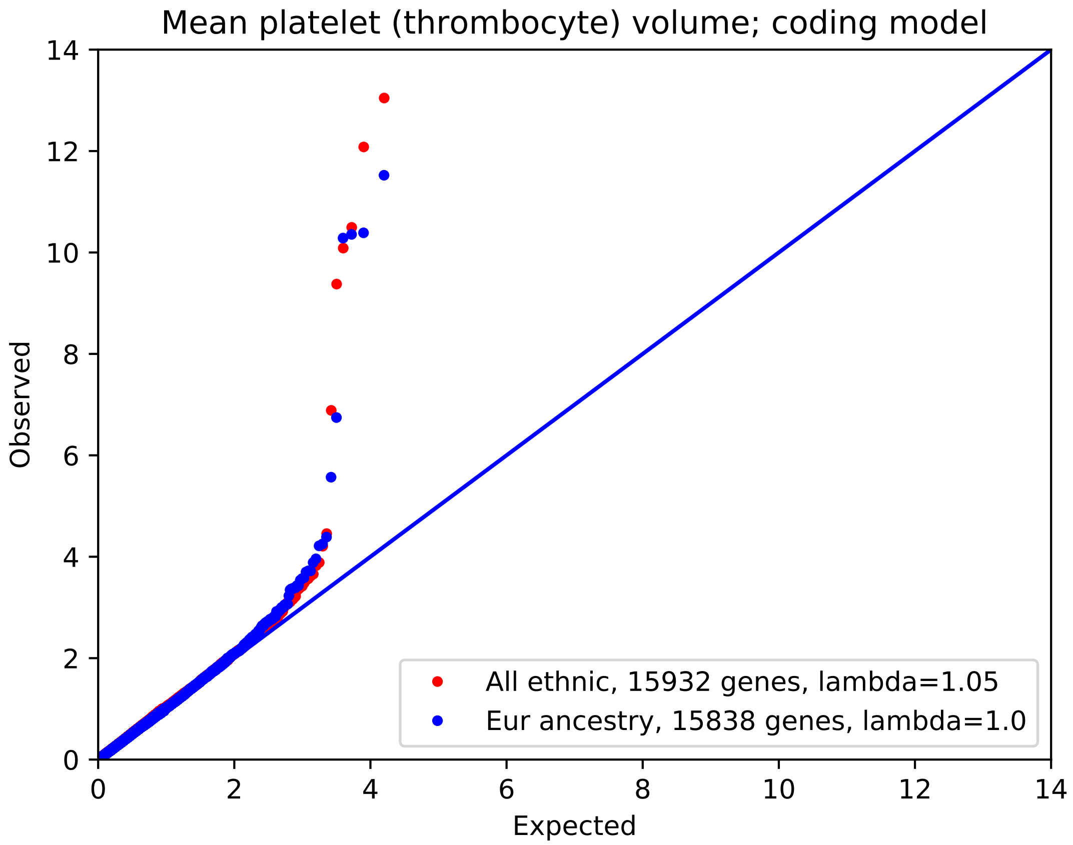

Mean platelet (thrombocyte) volume; coding model
Observed
Expected
0
2
4
6
8
10
12
14
All ethnic, 15932 genes, lambda=1.05
Eur ancestry, 15838 genes, lambda=1.0

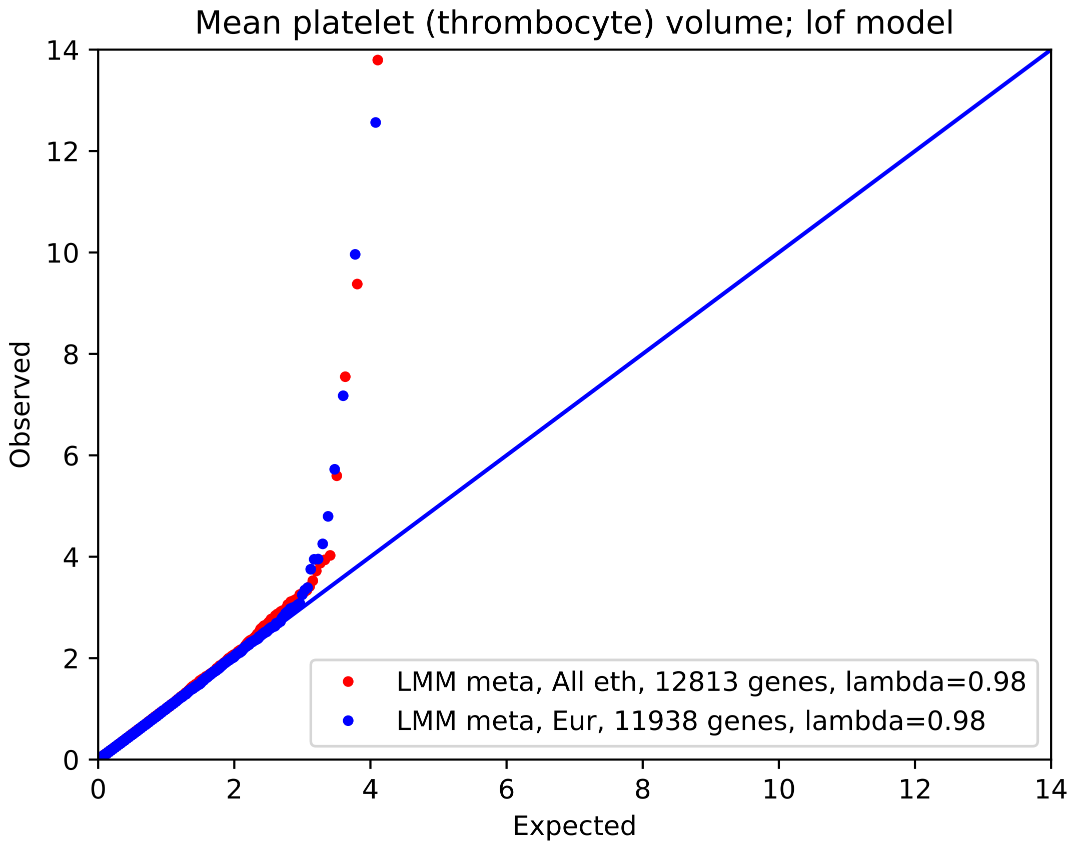

Mean platelet (thrombocyte) volume; lof model
Observed
Expected
0
2
4
6
8
10
12
14
LMM meta, All eth, 12813 genes, lambda=0.98
LMM meta, Eur, 11938 genes, lambda=0.98

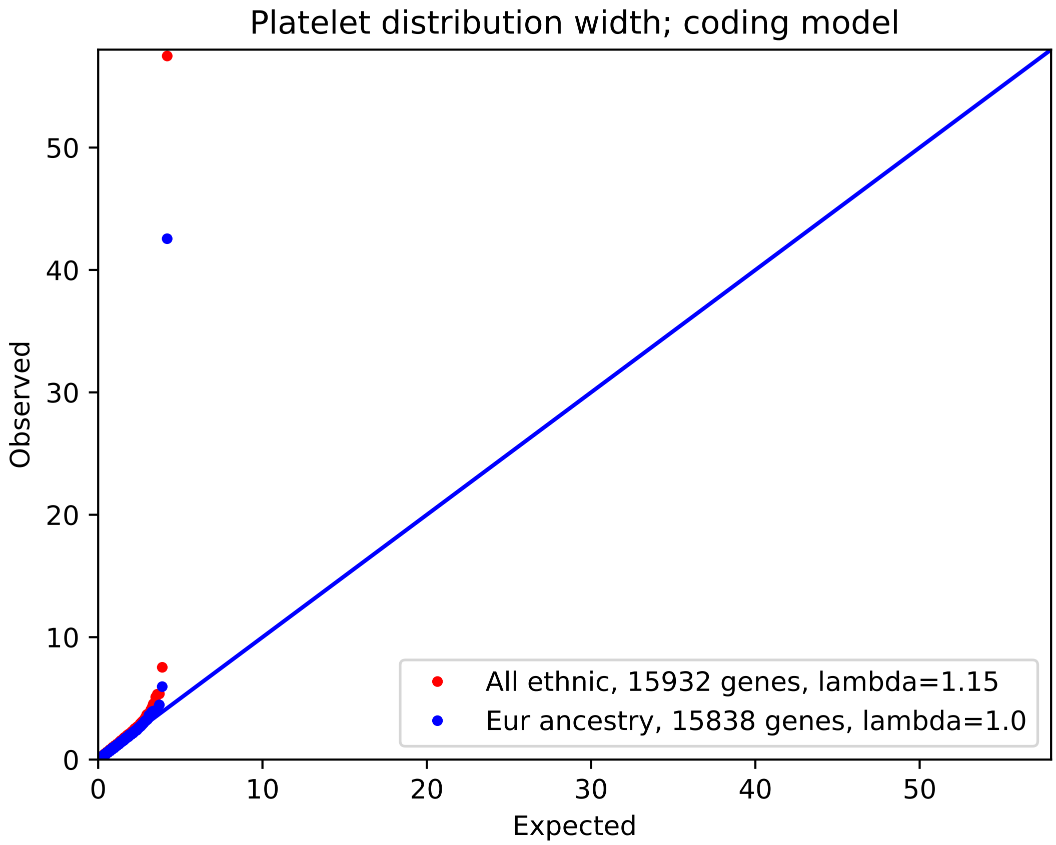

Platelet distribution width; coding model
Observed
Expected
0
10
20
30
40
50
All ethnic, 15932 genes, lambda=1.15
Eur ancestry, 15838 genes, lambda=1.0

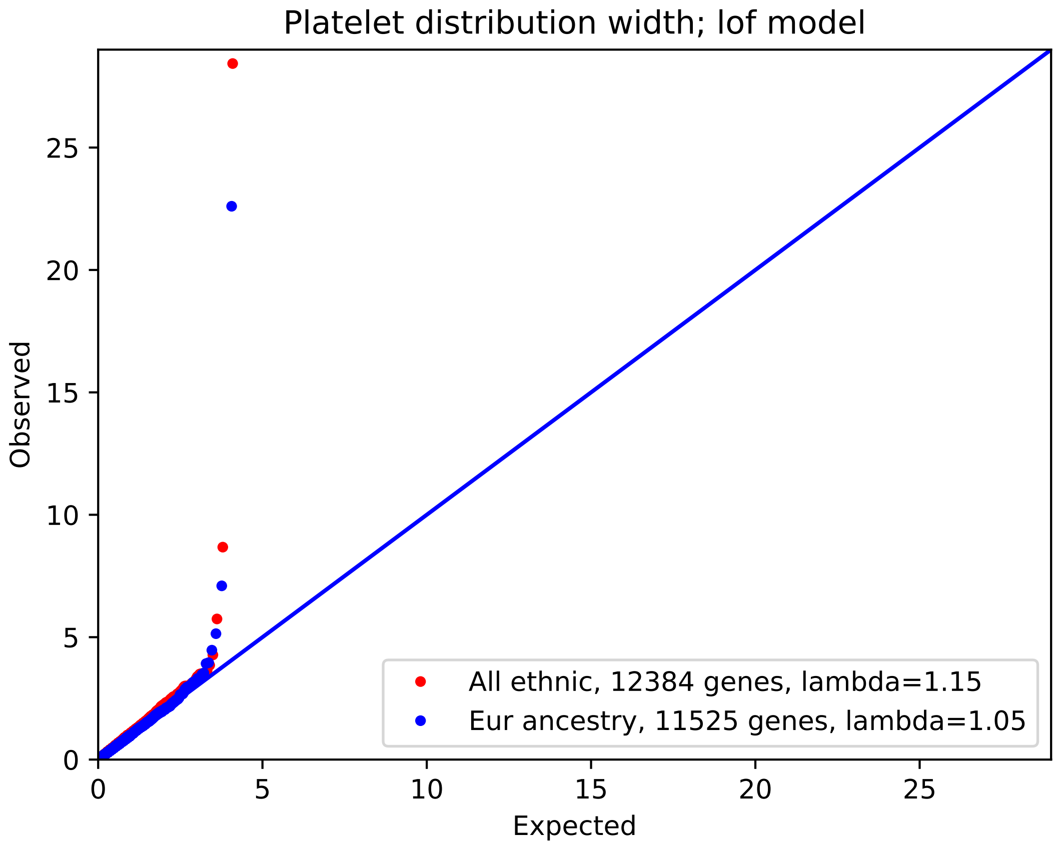
Platelet distribution width; lof model
Observed
Expected
0
5
10
15
20
25
All ethnic, 12384 genes, lambda=1.15
Eur ancestry, 11525 genes, lambda=1.05

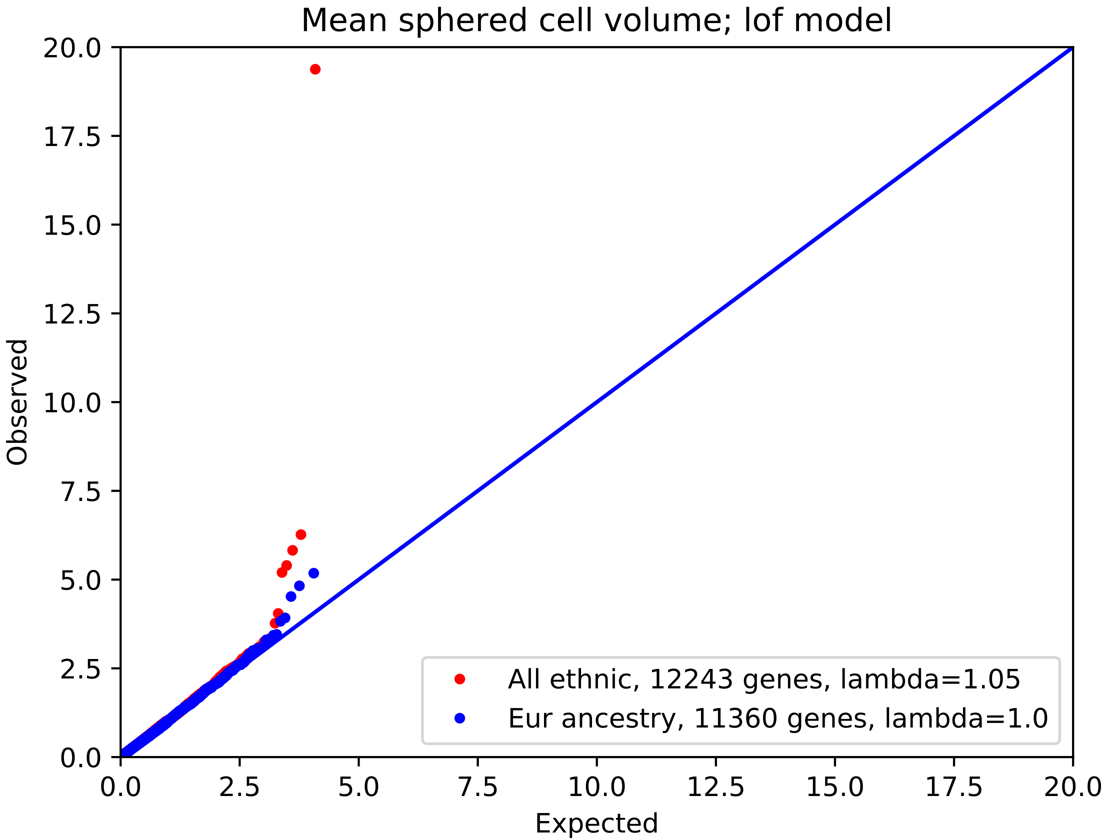

Mean sphered cell volume; lof model
Observed
Expected
20.0
17.5
15.0
12.5
10.0
7.5
5.0
2.5
0.0
0.0
2.5
5.0
7.5
10.0
12.5
15.0
17.5
20.0
All ethnic, 12243 genes, lambda=1.05
Eur ancestry, 11360 genes, lambda=1.0

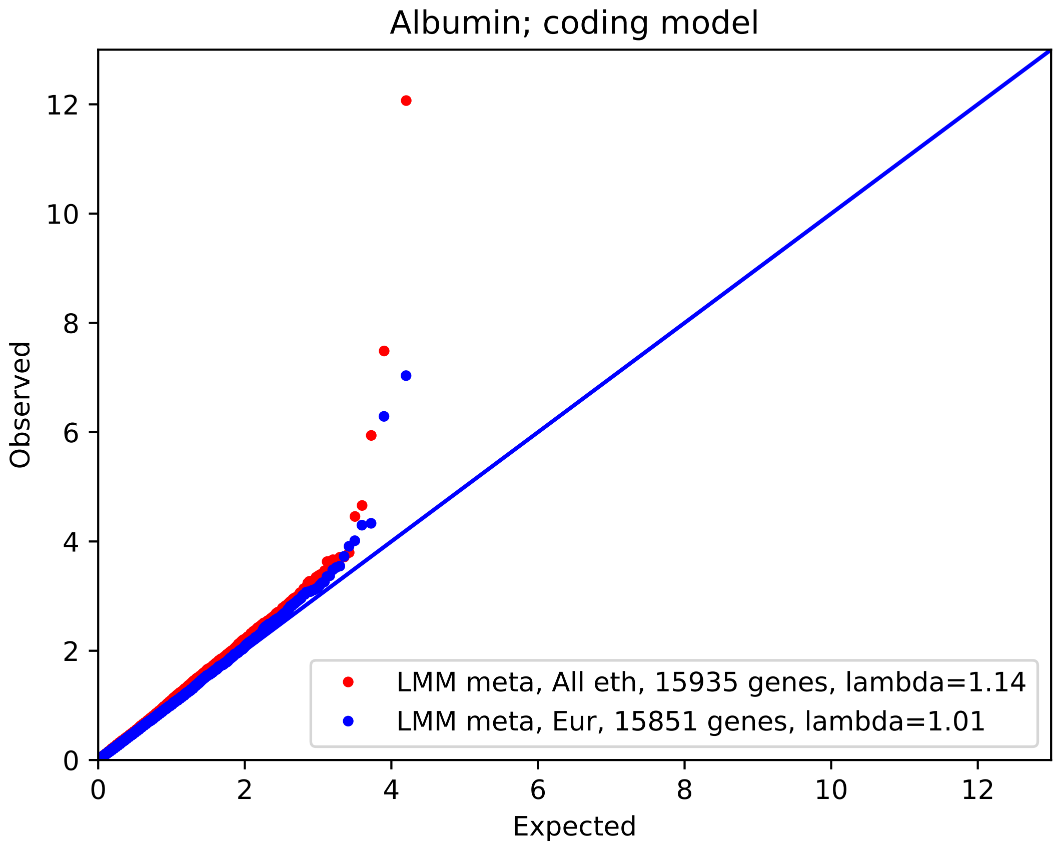
Albumin; coding model
Observed
Expected
0
2
4
6
8
10
12
LMM meta, All eth, 15935 genes, lambda=1.14
LMM meta, Eur, 15851 genes, lambda=1.01

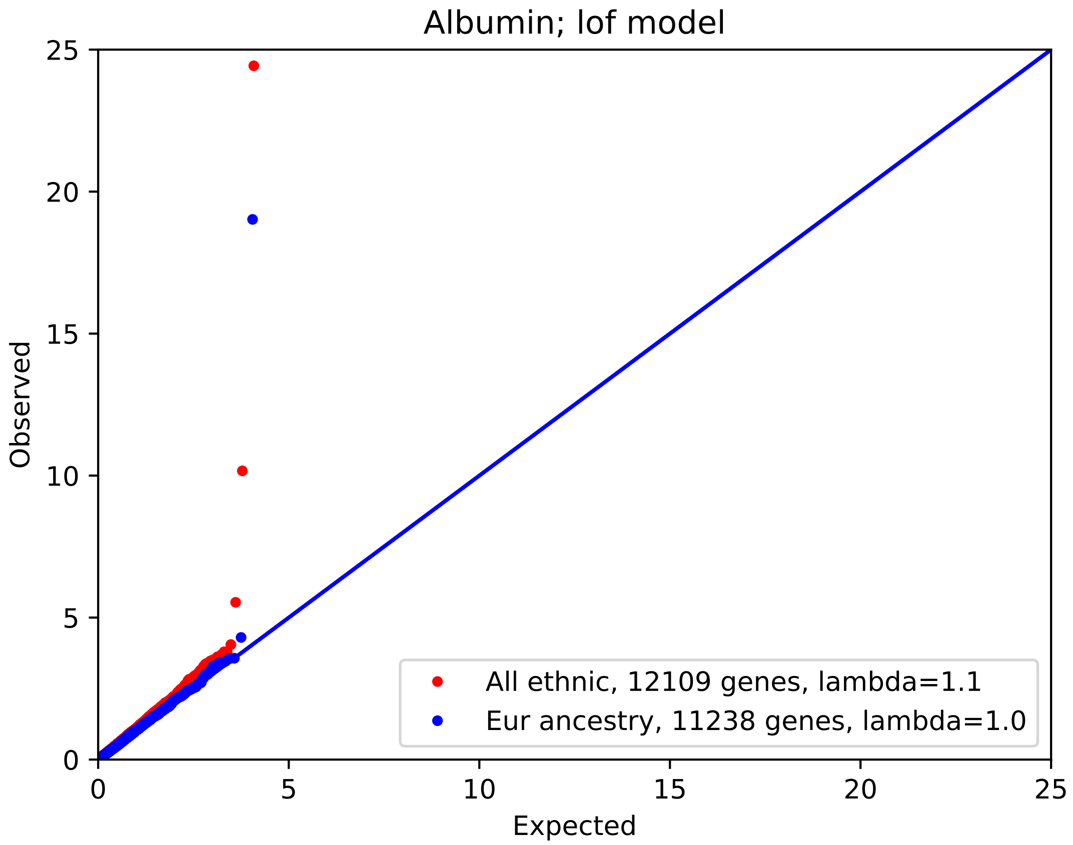
Albumin; lof model
Observed
Expected
0
5
10
15
20
25
All ethnic, 12109 genes, lambda=1.1
Eur ancestry, 11238 genes, lambda=1.0

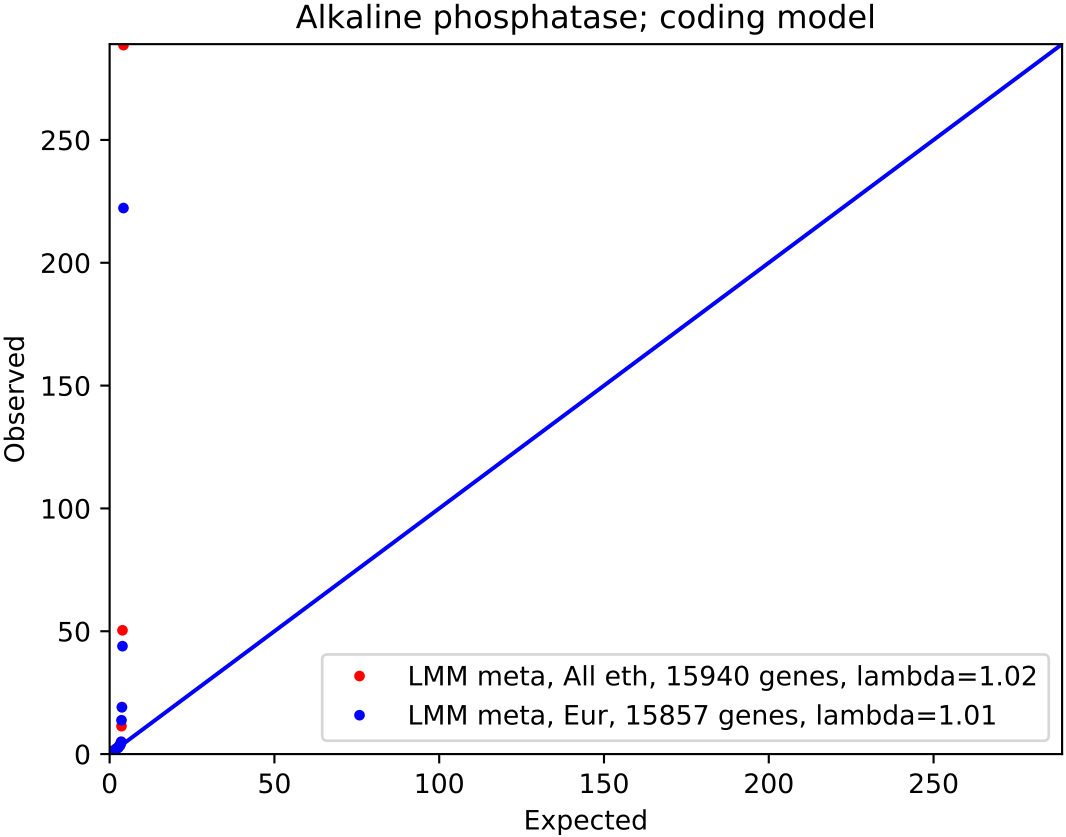

Alkaline phosphatase; coding model
Observed
Expected
0
50
100
150
200
250
LMM meta, All eth, 15940 genes, lambda=1.02
LMM meta, Eur, 15857 genes, lambda=1.01

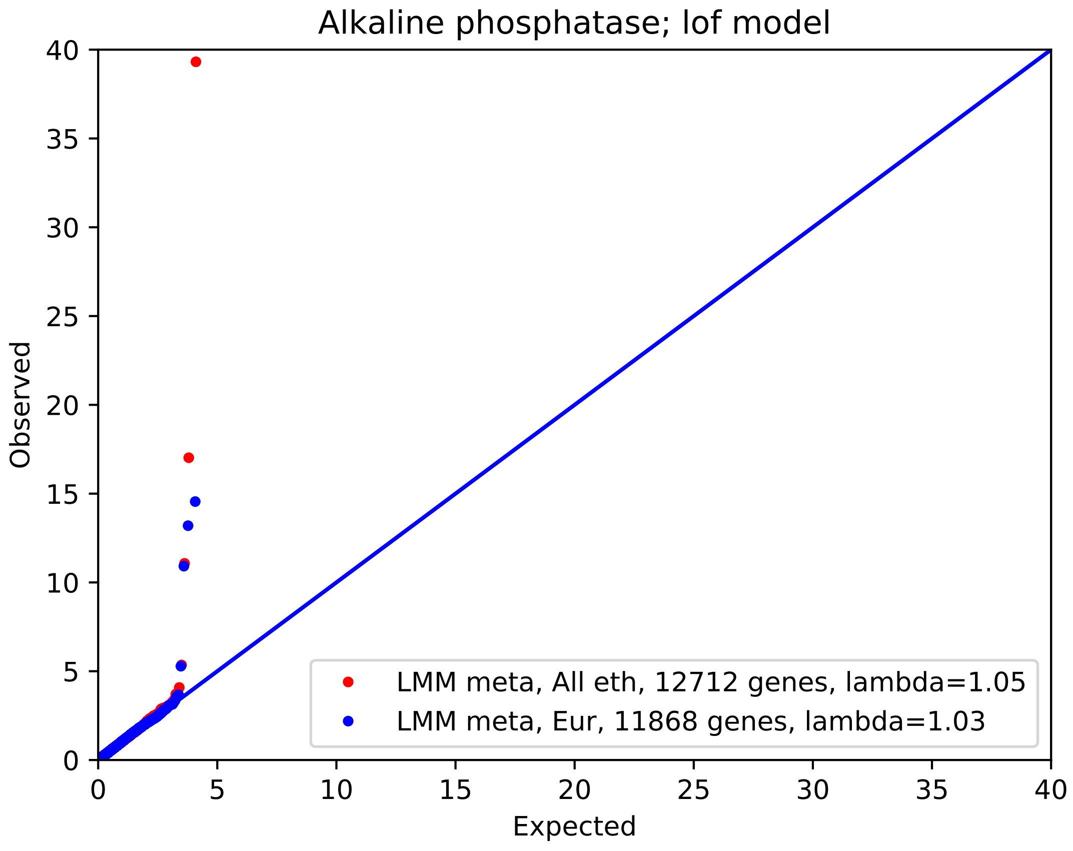

Alkaline phosphatase; lof model
Observed
Expected
0
5
10
15
20
25
30
35
40
LMM meta, All eth, 12712 genes, lambda=1.05
LMM meta, Eur, 11868 genes, lambda=1.03

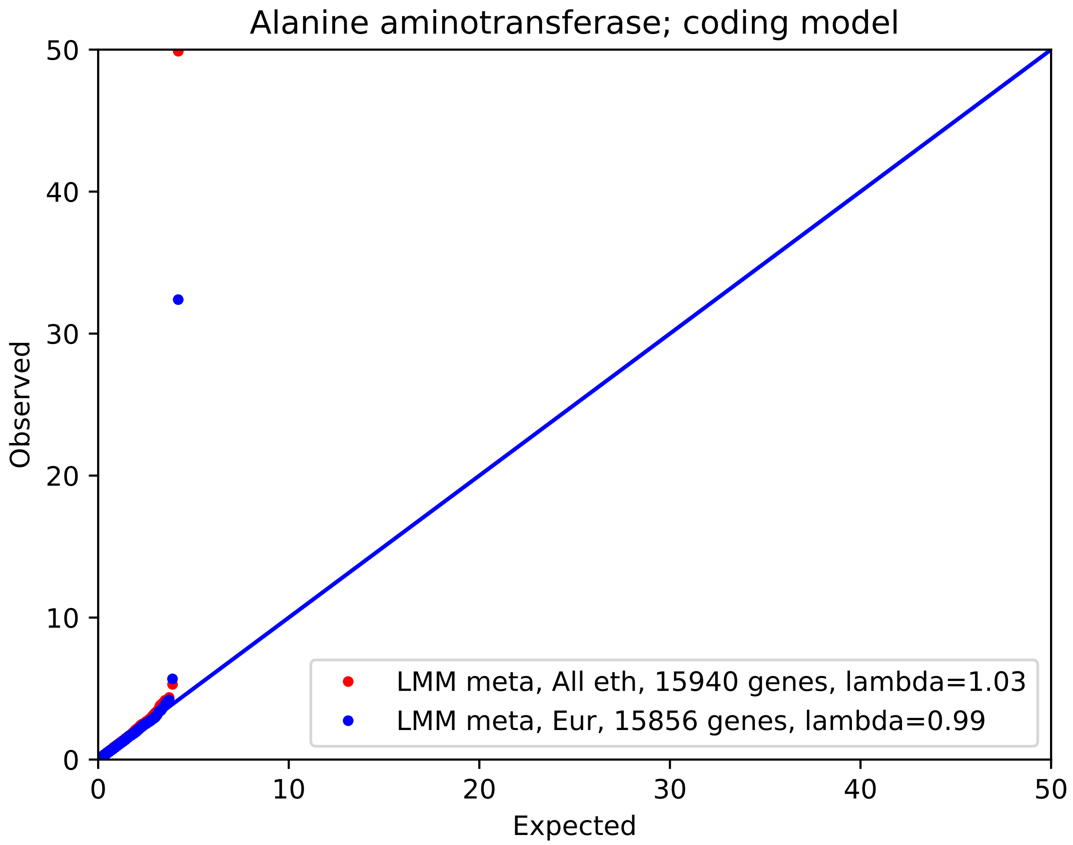

Alanine aminotransferase; coding model
Observed
Expected
0
10
20
30
40
50
LMM meta, All eth, 15940 genes, lambda=1.03
LMM meta, Eur, 15856 genes, lambda=0.99

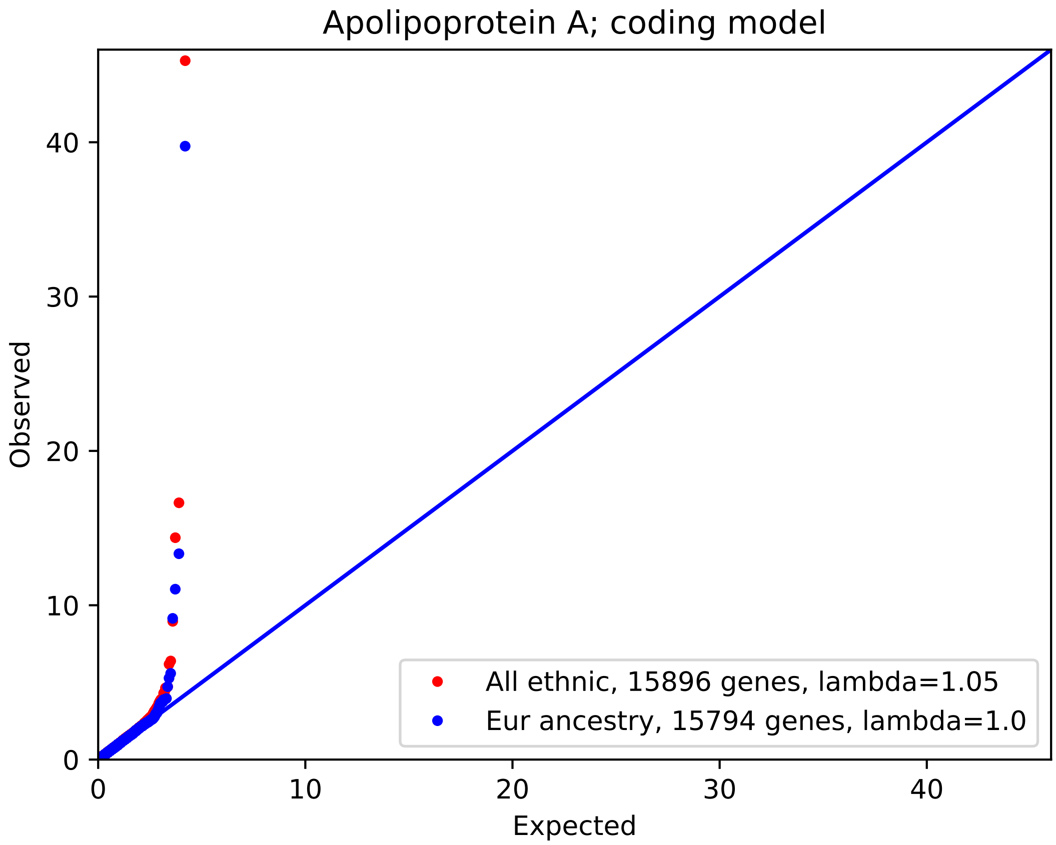

Apolipoprotein A; coding model
Observed
Expected
0
10
20
30
40
All ethnic, 15896 genes, lambda=1.05
Eur ancestry, 15794 genes, lambda=1.0

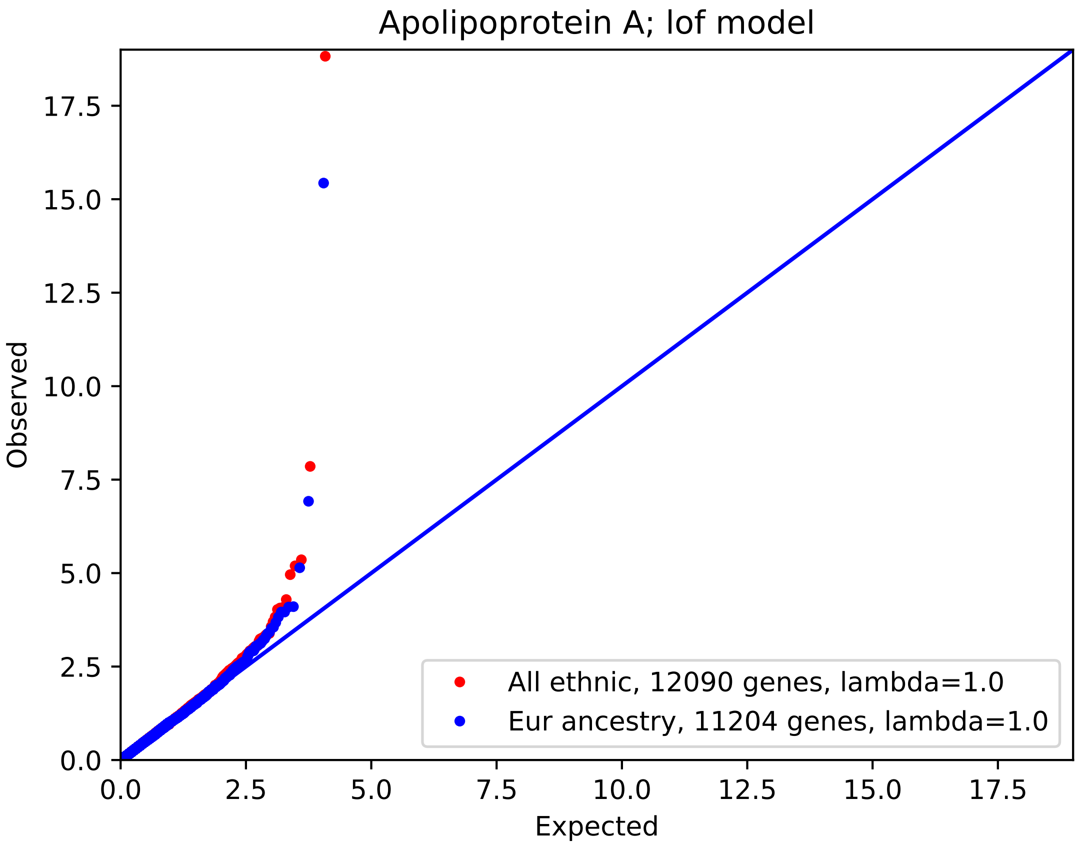
Apolipoprotein A; lof model
Observed
Expected
0.0
2.5
5.0
7.5
10.0
12.5
15.0
17.5
All ethnic, 12090 genes, lambda=1.0
Eur ancestry, 11204 genes, lambda=1.0

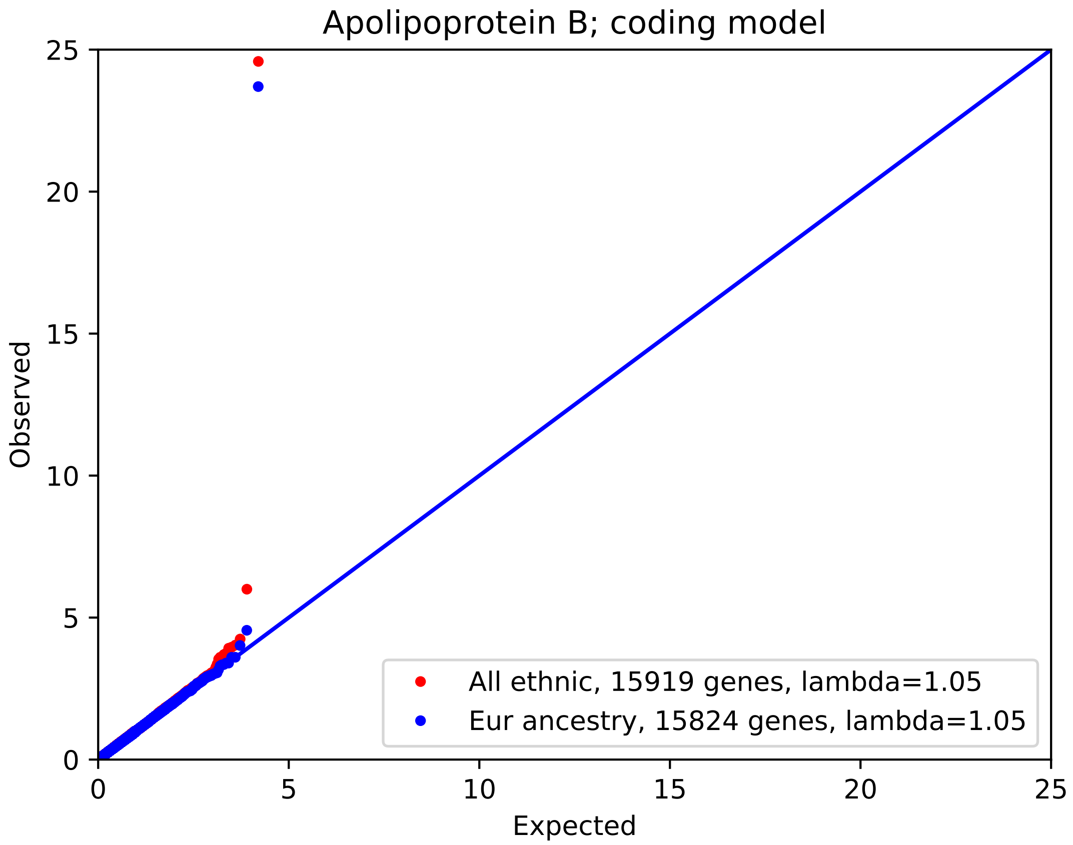

Apolipoprotein B; coding model
25
20
15
10
5
0
0
5
10
15
20
25
Observed
Expected
All ethnic, 15919 genes, lambda=1.05
Eur ancestry, 15824 genes, lambda=1.05

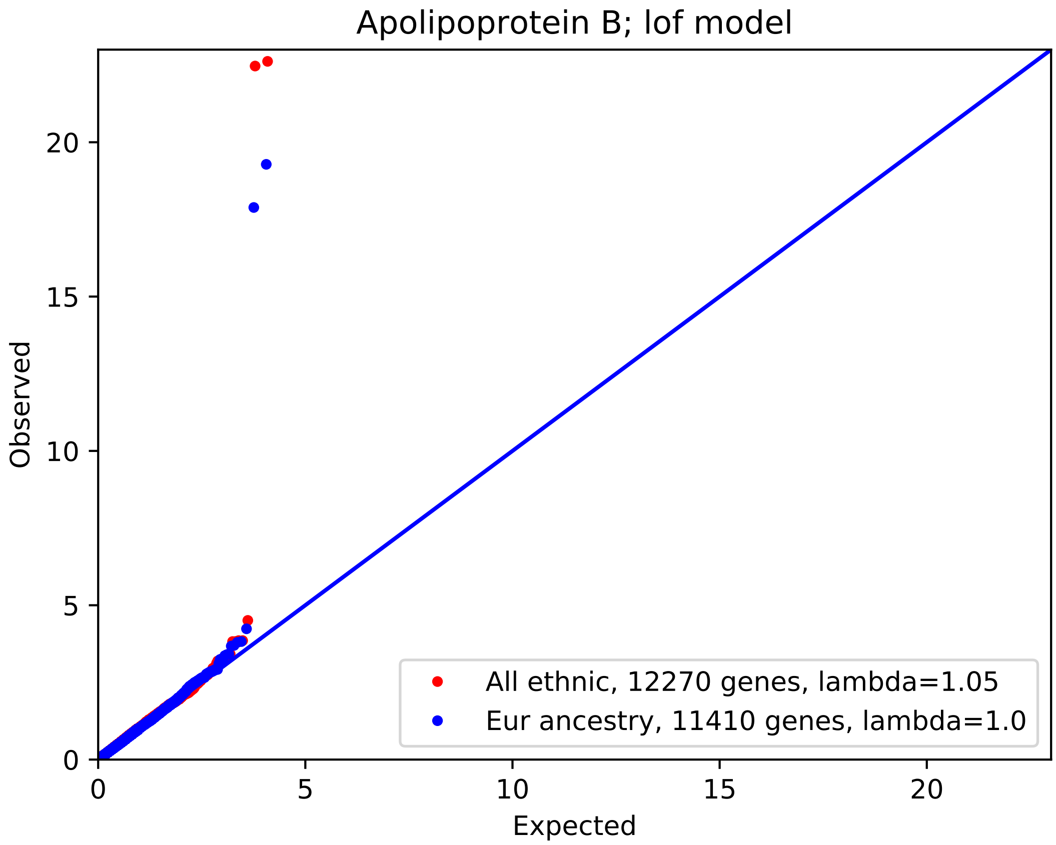
Apolipoprotein B; lof model
Observed
Expected
0
5
10
15
20
All ethnic, 12270 genes, lambda=1.05
Eur ancestry, 11410 genes, lambda=1.0

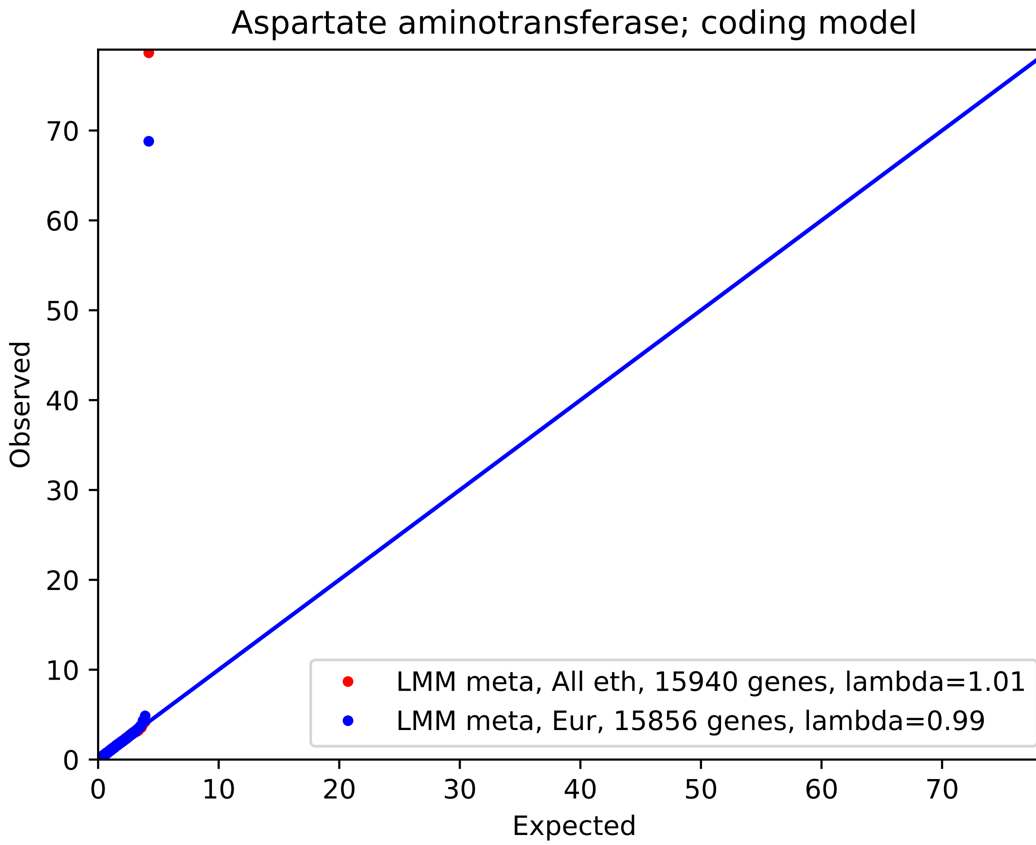

Aspartate aminotransferase; coding model
Observed
Expected
0
10
20
30
40
50
60
70
LMM meta, All eth, 15940 genes, lambda=1.01
LMM meta, Eur, 15856 genes, lambda=0.99

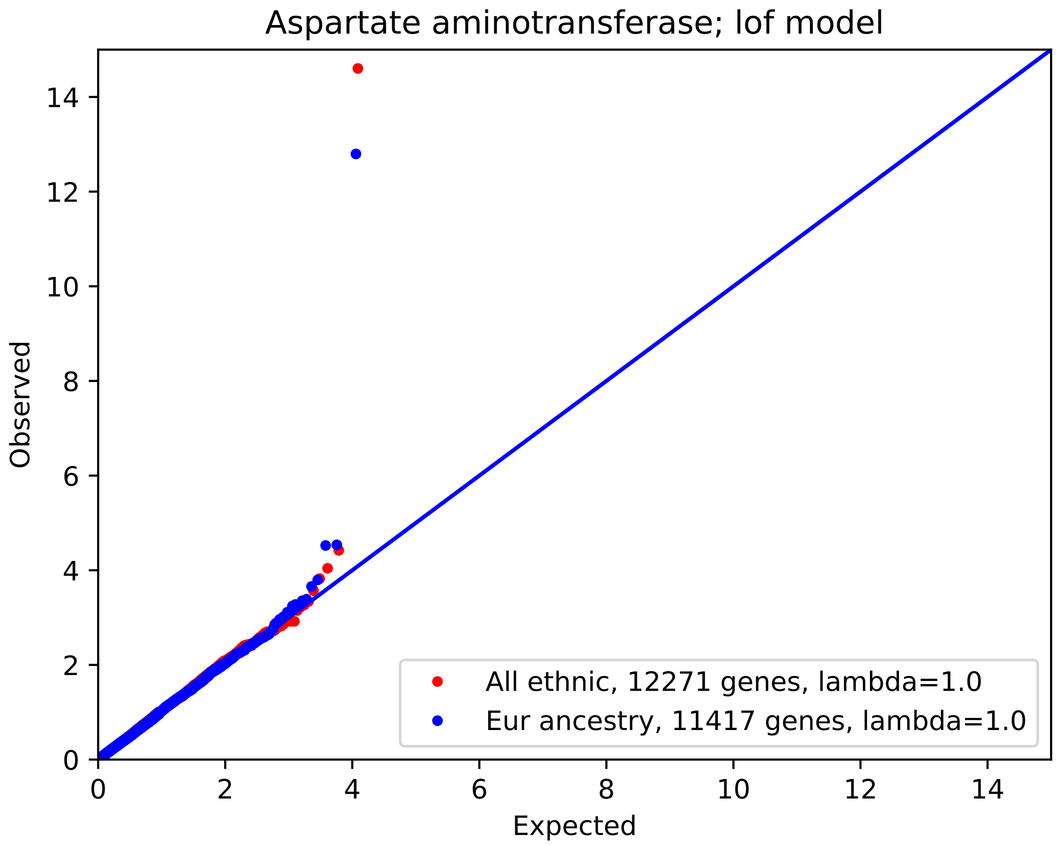
Aspartate aminotransferase; lof model
Observed
Expected
0
2
4
6
8
10
12
14
All ethnic, 12271 genes, lambda=1.0
Eur ancestry, 11417 genes, lambda=1.0

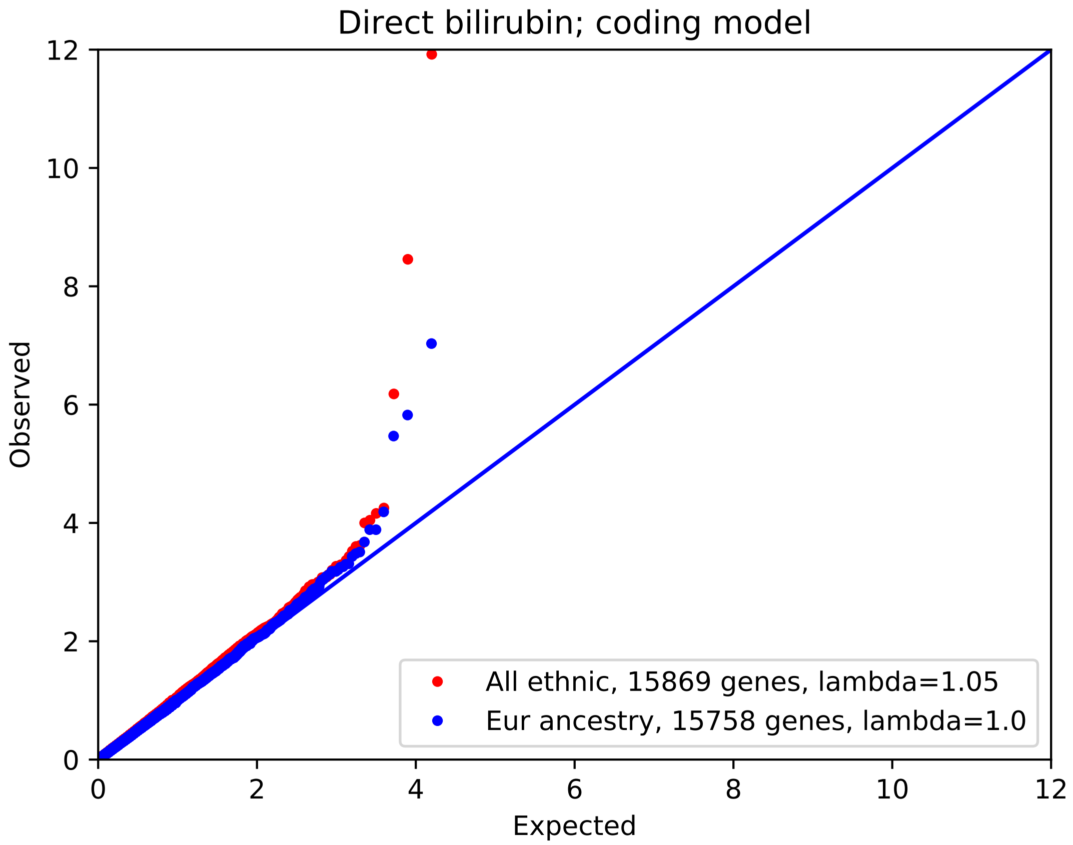

Direct bilirubin; coding model
Observed
Expected
0
2
4
6
8
10
12
All ethnic, 15869 genes, lambda=1.05
Eur ancestry, 15758 genes, lambda=1.0

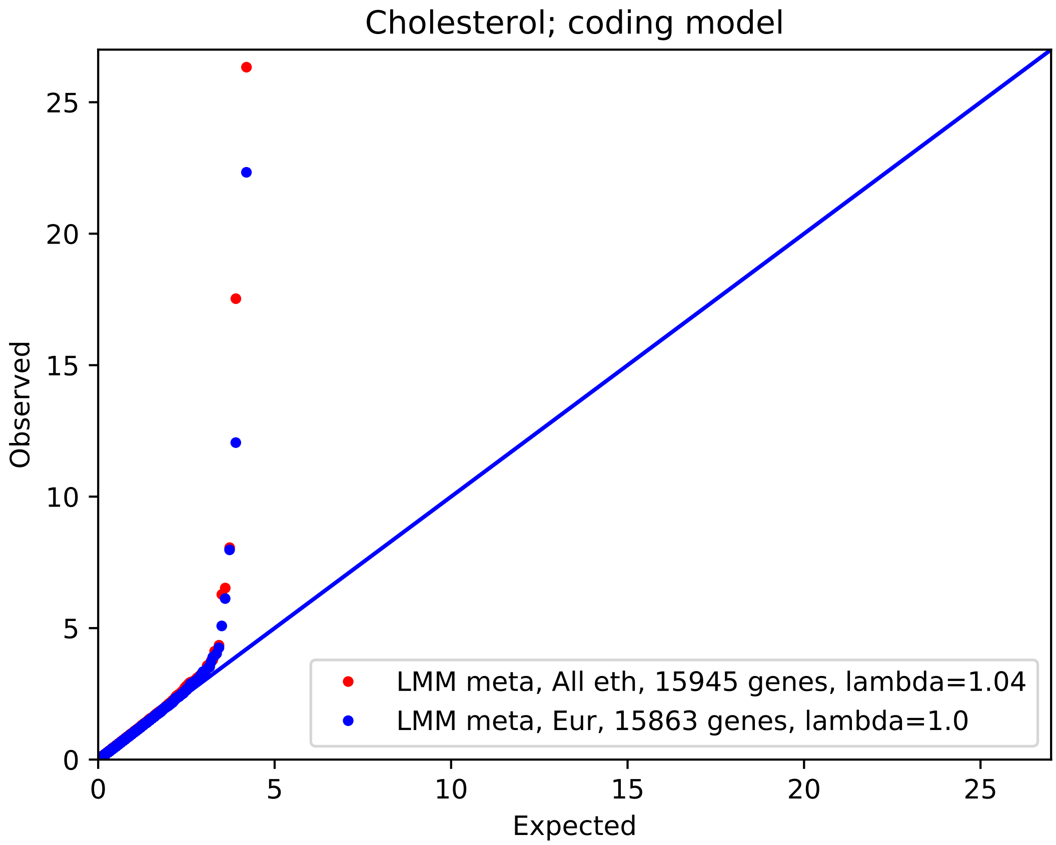

Cholesterol; coding model
Observed
Expected
0
5
10
15
20
25
LMM meta, All eth, 15945 genes, lambda=1.04
LMM meta, Eur, 15863 genes, lambda=1.0

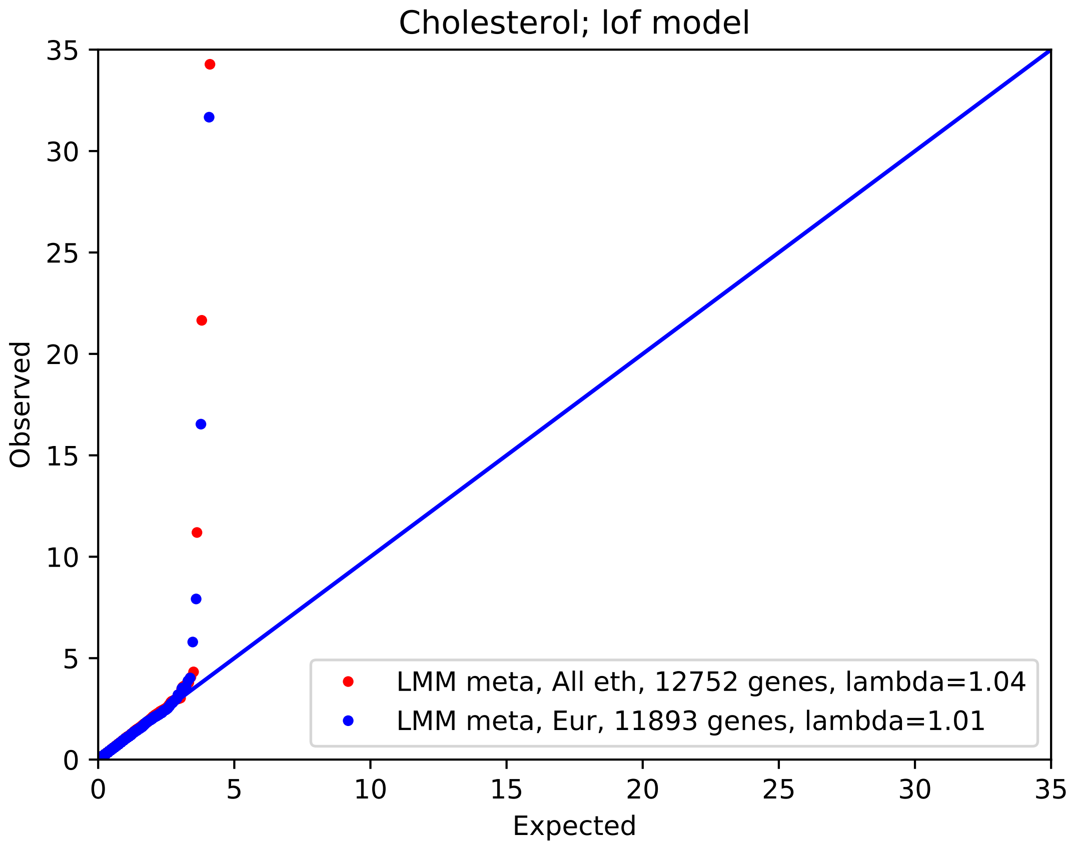
Cholesterol; lof model
Observed
Expected
0
5
10
15
20
25
30
35
LMM meta, All eth, 12752 genes, lambda=1.04
LMM meta, Eur, 11893 genes, lambda=1.01

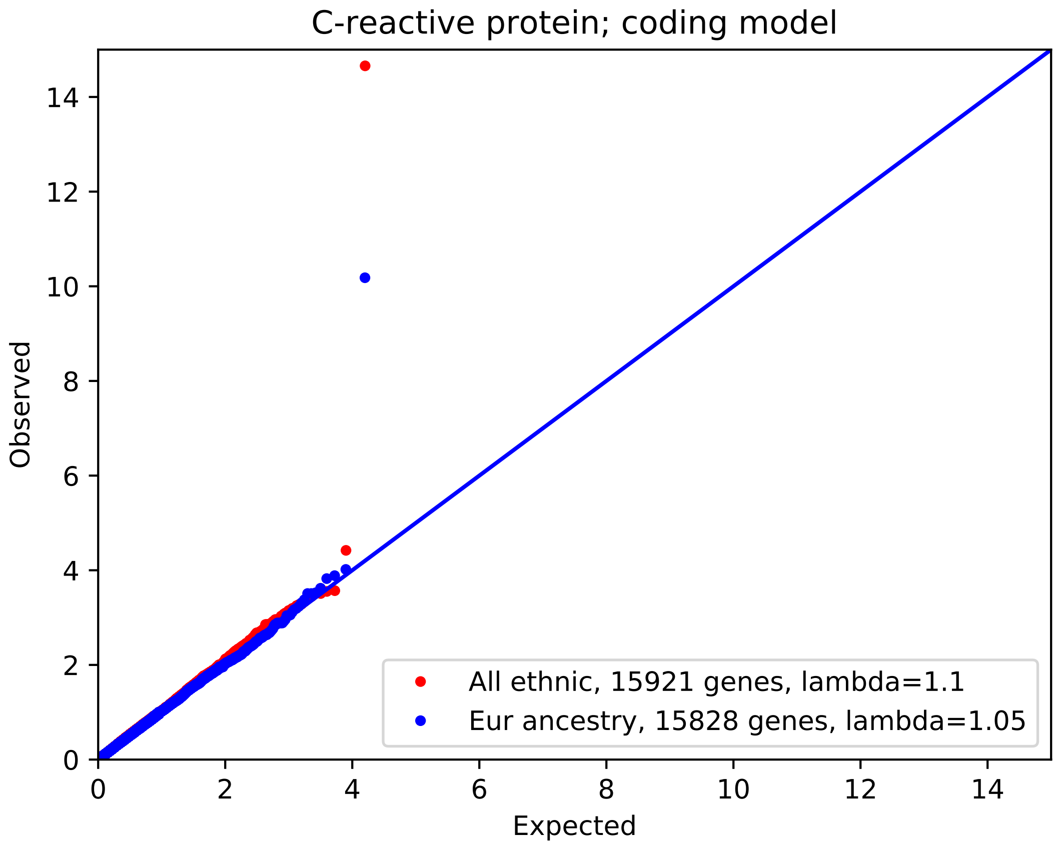

C-reactive protein; coding model
Observed
Expected
0
2
4
6
8
10
12
14
All ethnic, 15921 genes, lambda=1.1
Eur ancestry, 15828 genes, lambda=1.05

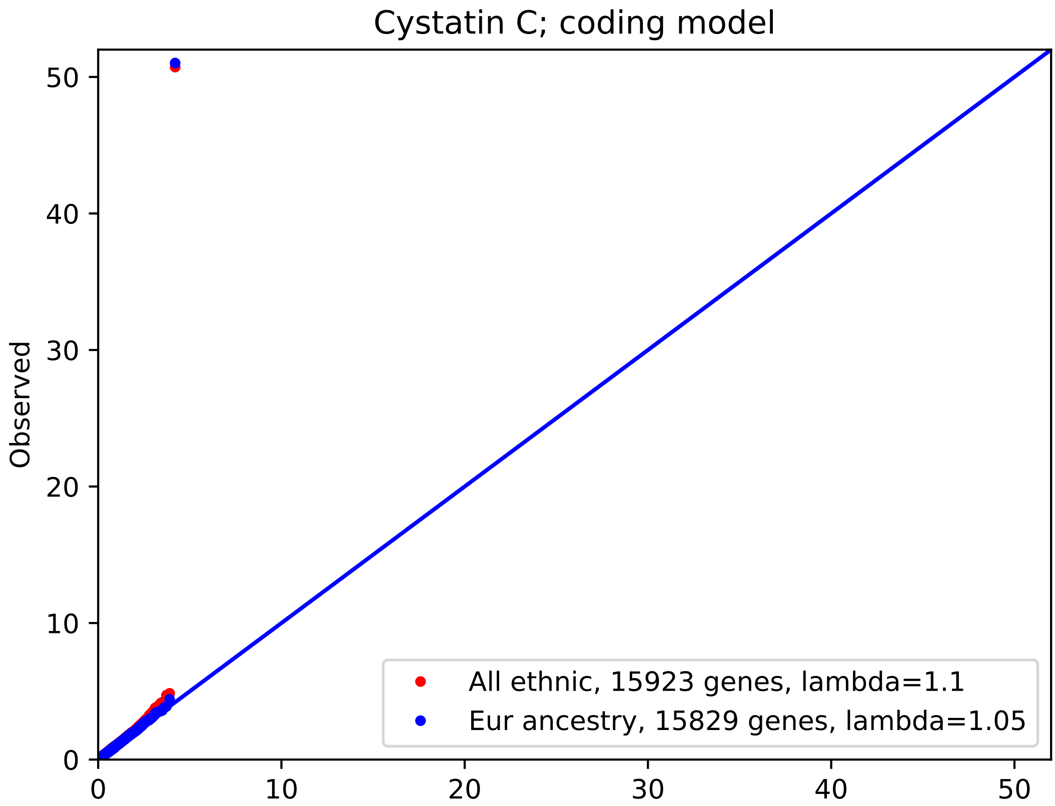

Cystatin C; coding model
Observed
Expected
0
10
20
30
40
50
All ethnic, 15923 genes, lambda=1.1
Eur ancestry, 15829 genes, lambda=1.05

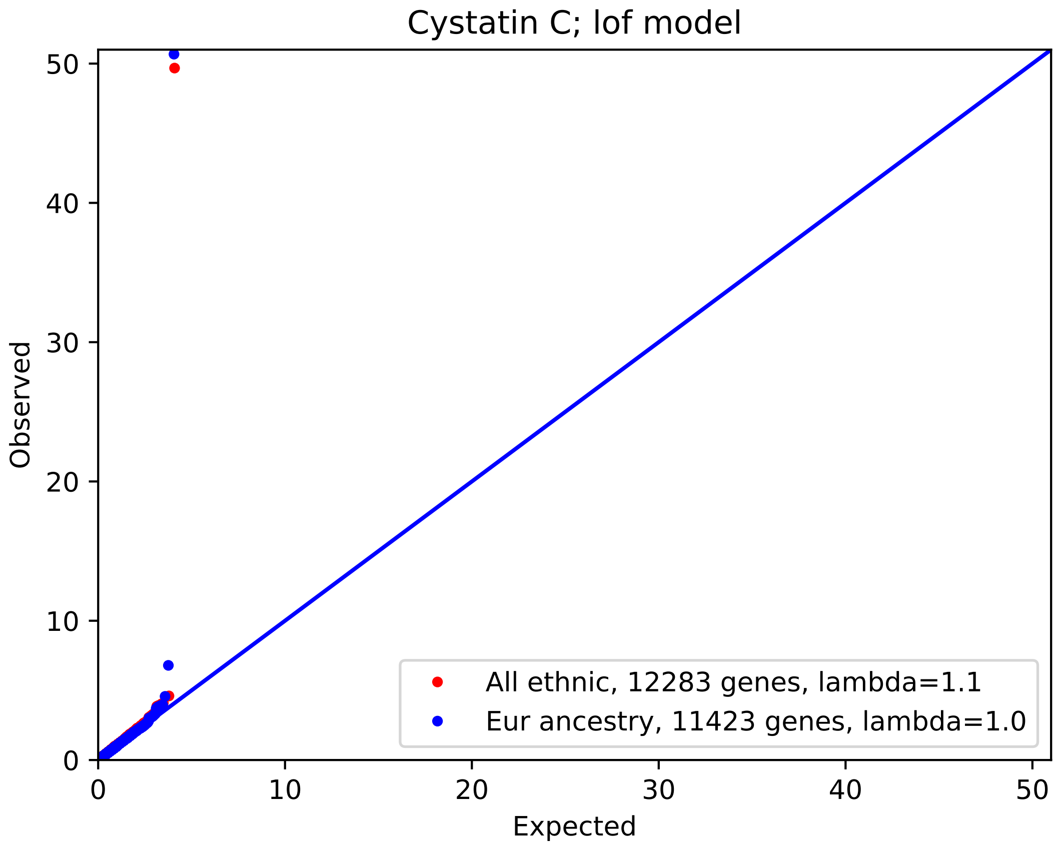
Cystatin C; lof model
Observed
Expected
0
10
20
30
40
50
All ethnic, 12283 genes, lambda=1.1
Eur ancestry, 11423 genes, lambda=1.0

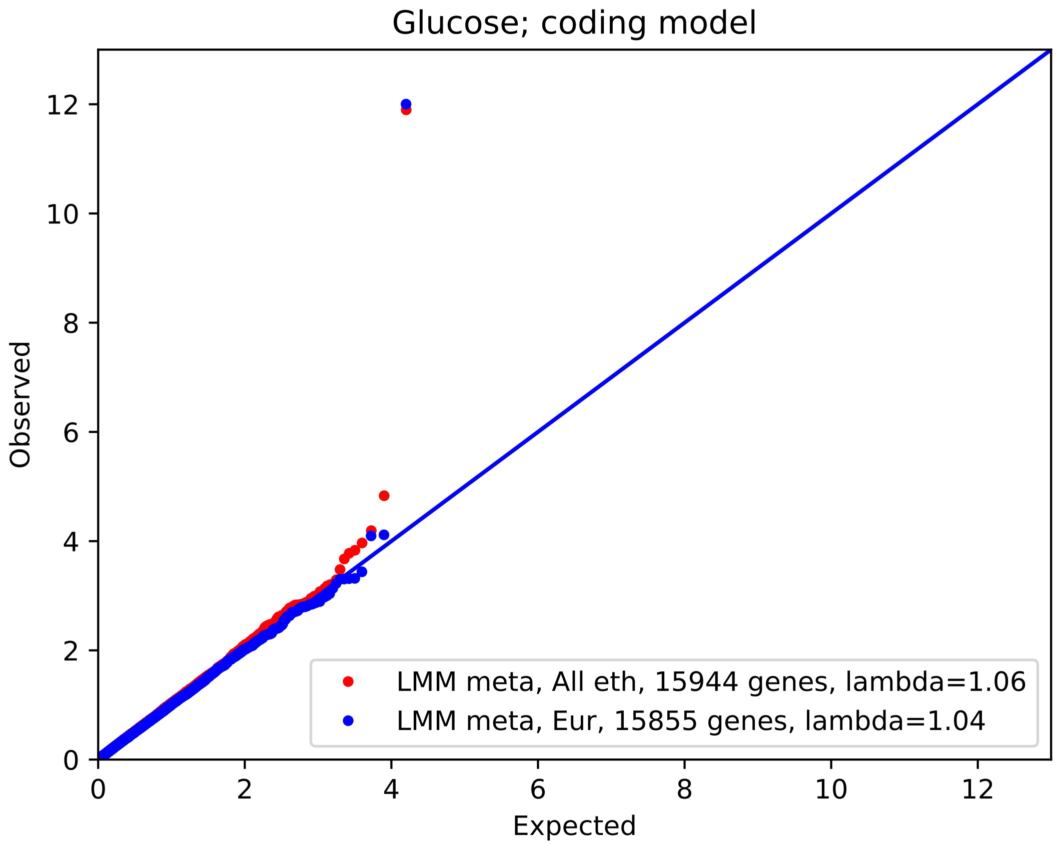
Glucose; coding model
Observed
Expected
0
2
4
6
8
10
12
0
2
4
6
8
10
12
LMM meta, All eth, 15944 genes, lambda=1.06
LMM meta, Eur, 15855 genes, lambda=1.04

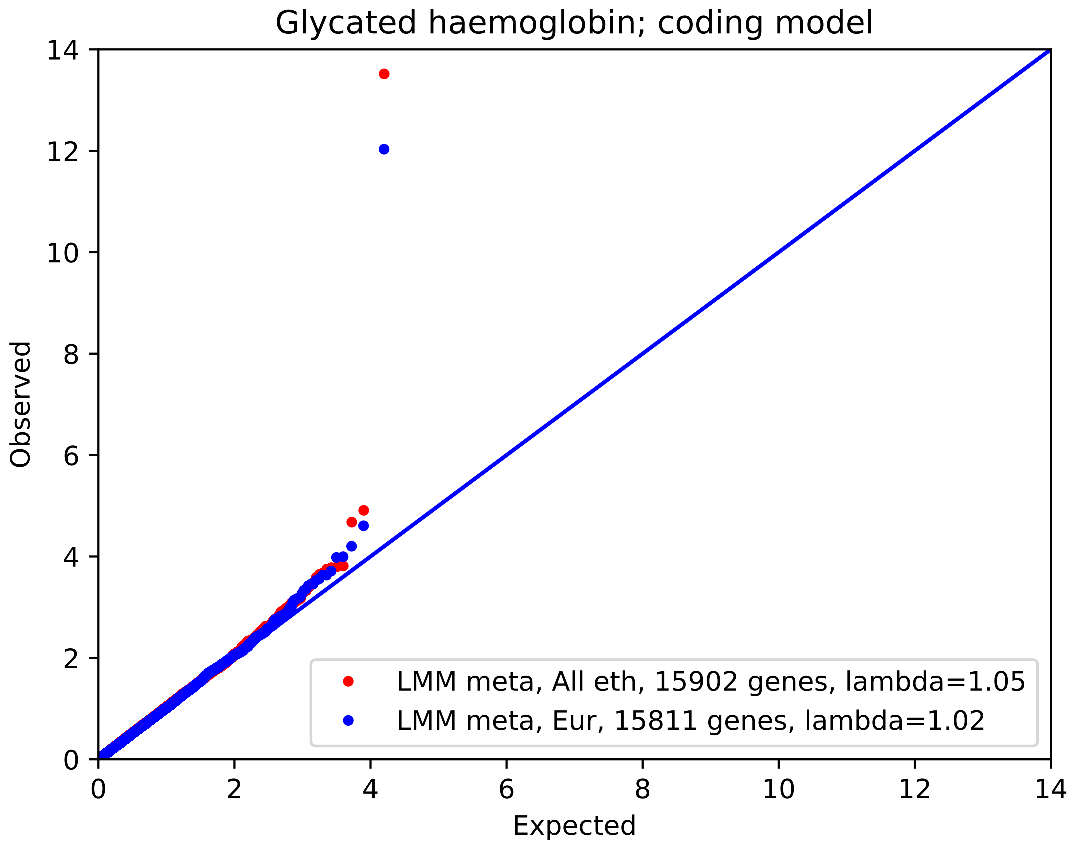
Glycated haemoglobin; coding model
Observed
Expected
0
2
4
6
8
10
12
14
LMM meta, All eth, 15902 genes, lambda=1.05
LMM meta, Eur, 15811 genes, lambda=1.02

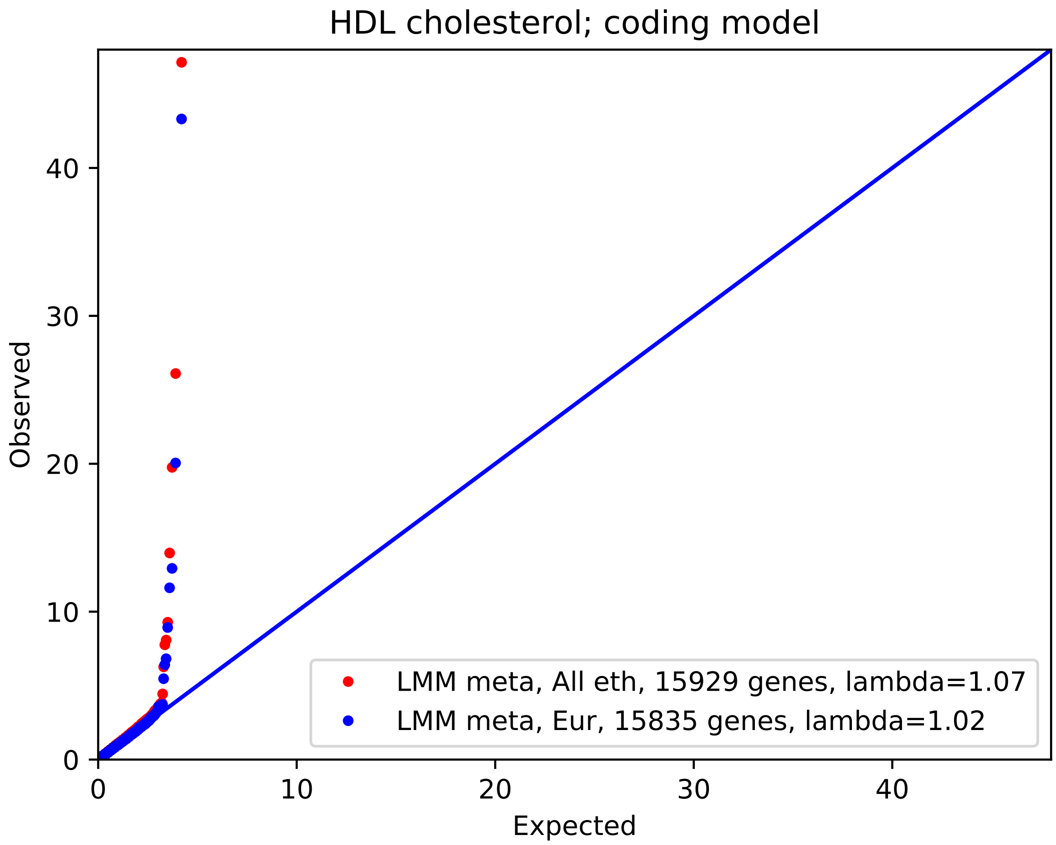

HDL cholesterol; coding model
Observed
Expected
0
10
20
30
40
0
10
20
30
40
LMM meta, All eth, 15929 genes, lambda=1.07
LMM meta, Eur, 15835 genes, lambda=1.02

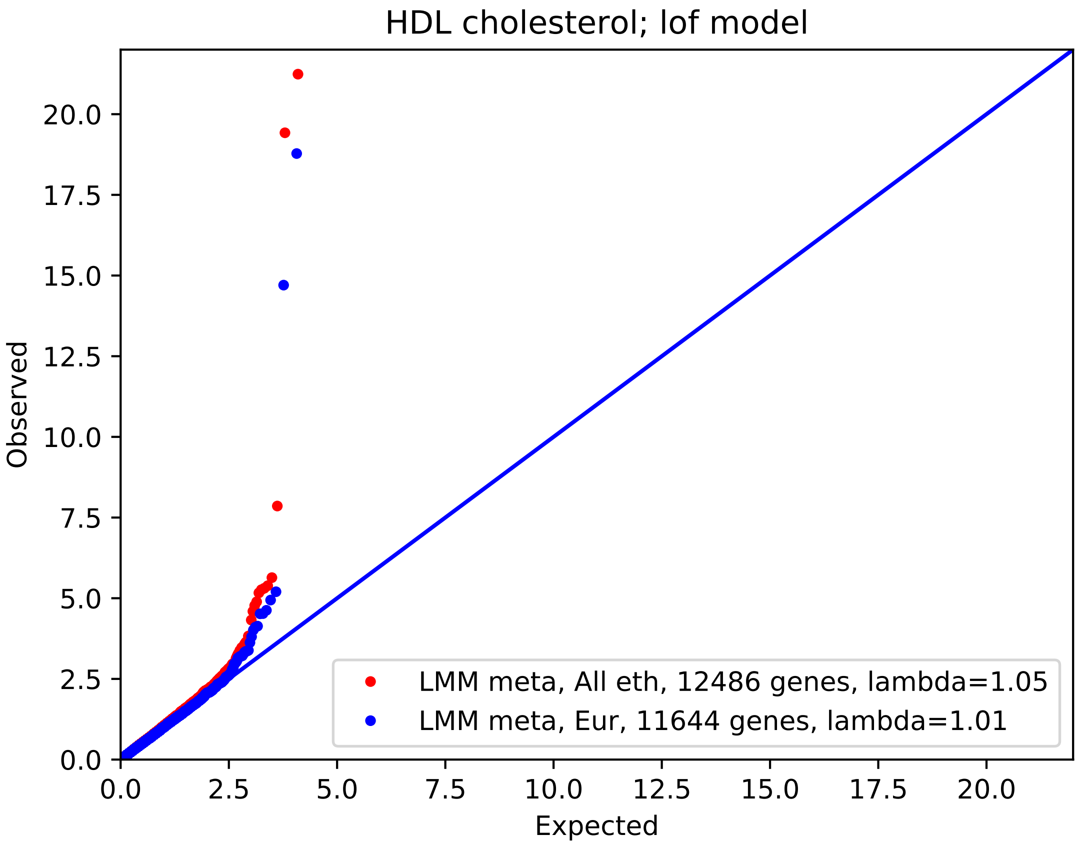
HDL cholesterol; lof model
Observed
Expected
0.0
2.5
5.0
7.5
10.0
12.5
15.0
17.5
20.0
LMM meta, All eth, 12486 genes, lambda=1.05
LMM meta, Eur, 11644 genes, lambda=1.01

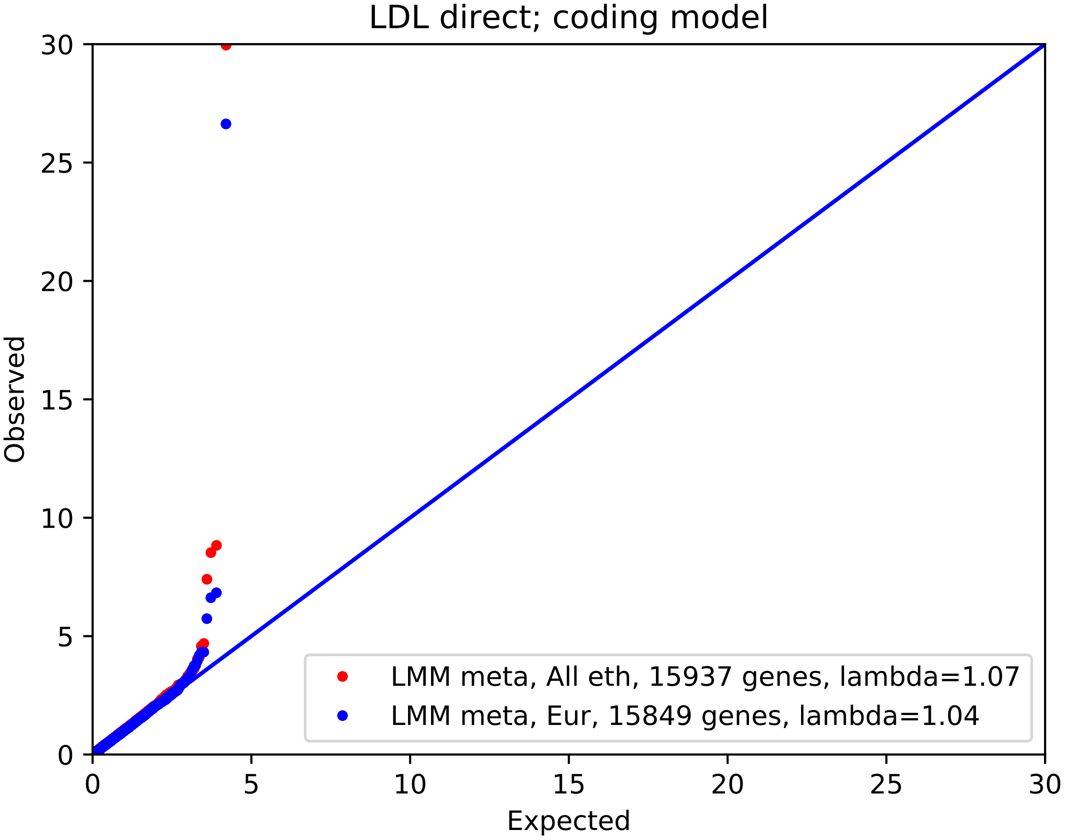

LDL direct; coding model
Observed
Expected
0
5
10
15
20
25
30
LMM meta, All eth, 15937 genes, lambda=1.07
LMM meta, Eur, 15849 genes, lambda=1.04

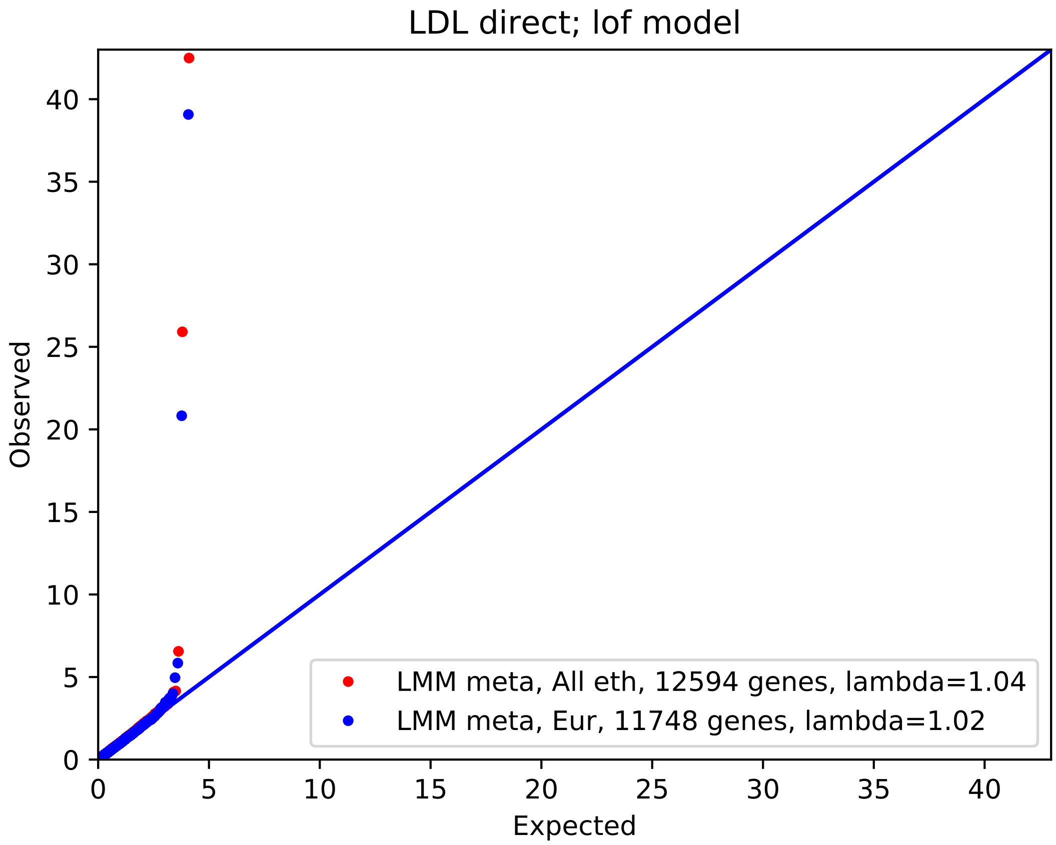
LDL direct; lof model
Observed
Expected
0
5
10
15
20
25
30
35
40
LMM meta, All eth, 12594 genes, lambda=1.04
LMM meta, Eur, 11748 genes, lambda=1.02

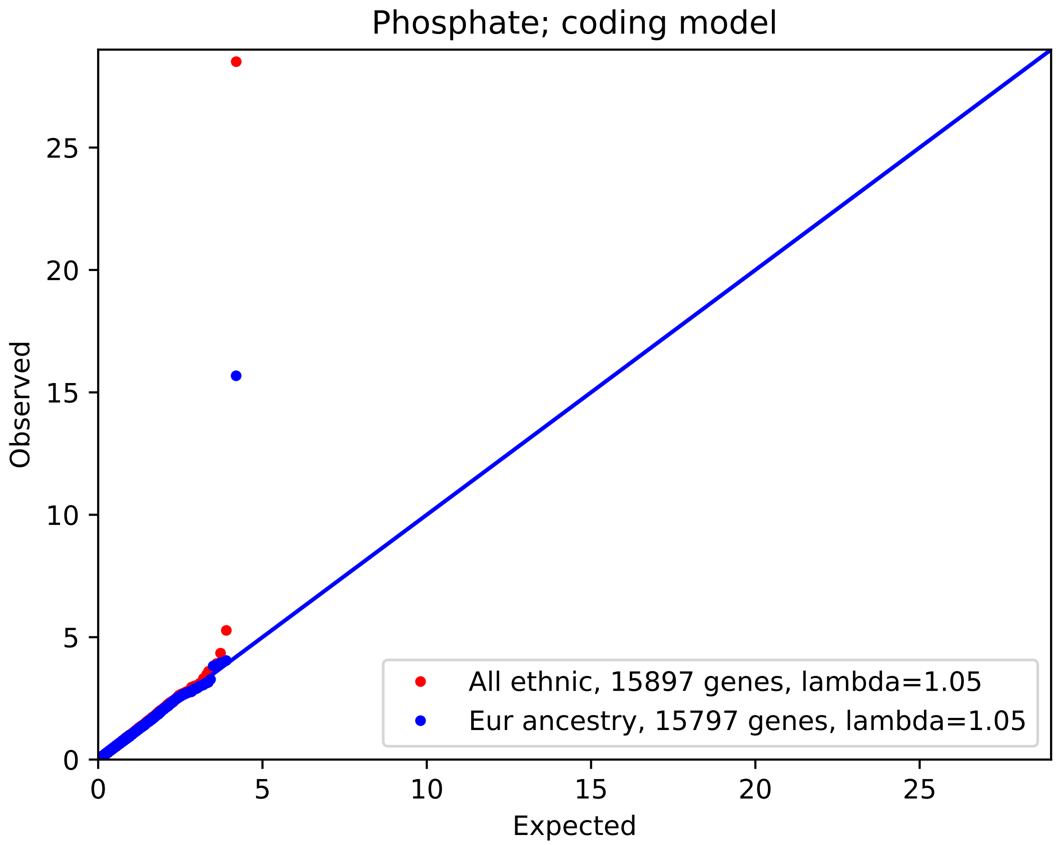
Phosphate; coding model
Observed
Expected
0
5
10
15
20
25
All ethnic, 15897 genes, lambda=1.05
Eur ancestry, 15797 genes, lambda=1.05

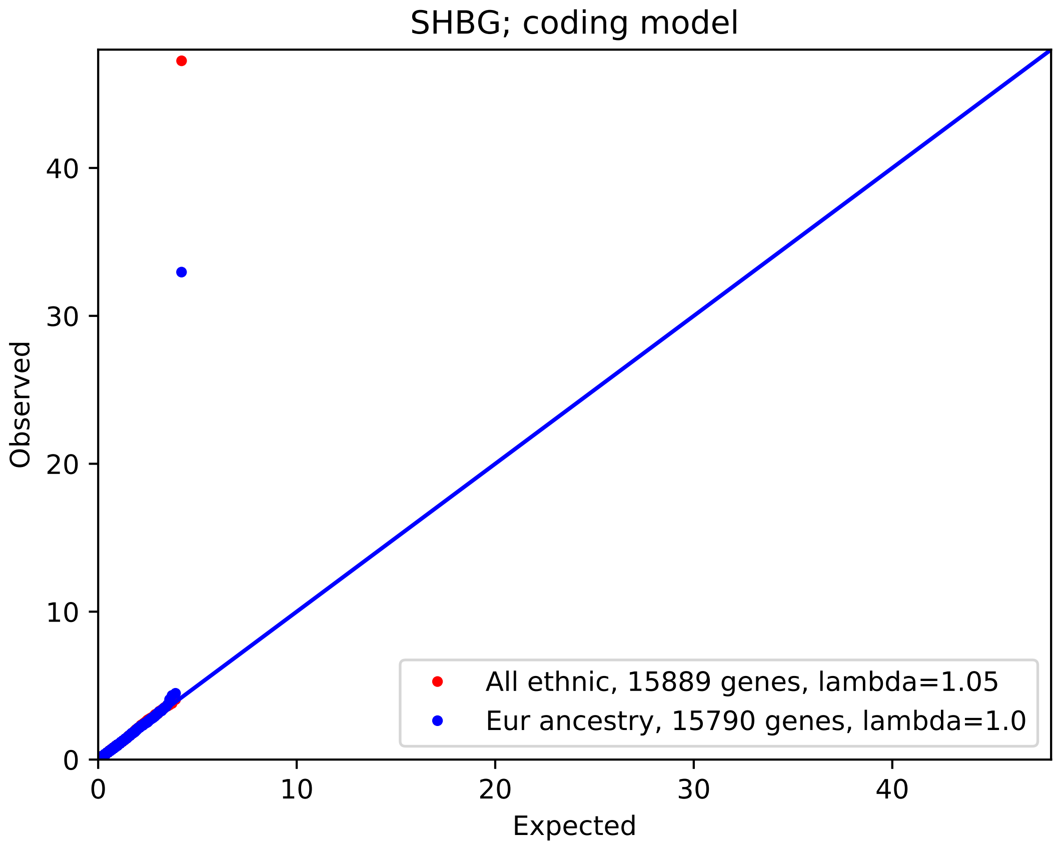

SHBG; coding model
Observed
Expected
0
10
20
30
40
0
10
20
30
40
All ethnic, 15889 genes, lambda=1.05
Eur ancestry, 15790 genes, lambda=1.0

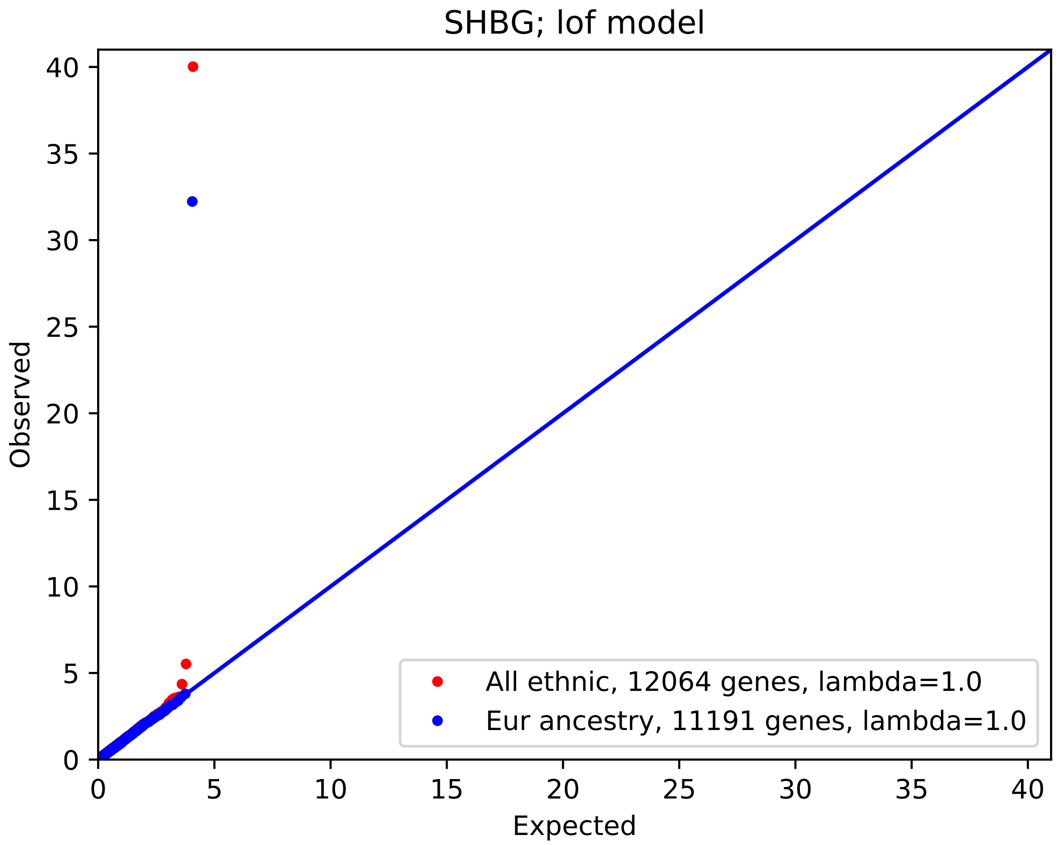

SHBG; lof model
Observed
Expected
0
5
10
15
20
25
30
35
40
All ethnic, 12064 genes, lambda=1.0
Eur ancestry, 11191 genes, lambda=1.0

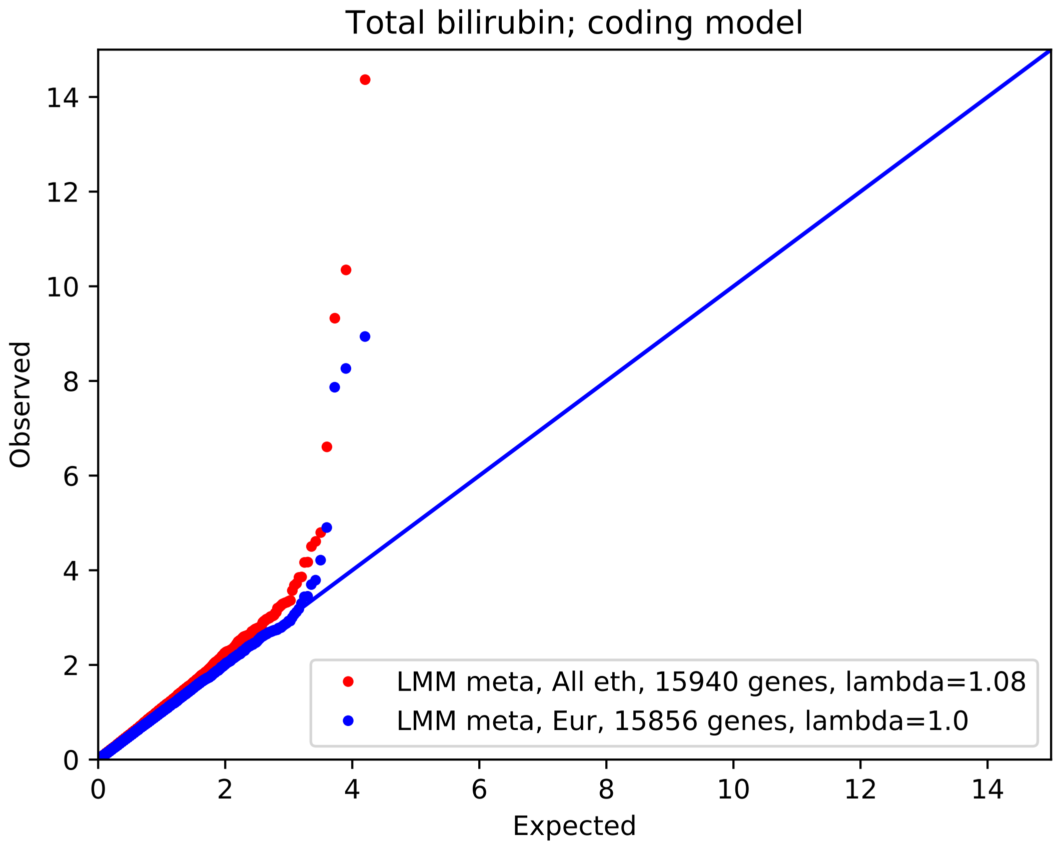
Total bilirubin; coding model
Observed
Expected
0
2
4
6
8
10
12
14
0
2
4
6
8
10
12
14
LMM meta, All eth, 15940 genes, lambda=1.08
LMM meta, Eur, 15856 genes, lambda=1.0

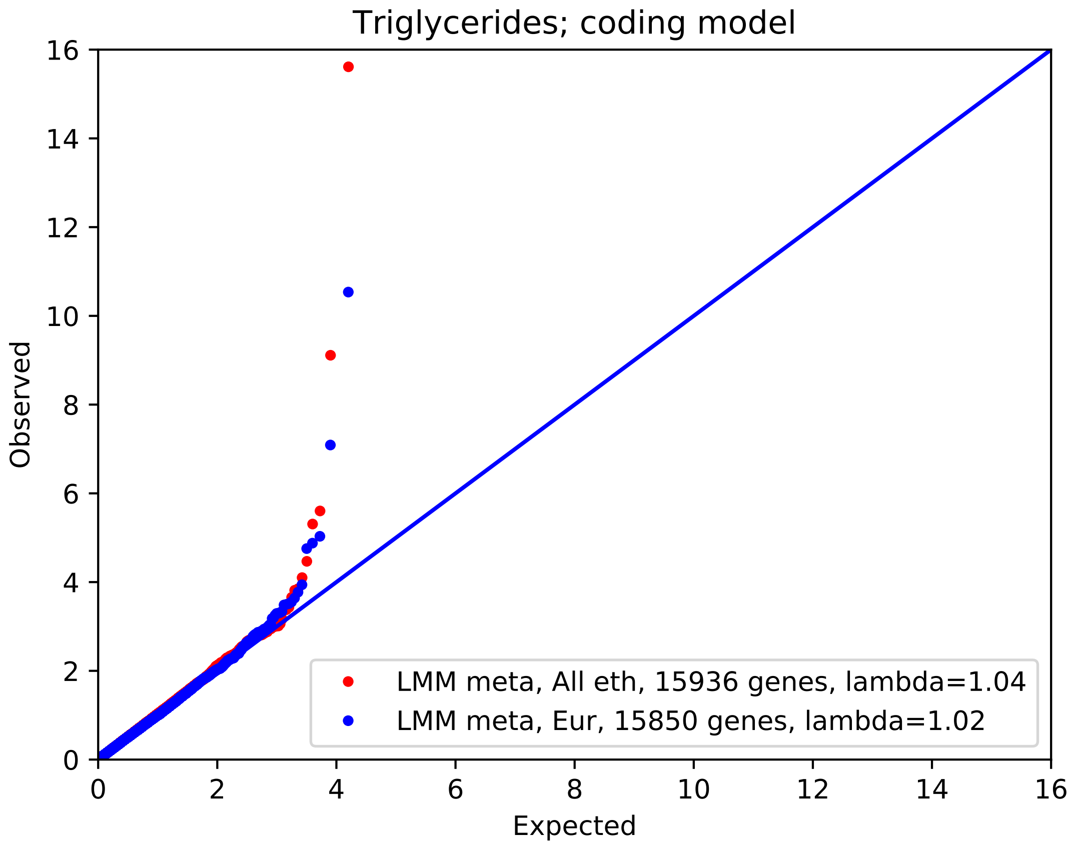
Triglycerides; coding model
Observed
Expected
0
2
4
6
8
10
12
14
16
LMM meta, All eth, 15936 genes, lambda=1.04
LMM meta, Eur, 15850 genes, lambda=1.02

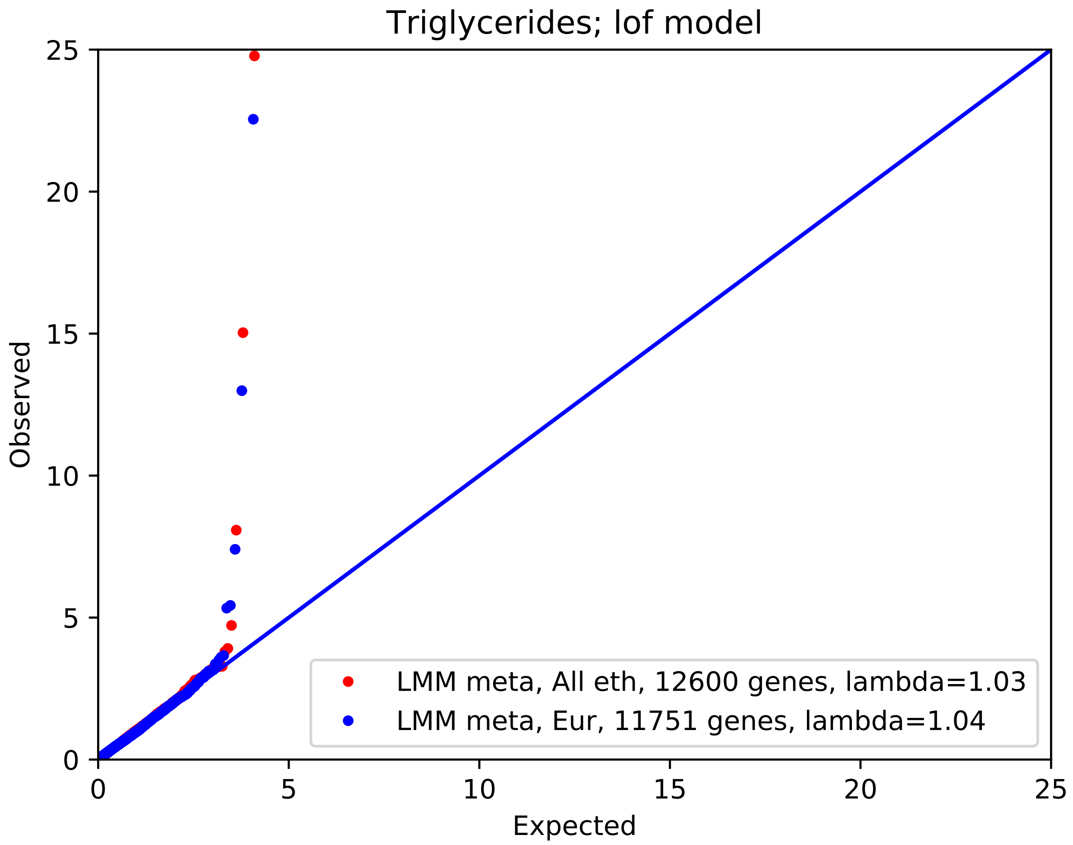
Triglycerides; lof model
Observed
Expected
0
5
10
15
20
25
LMM meta, All eth, 12600 genes, lambda=1.03
LMM meta, Eur, 11751 genes, lambda=1.04

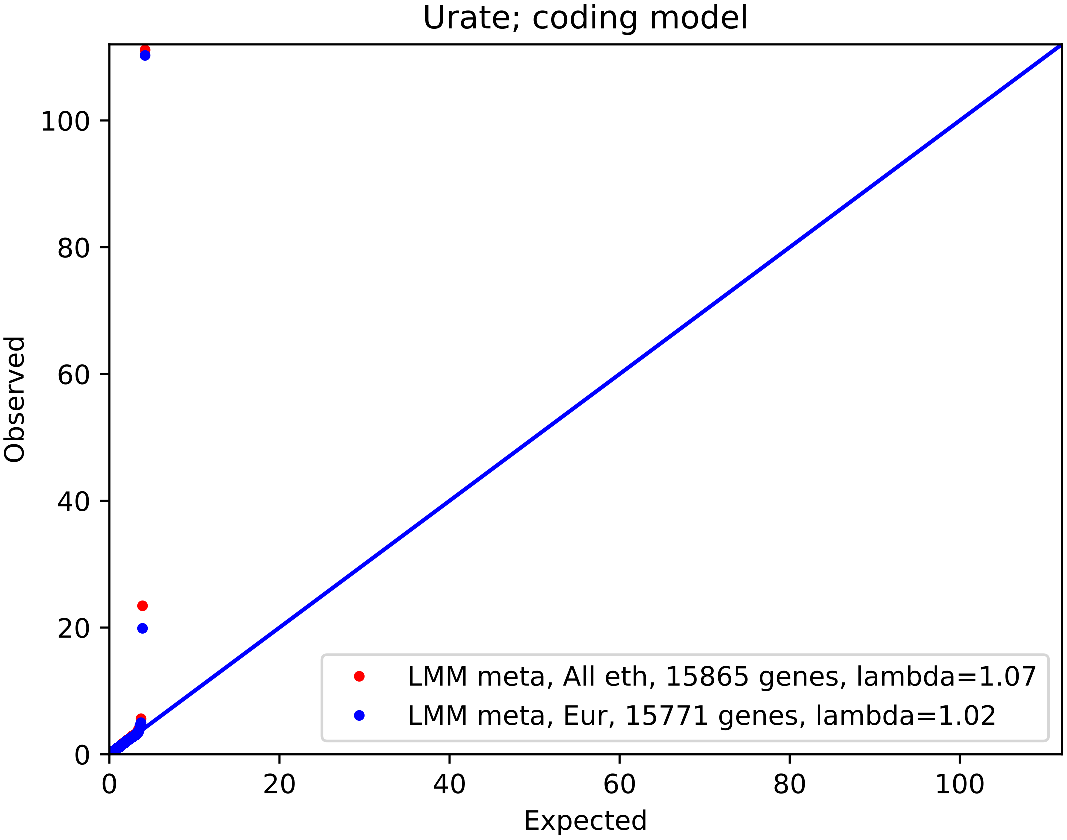
Urate; coding model
Observed
Expected
0
20
40
60
80
100
LMM meta, All eth, 15865 genes, lambda=1.07
LMM meta, Eur, 15771 genes, lambda=1.02

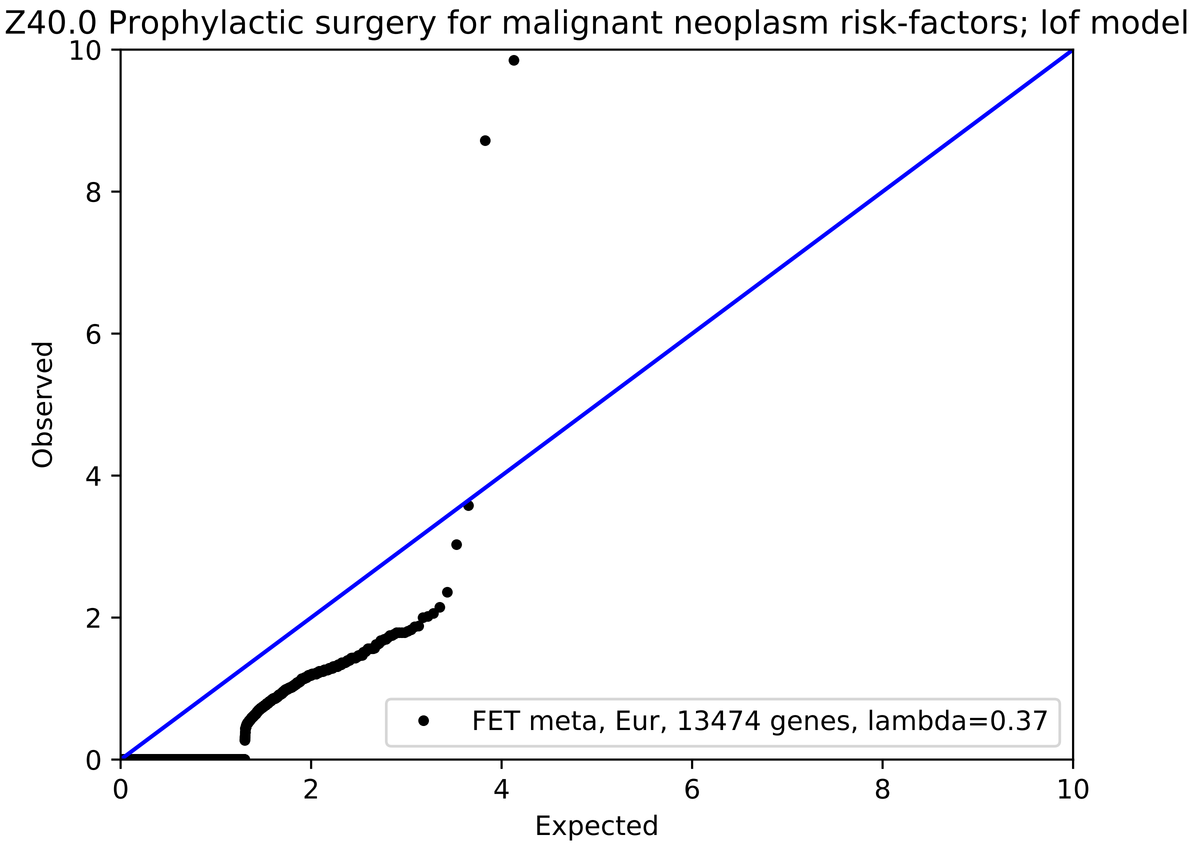
Z40.0 Prophylactic surgery for malignant neoplasm risk-factors; lof model
Observed
Expected
0
2
4
6
8
10
FET meta, Eur, 13474 genes, lambda=0.37

Supplementary Figure 2. Overlaid QQ plots for each each phenotype that produced a statistically significant association. Shown is the number of genes analyzed and resulting lambda (genomic inflation factor) for each analysis.

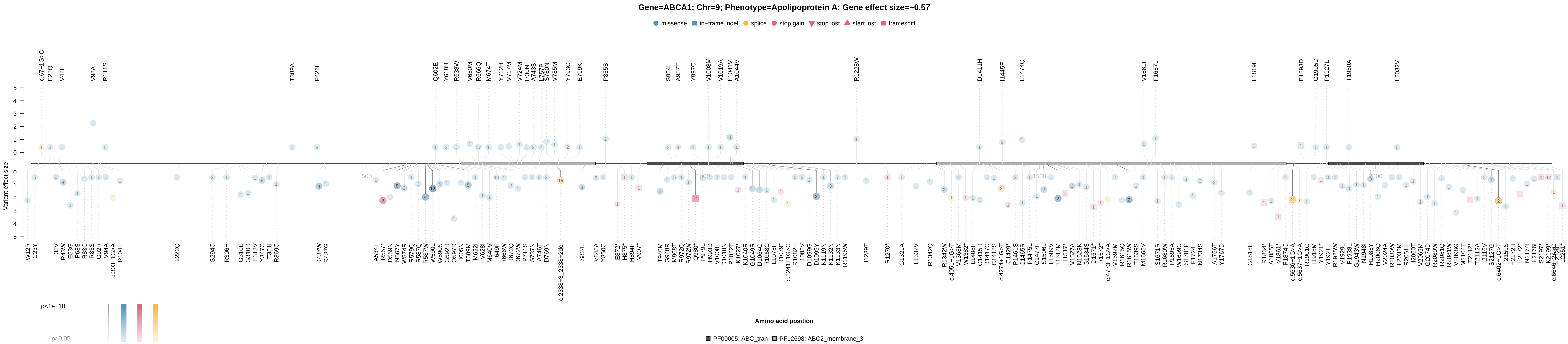

Gene=ABCA1; Chr=9; Phenotype=Apolipoprotein A; Gene effect size=−0.57
missense
in−frame indel
splice
stop gain
stop lost
start lost
frameshift
Variant effect size
Amino acid position
p<1e−10
p>0.05
PF00005: ABC_tran
PF12698: ABC2_membrane_3

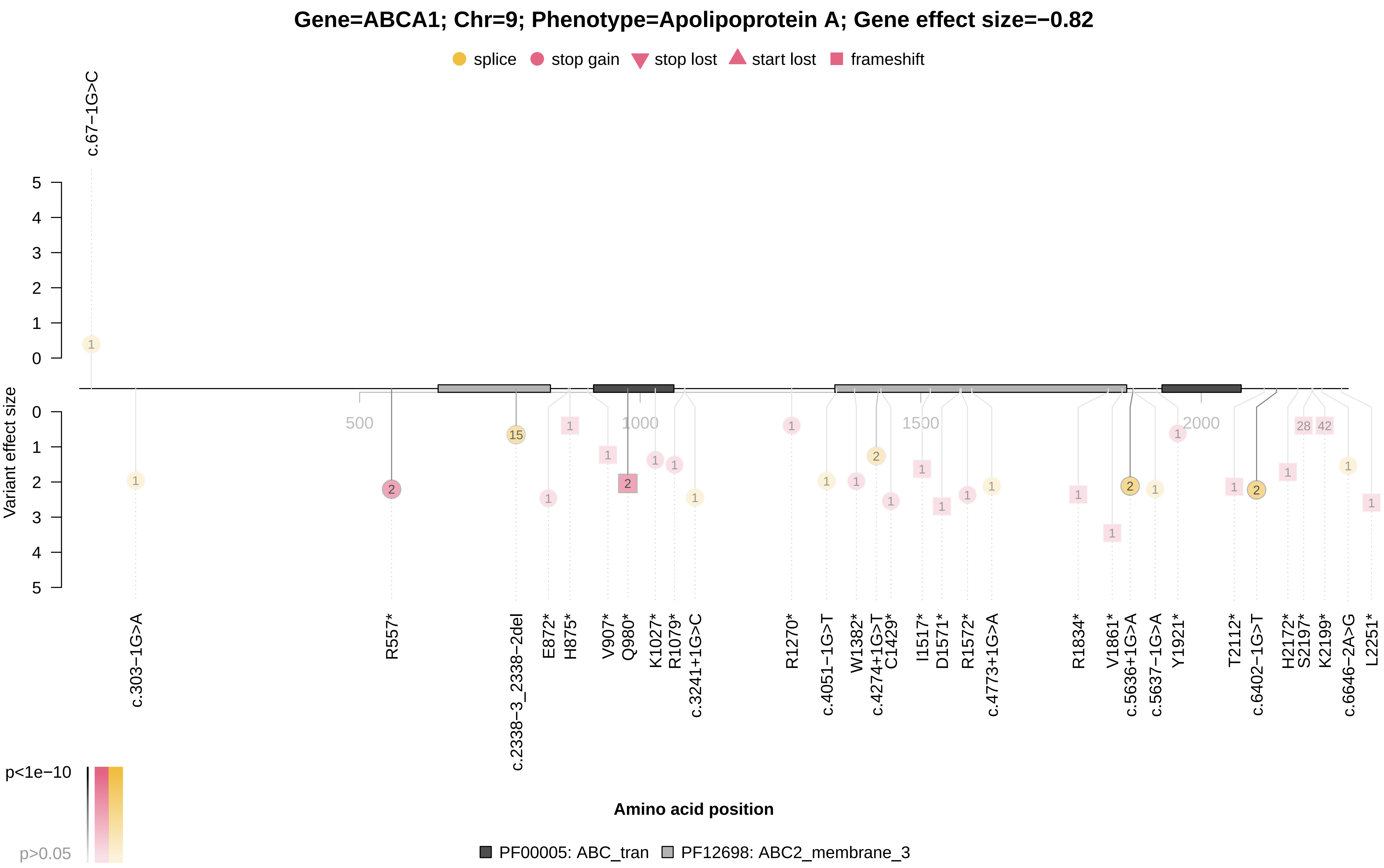

Gene=ABCA1; Chr=9; Phenotype=Apolipoprotein A; Gene effect size=−0.82
splice
stop gain
stop lost
start lost
frameshift
c.67−1G>C
Variant effect size
500
1000
1500
2000
c.303−1G>A
R557*
c.2338−3_2338−2del
E872*
H875*
V907*
Q980*
K1027*
R1079*
c.3241+1G>C
R1270*
c.4051−1G>T
W1382*
c.4274+1G>T
C1429*
I1517*
D1571*
R1572*
c.4773+1G>A
R1834*
V1861*
c.5636+1G>A
c.5637−1G>A
Y1921*
T2112*
c.6402−1G>T
H2172*
S2197*
K2199*
c.6646−2A>G
L2251*
p<1e−10
p>0.05
Amino acid position
PF00005: ABC_tran
PF12698: ABC2_membrane_3

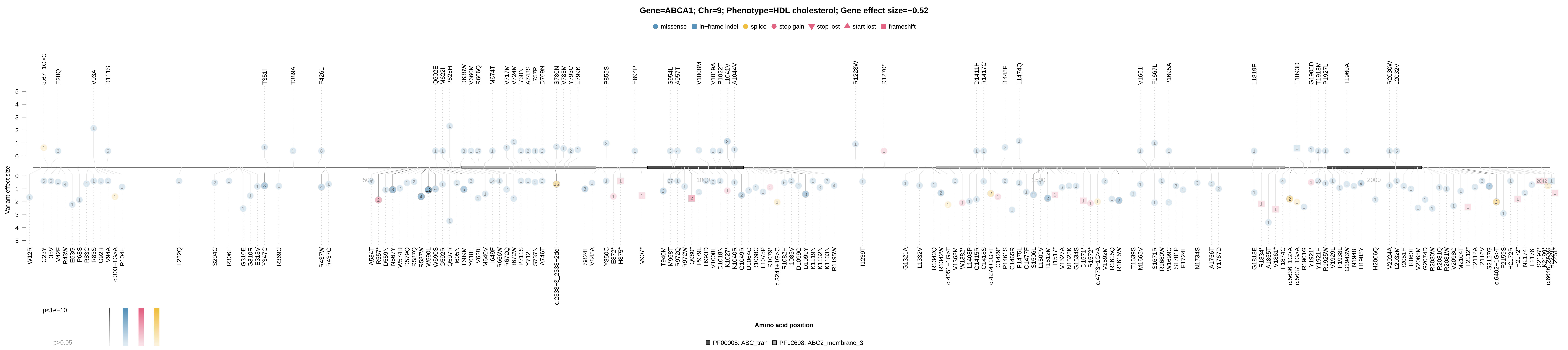

Gene=ABCA1; Chr=9; Phenotype=HDL cholesterol; Gene effect size=−0.52
missense
in−frame indel
splice
stop gain
stop lost
start lost
frameshift
Variant effect size
Amino acid position
p<1e−10
p>0.05
PF00005: ABC_tran
PF12698: ABC2_membrane_3

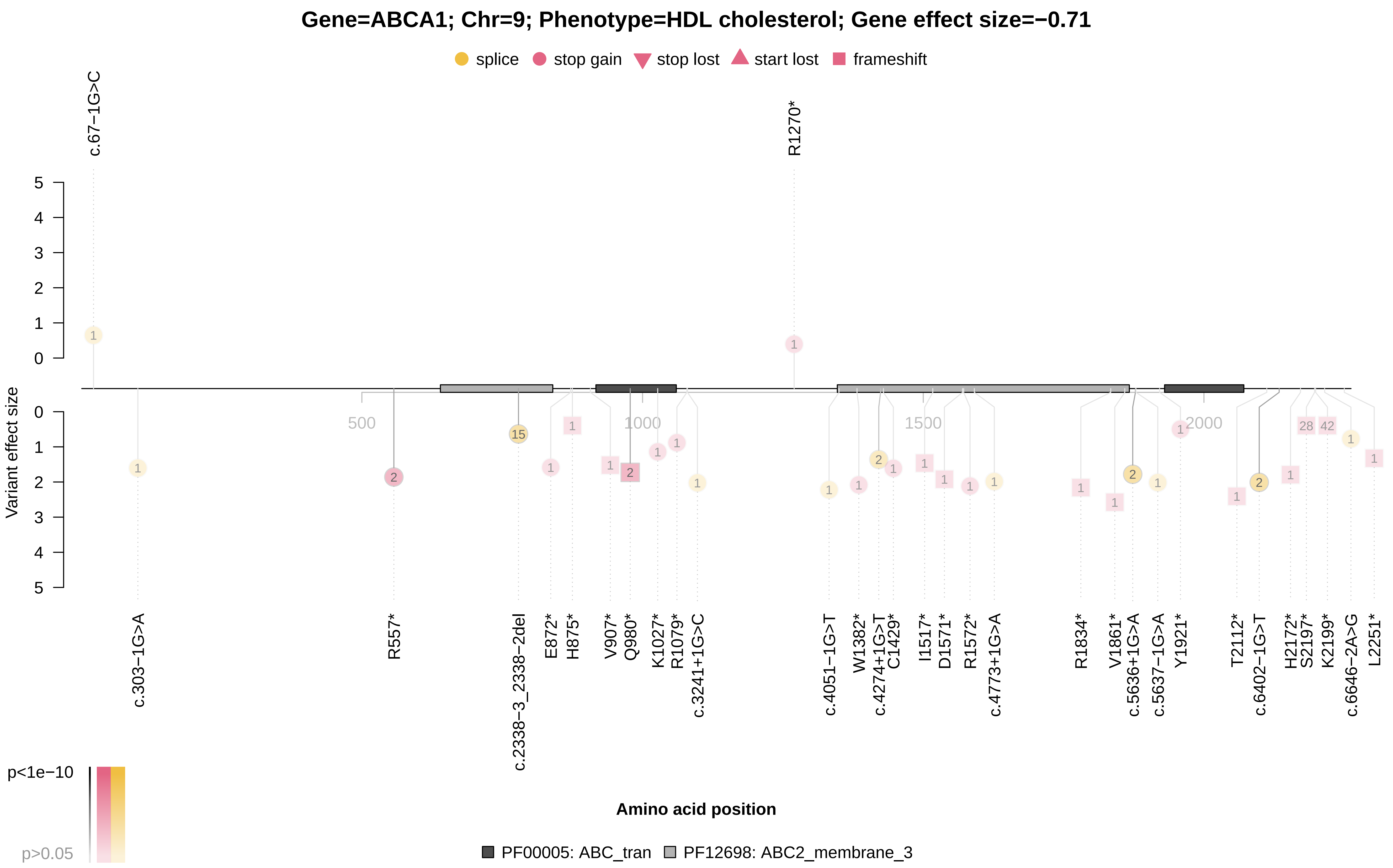

Gene=ABCA1; Chr=9; Phenotype=HDL cholesterol; Gene effect size=−0.71
splice
stop gain
stop lost
start lost
frameshift
c.67−1G>C
R1270*
Variant effect size
500
1000
1500
2000
c.303−1G>A
R557*
c.2338−3_2338−2del
E872*
H875*
V907*
Q980*
K1027*
R1079*
c.3241+1G>C
c.4051−1G>T
W1382*
c.4274+1G>T
C1429*
I1517*
D1571*
R1572*
c.4773+1G>A
R1834*
V1861*
c.5636+1G>A
c.5637−1G>A
Y1921*
T2112*
c.6402−1G>T
H2172*
S2197*
K2199*
c.6646−2A>G
L2251*
p<1e−10
p>0.05
Amino acid position
PF00005: ABC_tran
PF12698: ABC2_membrane_3

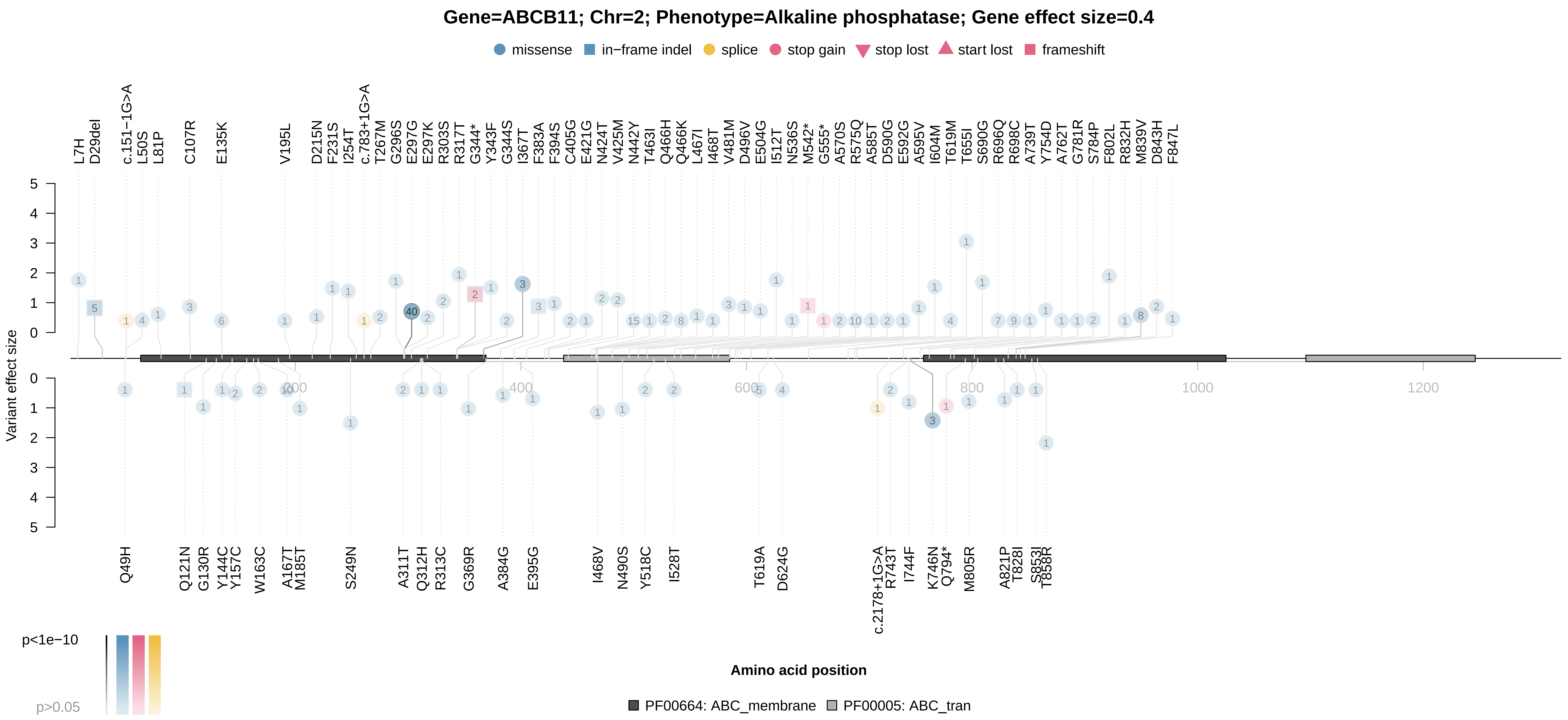

Gene=ABCB11; Chr=2; Phenotype=Alkaline phosphatase; Gene effect size=0.4
missense
in−frame indel
splice
stop gain
stop lost
start lost
frameshift
L7H
D29del
c.151−1G>A
L50S
L81P
C107R
E135K
V195L
D215N
F231S
I254T
c.783+1G>A
T267M
G296S
E297G
E297K
R303S
R317T
G344*
Y343F
G344S
I367T
F383A
F394S
C405G
E421G
N424T
V425M
N442Y
T463I
Q466H
Q466K
L467I
I468T
V481M
D496V
E504G
I512T
N536S
M542*
G555*
A570S
R575Q
A585T
D590G
E592G
A595V
I604M
T619M
T655I
S690G
R696Q
R698C
A739T
Y754D
A762T
G781R
S784P
F802L
R832H
M839V
D843H
F847L
Variant effect size
5
4
3
2
1
0
0
1
2
3
4
5
200
400
600
800
1000
1200
Q49H
Q121N
G130R
Y144C
Y157C
W163C
A167T
M185T
S249N
A311T
Q312H
R313C
G369R
A384G
E395G
I468V
N490S
Y518C
I528T
T619A
D624G
c.2178+1G>A
R743T
I744F
K746N
Q794*
M805R
A821P
T828I
S853I
T858R
p<1e−10
p>0.05
Amino acid position
PF00664: ABC_membrane
PF00005: ABC_tran

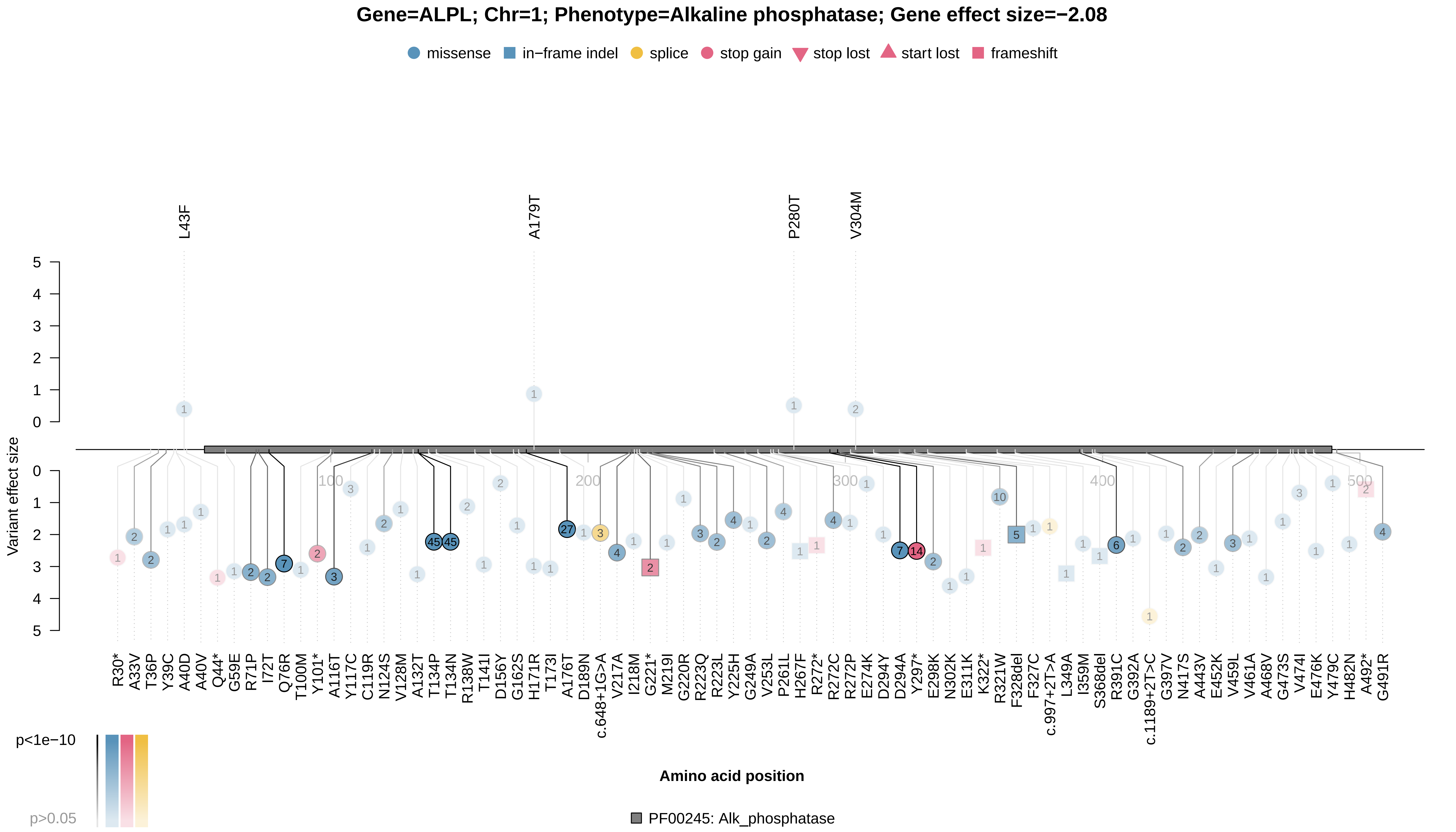

Gene=ALPL; Chr=1; Phenotype=Alkaline phosphatase; Gene effect size=−2.08
missense
in−frame indel
splice
stop gain
stop lost
start lost
frameshift
L43F
A179T
P280T
V304M
Variant effect size
Amino acid position
p<1e−10
p>0.05
PF00245: Alk_phosphatase
R30* A33V T36P Y39C A40D A40V Q44* G59E R71P I72T Q76R T100M Y101* A116T Y117C C119R N124S V128M A132T T134P T134N R138W T141I D156Y G162S H171R T173I A176T D189N c.648+1G>A V217A I218M G221* M219I G220R R223Q R223L Y225H G249A V253L P261L H267F R272* R272C R272P E274K D294Y D294A Y297* E298K N302K E311K K322* R321W F328del F327C c.997+2T>A L349A I359M S368del R391C G392A c.1189+2T>C G397V N417S A443V E452K V459L V461A A468V G473S V474I E476K Y479C H482N A492* G491R

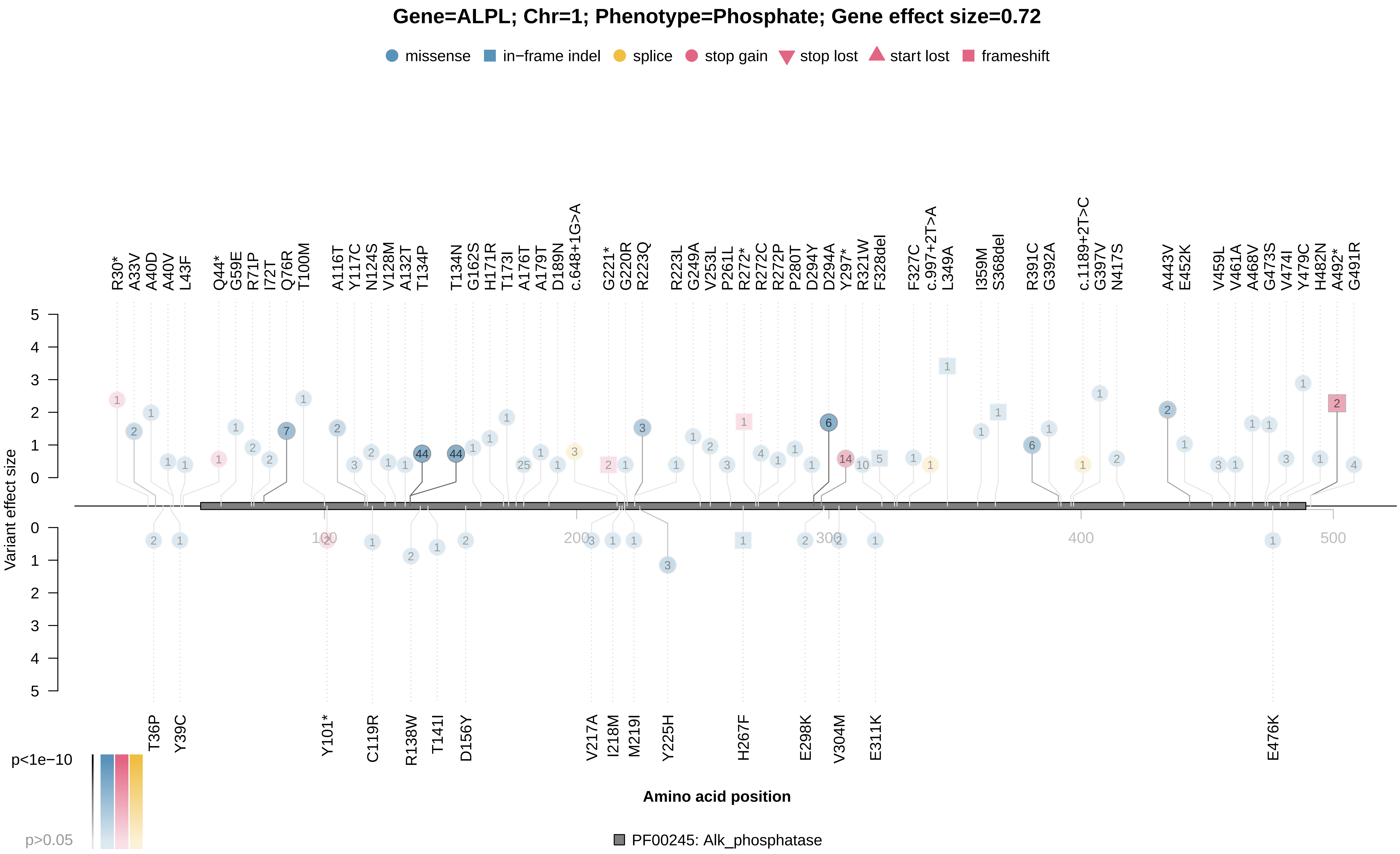
Gene=ALPL; Chr=1; Phenotype=Phosphate; Gene effect size=0.72
missense
in−frame indel
splice
stop gain
stop lost
start lost
frameshift
Variant effect size
Amino acid position
p<1e−10
p>0.05
PF00245: Alk_phosphatase

Gene=ANGPTL3; Chr=1; Phenotype=Apolipoprotein A; Gene effect size=−0.52

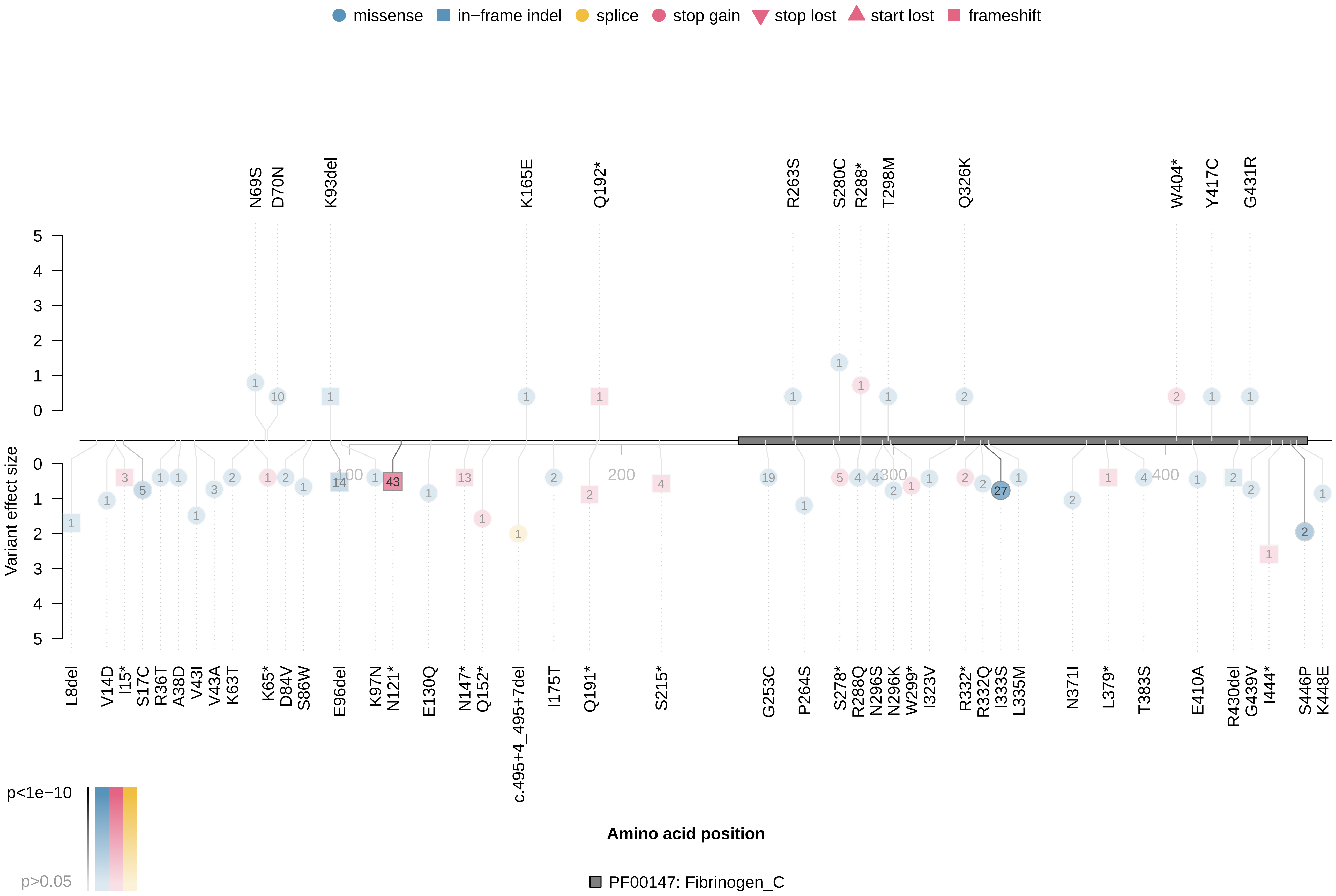

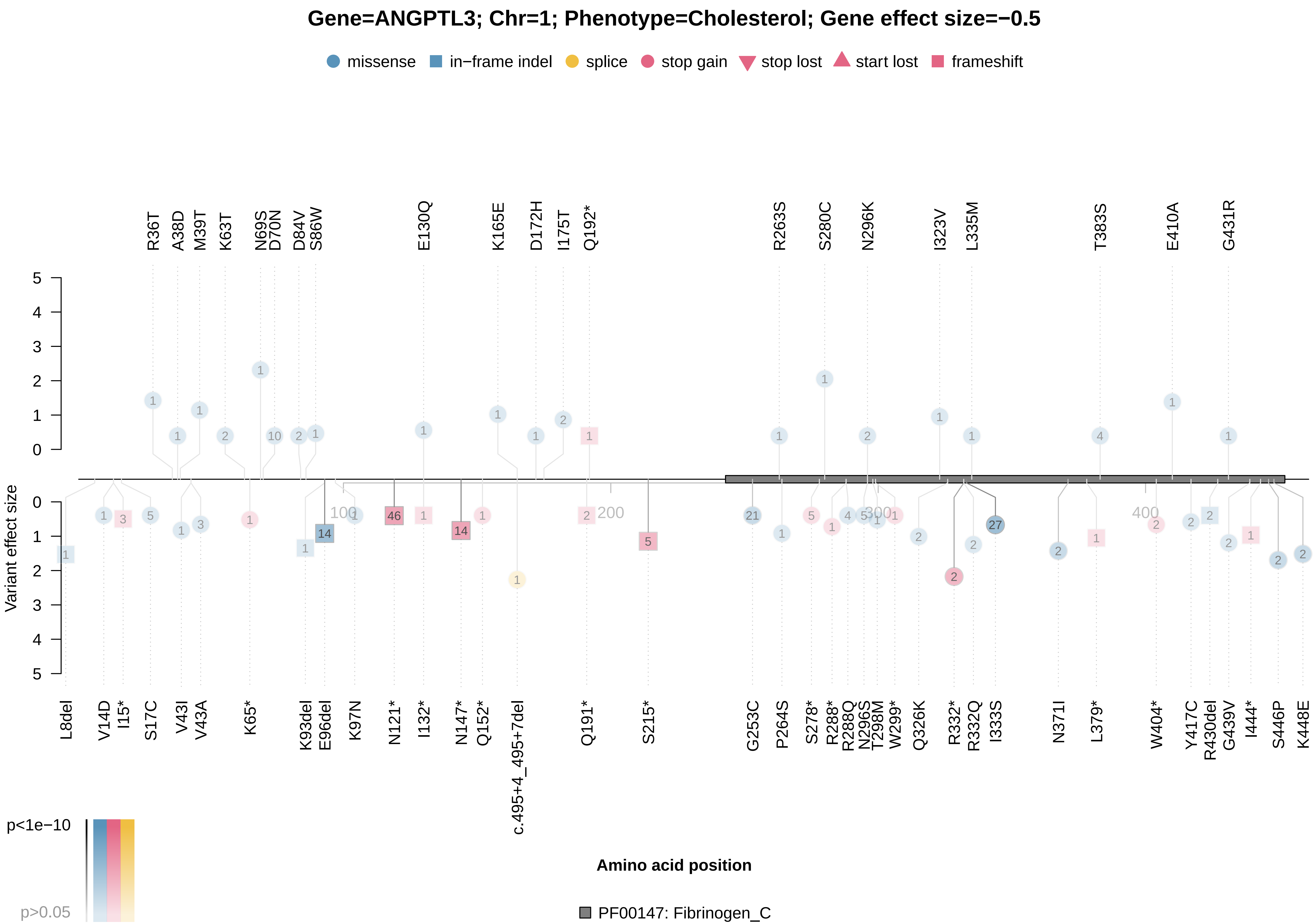

Gene=ANGPTL3; Chr=1; Phenotype=Cholesterol; Gene effect size=−0.5
missense
in−frame indel
splice
stop gain
stop lost
start lost
frameshift
Variant effect size
Amino acid position
p<1e−10
p>0.05
PF00147: Fibrinogen_C
R36T
A38D
M39T
K63T
N69S
D70N
D84V
S86W
E130Q
K165E
D172H
I175T
Q192*
R263S
S280C
N296K
I323V
L335M
T383S
E410A
G431R
L8del
V14D
I15*
S17C
V43I
V43A
K65*
K93del
E96del
K97N
N121*
I132*
N147*
Q152*
c.495+4_495+7del
Q191*
S215*
G253C
P264S
S278*
R288*
R288Q
N296S
T298M
W299*
Q326K
R332*
R332Q
I333S
N371I
L379*
W404*
Y417C
R430del
G439V
I444*
S446P
K448E

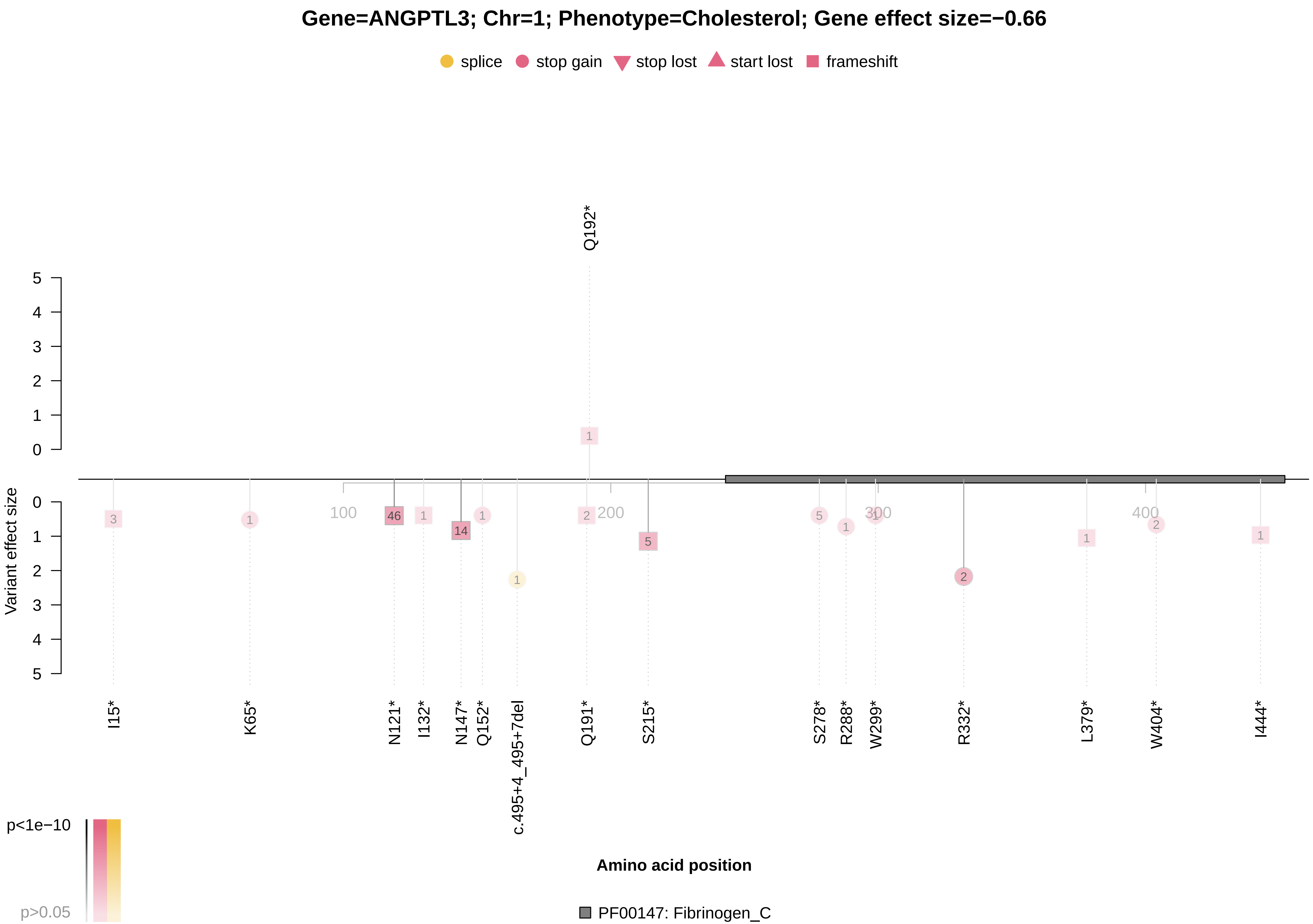

Gene=ANGPTL3; Chr=1; Phenotype=Cholesterol; Gene effect size=−0.66
splice
stop gain
stop lost
start lost
frameshift
Q192*
Variant effect size
100
200
300
400
I15*
K65*
N121*
I132*
N147*
Q152*
c.495+4_495+7del
Q191*
S215*
S278*
R288*
W299*
R332*
L379*
W404*
I444*
p<1e−10
p>0.05
Amino acid position
PF00147: Fibrinogen_C

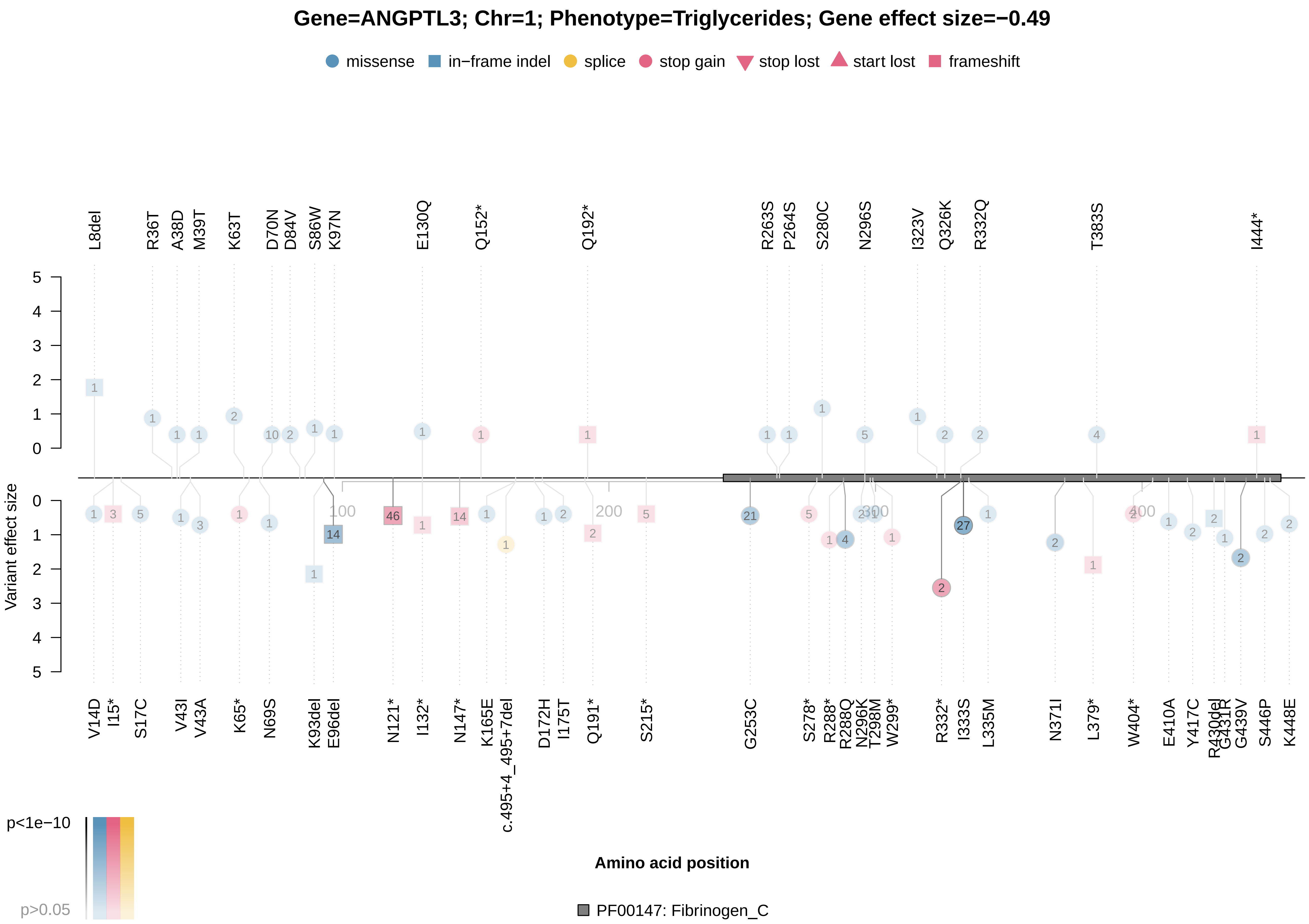

Gene=ANGPTL3; Chr=1; Phenotype=Triglycerides; Gene effect size=−0.49
missense
in−frame indel
splice
stop gain
stop lost
start lost
frameshift
L8del
R36T
A38D
M39T
K63T
D70N
D84V
S86W
K97N
E130Q
Q152*
Q192*
R263S
P264S
S280C
N296S
I323V
Q326K
R332Q
T383S
I444*
Variant effect size
100
200
300
400
V14D
I15*
S17C
V43I
V43A
K65*
N69S
K93del
E96del
N121*
I132*
N147*
K165E
c.495+4_495+7del
D172H
I175T
Q191*
S215*
G253C
S278*
R288*
R288Q
N296K
T298M
W299*
R332*
I333S
L335M
N371I
L379*
W404*
E410A
Y417C
R430del
G431R
G439V
S446P
K448E
p<1e−10
p>0.05
Amino acid position
PF00147: Fibrinogen_C

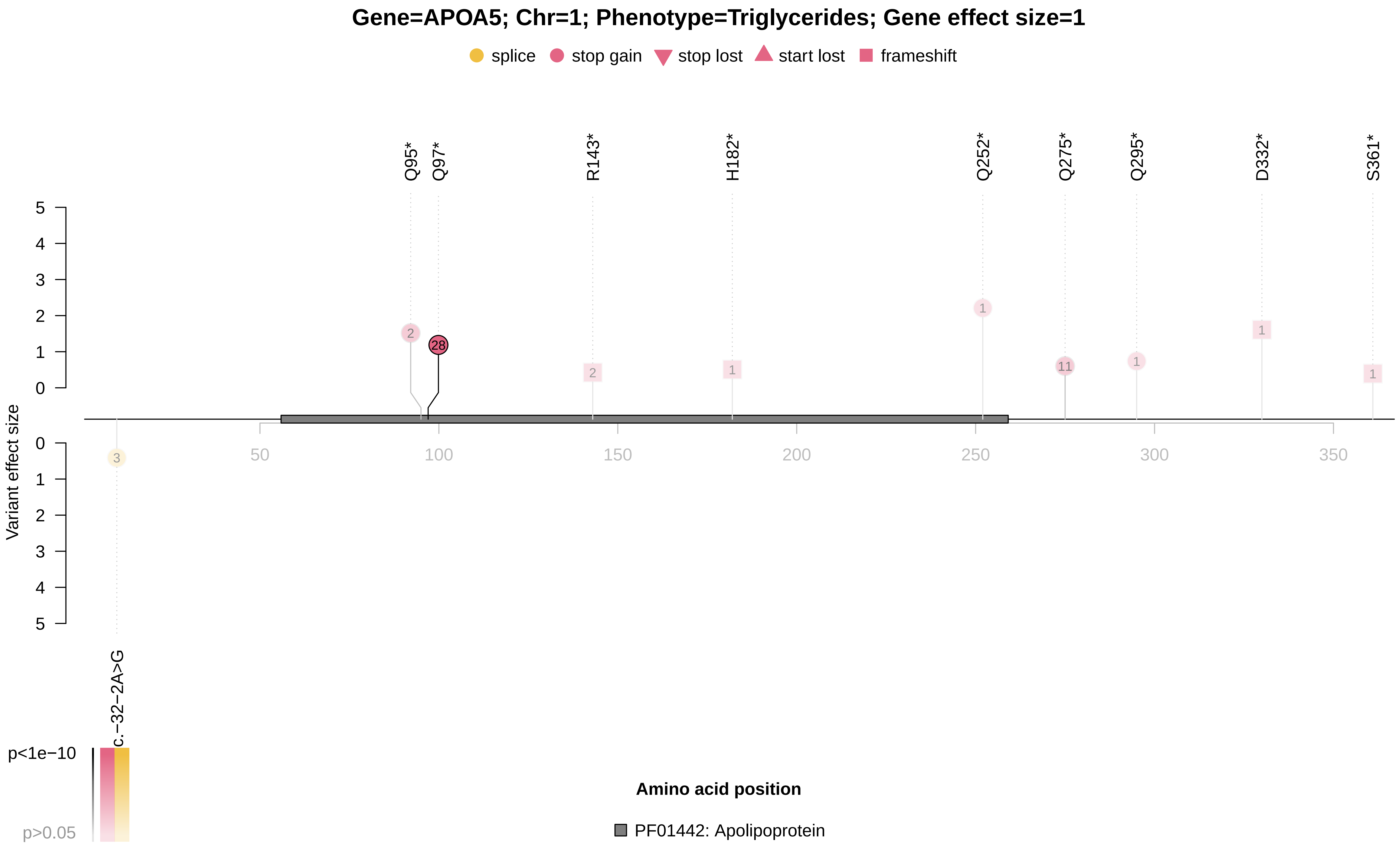

Gene=APOA5; Chr=1; Phenotype=Triglycerides; Gene effect size=1
splice
stop gain
stop lost
start lost
frameshift
Q95*
Q97*
R143*
H182*
Q252*
Q275*
Q295*
D332*
S361*
Variant effect size
5
4
3
2
1
0
2
28
2
1
1
11
1
1
1
3
50
100
150
200
250
300
350
c.−32−2A>G
p<1e−10
p>0.05
Amino acid position
PF01442: Apolipoprotein

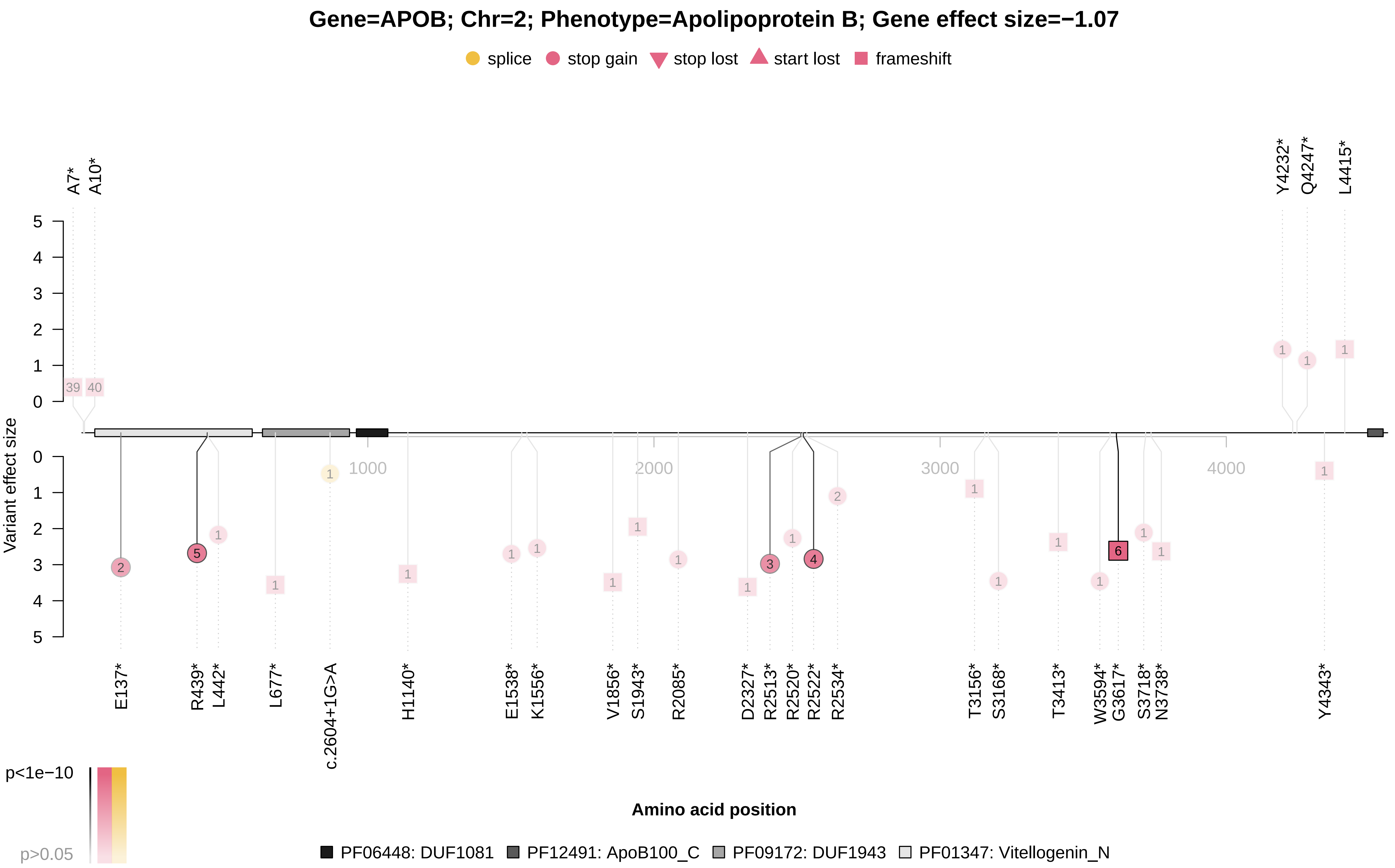

Gene=APOB; Chr=2; Phenotype=Apolipoprotein B; Gene effect size=−1.07
splice
stop gain
stop lost
start lost
frameshift
A7*
A10*
Y4232*
Q4247*
L4415*
Variant effect size
1000
2000
3000
4000
E137*
R439*
L442*
L677*
c.2604+1G>A
H1140*
E1538*
K1556*
V1856*
S1943*
R2085*
D2327*
R2513*
R2520*
R2522*
R2534*
T3156*
S3168*
T3413*
W3594*
G3617*
S3718*
N3738*
Y4343*
p<1e−10
p>0.05
Amino acid position
PF06448: DUF1081
PF12491: ApoB100_C
PF09172: DUF1943
PF01347: Vitellogenin_N

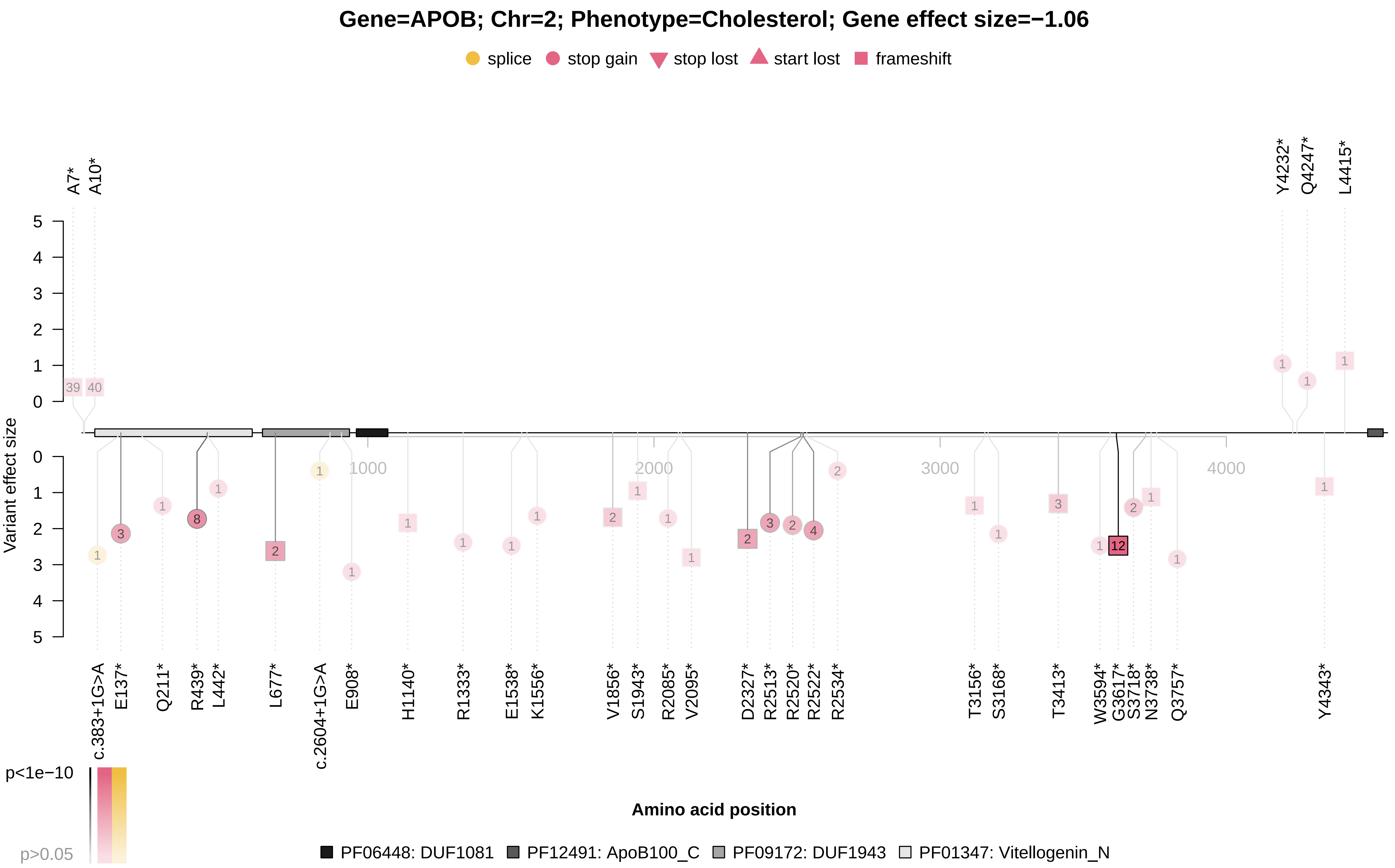

Gene=APOB; Chr=2; Phenotype=Cholesterol; Gene effect size=−1.06
splice
stop gain
stop lost
start lost
frameshift
A7*
A10*
Y4232*
Q4247*
L4415*
Variant effect size
Amino acid position
1000
2000
3000
4000
c.383+1G>A
E137*
Q211*
R439*
L442*
L677*
c.2604+1G>A
E908*
H1140*
R1333*
E1538*
K1556*
V1856*
S1943*
R2085*
V2095*
D2327*
R2513*
R2520*
R2522*
R2534*
T3156*
S3168*
T3413*
W3594*
G3617*
S3718*
N3738*
Q3757*
Y4343*
p<1e−10
p>0.05
PF06448: DUF1081
PF12491: ApoB100_C
PF09172: DUF1943
PF01347: Vitellogenin_N

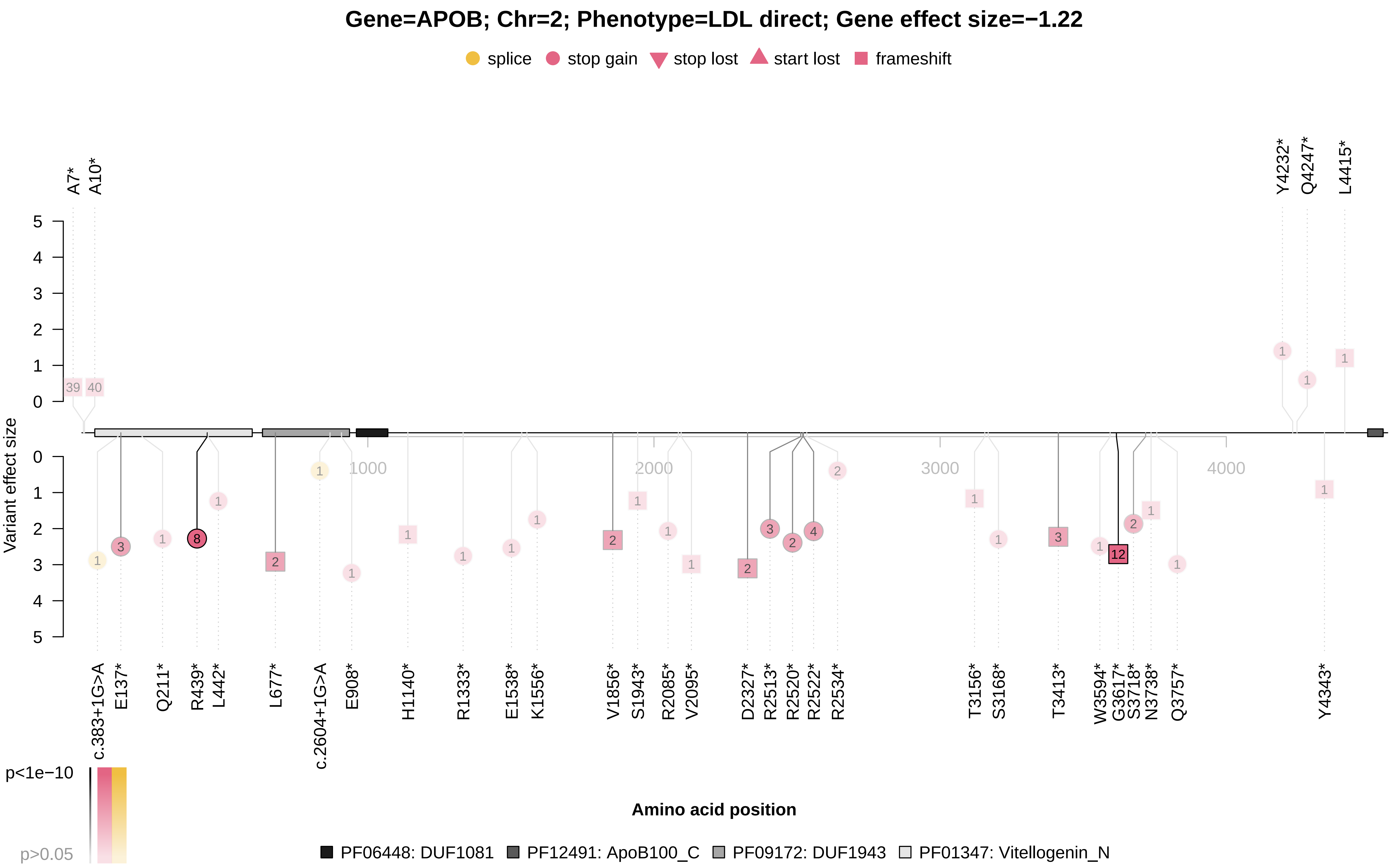
Gene=APOB; Chr=2; Phenotype=LDL direct; Gene effect size=−1.22
splice
stop gain
stop lost
start lost
frameshift
A7*
A10*
Y4232*
Q4247*
L4415*
Variant effect size
1000
2000
3000
4000
c.383+1G>A
E137*
Q211*
R439*
L442*
L677*
c.2604+1G>A
E908*
H1140*
R1333*
E1538*
K1556*
V1856*
S1943*
R2085*
V2095*
D2327*
R2513*
R2520*
R2522*
R2534*
T3156*
S3168*
T3413*
W3594*
G3617*
S3718*
N3738*
Q3757*
Y4343*
p<1e−10
p>0.05
Amino acid position
PF06448: DUF1081
PF12491: ApoB100_C
PF09172: DUF1943
PF01347: Vitellogenin_N

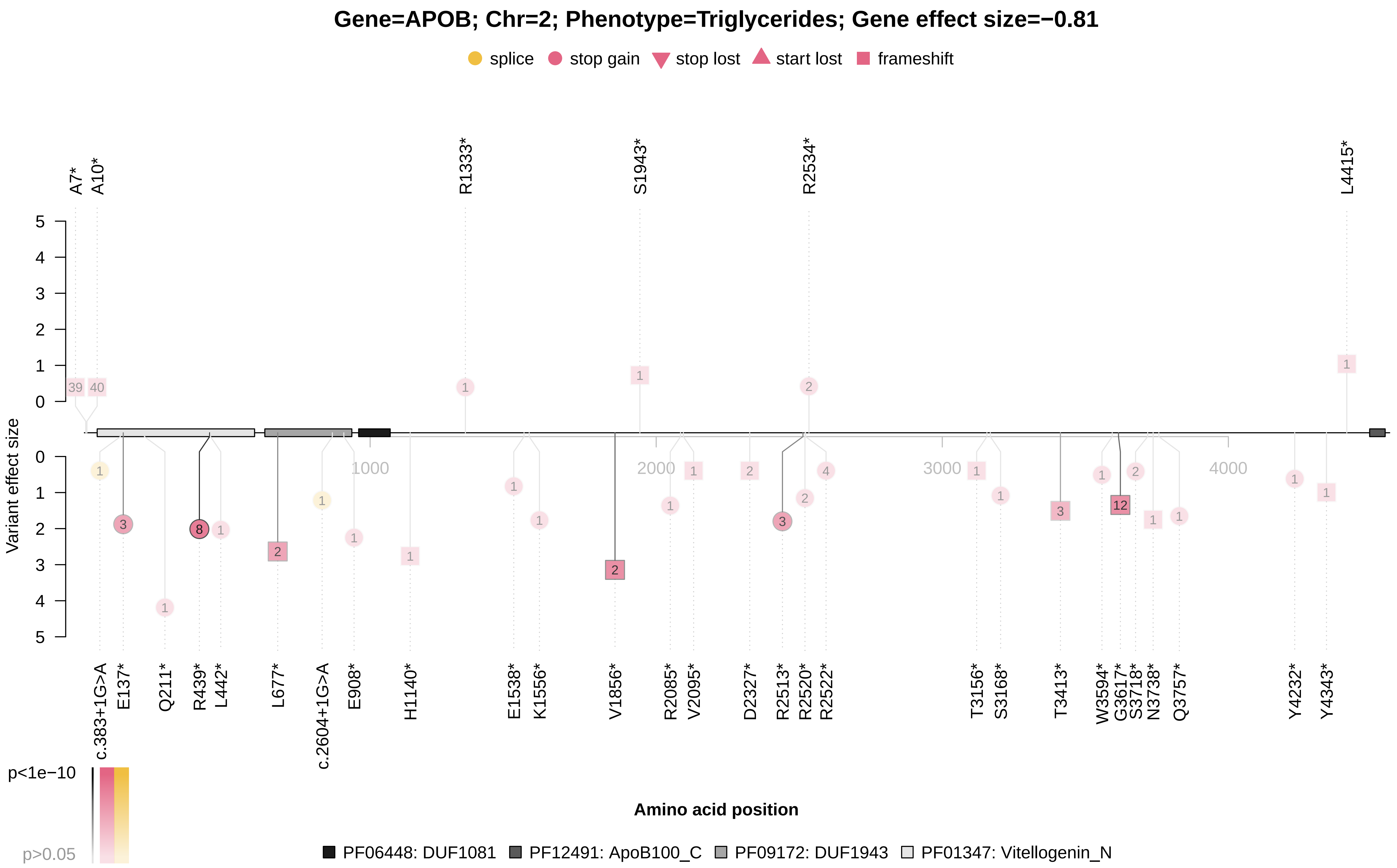

Gene=APOB; Chr=2; Phenotype=Triglycerides; Gene effect size=−0.81
splice
stop gain
stop lost
start lost
frameshift
Variant effect size
Amino acid position
p<1e−10
p>0.05
A7*
A10*
R1333*
S1943*
R2534*
L4415*
c.383+1G>A
E137*
Q211*
R439*
L442*
L677*
c.2604+1G>A
E908*
H1140*
E1538*
K1556*
V1856*
R2085*
V2095*
D2327*
R2513*
R2520*
R2522*
T3156*
S3168*
T3413*
W3594*
G3617*
S3718*
N3738*
Q3757*
Y4232*
Y4343*
PF06448: DUF1081
PF12491: ApoB100_C
PF09172: DUF1943
PF01347: Vitellogenin_N

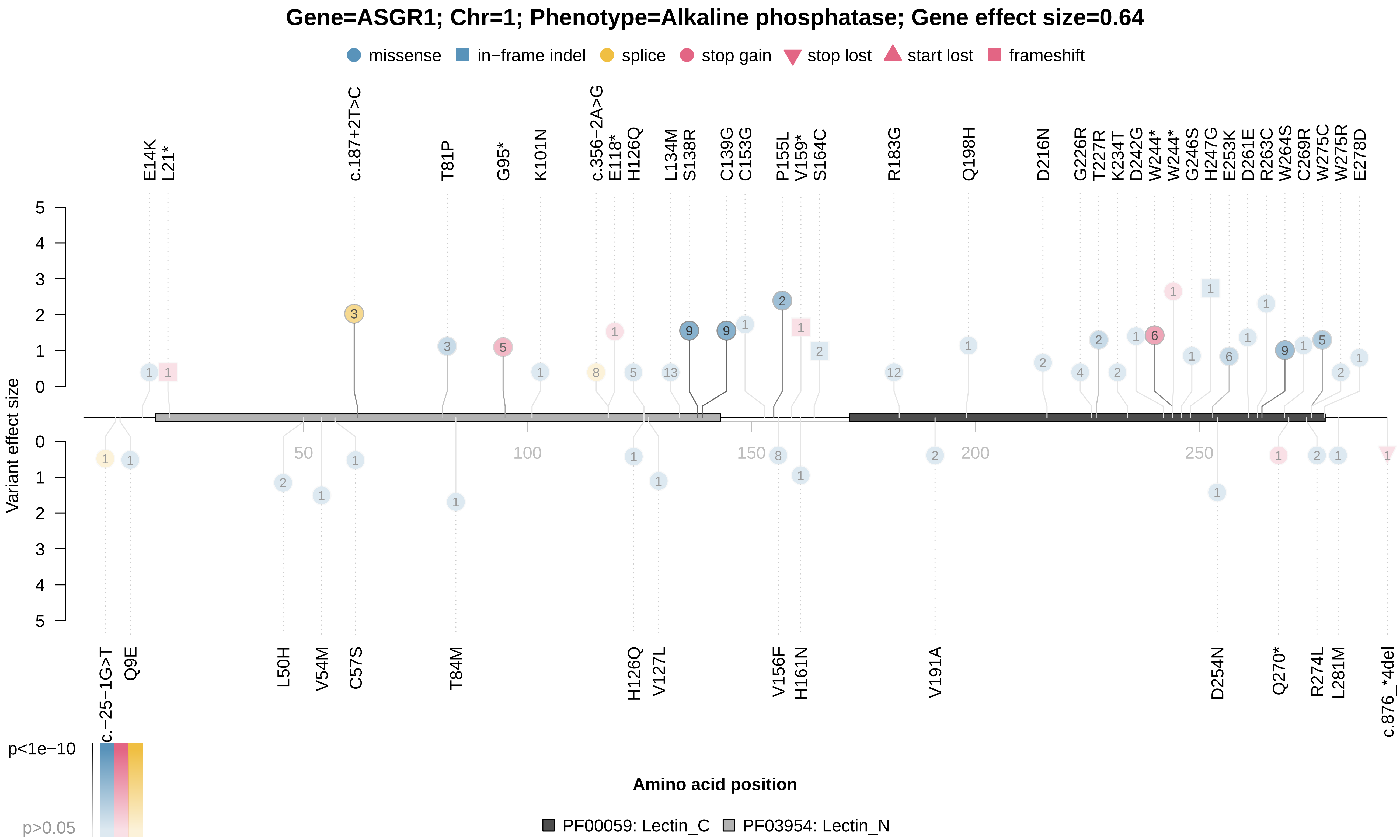

Gene=ASGR1; Chr=1; Phenotype=Alkaline phosphatase; Gene effect size=0.64
missense
in−frame indel
splice
stop gain
stop lost
start lost
frameshift
E14K L21* c.187+2T>C T81P G95* K101N c.356−2A>G E118* H126Q L134M S138R C139G C153G P155L V159* S164C R183G Q198H D216N G226R T227R K234T D242G W244* W244* G246S H247G E253K D261E R263C W264S C269R W275C W275R E278D
c.−25−1G>T Q9E L50H V54M C57S T84M H126Q V127L V156F H161N V191A D254N Q270* R274L L281M c.876_*4del
Variant effect size
Amino acid position
p<1e−10
p>0.05
PF00059: Lectin_C
PF03954: Lectin_N

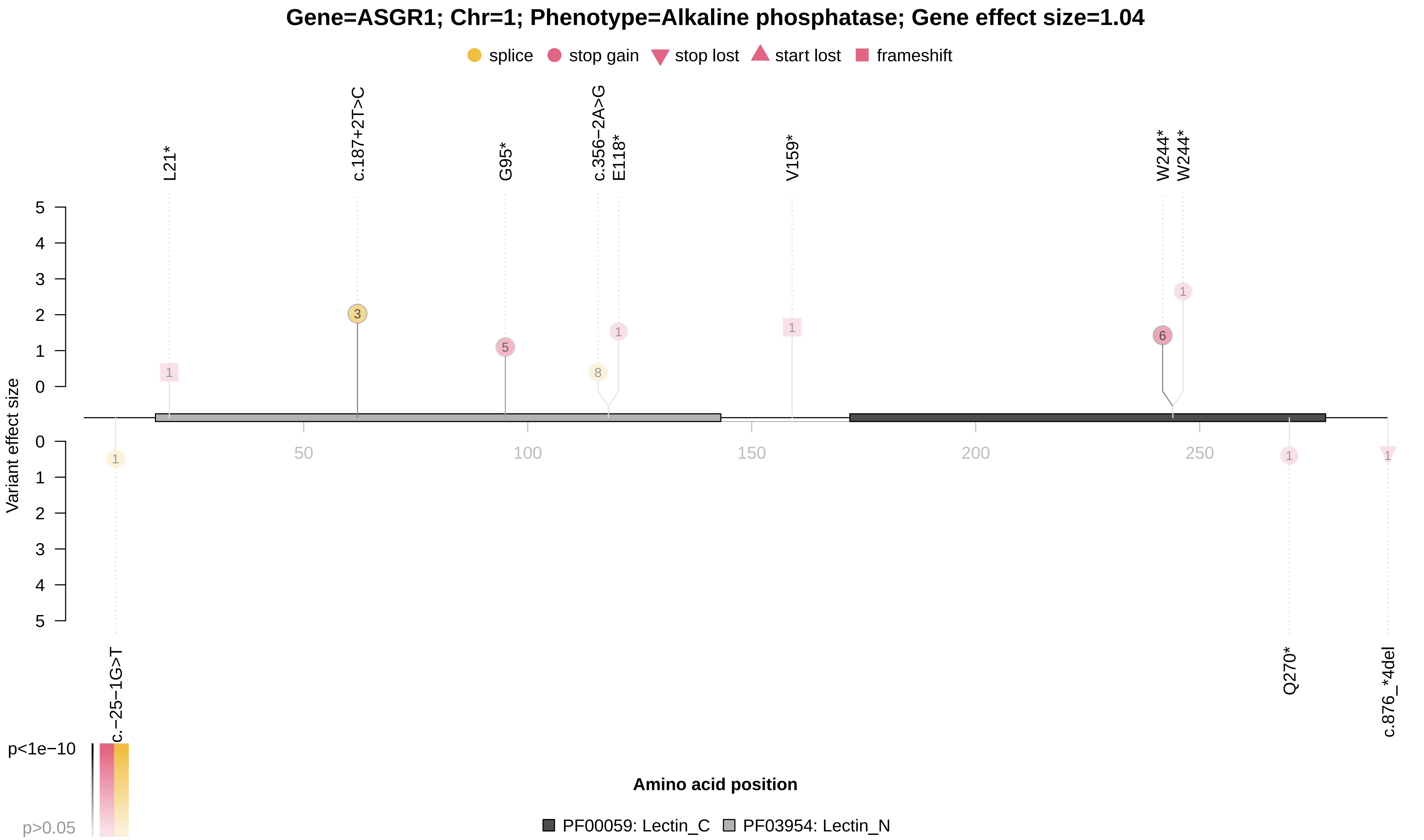

Gene=ASGR1; Chr=1; Phenotype=Alkaline phosphatase; Gene effect size=1.04
splice
stop gain
stop lost
start lost
frameshift
L21*
c.187+2T>C
G95*
c.356−2A>G
E118*
V159*
W244*
W244*
Variant effect size
5
4
3
2
1
0
0
1
2
3
4
5
1
3
5
8
1
1
6
1
1
1
1
50
100
150
200
250
c.−25−1G>T
Q270*
c.876_*4del
p<1e−10
p>0.05
Amino acid position
PF00059: Lectin_C
PF03954: Lectin_N

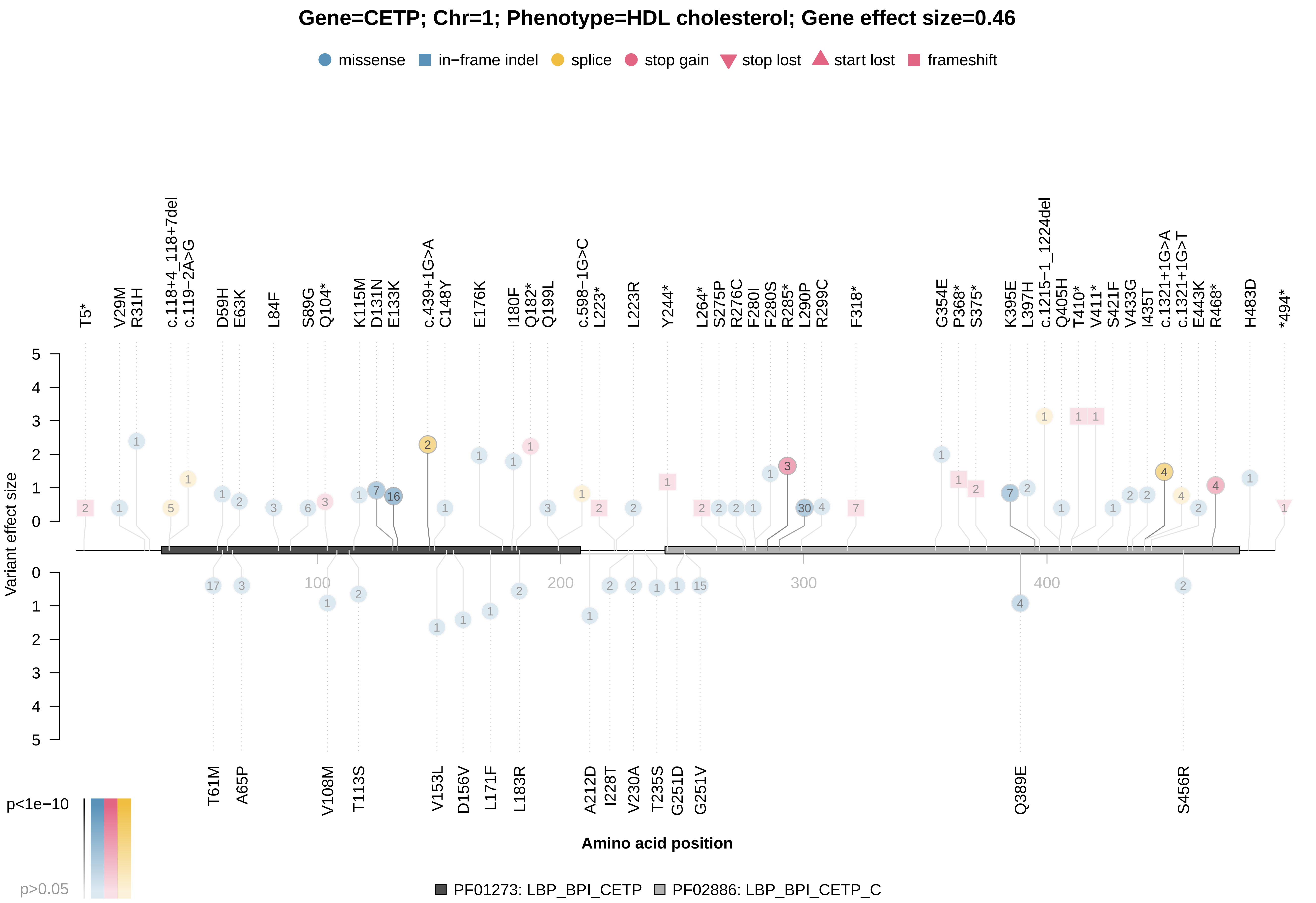

Gene=CETP; Chr=1; Phenotype=HDL cholesterol; Gene effect size=0.46
missense
in−frame indel
splice
stop gain
stop lost
start lost
frameshift
T5*
V29M
R31H
c.118+4_118+7del
c.119−2A>G
D59H
E63K
L84F
S89G
Q104*
K115M
D131N
E133K
c.439+1G>A
C148Y
E176K
I180F
Q182*
Q199L
c.598−1G>C
L223*
L223R
Y244*
L264*
S275P
R276C
F280I
F280S
R285*
L290P
R299C
F318*
G354E
P368*
S375*
K395E
L397H
c.1215−1_1224del
Q405H
T410*
V411*
S421F
V433G
I435T
c.1321+1G>A
c.1321+1G>T
E443K
R468*
H483D
494
Variant effect size
T61M
A65P
V108M
T113S
V153L
D156V
L171F
L183R
A212D
I228T
V230A
T235S
G251D
G251V
Q389E
S456R
Amino acid position
p<1e−10
p>0.05
PF01273: LBP_BPI_CETP
PF02886: LBP_BPI_CETP_C

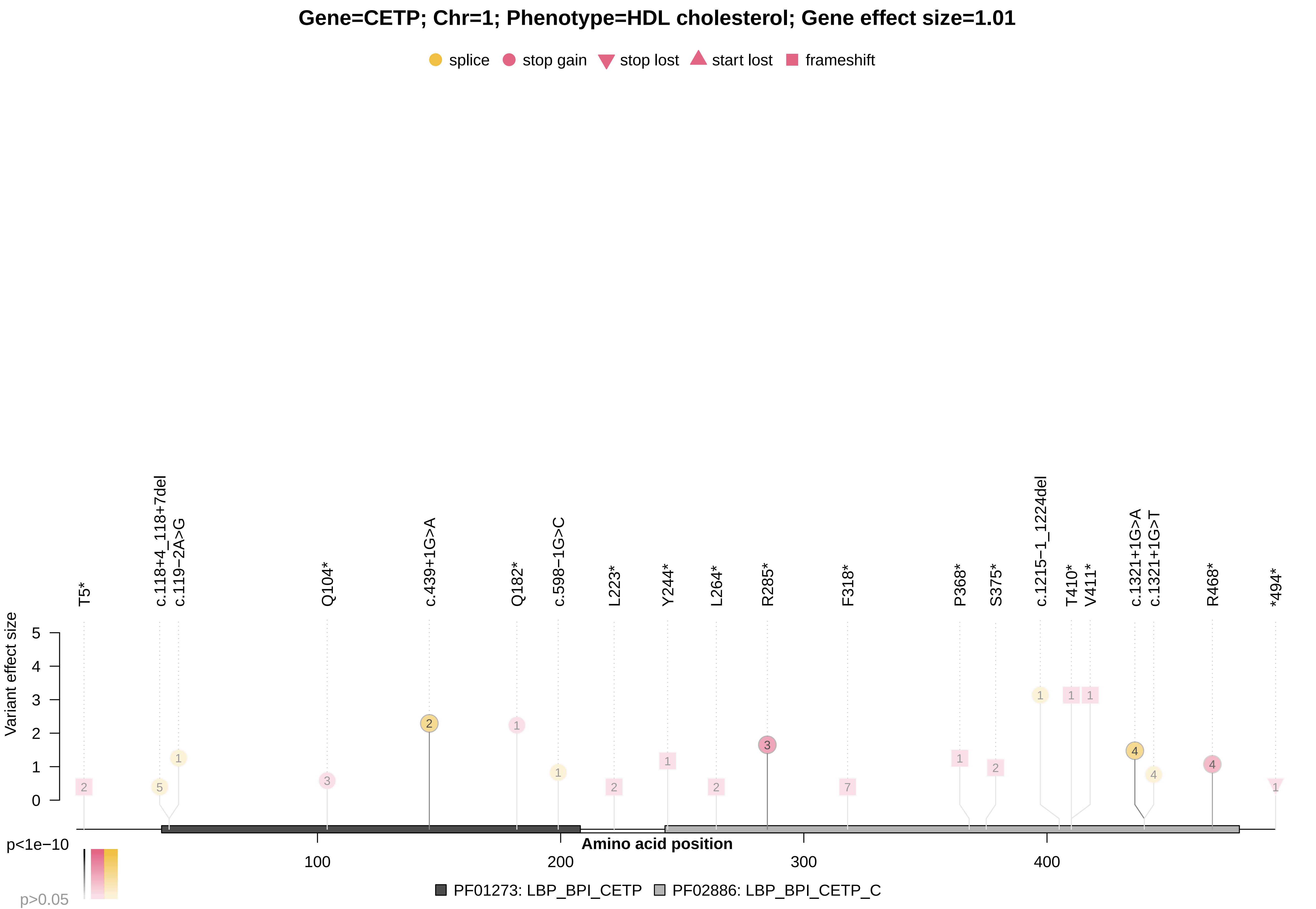
Gene=CETP; Chr=1; Phenotype=HDL cholesterol; Gene effect size=1.01
splice
stop gain
stop lost
start lost
frameshift
T5*
c.118+4_118+7del
c.119−2A>G
Q104*
c.439+1G>A
Q182*
c.598−1G>C
L223*
Y244*
L264*
R285*
F318*
P368*
S375*
c.1215−1_1224del
T410*
V411*
c.1321+1G>A
c.1321+1G>T
R468*
494
Variant effect size
0
1
2
3
4
5
Amino acid position
100
200
300
400
p<1e−10
p>0.05
PF01273: LBP_BPI_CETP
PF02886: LBP_BPI_CETP_C

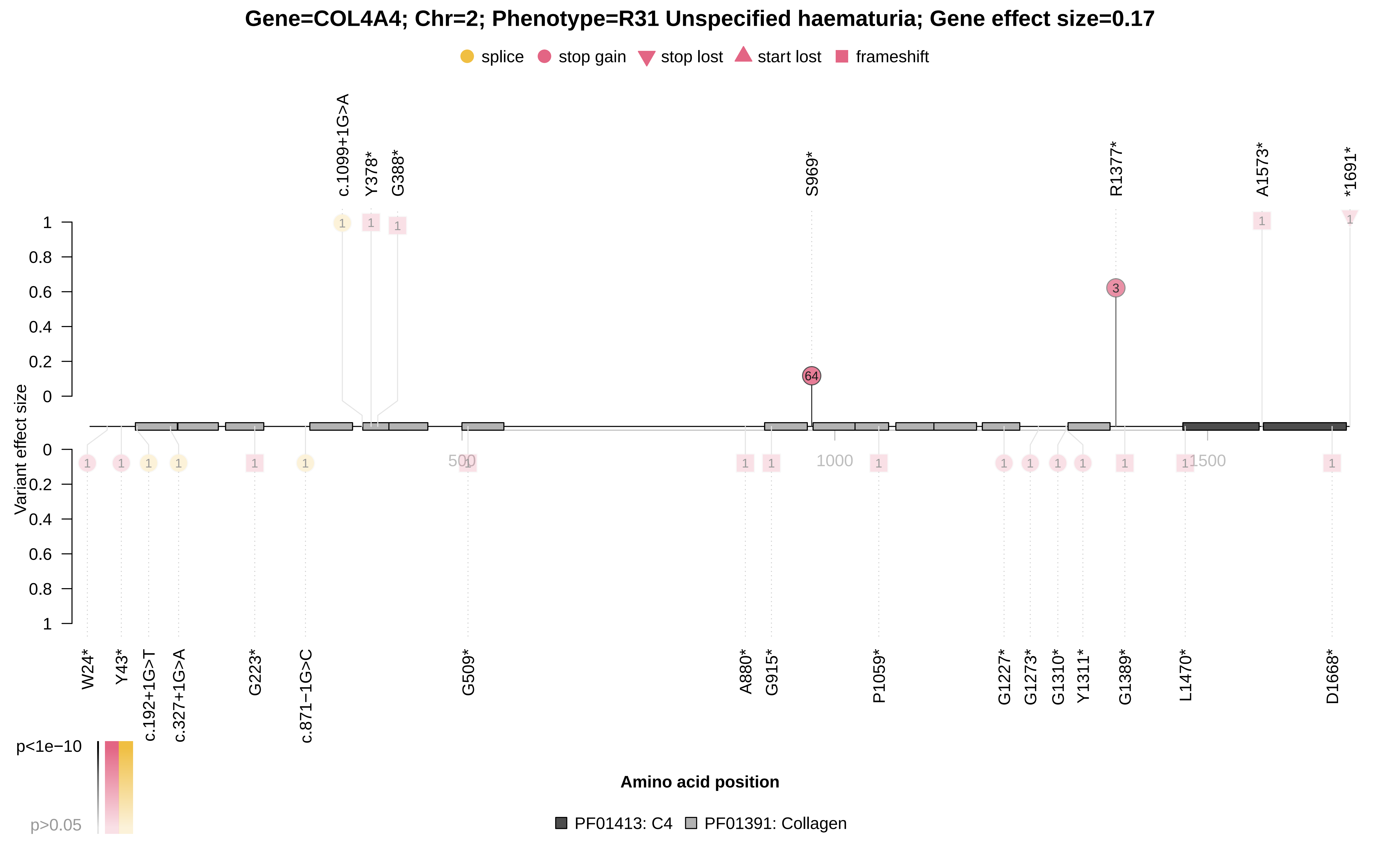

Gene=COL4A4; Chr=2; Phenotype=R31 Unspecified haematuria; Gene effect size=0.17
splice
stop gain
stop lost
start lost
frameshift
c.1099+1G>A
Y378*
G388*
S969*
R1377*
A1573*
1691
Variant effect size
Amino acid position
W24*
Y43*
c.192+1G>T
c.327+1G>A
G223*
c.871−1G>C
G509*
A880*
G915*
P1059*
G1227*
G1273*
G1310*
Y1311*
G1389*
L1470*
D1668*
500
1000
1500
p<1e−10
p>0.05
PF01413: C4
PF01391: Collagen

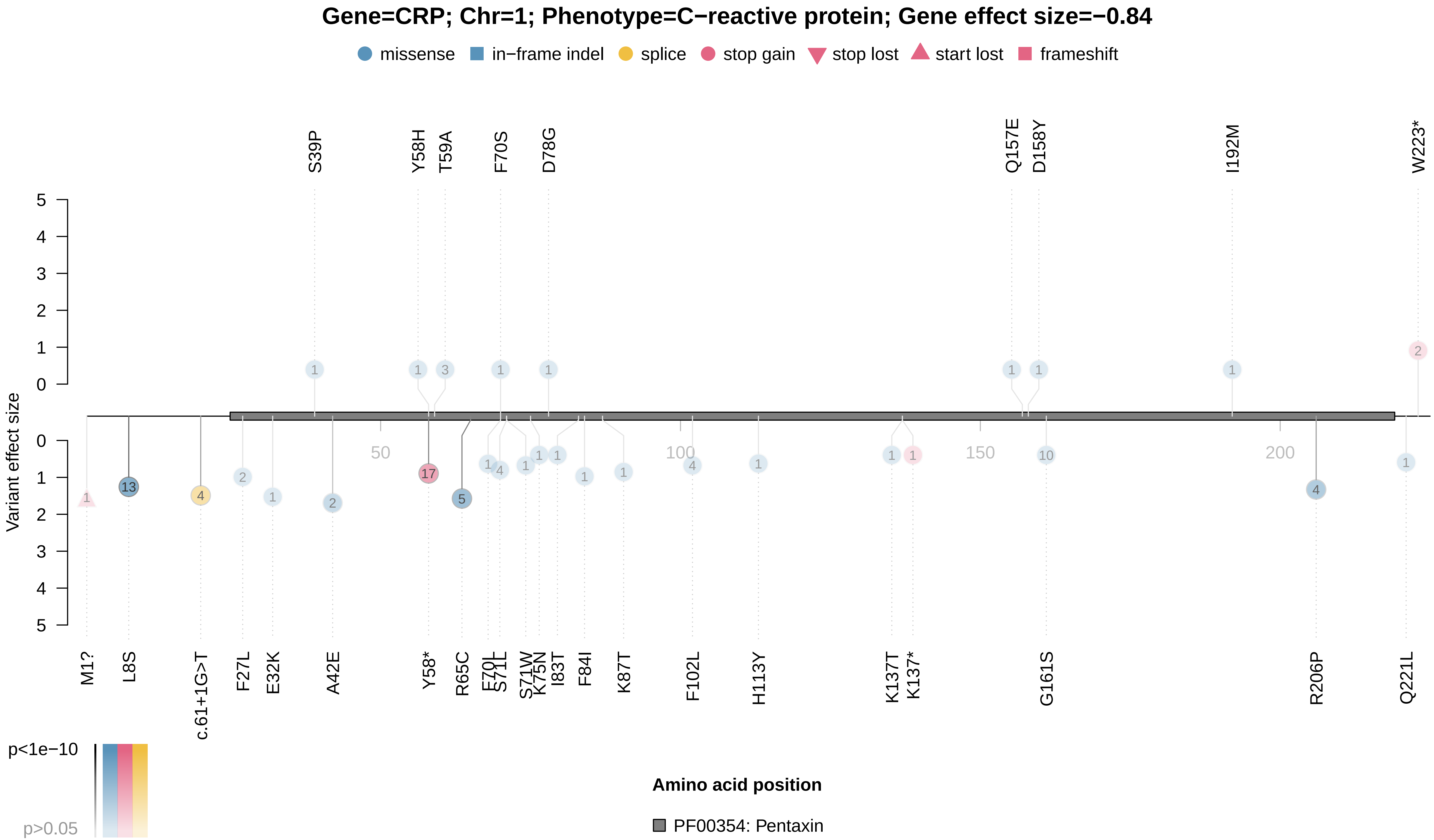

Gene=CRP; Chr=1; Phenotype=C−reactive protein; Gene effect size=−0.84
missense
in−frame indel
splice
stop gain
stop lost
start lost
frameshift
S39P
Y58H
T59A
F70S
D78G
Q157E
D158Y
I192M
W223*
Variant effect size
M1?
L8S
c.61+1G>T
F27L
E32K
A42E
Y58*
R65C
F70L
S71L
S71W
K75N
I83T
F84I
K87T
F102L
H113Y
K137T
K137*
G161S
R206P
Q221L
p<1e−10
p>0.05
Amino acid position
PF00354: Pentaxin

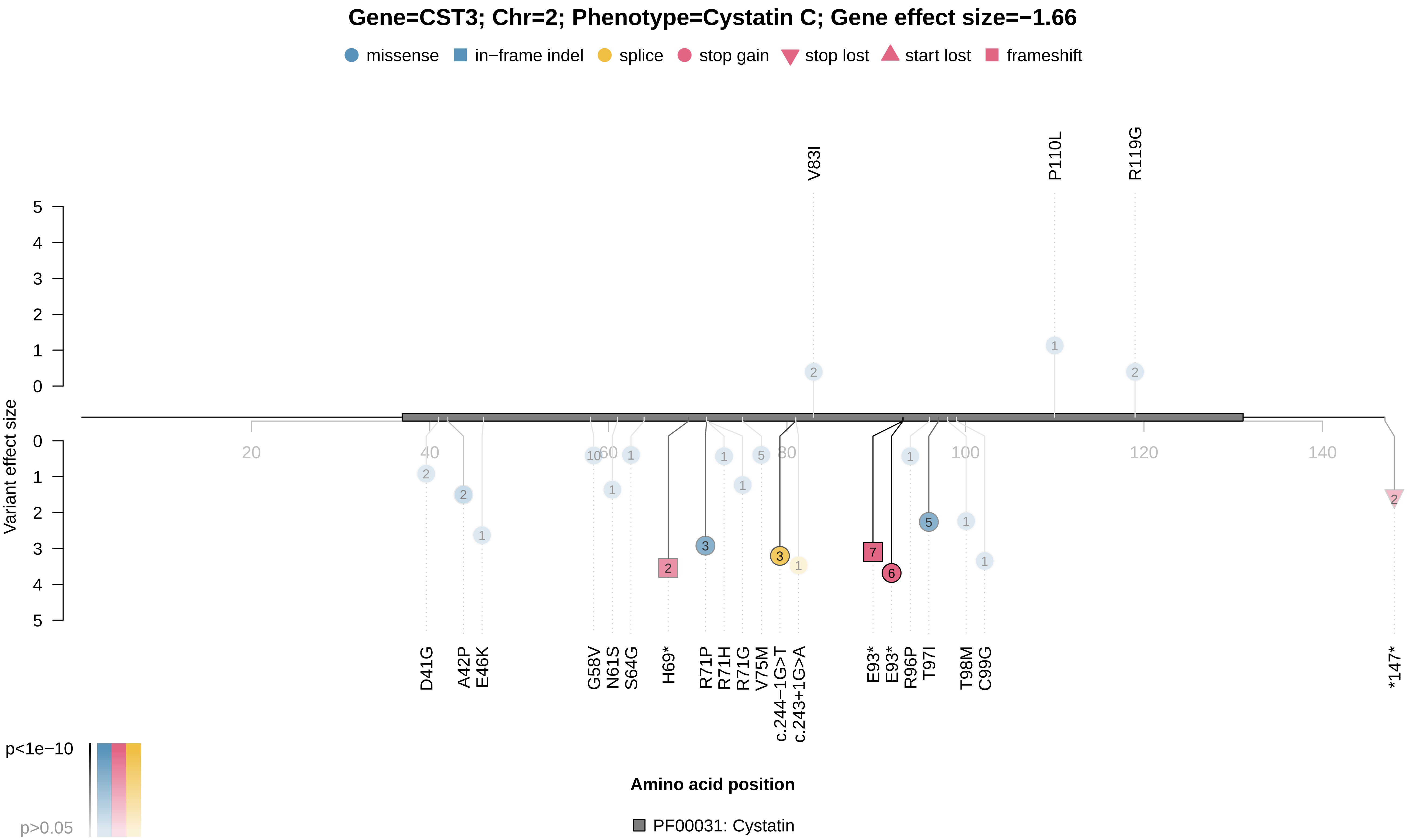

Gene=CST3; Chr=2; Phenotype=Cystatin C; Gene effect size=−1.66
missense
in−frame indel
splice
stop gain
stop lost
start lost
frameshift
Variant effect size
V83I
P110L
R119G
D41G
A42P
E46K
G58V
N61S
S64G
H69*
R71P
R71H
R71G
V75M
c.244−1G>T
c.243+1G>A
E93*
E93*
R96P
T97I
T98M
C99G
147
p<1e−10
p>0.05
Amino acid position
PF00031: Cystatin

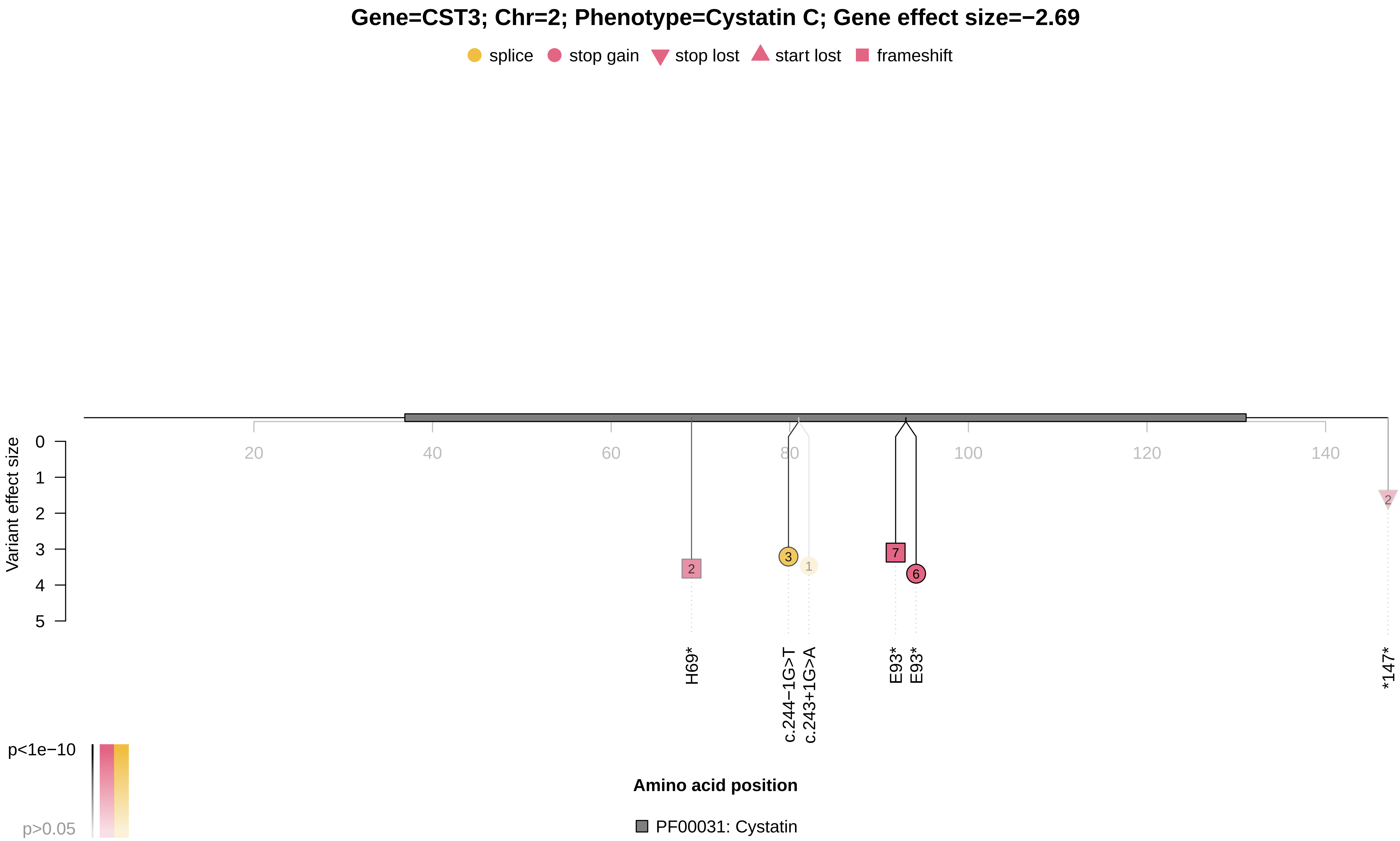

Gene=CST3; Chr=2; Phenotype=Cystatin C; Gene effect size=−2.69
splice
stop gain
stop lost
start lost
frameshift
Variant effect size
0
1
2
3
4
5
20
40
60
80
100
120
140
2
3
1
7
6
2
H69*
c.244−1G>T
c.243+1G>A
E93*
E93*
147
p<1e−10
p>0.05
Amino acid position
PF00031: Cystatin

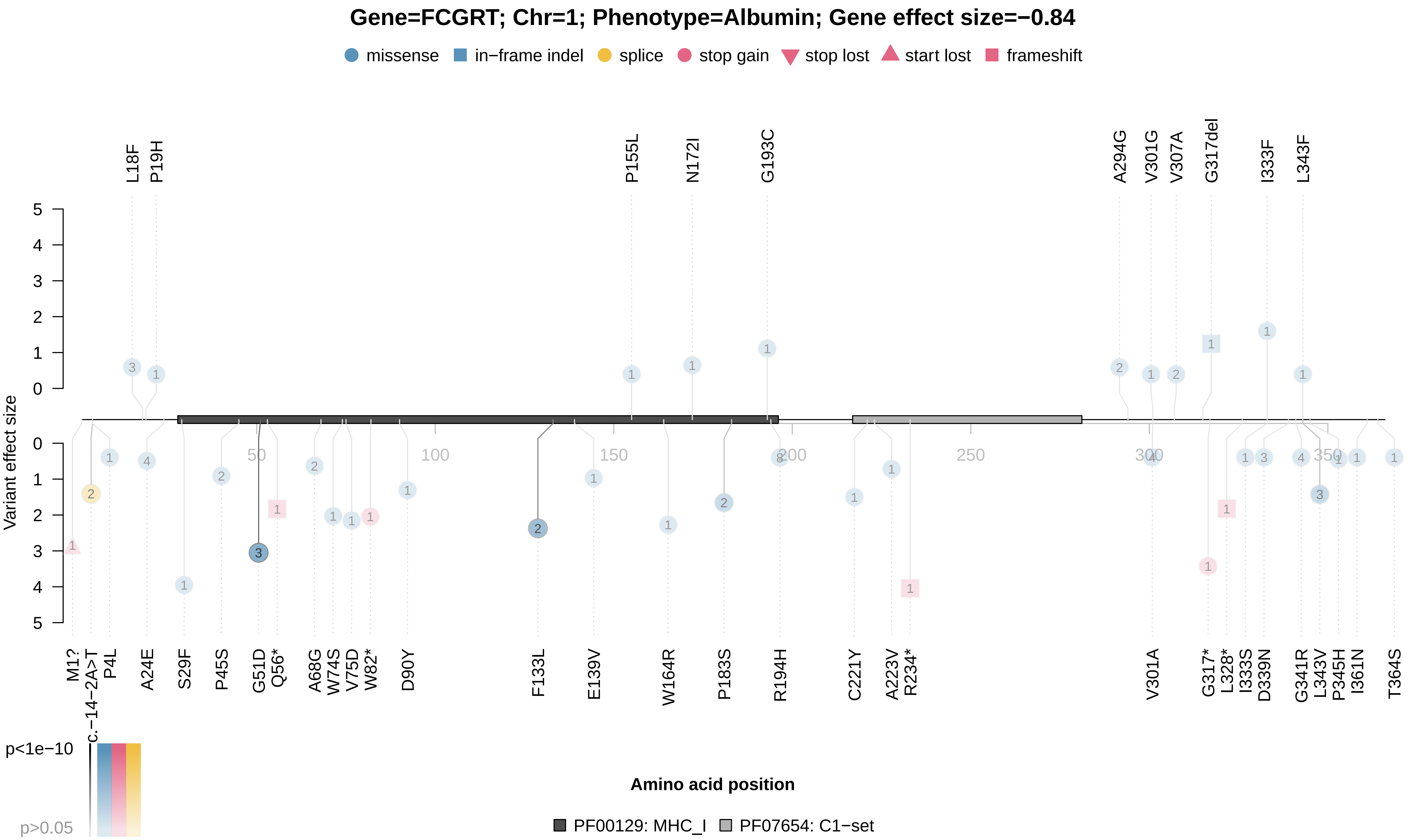

Gene=FCGRT; Chr=1; Phenotype=Albumin; Gene effect size=−0.84
missense
in−frame indel
splice
stop gain
stop lost
start lost
frameshift
L18F
P19H
P155L
N172I
G193C
A294G
V301G
V307A
G317del
I333F
L343F
Variant effect size
M1?
c.−14−2A>T
P4L
A24E
S29F
P45S
G51D
Q56*
A68G
W74S
V75D
W82*
D90Y
F133L
E139V
W164R
P183S
R194H
C221Y
A223V
R234*
V301A
G317*
L328*
I333S
D339N
G341R
L343V
P345H
I361N
T364S
p<1e−10
p>0.05
Amino acid position
PF00129: MHC_I
PF07654: C1−set

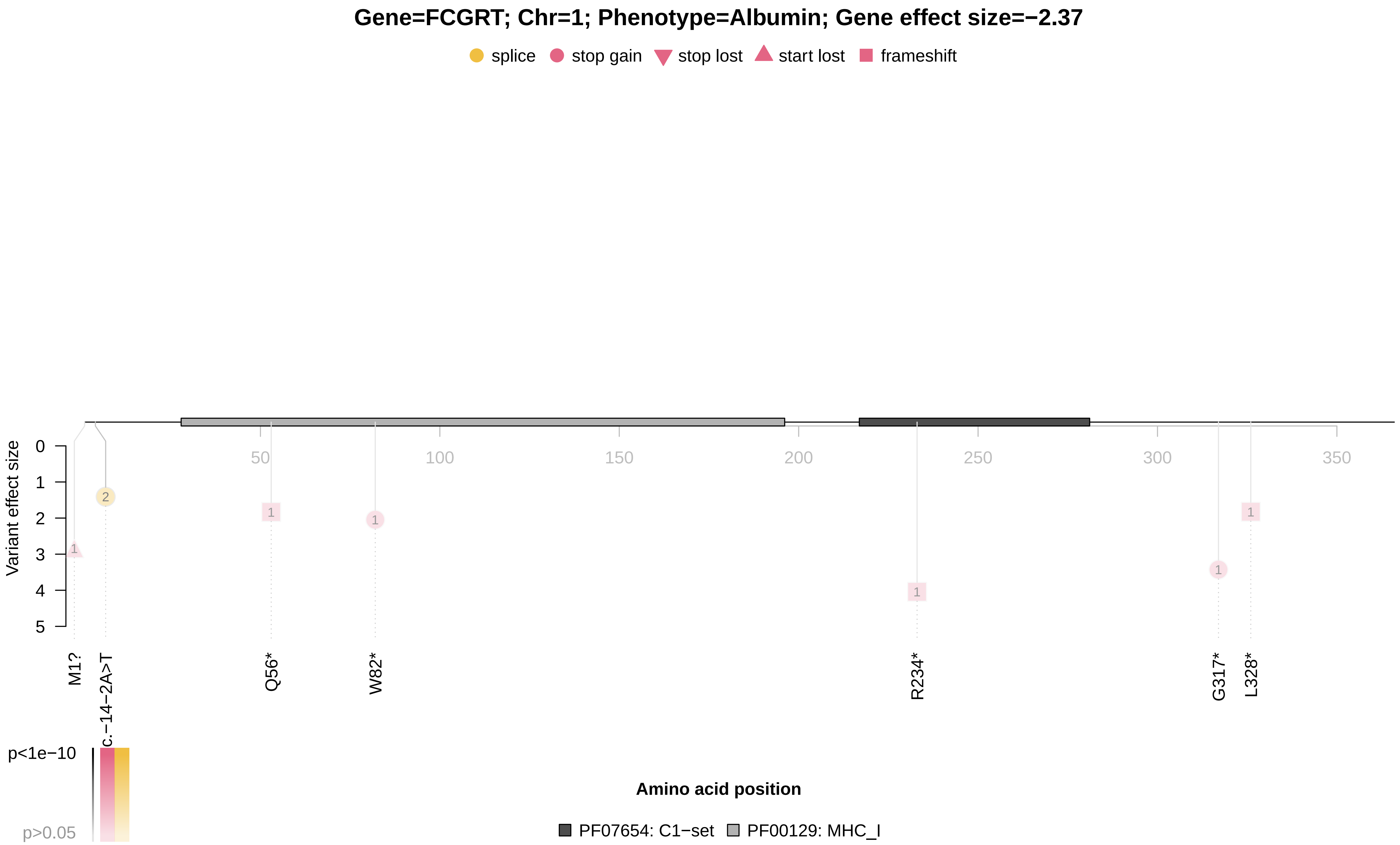

Gene=FCGRT; Chr=1; Phenotype=Albumin; Gene effect size=−2.37
splice
stop gain
stop lost
start lost
frameshift
Variant effect size
0
1
2
3
4
5
50
100
150
200
250
300
350
M1?
c.−14−2A>T
Q56*
W82*
R234*
G317*
L328*
p<1e−10
p>0.05
Amino acid position
PF07654: C1−set
PF00129: MHC_I

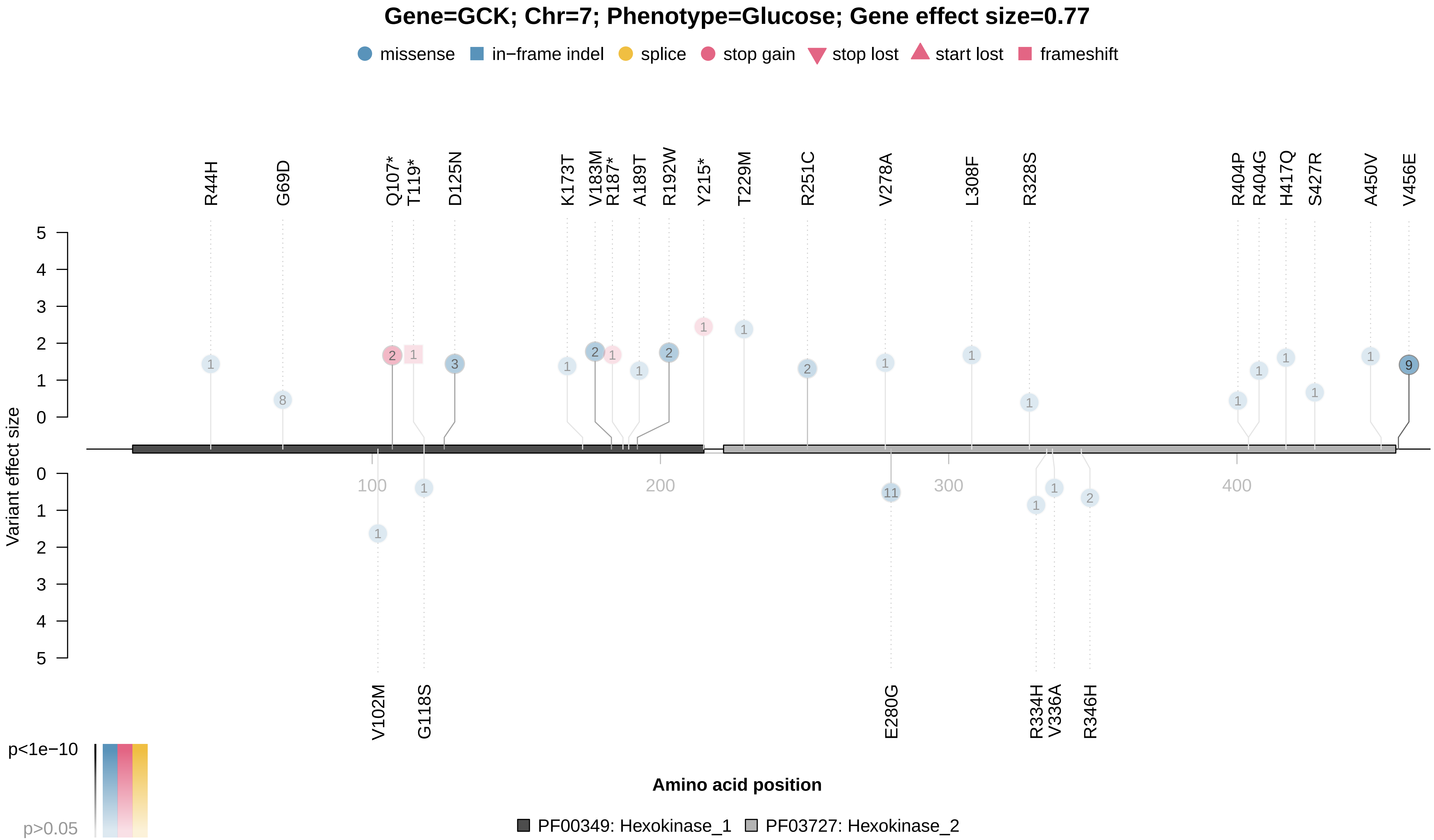

Gene=GCK; Chr=7; Phenotype=Glucose; Gene effect size=0.77
missense
in−frame indel
splice
stop gain
stop lost
start lost
frameshift
R44H
G69D
Q107*
T119*
D125N
K173T
V183M
R187*
A189T
R192W
Y215*
T229M
R251C
V278A
L308F
R328S
R404P
R404G
H417Q
S427R
A450V
V456E
V102M
G118S
E280G
R334H
V336A
R346H
Variant effect size
100
200
300
400
Amino acid position
p<1e−10
p>0.05
PF00349: Hexokinase_1
PF03727: Hexokinase_2

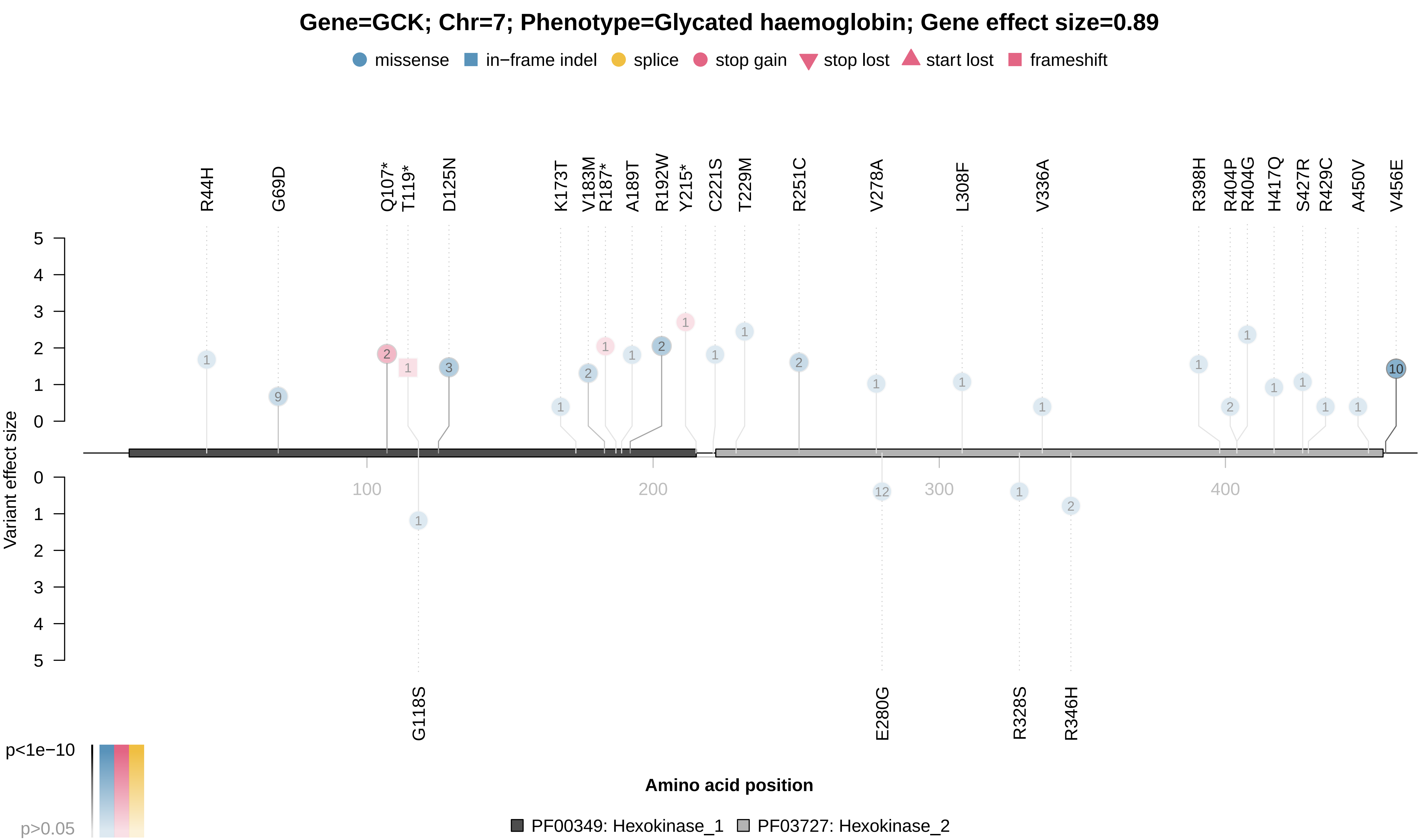

Gene=GCK; Chr=7; Phenotype=Glycated haemoglobin; Gene effect size=0.89
missense
in−frame indel
splice
stop gain
stop lost
start lost
frameshift
R44H
G69D
Q107*
T119*
D125N
K173T
V183M
R187*
A189T
R192W
Y215*
C221S
T229M
R251C
V278A
L308F
V336A
R398H
R404P
R404G
H417Q
S427R
R429C
A450V
V456E
G118S
E280G
R328S
R346H
Variant effect size
100
200
300
400
Amino acid position
p<1e−10
p>0.05
PF00349: Hexokinase_1
PF03727: Hexokinase_2

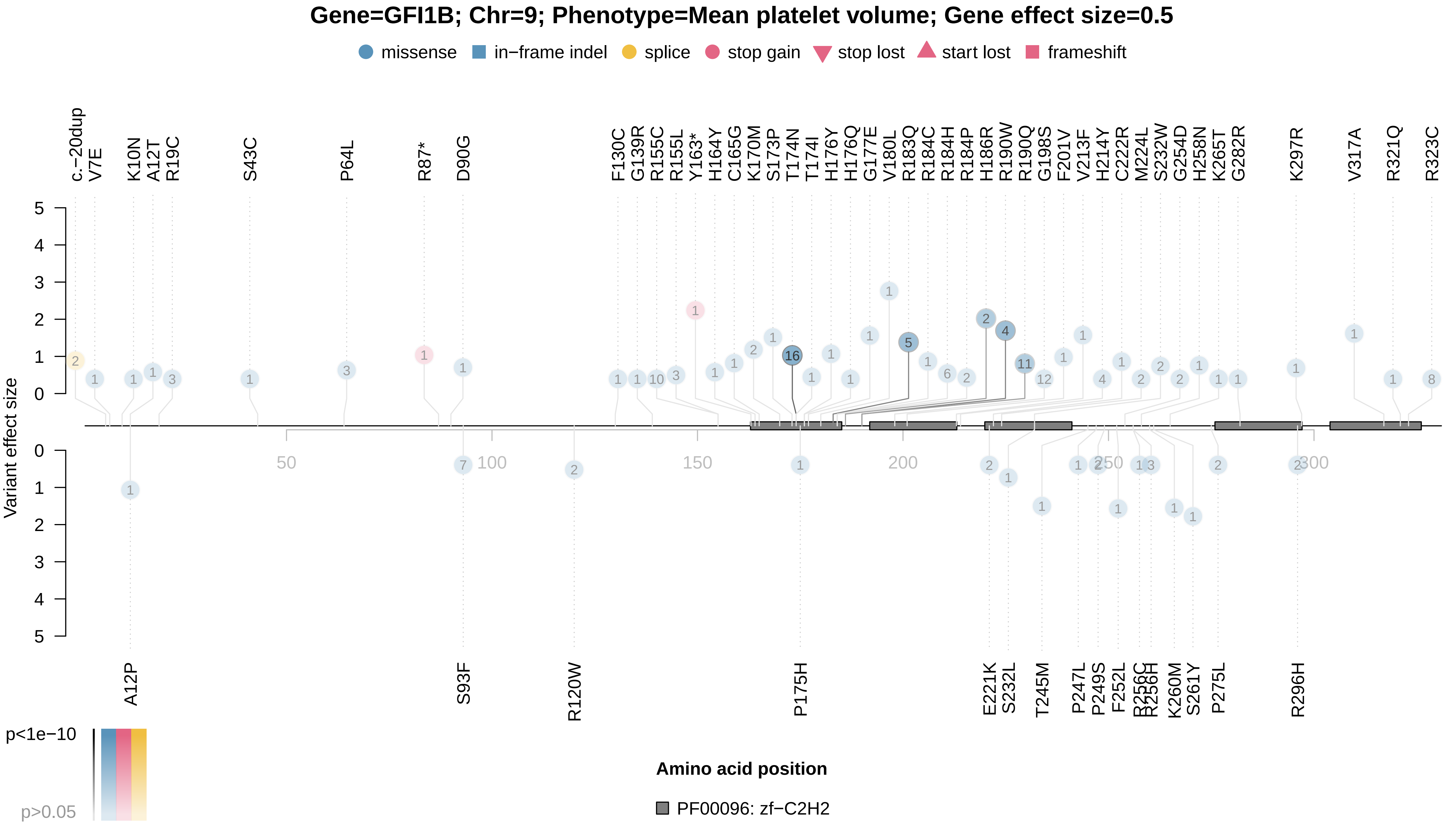

Gene=GFI1B; Chr=9; Phenotype=Mean platelet volume; Gene effect size=0.5
missense
in−frame indel
splice
stop gain
stop lost
start lost
frameshift
Variant effect size
Amino acid position
PF00096: zf−C2H2
p<1e−10
p>0.05
c.−20dup
V7E
K10N
A12T
R19C
S43C
P64L
R87*
D90G
F130C
G139R
R155C
R155L
Y163*
H164Y
C165G
K170M
S173P
T174N
T174I
H176Y
H176Q
G177E
V180L
R183Q
R184C
R184H
R184P
H186R
R190W
R190Q
G198S
F201V
V213F
H214Y
C222R
M224L
S232W
G254D
H258N
K265T
G282R
K297R
V317A
R321Q
R323C
A12P
S93F
R120W
P175H
E221K
S232L
T245M
P247L
P249S
F252L
R256C
R256H
K260M
S261Y
P275L
R296H

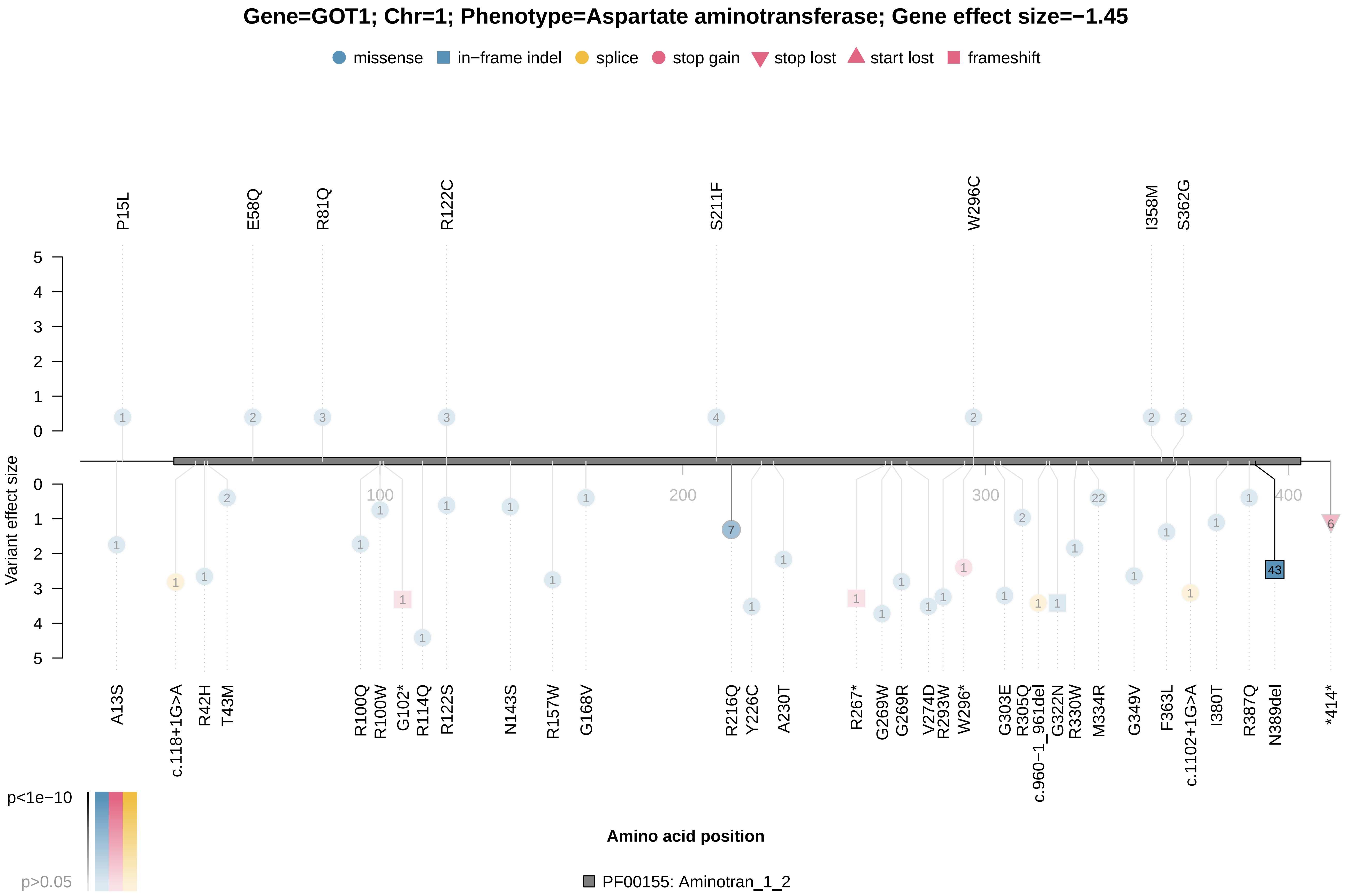
Gene=GOT1; Chr=1; Phenotype=Aspartate aminotransferase; Gene effect size=−1.45
missense
in−frame indel
splice
stop gain
stop lost
start lost
frameshift
P15L
E58Q
R81Q
R122C
S211F
W296C
I358M
S362G
Variant effect size
5
4
3
2
1
0
1
2
3
4
5
100
200
300
400
A13S
c.118+1G>A
R42H
T43M
R100Q
R100W
G102*
R114Q
R122S
N143S
R157W
G168V
R216Q
Y226C
A230T
R267*
G269W
G269R
V274D
R293W
W296*
G303E
R305Q
c.960−1_961del
G322N
R330W
M334R
G349V
F363L
c.1102+1G>A
I380T
R387Q
N389del
414
p<1e−10
p>0.05
Amino acid position
PF00155: Aminotran_1_2

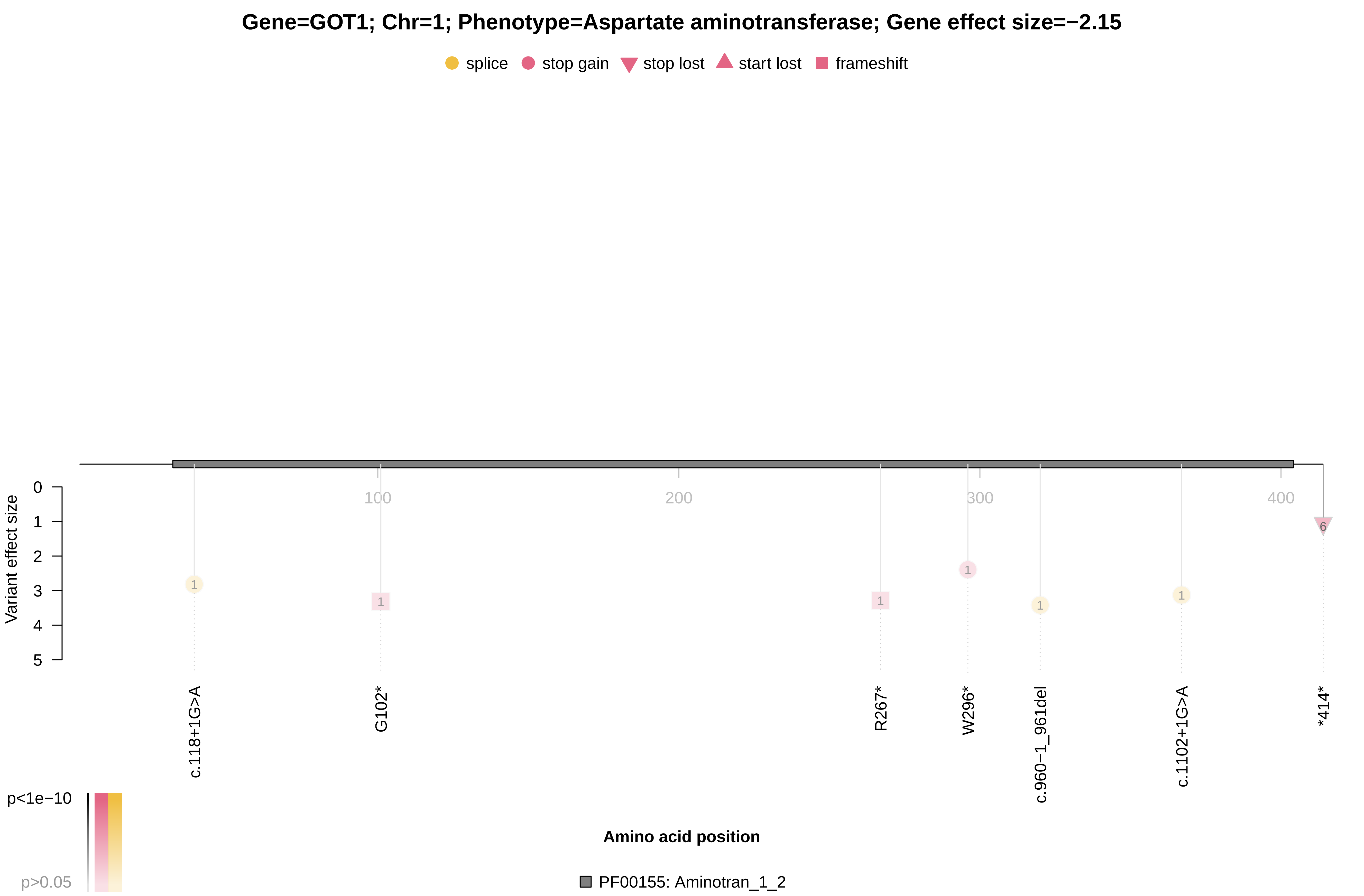
Gene=GOT1; Chr=1; Phenotype=Aspartate aminotransferase; Gene effect size=−2.15
splice
stop gain
stop lost
start lost
frameshift
Variant effect size
0
1
2
3
4
5
100
200
300
400
c.118+1G>A
G102*
R267*
W296*
c.960−1_961del
c.1102+1G>A
414
p<1e−10
p>0.05
Amino acid position
PF00155: Aminotran_1_2

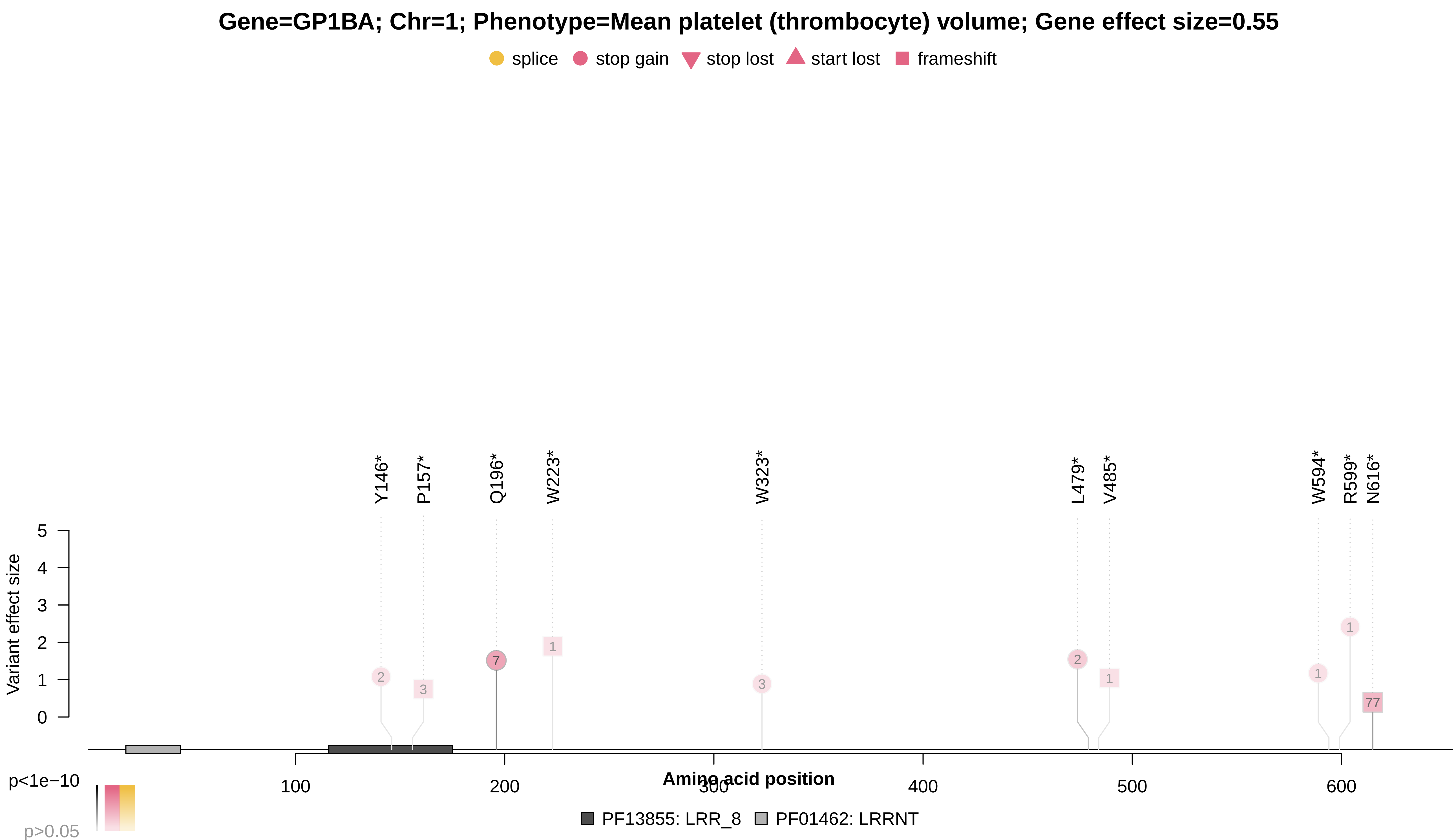

Gene=GP1BA; Chr=1; Phenotype=Mean platelet (thrombocyte) volume; Gene effect size=0.55
splice
stop gain
stop lost
start lost
frameshift
Y146*
P157*
Q196*
W223*
W323*
L479*
V485*
W594*
R599*
N616*
Variant effect size
0
1
2
3
4
5
2
3
7
1
3
2
1
1
1
77
100
200
300
400
500
600
Amino acid position
p<1e−10
p>0.05
PF13855: LRR_8
PF01462: LRRNT

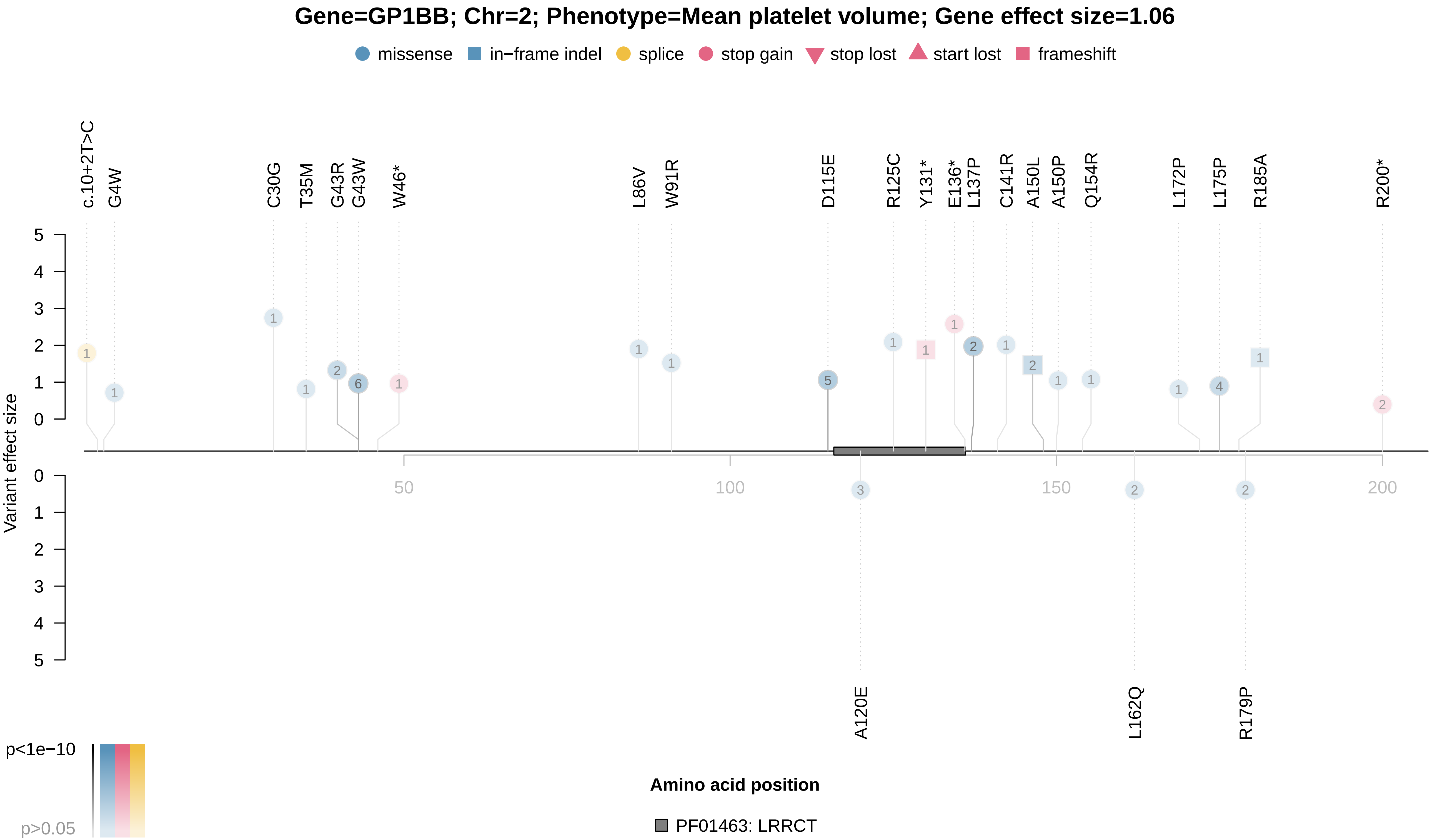

Gene=GP1BB; Chr=2; Phenotype=Mean platelet volume; Gene effect size=1.06
missense
in−frame indel
splice
stop gain
stop lost
start lost
frameshift
c.10+2T>C
G4W
C30G
T35M
G43R
G43W
W46*
L86V
W91R
D115E
R125C
Y131*
E136*
L137P
C141R
A150L
A150P
Q154R
L172P
L175P
R185A
R200*
A120E
L162Q
R179P
Variant effect size
Amino acid position
50
100
150
200
p<1e−10
p>0.05
PF01463: LRRCT

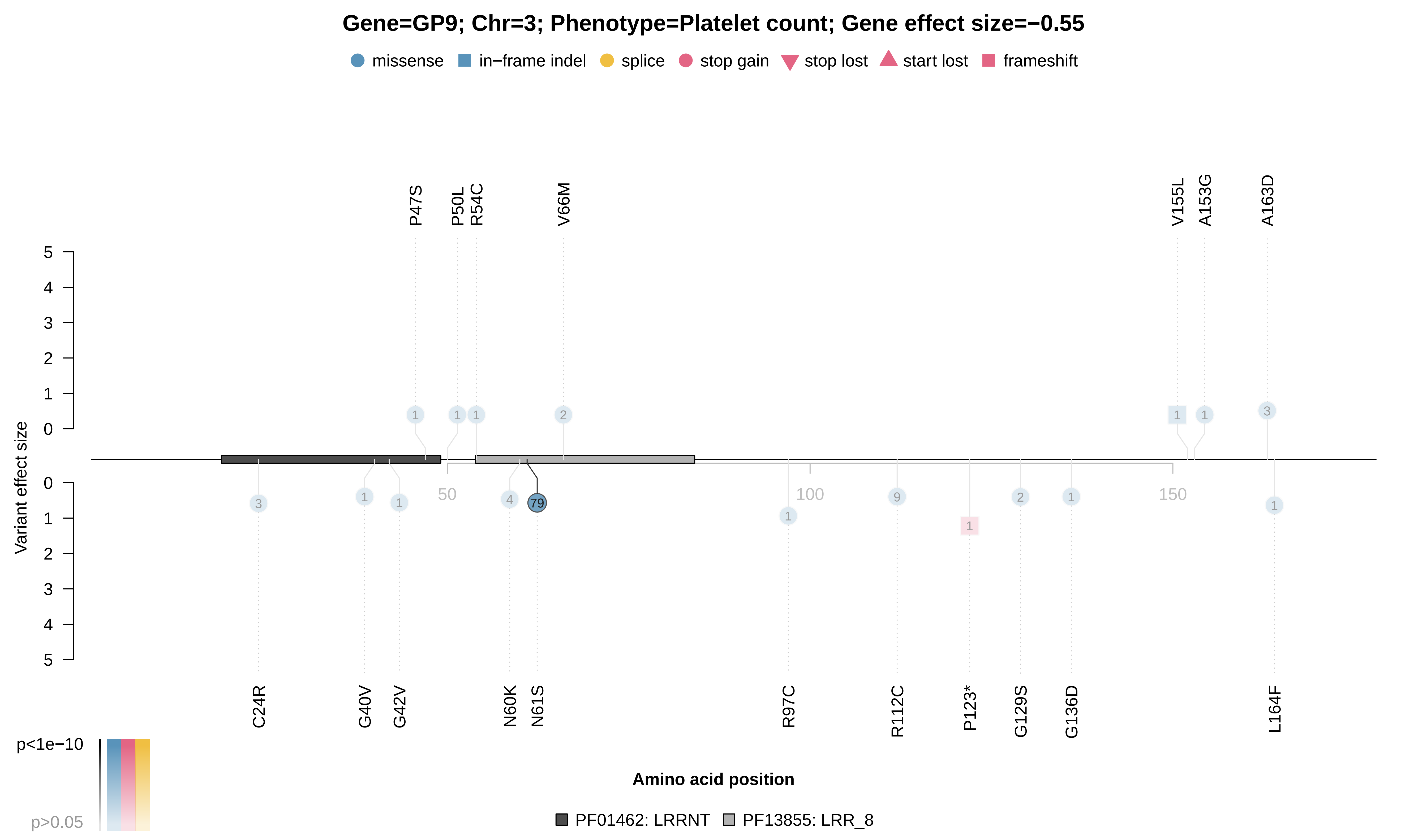

Gene=GP9; Chr=3; Phenotype=Platelet count; Gene effect size=−0.55
missense
in−frame indel
splice
stop gain
stop lost
start lost
frameshift
P47S
P50L
R54C
V66M
V155L
A153G
A163D
Variant effect size
C24R
G40V
G42V
N60K
N61S
R97C
R112C
P123*
G129S
G136D
L164F
50
100
150
p<1e−10
p>0.05
Amino acid position
PF01462: LRRNT
PF13855: LRR_8

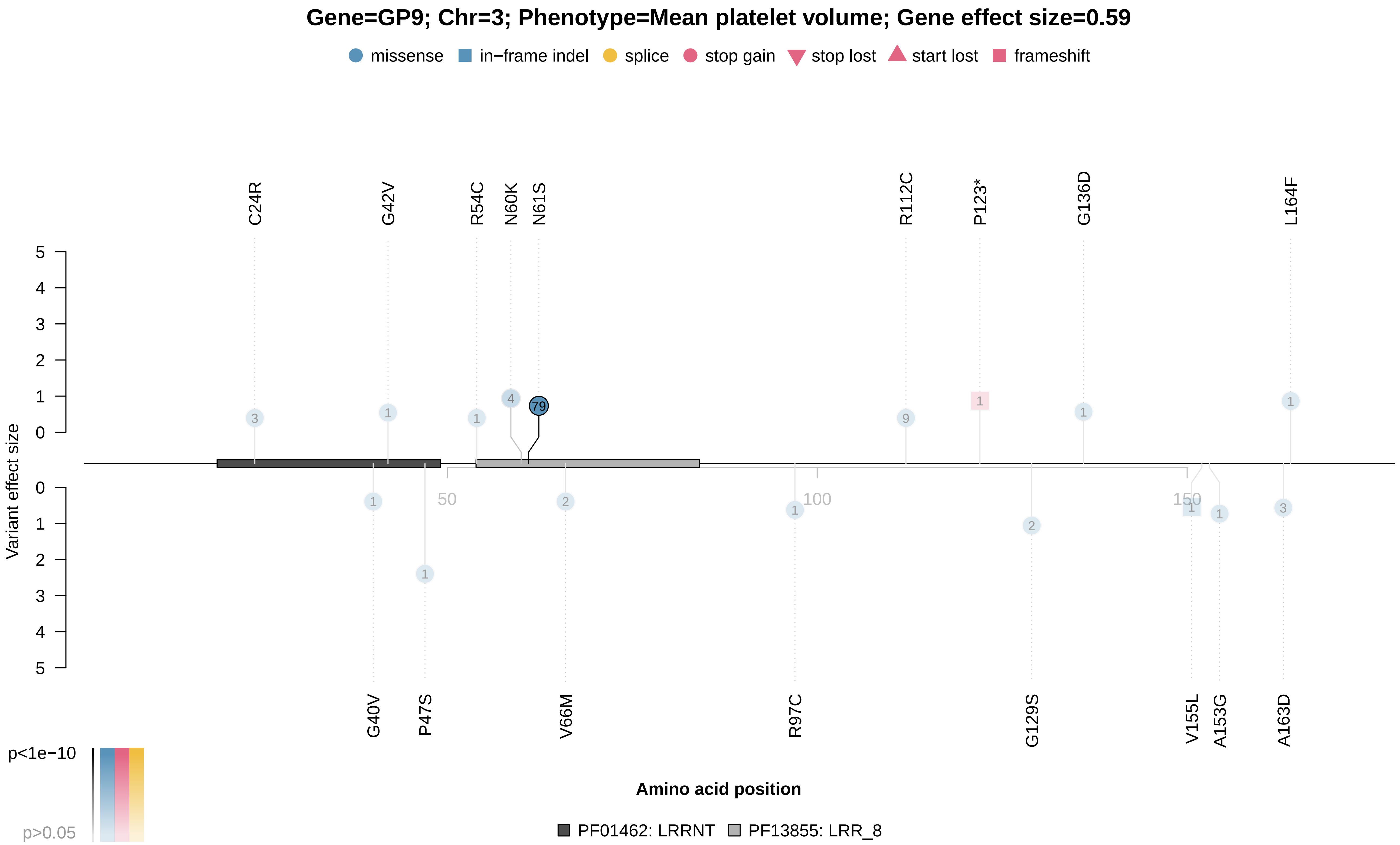

Gene=GP9; Chr=3; Phenotype=Mean platelet volume; Gene effect size=0.59
missense
in−frame indel
splice
stop gain
stop lost
start lost
frameshift
C24R
G42V
R54C
N60K
N61S
R112C
P123*
G136D
L164F
G40V
P47S
V66M
R97C
G129S
V155L
A153G
A163D
Variant effect size
50
100
150
p<1e−10
p>0.05
Amino acid position
PF01462: LRRNT
PF13855: LRR_8

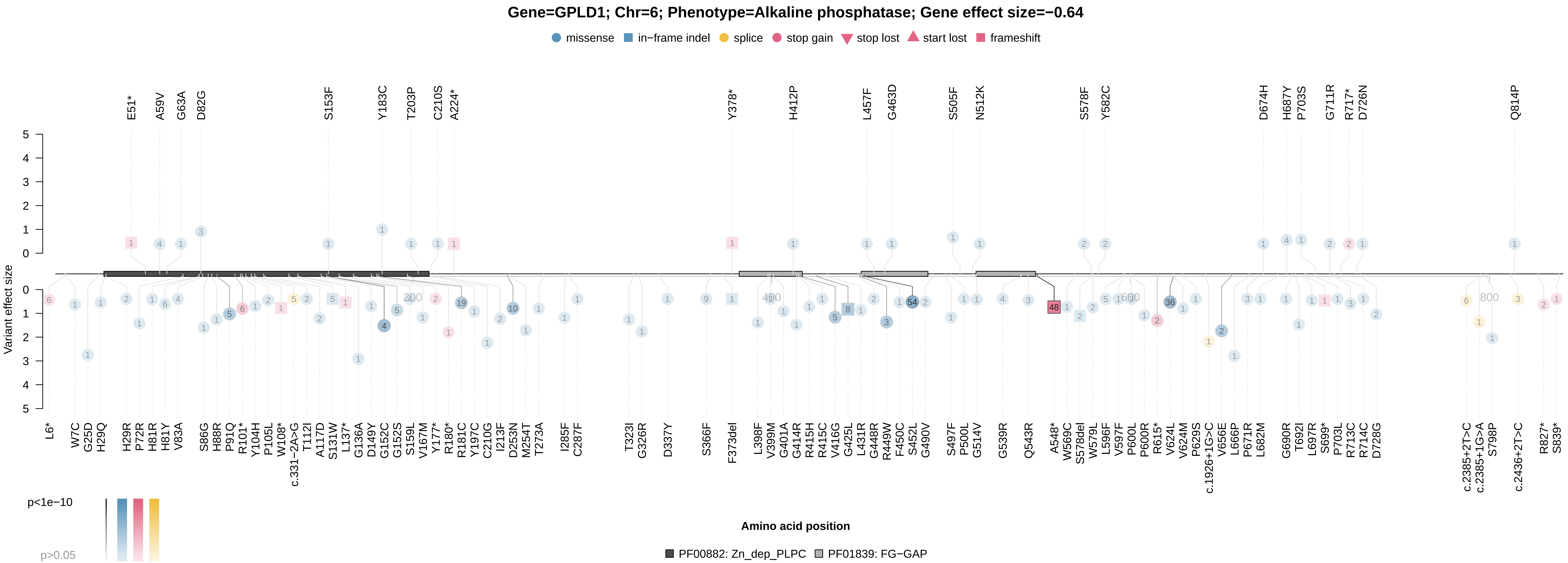
Gene=GPLD1; Chr=6; Phenotype=Alkaline phosphatase; Gene effect size=−0.64
missense
in−frame indel
splice
stop gain
stop lost
start lost
frameshift
E51*
A59V
G63A
D82G
S153F
Y183C
T203P
C210S
A224*
Y378*
H412P
L457F
G463D
S505F
N512K
S578F
Y582C
D674H
H687Y
P703S
G711R
R717*
D726N
Q814P
Variant effect size
5
4
3
2
1
0
200
400
600
800
L6*
W7C
G25D
H29Q
H29R
P72R
H81R
H81Y
V83A
S86G
H88R
P91Q
R101*
Y104H
P105L
W108*
c.331−2A>G
T112I
A117D
S131W
L137*
G136A
D149Y
G152C
G152S
S159L
V167M
Y177*
R180*
R181C
Y197C
C210G
I213F
D253N
M254T
T273A
I285F
C287F
T323I
G326R
D337Y
S366F
F373del
L398F
V399M
G401A
G414R
R415H
R415C
V416G
G425L
L431R
G448R
R449W
F450C
S452L
G490V
S497F
P500L
G514V
G539R
Q543R
A548*
W569C
S578del
W579L
L596F
V597F
P600L
P600R
R615*
V624L
V624M
P629S
c.1926+1G>C
V656E
L666P
P671R
L682M
G690R
T692I
L697R
S699*
P703L
R713C
R714C
D728G
c.2385+2T>C
c.2385+1G>A
S798P
c.2436+2T>C
R827*
S839*
p<1e−10
p>0.05
Amino acid position
PF00882: Zn_dep_PLPC
PF01839: FG−GAP

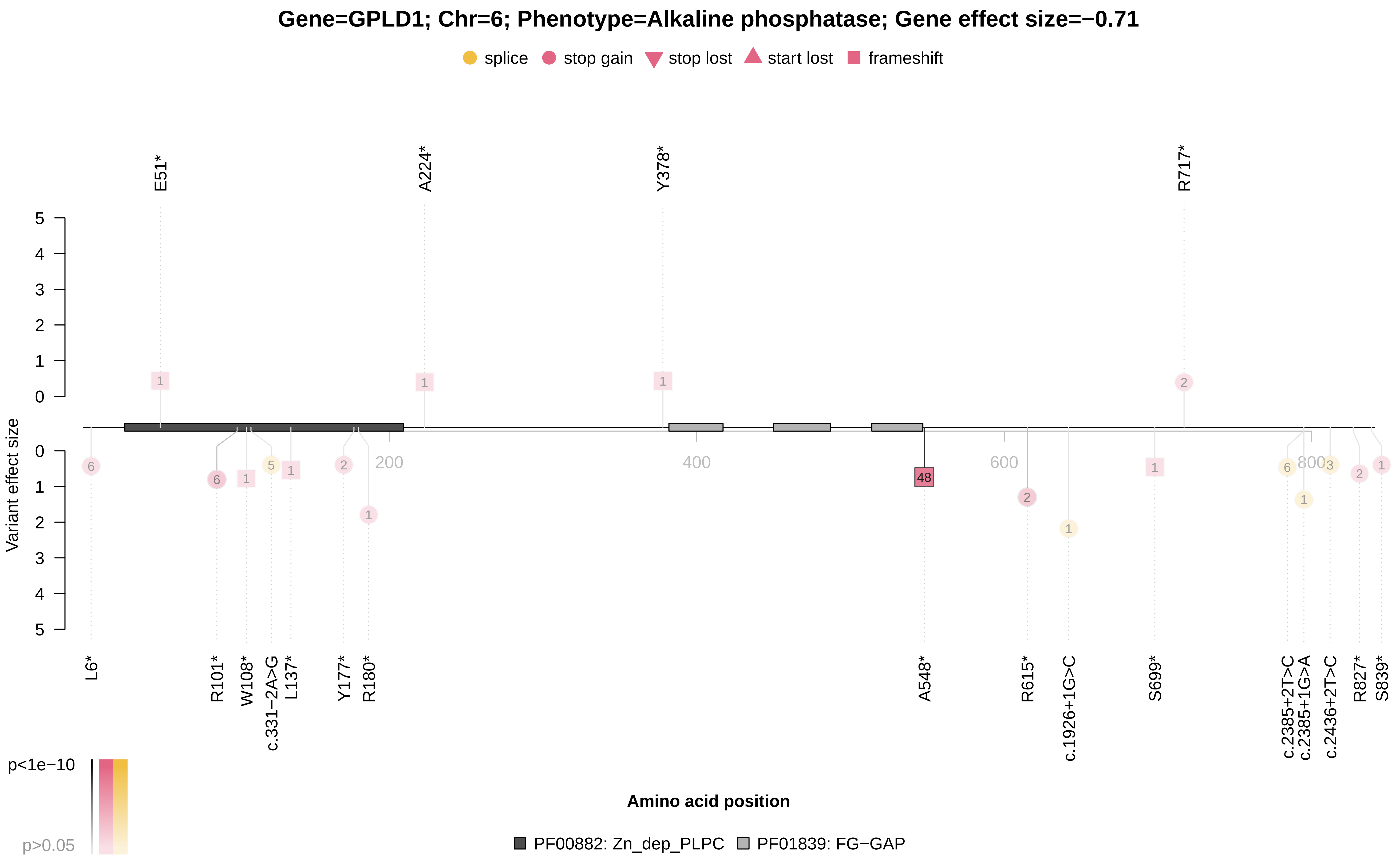

Gene=GPLD1; Chr=6; Phenotype=Alkaline phosphatase; Gene effect size=−0.71
splice
stop gain
stop lost
start lost
frameshift
E51*
A224*
Y378*
R717*
Variant effect size
200
400
600
800
L6*
R101*
W108*
c.331−2A>G
L137*
Y177*
R180*
A548*
R615*
c.1926+1G>C
S699*
c.2385+2T>C
c.2385+1G>A
c.2436+2T>C
R827*
S839*
p<1e−10
p>0.05
Amino acid position
PF00882: Zn_dep_PLPC
PF01839: FG−GAP

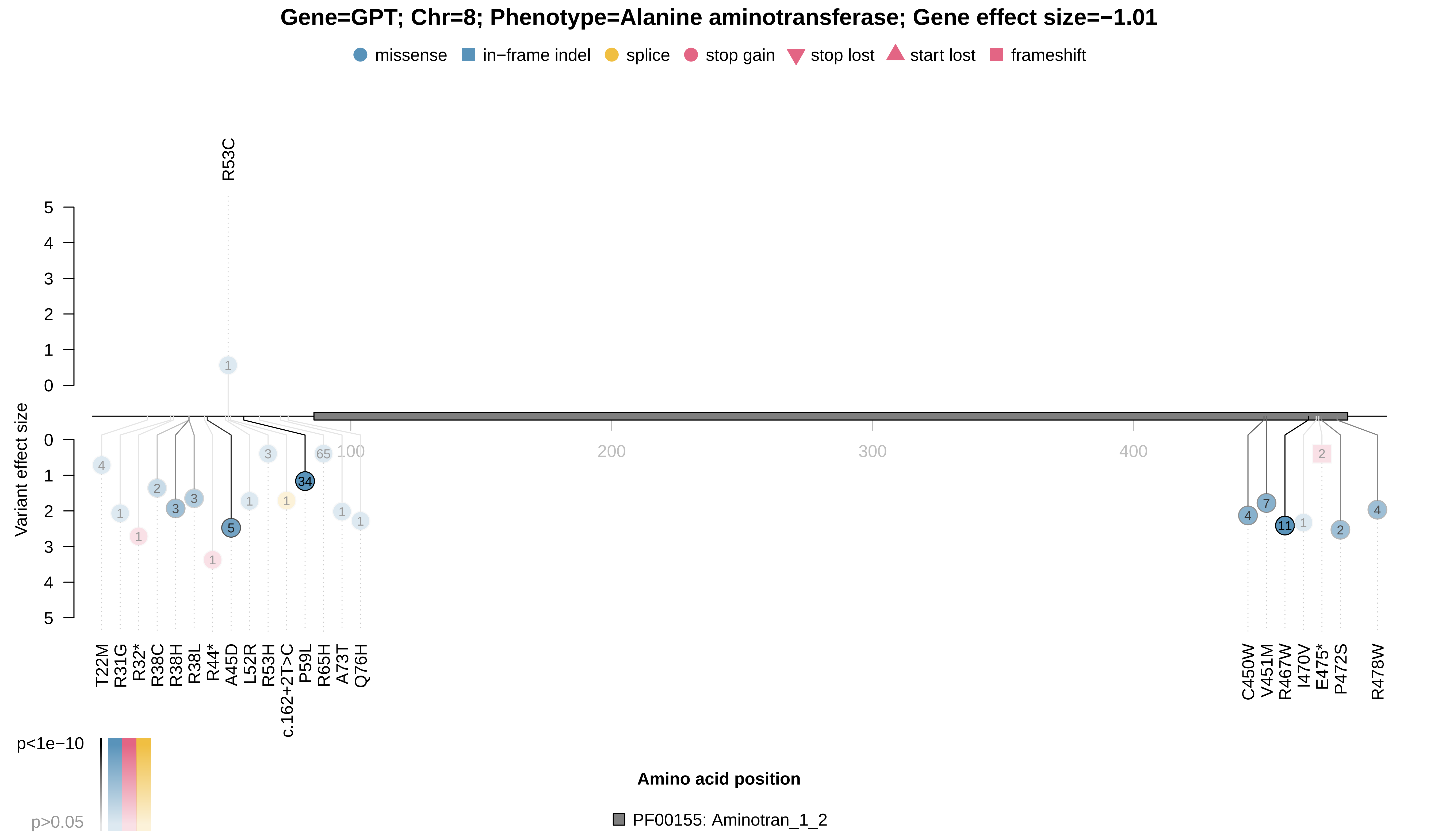

Gene=GPT; Chr=8; Phenotype=Alanine aminotransferase; Gene effect size=−1.01
missense
in−frame indel
splice
stop gain
stop lost
start lost
frameshift
R53C
Variant effect size
T22M
R31G
R32*
R38C
R38H
R38L
R44*
A45D
L52R
R53H
c.162+2T>C
P59L
R65H
A73T
Q76H
C450W
V451M
R467W
I470V
E475*
P472S
R478W
p<1e−10
p>0.05
Amino acid position
PF00155: Aminotran_1_2

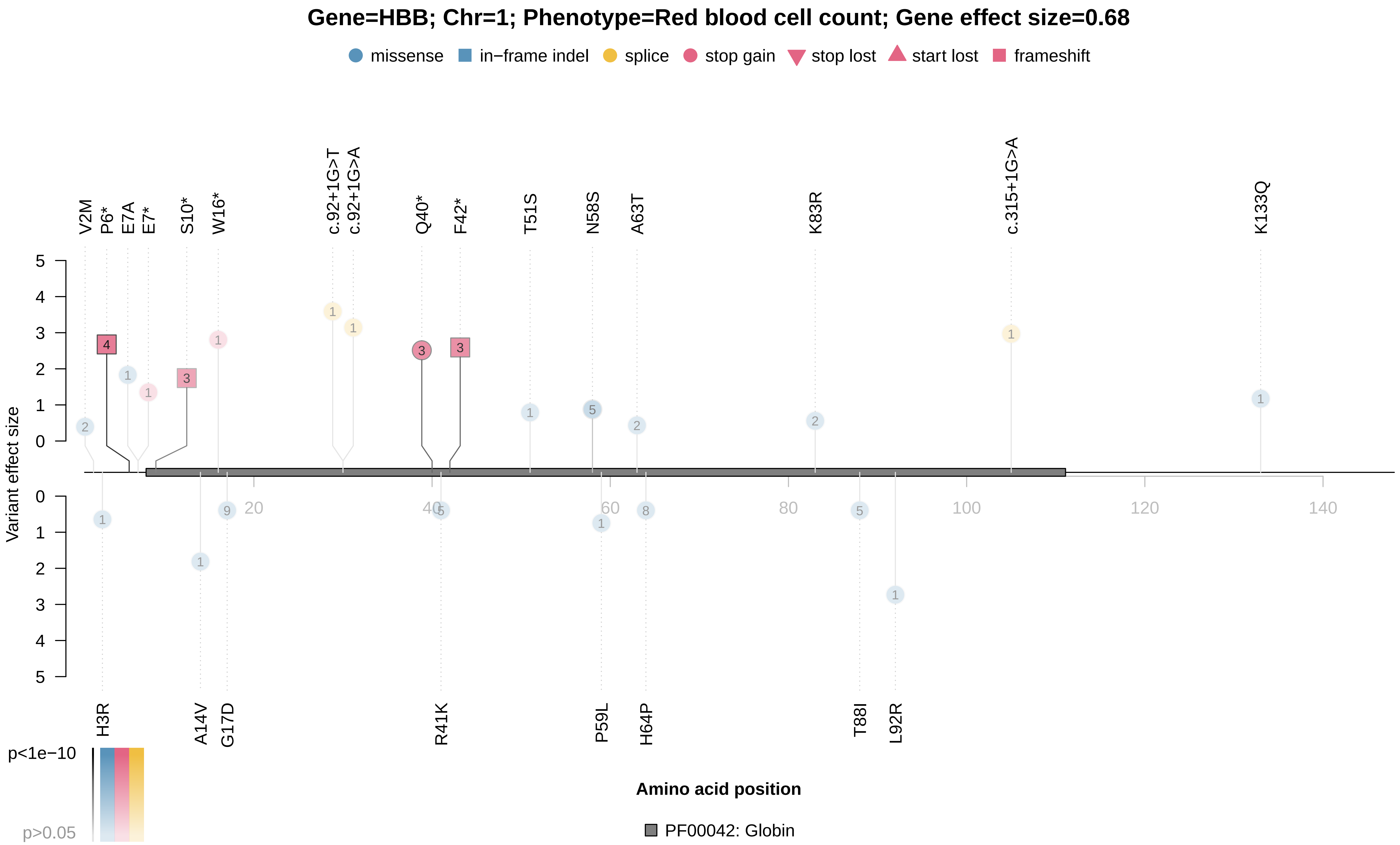

Gene=HBB; Chr=1; Phenotype=Red blood cell count; Gene effect size=0.68
missense
in−frame indel
splice
stop gain
stop lost
start lost
frameshift
V2M
P6*
E7A
E7*
S10*
W16*
c.92+1G>T
c.92+1G>A
Q40*
F42*
T51S
N58S
A63T
K83R
c.315+1G>A
K133Q
Variant effect size
20
40
60
80
100
120
140
H3R
A14V
G17D
R41K
P59L
H64P
T88I
L92R
p<1e−10
p>0.05
Amino acid position
PF00042: Globin

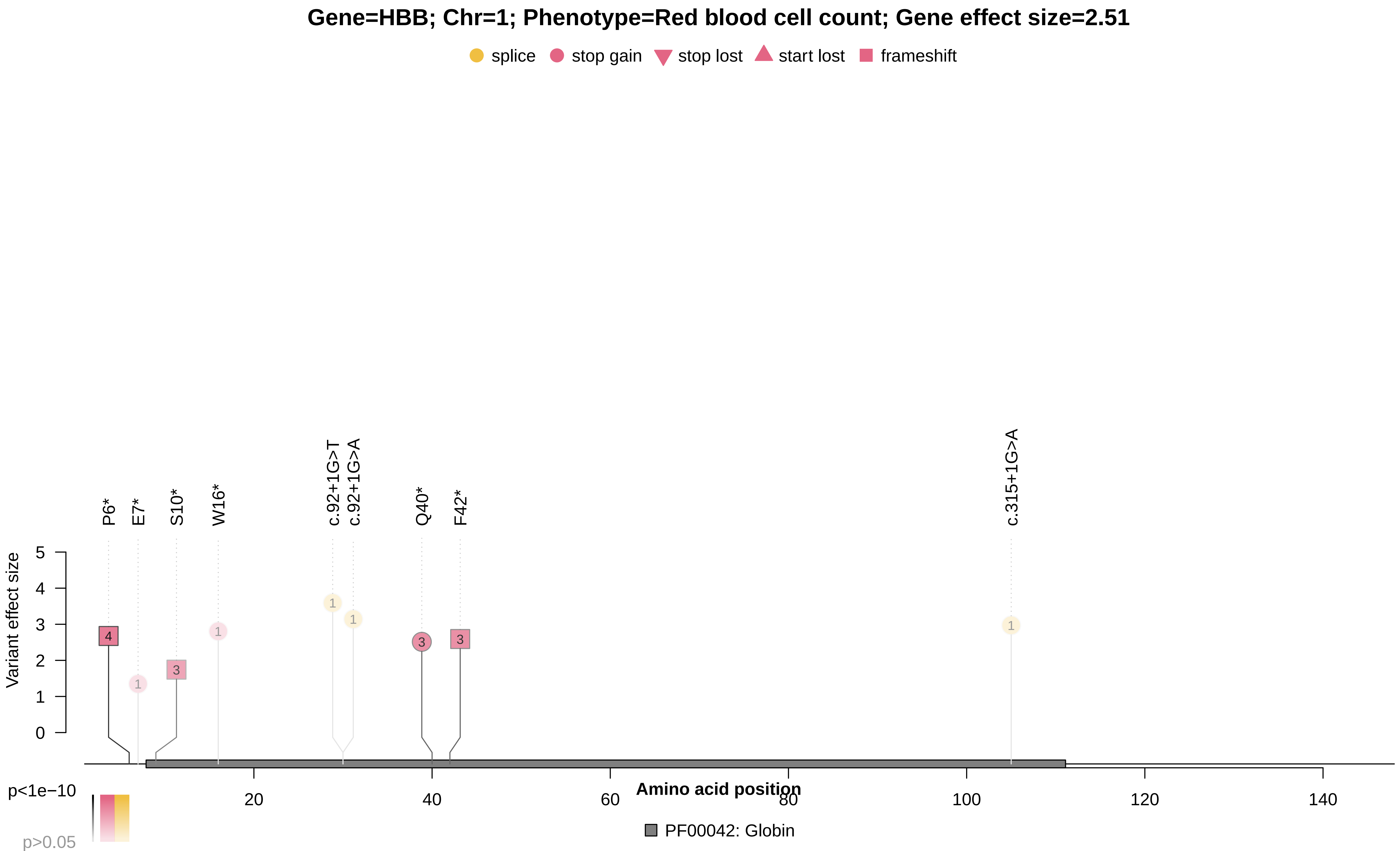

Gene=HBB; Chr=1; Phenotype=Red blood cell count; Gene effect size=2.51
splice
stop gain
stop lost
start lost
frameshift
P6*
E7*
S10*
W16*
c.92+1G>T
c.92+1G>A
Q40*
F42*
c.315+1G>A
Variant effect size
Amino acid position
p<1e−10
p>0.05
PF00042: Globin

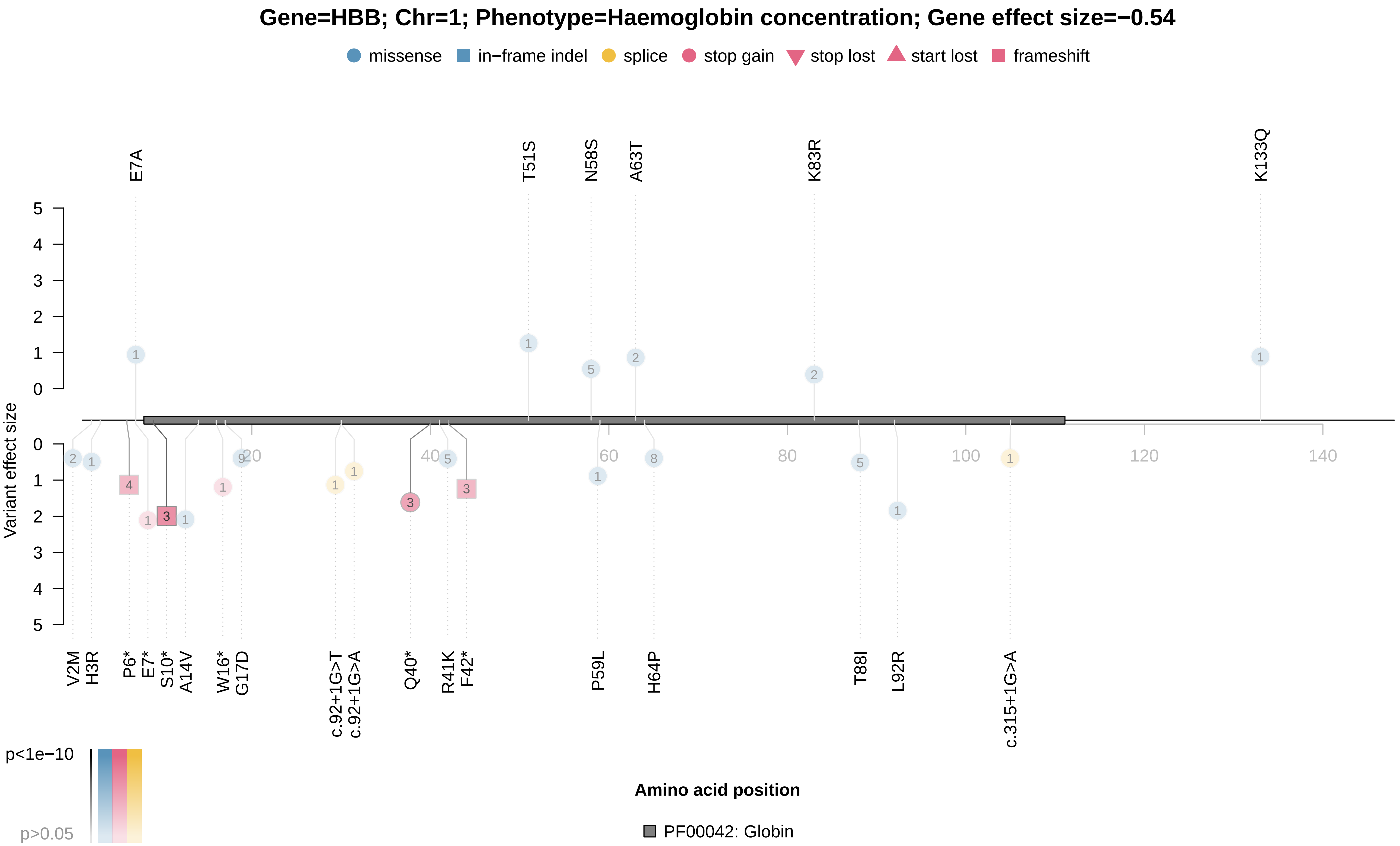

Gene=HBB; Chr=1; Phenotype=Haemoglobin concentration; Gene effect size=−0.54
missense
in−frame indel
splice
stop gain
stop lost
start lost
frameshift
E7A
T51S
N58S
A63T
K83R
K133Q
Variant effect size
Amino acid position
V2M
H3R
P6*
E7*
S10*
A14V
W16*
G17D
c.92+1G>T
c.92+1G>A
Q40*
R41K
F42*
P59L
H64P
T88I
L92R
c.315+1G>A
p<1e−10
p>0.05
PF00042: Globin

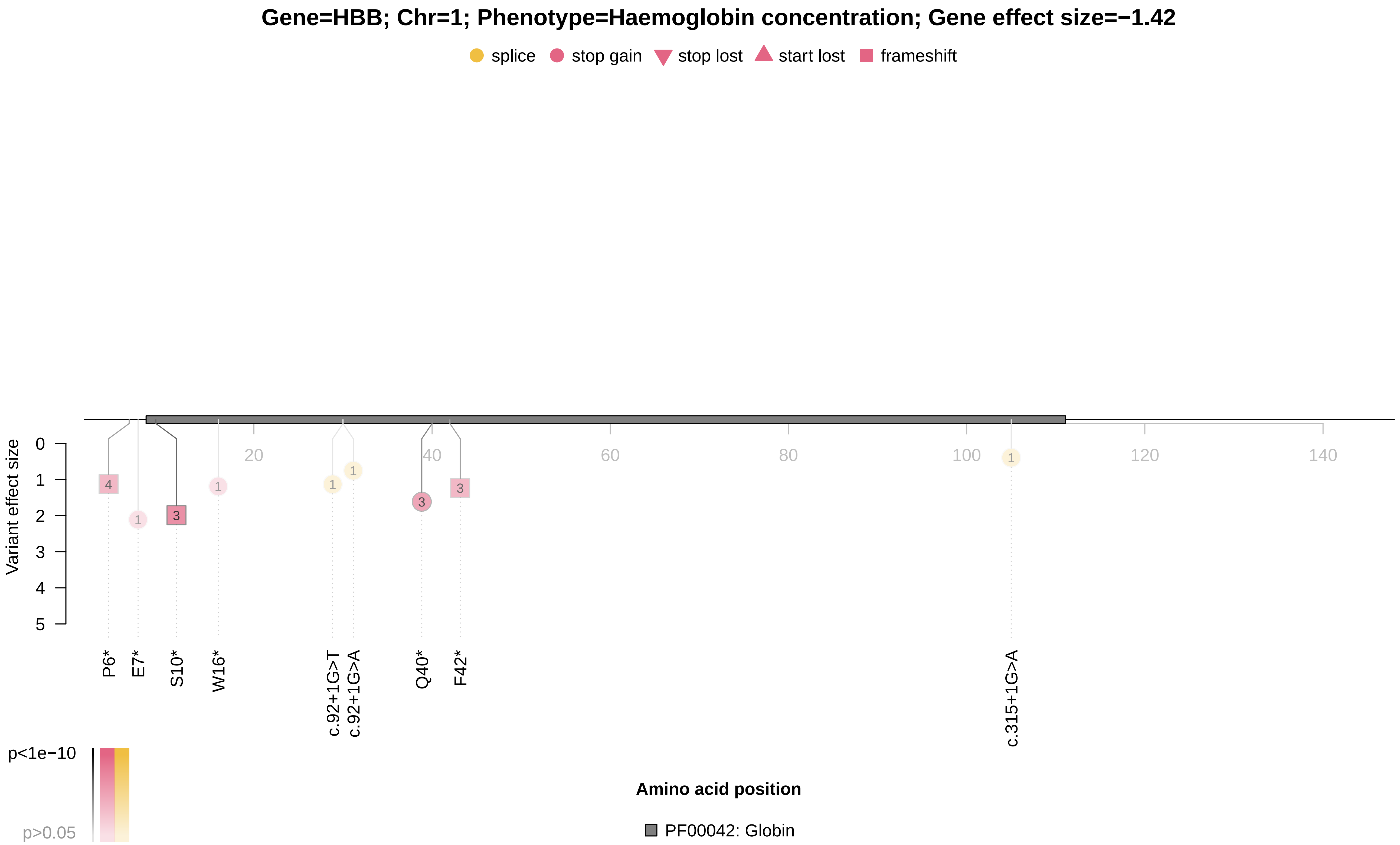
Gene=HBB; Chr=1; Phenotype=Haemoglobin concentration; Gene effect size=−1.42
splice
stop gain
stop lost
start lost
frameshift
Variant effect size
0
1
2
3
4
5
20
40
60
80
100
120
140
4
1
3
1
1
1
3
3
1
P6*
E7*
S10*
W16*
c.92+1G>T
c.92+1G>A
Q40*
F42*
c.315+1G>A
p<1e−10
p>0.05
Amino acid position
PF00042: Globin

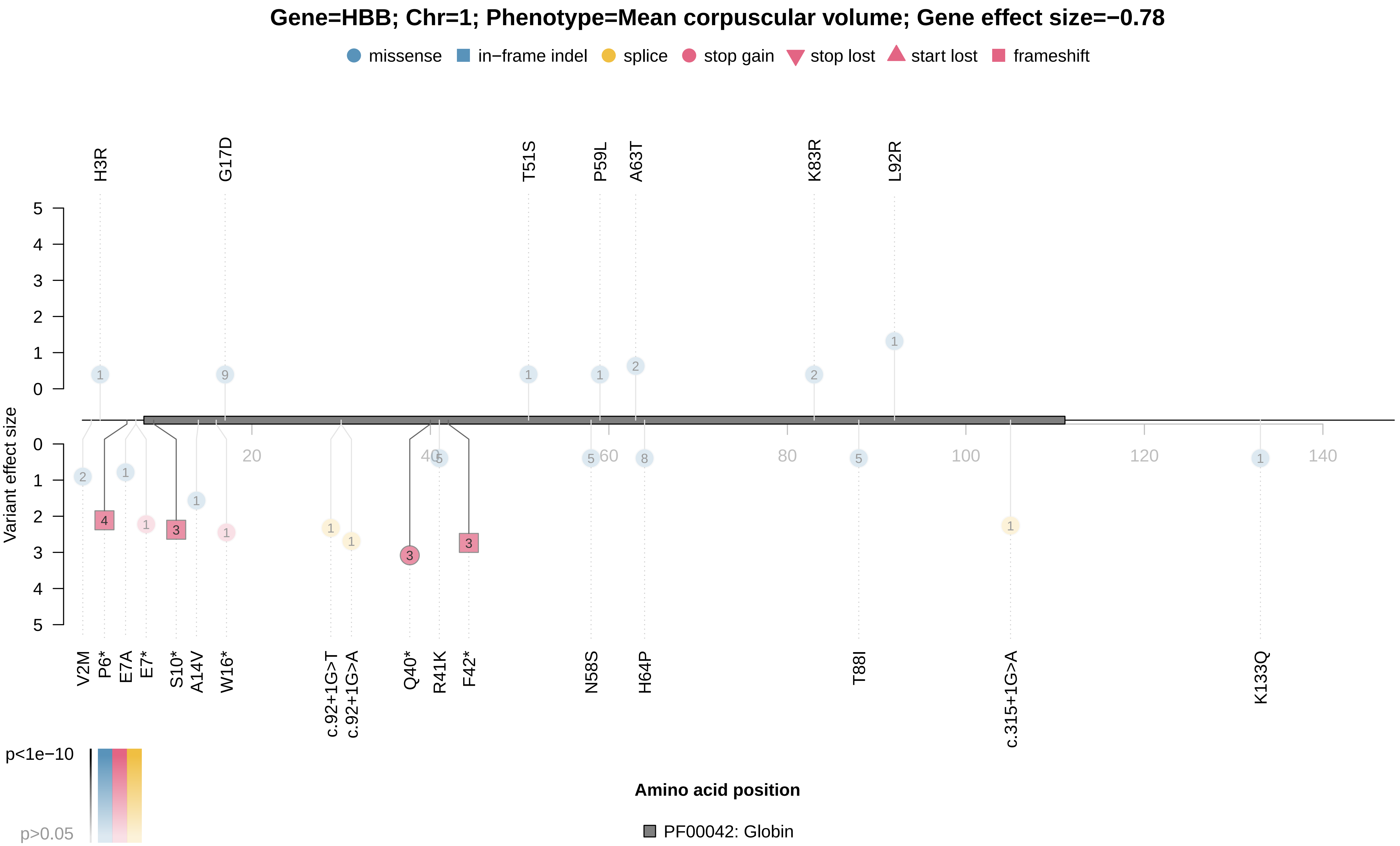

Gene=HBB; Chr=1; Phenotype=Mean corpuscular volume; Gene effect size=−0.78
missense
in−frame indel
splice
stop gain
stop lost
start lost
frameshift
H3R
G17D
T51S
P59L
A63T
K83R
L92R
Variant effect size
V2M
P6*
E7A
E7*
S10*
A14V
W16*
c.92+1G>T
c.92+1G>A
Q40*
R41K
F42*
N58S
H64P
T88I
c.315+1G>A
K133Q
p<1e−10
p>0.05
Amino acid position
PF00042: Globin

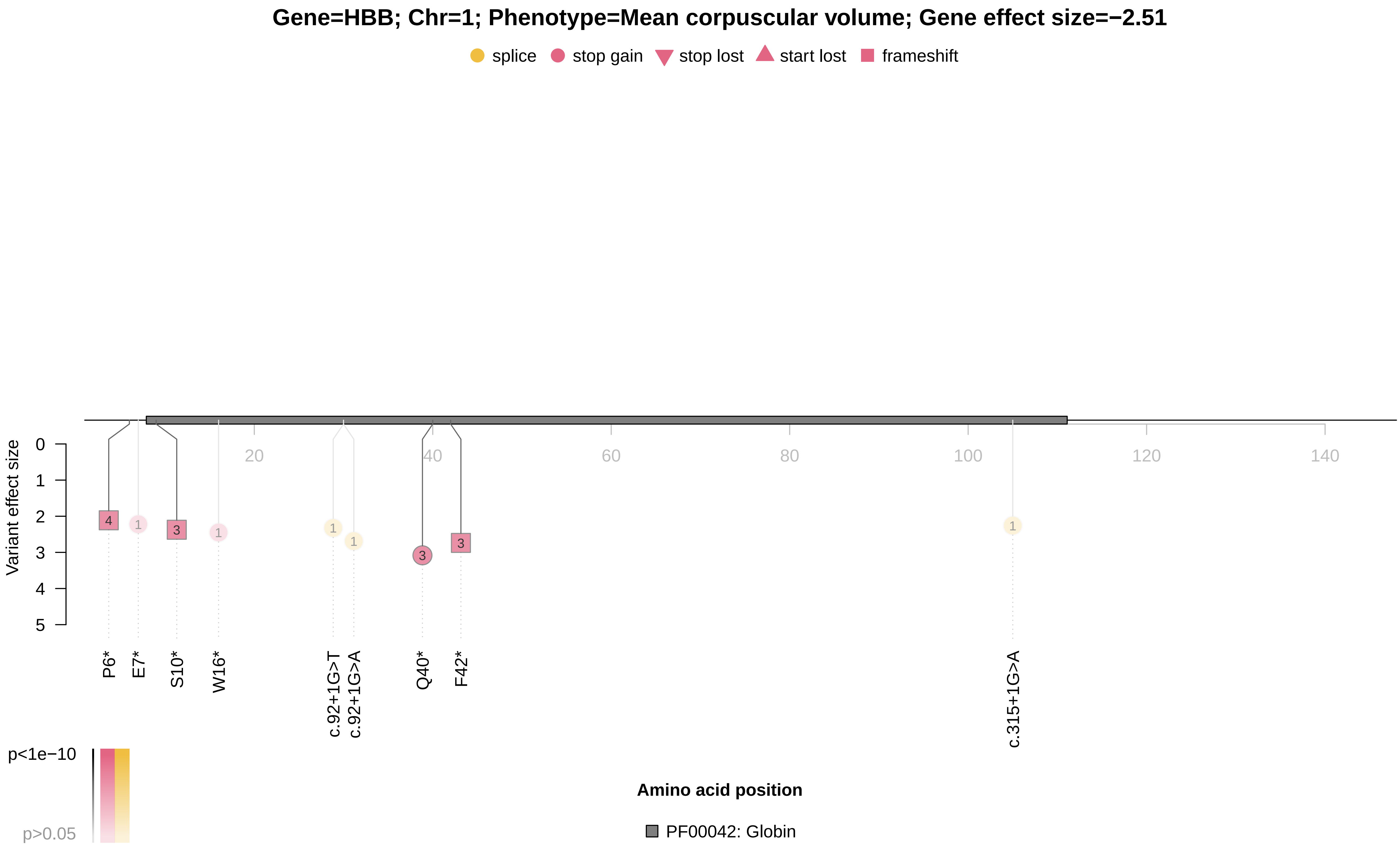

Gene=HBB; Chr=1; Phenotype=Mean corpuscular volume; Gene effect size=−2.51
splice
stop gain
stop lost
start lost
frameshift
Variant effect size
0
1
2
3
4
5
20
40
60
80
100
120
140
P6*
E7*
S10*
W16*
c.92+1G>T
c.92+1G>A
Q40*
F42*
c.315+1G>A
p<1e−10
p>0.05
Amino acid position
PF00042: Globin

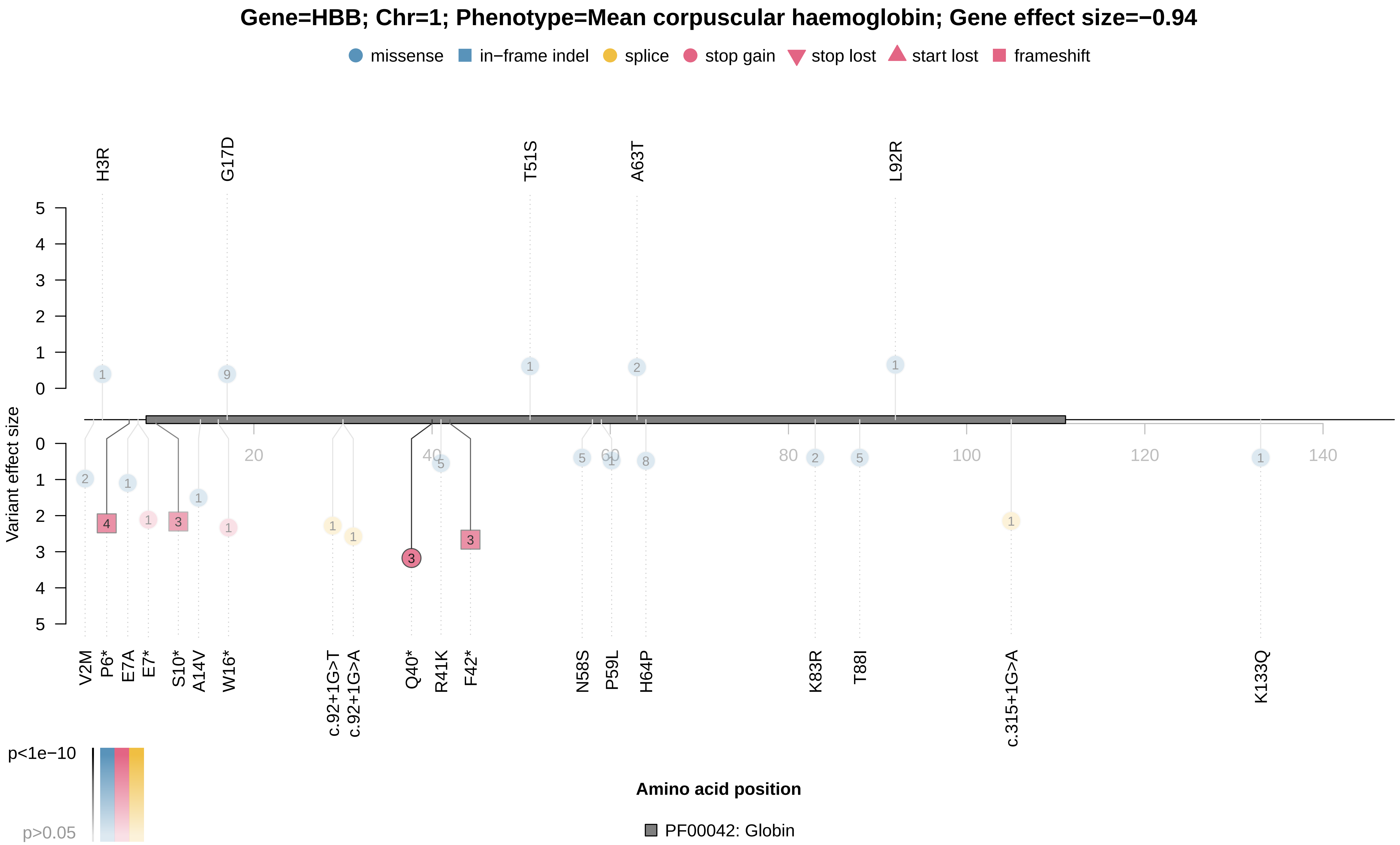

Gene=HBB; Chr=1; Phenotype=Mean corpuscular haemoglobin; Gene effect size=−0.94
missense
in−frame indel
splice
stop gain
stop lost
start lost
frameshift
H3R
G17D
T51S
A63T
L92R
Variant effect size
V2M
P6*
E7A
E7*
S10*
A14V
W16*
c.92+1G>T
c.92+1G>A
Q40*
R41K
F42*
N58S
P59L
H64P
K83R
T88I
c.315+1G>A
K133Q
p<1e−10
p>0.05
Amino acid position
PF00042: Globin

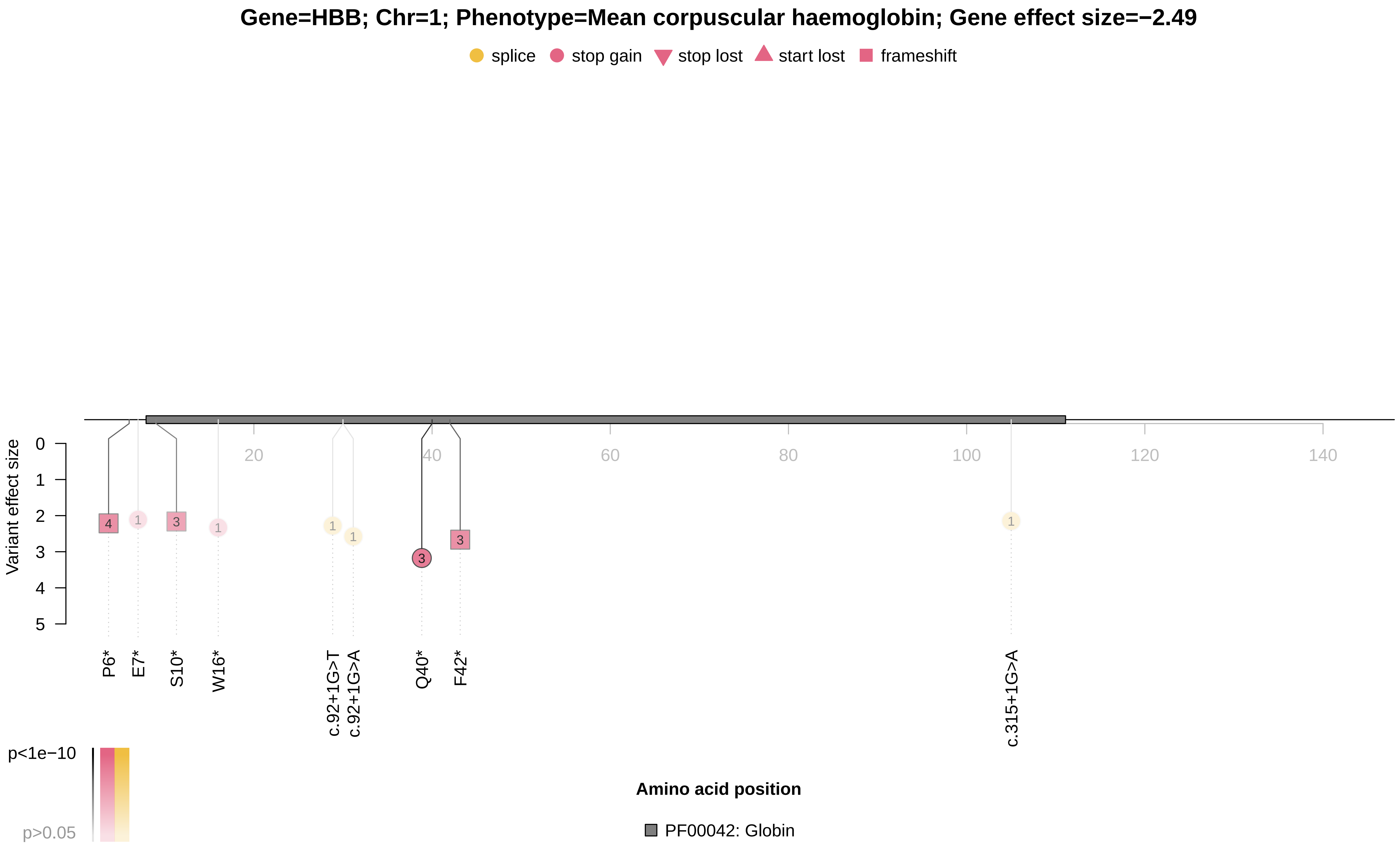

Gene=HBB; Chr=1; Phenotype=Mean corpuscular haemoglobin; Gene effect size=−2.49
splice
stop gain
stop lost
start lost
frameshift
Variant effect size
0
1
2
3
4
5
20
40
60
80
100
120
140
4
1
3
1
1
1
3
3
1
P6*
E7*
S10*
W16*
c.92+1G>T
c.92+1G>A
Q40*
F42*
c.315+1G>A
p<1e−10
p>0.05
Amino acid position
PF00042: Globin

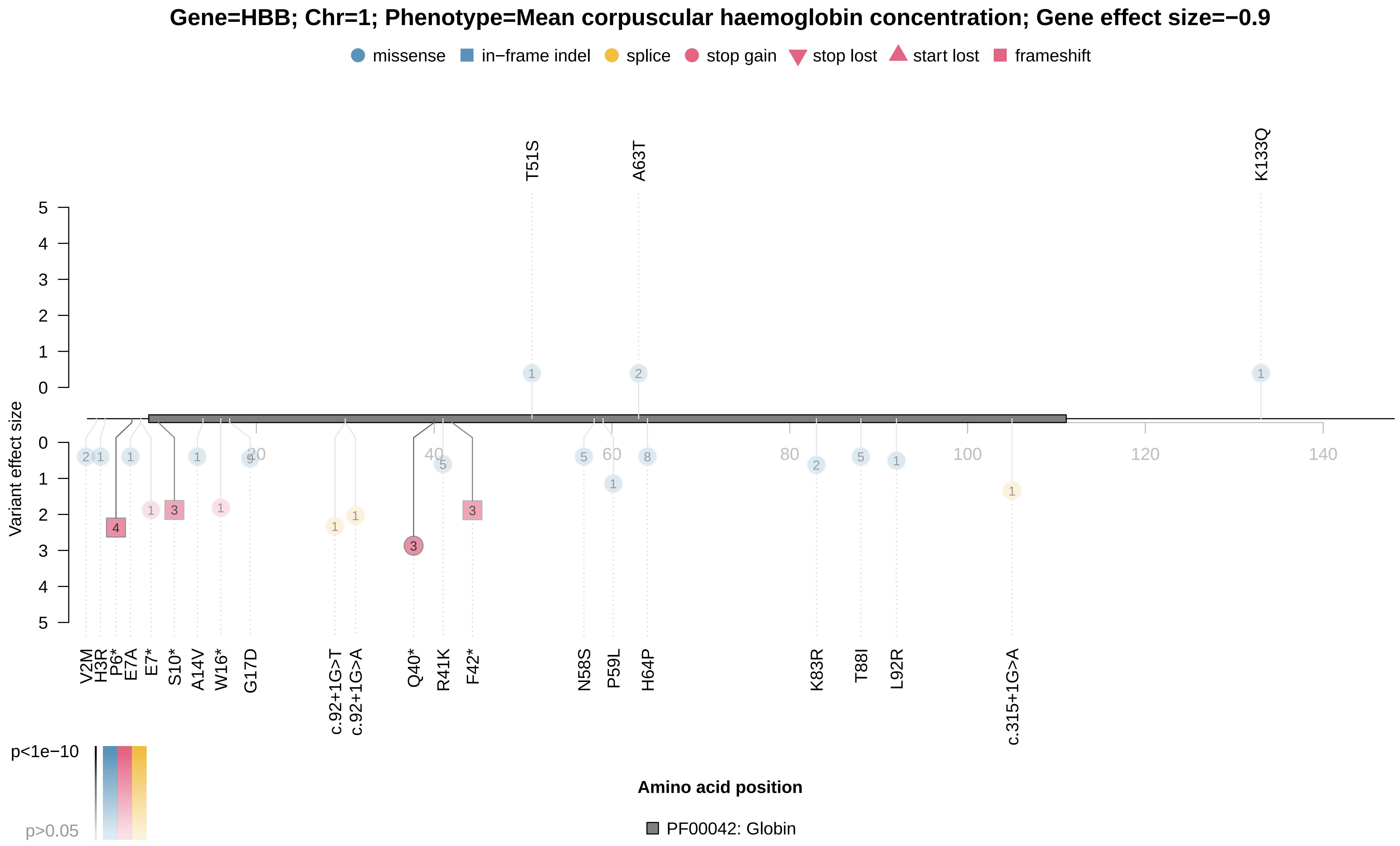
Gene=HBB; Chr=1; Phenotype=Mean corpuscular haemoglobin concentration; Gene effect size=−0.9
missense
in−frame indel
splice
stop gain
stop lost
start lost
frameshift
T51S
A63T
K133Q
Variant effect size
V2M
H3R
P6*
E7A
E7*
S10*
A14V
W16*
G17D
c.92+1G>T
c.92+1G>A
Q40*
R41K
F42*
N58S
P59L
H64P
K83R
T88I
L92R
c.315+1G>A
p<1e−10
p>0.05
Amino acid position
PF00042: Globin

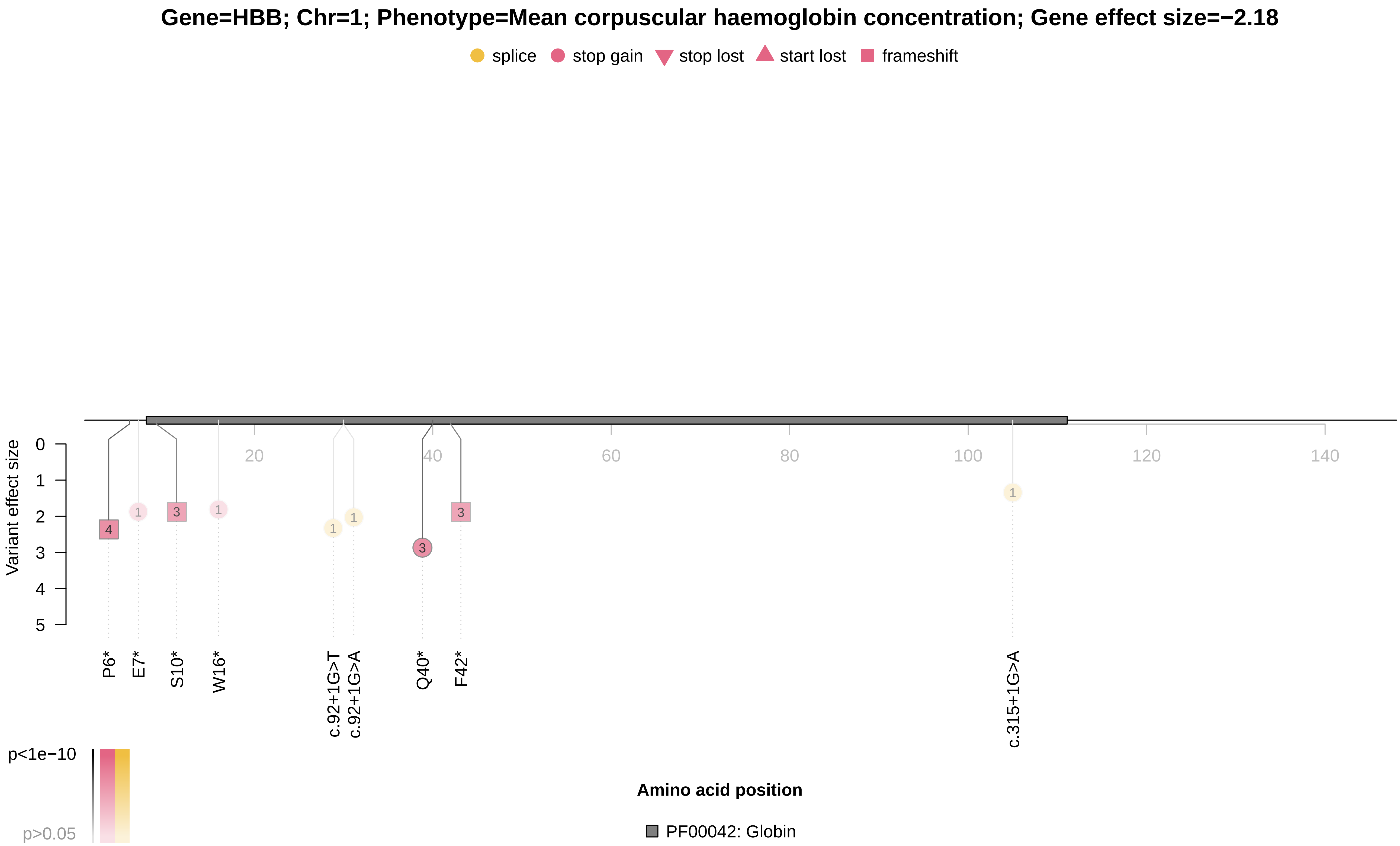

Gene=HBB; Chr=1; Phenotype=Mean corpuscular haemoglobin concentration; Gene effect size=−2.18
splice
stop gain
stop lost
start lost
frameshift
Variant effect size
0
1
2
3
4
5
20
40
60
80
100
120
140
4
1
3
1
1
1
3
3
1
P6*
E7*
S10*
W16*
c.92+1G>T
c.92+1G>A
Q40*
F42*
c.315+1G>A
p<1e−10
p>0.05
Amino acid position
PF00042: Globin

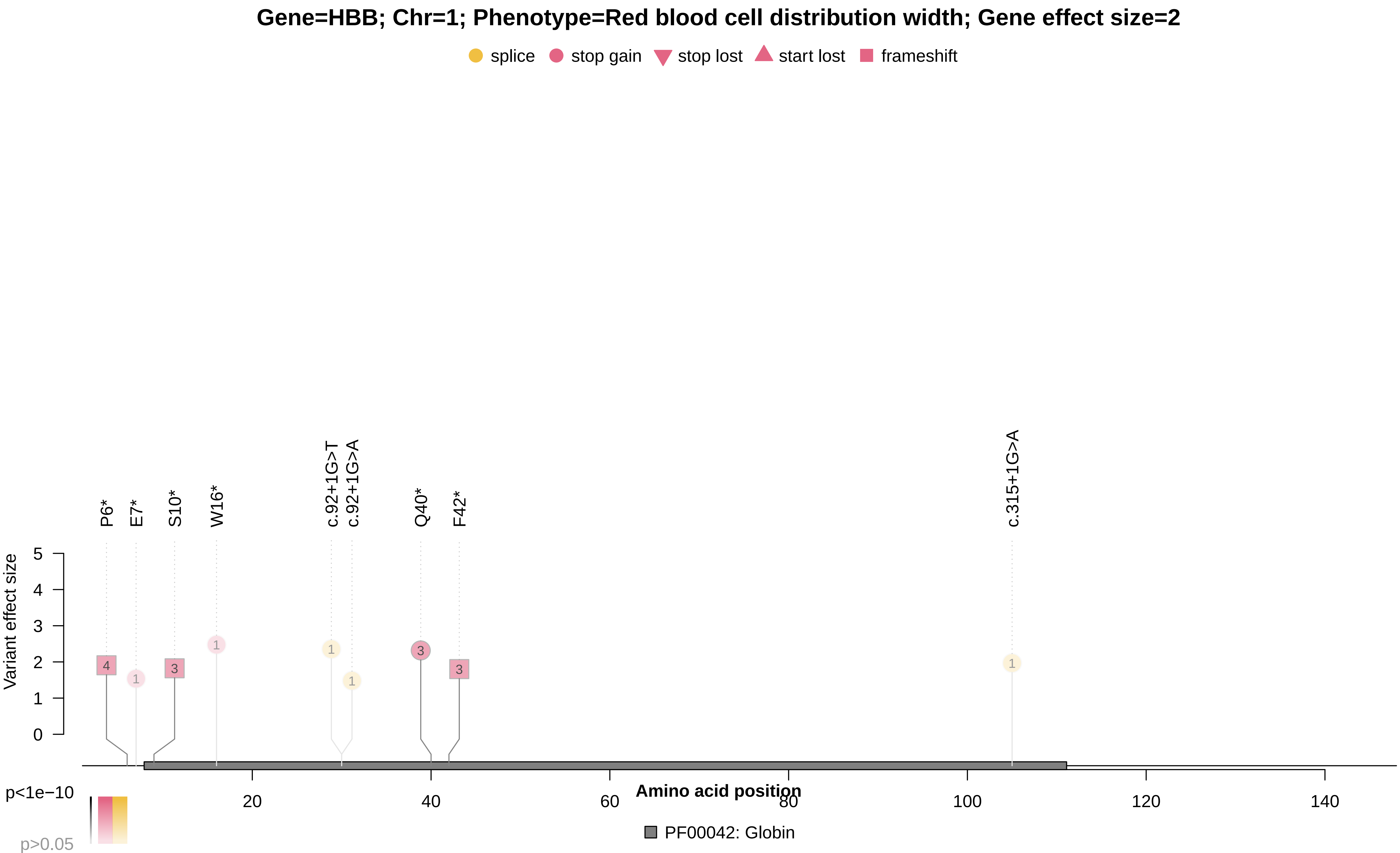
Gene=HBB; Chr=1; Phenotype=Red blood cell distribution width; Gene effect size=2
splice
stop gain
stop lost
start lost
frameshift
P6*
E7*
S10*
W16*
c.92+1G>T
c.92+1G>A
Q40*
F42*
c.315+1G>A
Variant effect size
0
1
2
3
4
5
4
1
3
1
1
1
3
3
1
20
40
60
80
100
120
140
Amino acid position
p<1e−10
p>0.05
PF00042: Globin

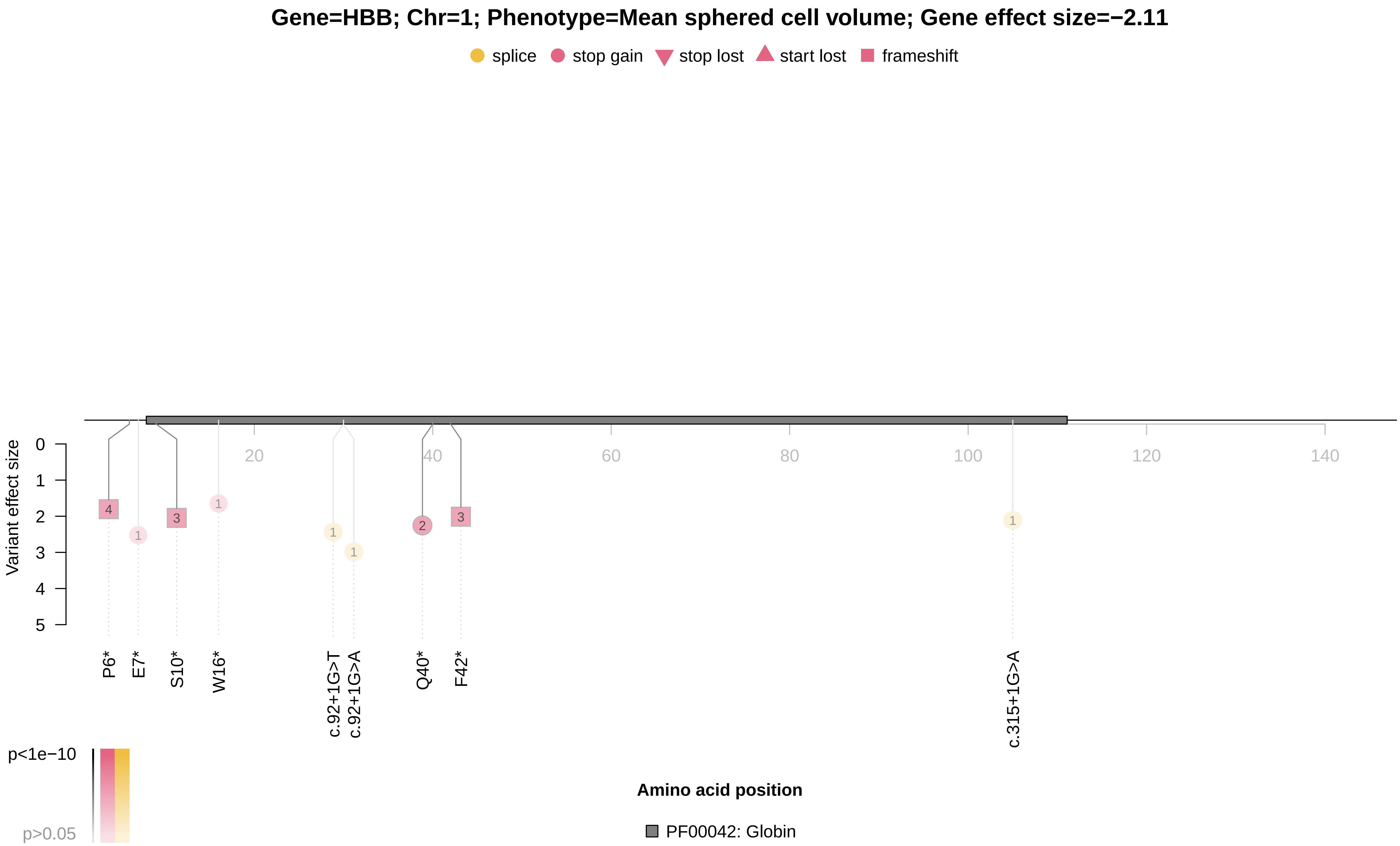

Gene=HBB; Chr=1; Phenotype=Mean sphered cell volume; Gene effect size=−2.11
splice
stop gain
stop lost
start lost
frameshift
Variant effect size
0
1
2
3
4
5
20
40
60
80
100
120
140
P6*
E7*
S10*
W16*
c.92+1G>T
c.92+1G>A
Q40*
F42*
c.315+1G>A
p<1e−10
p>0.05
Amino acid position
PF00042: Globin

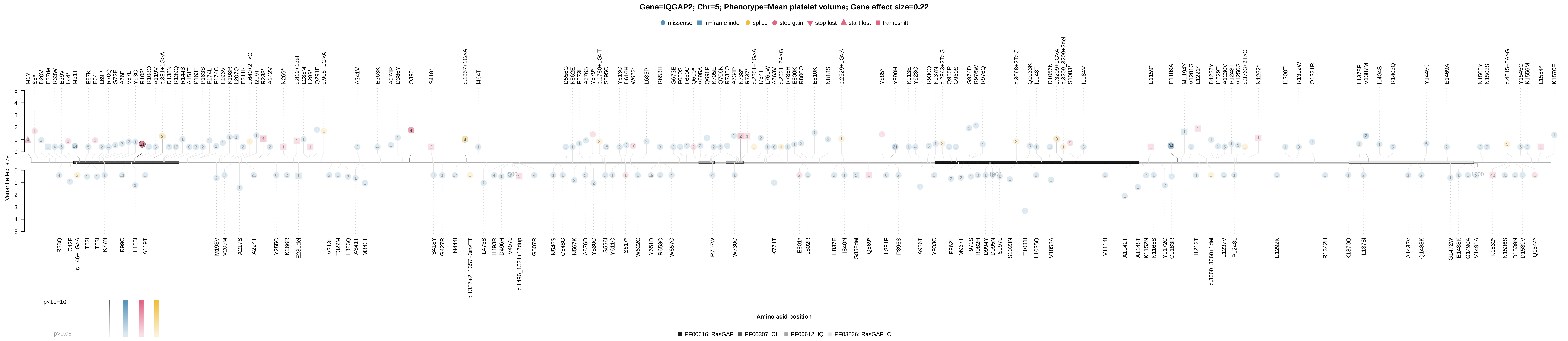

Gene=IQGAP2; Chr=5; Phenotype=Mean platelet volume; Gene effect size=0.22
missense
in-frame indel
splice
stop gain
stop lost
start lost
frameshift
Variant effect size
Amino acid position
p<1e-10
p>0.05
PF00616: RasGAP
PF00307: CH
PF00612: IQ
PF03836: RasGAP_C

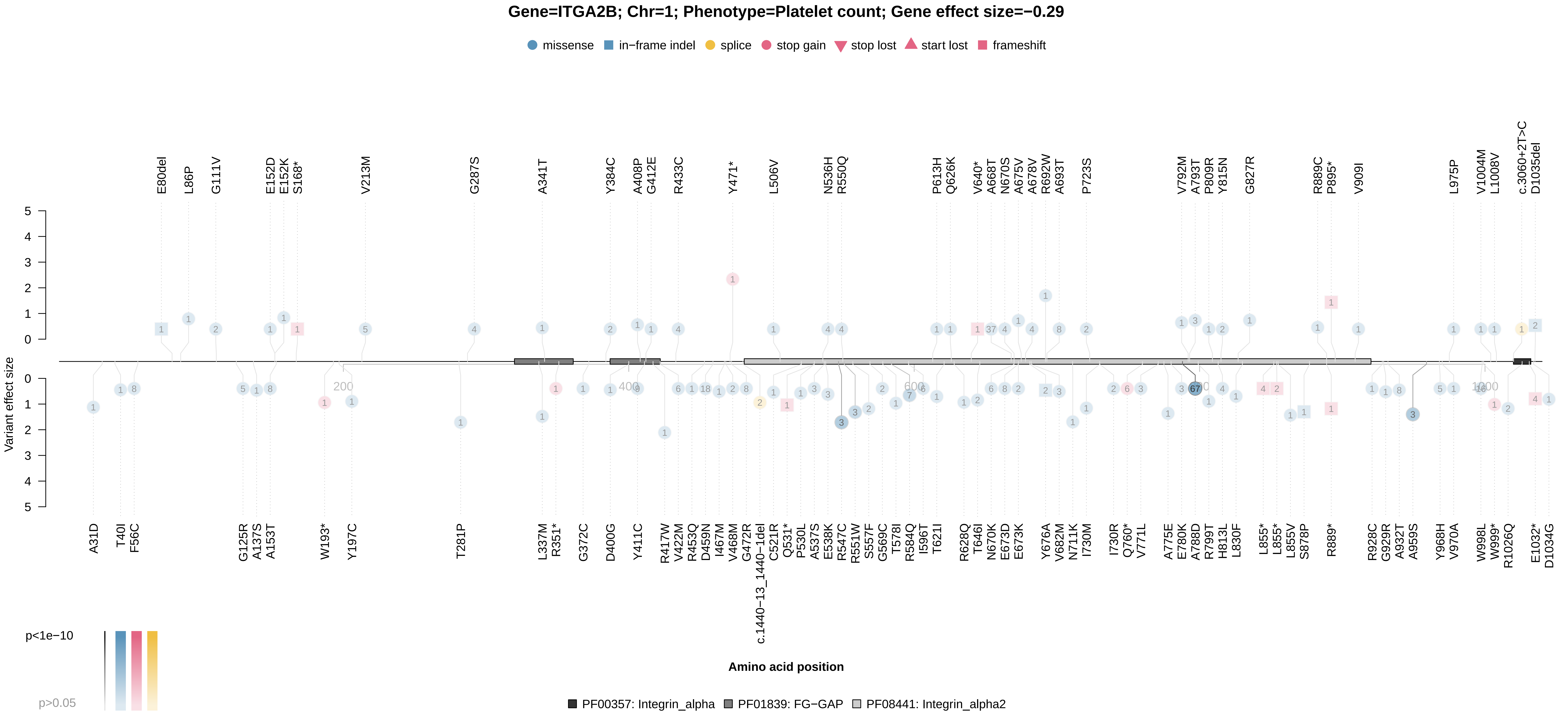

Gene=ITGA2B; Chr=1; Phenotype=Platelet count; Gene effect size=−0.29
missense
in−frame indel
splice
stop gain
stop lost
start lost
frameshift
Variant effect size
Amino acid position
p<1e−10
p>0.05
PF00357: Integrin_alpha
PF01839: FG−GAP
PF08441: Integrin_alpha2

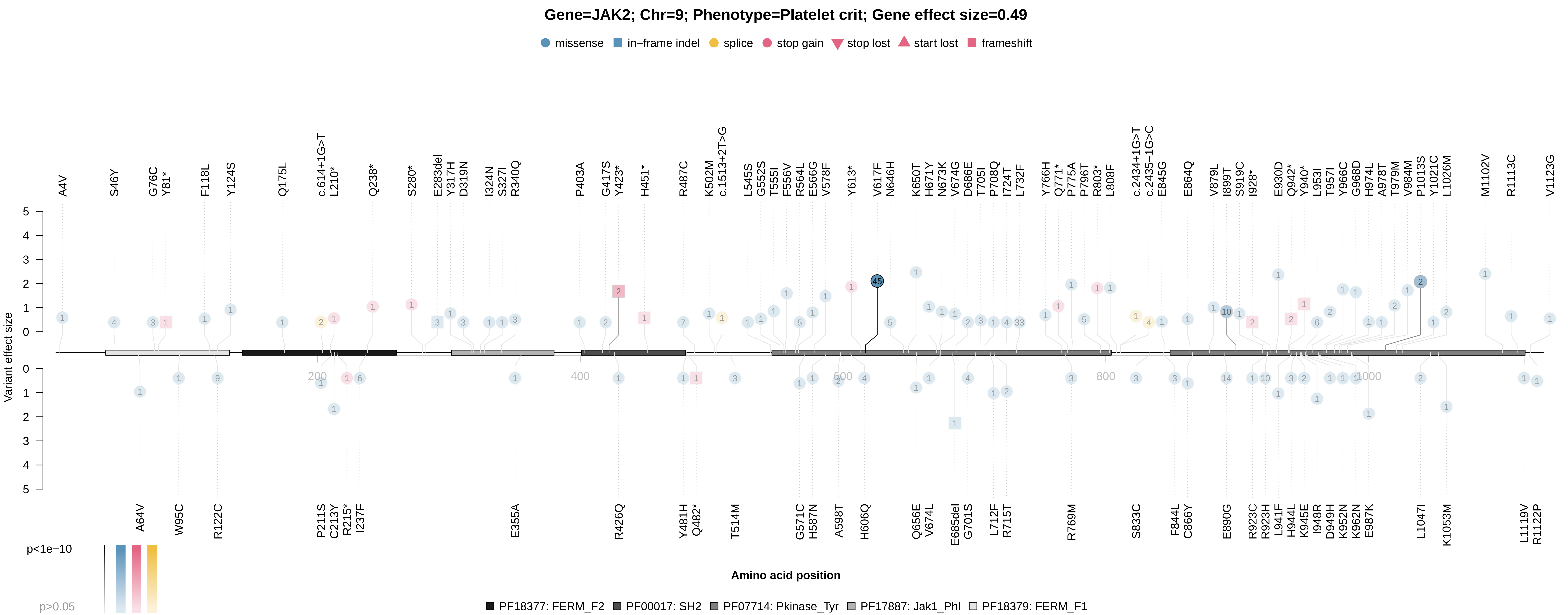

Gene=JAK2; Chr=9; Phenotype=Platelet crit; Gene effect size=0.49
missense
in−frame indel
splice
stop gain
stop lost
start lost
frameshift
A4V
S46Y
G76C
Y81*
F118L
Y124S
Q175L
c.614+1G>T
L210*
Q238*
S280*
E283del
Y317H
D319N
I324N
S327I
R340Q
P403A
G417S
Y423*
H451*
R487C
K502M
c.1513+2T>G
L545S
G552S
T555I
F556V
R564L
E566G
V578F
Y613*
V617F
N646H
K650T
H671Y
N673K
V674G
D686E
T705I
P708Q
I724T
L732F
Y766H
Q771*
P775A
P796T
R803*
L808F
c.2434+1G>T
c.2435−1G>C
E845G
E864Q
V879L
I899T
S919C
I928*
E930D
Q942*
Y940*
L953I
T957I
Y966C
G968D
H974L
A978T
T979M
V984M
P1013S
Y1021C
L1026M
M1102V
R1113C
V1123G
Variant effect size
200
400
600
800
1000
A64V
W95C
R122C
P211S
C213Y
R215*
I237F
E355A
R426Q
Y481H
Q482*
T514M
G571C
H587N
A598T
H606Q
Q656E
V674L
E685del
G701S
L712F
R715T
R769M
S833C
F844L
C866Y
E890G
R923C
R923H
L941F
H944L
K945E
I948R
D949H
K952N
K962N
E987K
L1047I
K1053M
L1119V
R1122P
p<1e−10
p>0.05
Amino acid position
PF18377: FERM_F2
PF00017: SH2
PF07714: Pkinase_Tyr
PF17887: Jak1_Phl
PF18379: FERM_F1

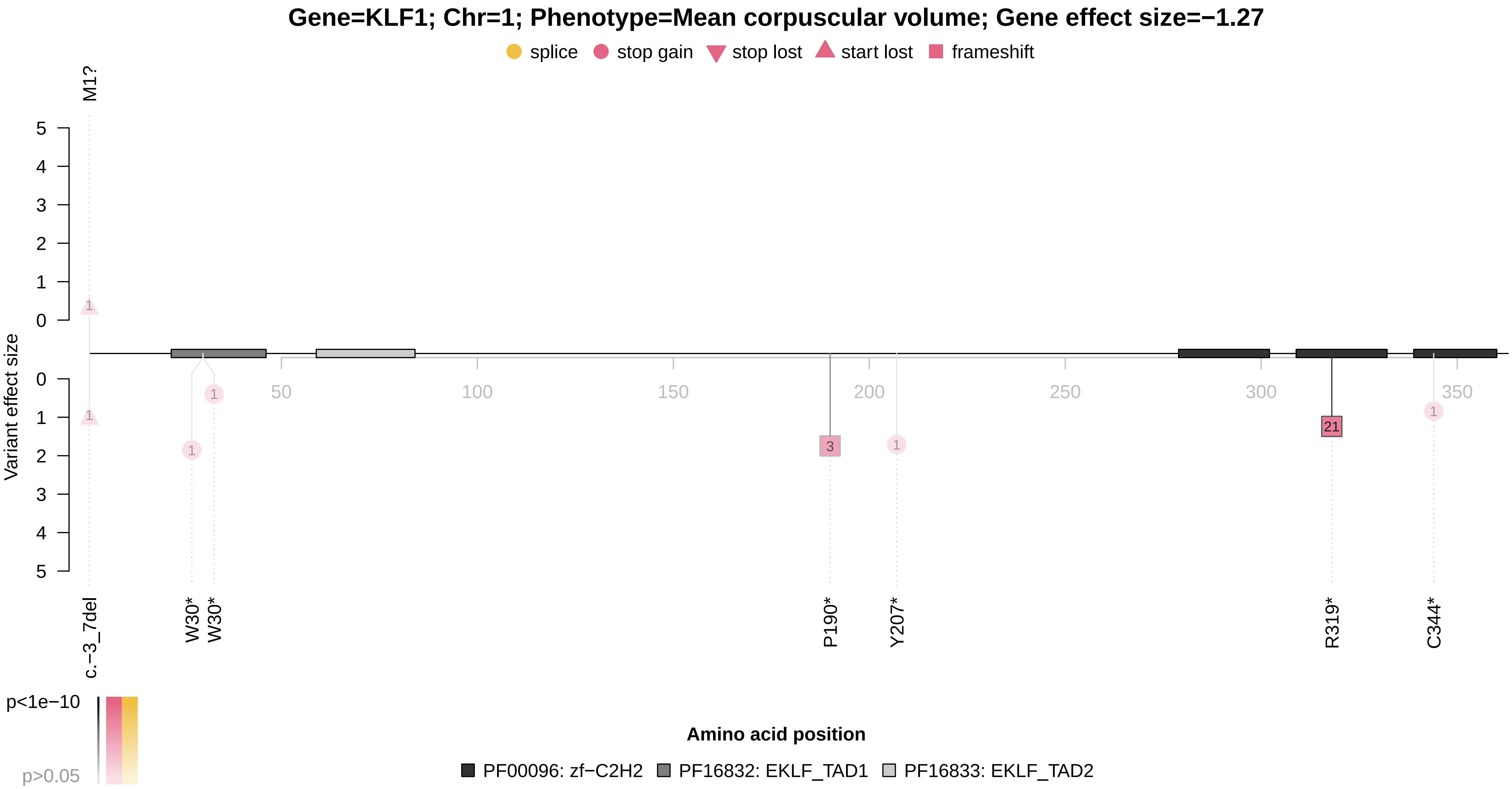

Gene=KLF1; Chr=1; Phenotype=Mean corpuscular volume; Gene effect size=−1.27
splice
stop gain
stop lost
start lost
frameshift
M1?
Variant effect size
c.−3_7del
W30*
W30*
P190*
Y207*
R319*
C344*
p<1e−10
p>0.05
Amino acid position
PF00096: zf−C2H2
PF16832: EKLF_TAD1
PF16833: EKLF_TAD2

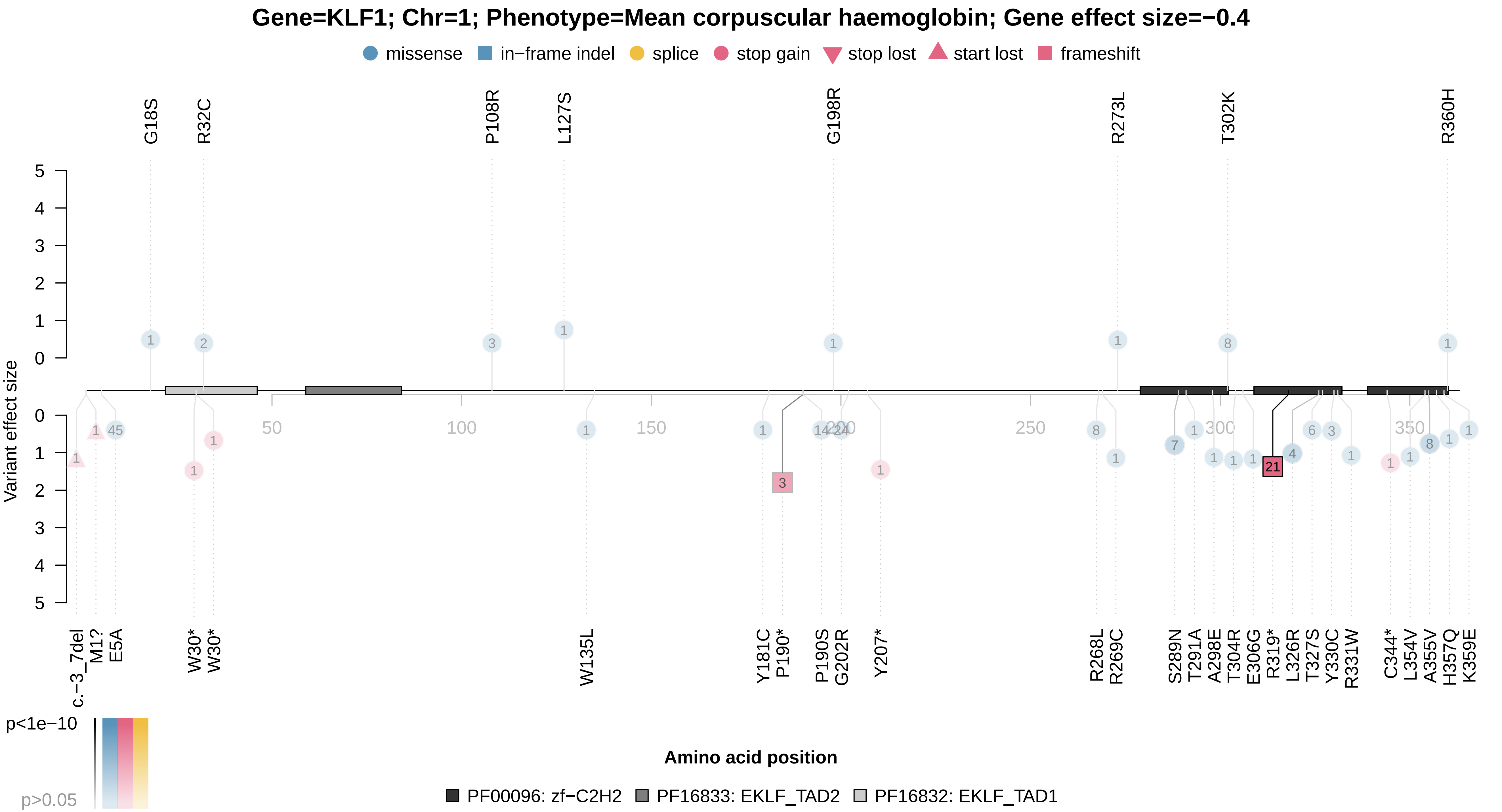

Gene=KLF1; Chr=1; Phenotype=Mean corpuscular haemoglobin; Gene effect size=−0.4
missense
in−frame indel
splice
stop gain
stop lost
start lost
frameshift
G18S
R32C
P108R
L127S
G198R
R273L
T302K
R360H
Variant effect size
Amino acid position
c.−3_7del
M1?
E5A
W30*
W30*
W135L
Y181C
P190*
P190S
G202R
Y207*
R268L
R269C
S289N
T291A
A298E
T304R
E306G
R319*
L326R
T327S
Y330C
R331W
C344*
L354V
A355V
H357Q
K359E
p<1e−10
p>0.05
PF00096: zf−C2H2
PF16833: EKLF_TAD2
PF16832: EKLF_TAD1

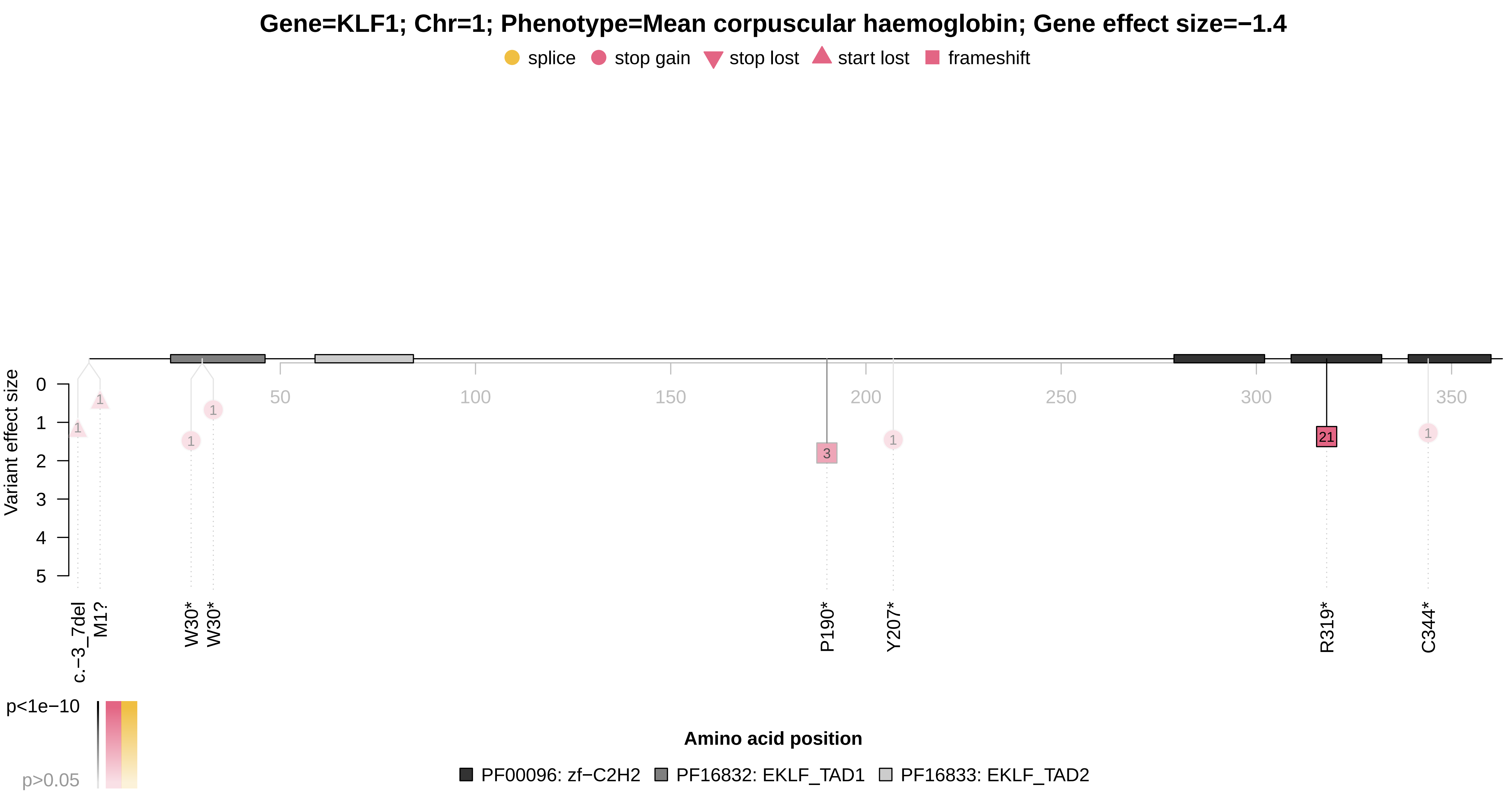

Gene=KLF1; Chr=1; Phenotype=Mean corpuscular haemoglobin; Gene effect size=−1.4
splice
stop gain
stop lost
start lost
frameshift
Variant effect size
0
1
2
3
4
5
50
100
150
200
250
300
350
1
1
1
1
3
1
21
1
c.−3_7del
M1?
W30*
W30*
P190*
Y207*
R319*
C344*
p<1e−10
p>0.05
Amino acid position
PF00096: zf−C2H2
PF16832: EKLF_TAD1
PF16833: EKLF_TAD2

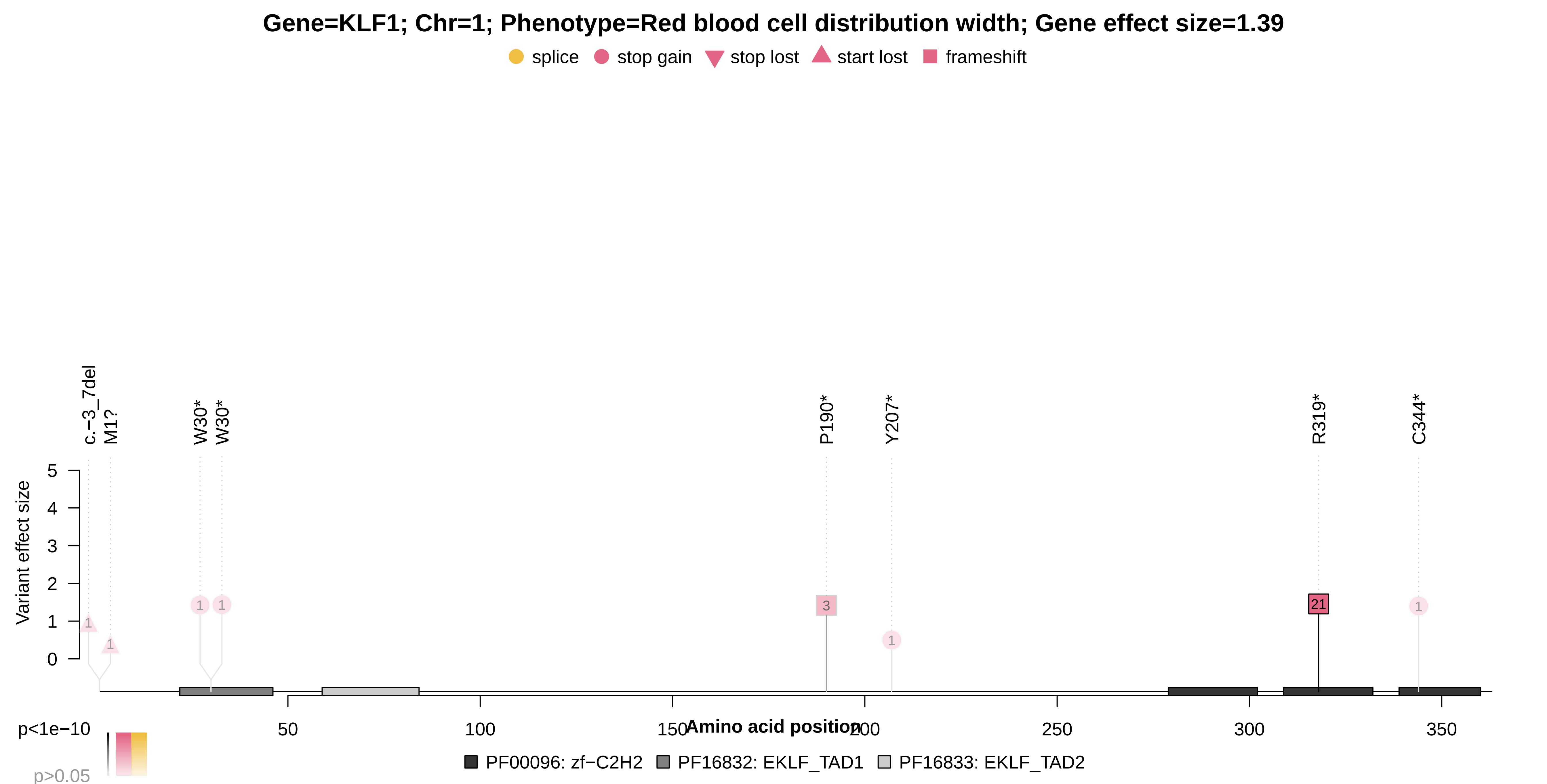

Gene=KLF1; Chr=1; Phenotype=Red blood cell distribution width; Gene effect size=1.39
splice
stop gain
stop lost
start lost
frameshift
c.-3_7del
M1?
W30*
W30*
P190*
Y207*
R319*
C344*
Variant effect size
5
4
3
2
1
0
1
1
1
1
3
1
21
1
50
100
150
200
250
300
350
Amino acid position
p<1e-10
p>0.05
PF00096: zf-C2H2
PF16832: EKLF_TAD1
PF16833: EKLF_TAD2

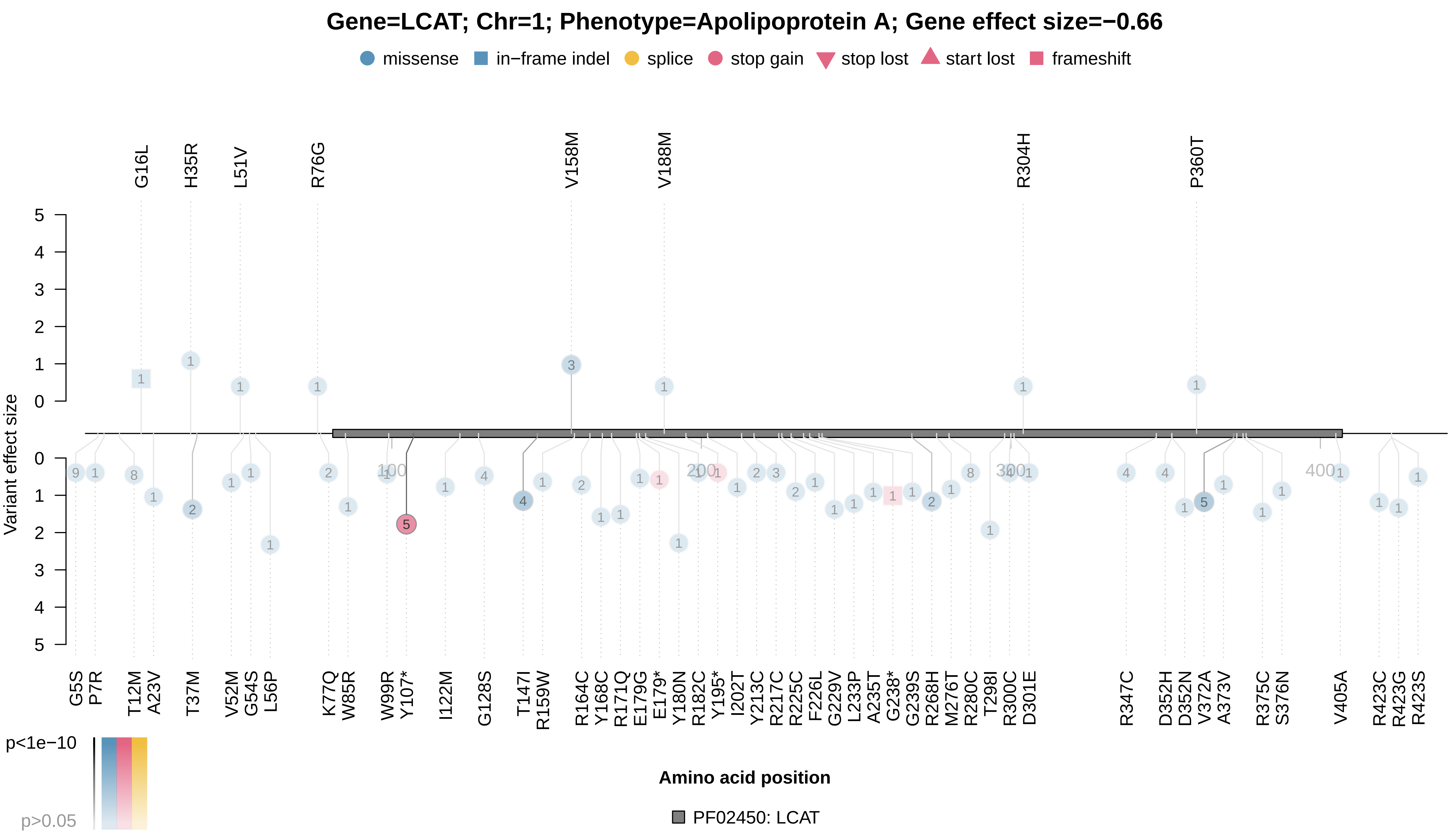
Gene=LCAT; Chr=1; Phenotype=Apolipoprotein A; Gene effect size=−0.66
missense
in−frame indel
splice
stop gain
stop lost
start lost
frameshift
G16L
H35R
L51V
R76G
V158M
V188M
R304H
P360T
Variant effect size
100
200
300
400
G5S
P7R
T12M
A23V
T37M
V52M
G54S
L56P
K77Q
W85R
W99R
Y107*
I122M
G128S
T147I
R159W
R164C
Y168C
R171Q
E179G
E179*
Y180N
R182C
Y195*
I202T
Y213C
R217C
R225C
F226L
G229V
L233P
A235T
G238*
G239S
R268H
M276T
R280C
T298I
R300C
D301E
R347C
D352H
D352N
V372A
A373V
R375C
S376N
V405A
R423C
R423G
R423S
p<1e−10
p>0.05
Amino acid position
PF02450: LCAT

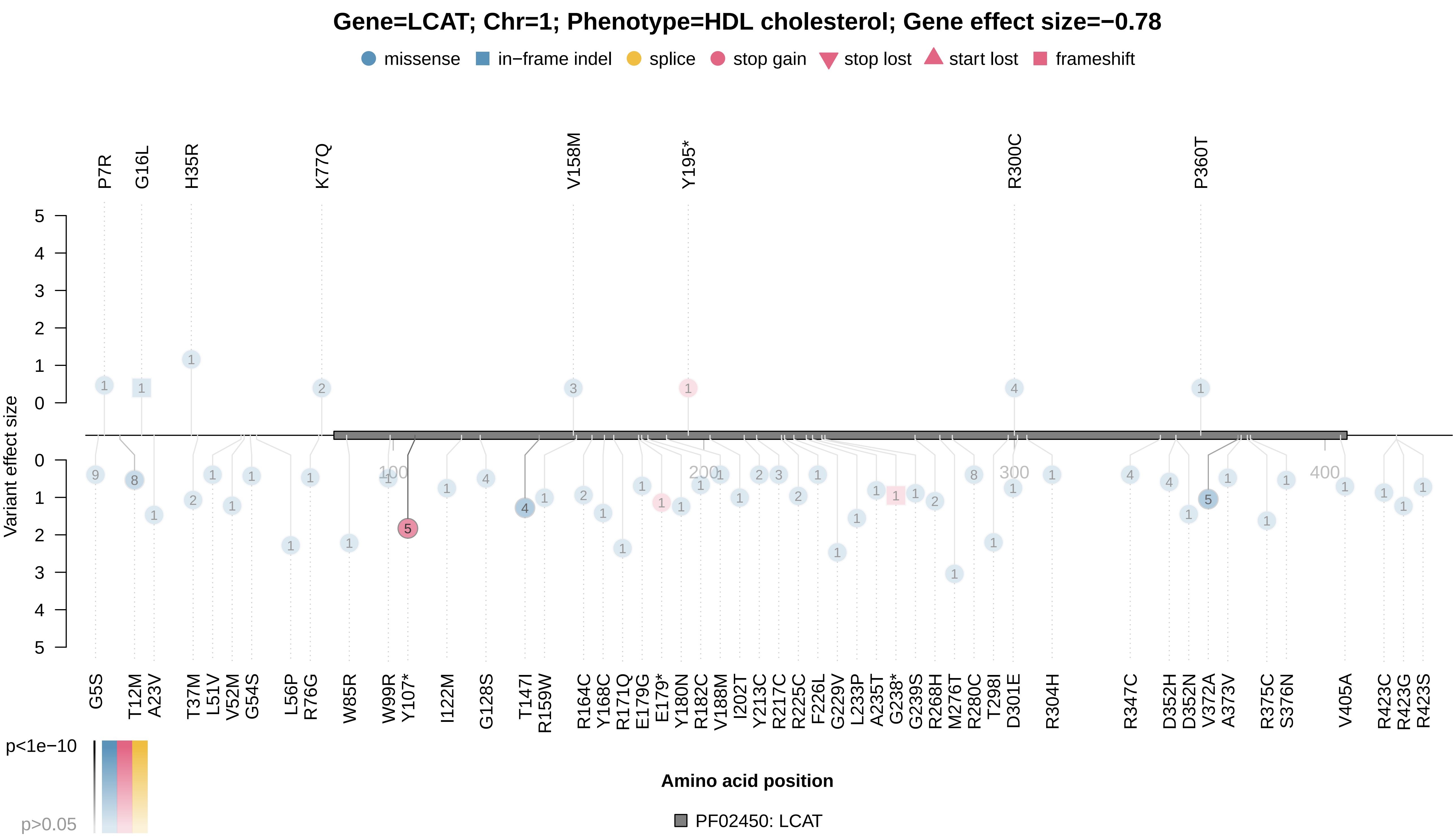

Gene=LCAT; Chr=1; Phenotype=HDL cholesterol; Gene effect size=−0.78
missense
in−frame indel
splice
stop gain
stop lost
start lost
frameshift
P7R
G16L
H35R
K77Q
V158M
Y195*
R300C
P360T
Variant effect size
100
200
300
400
G5S
T12M
A23V
T37M
L51V
V52M
G54S
L56P
R76G
W85R
W99R
Y107*
I122M
G128S
T147I
R159W
R164C
Y168C
R171Q
E179G
E179*
Y180N
R182C
V188M
I202T
Y213C
R217C
R225C
F226L
G229V
L233P
A235T
G238*
G239S
R268H
M276T
R280C
T298I
D301E
R304H
R347C
D352H
D352N
V372A
A373V
R375C
S376N
V405A
R423C
R423G
R423S
p<1e−10
p>0.05
Amino acid position
PF02450: LCAT

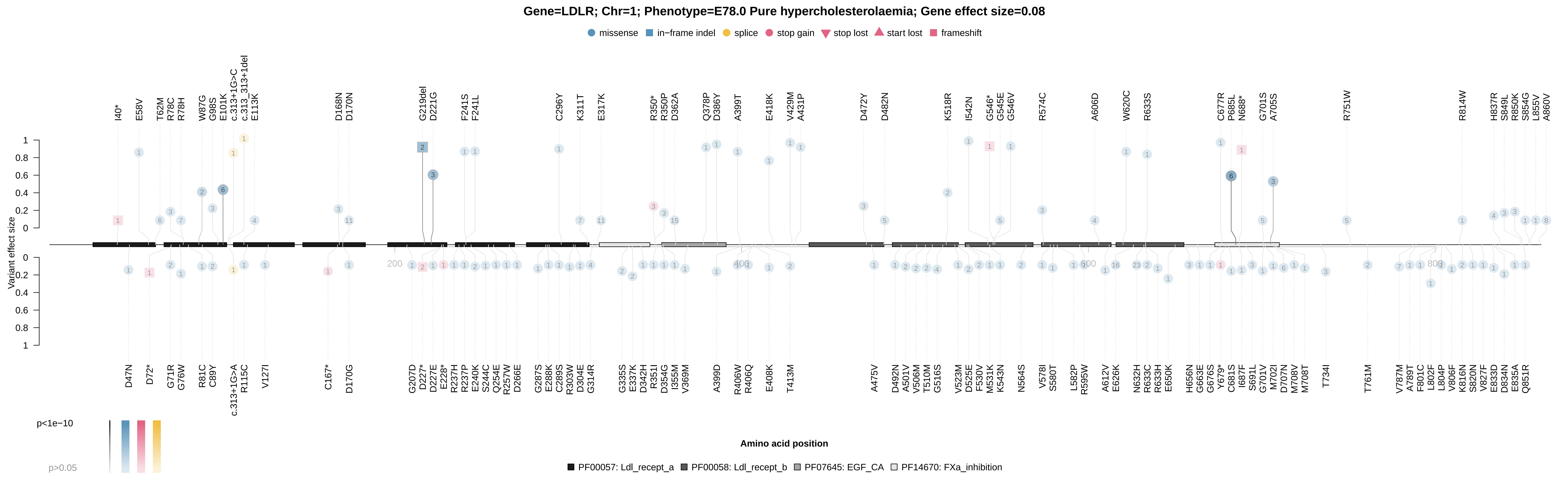
Gene=LDLR; Chr=1; Phenotype=E78.0 Pure hypercholesterolaemia; Gene effect size=0.08
missense
in−frame indel
splice
stop gain
stop lost
start lost
frameshift
I40*
E58V
T62M
R78C
R78H
W87G
G98S
E101K
c.313+1G>C
c.313_313+1del
E113K
D168N
D170N
G219del
D221G
F241S
F241L
C296Y
K311T
E317K
R350*
R350P
D362A
Q378P
D386Y
A399T
E418K
V429M
A431P
D472Y
D482N
K518R
I542N
G546*
G545E
G546V
R574C
A606D
W620C
R633S
C677R
P685L
N688*
G701S
A705S
R751W
R814W
H837R
S849L
R850K
S854G
L855V
A860V
Variant effect size
D47N
D72*
G71R
G76W
R81C
C89Y
c.313+1G>A
R115C
V127I
C167*
D170G
G207D
D227*
D227E
E228*
R237H
R237P
E240K
S244C
Q254E
R257W
D266E
G287S
E288K
C289S
R303W
D304E
G314R
G335S
E337K
D342H
R351I
D354G
I355M
V369M
A399D
R406W
R406Q
E408K
T413M
A475V
D492N
A501V
V506M
T510M
G516S
V523M
D525E
F530V
M531K
K543N
N564S
V578I
S580T
L582P
R595W
A612V
E626K
N632H
R633C
R633H
E650K
H656N
G663E
G676S
Y679*
C681S
I687F
S691L
G701V
M702I
D707N
M708V
M708T
T734I
T761M
V787M
A789T
F801C
L802F
L804P
V806F
K816N
S820N
V827F
E833D
D834N
E835A
Q851R
p<1e−10
p>0.05
Amino acid position
PF00057: Ldl_recept_a
PF00058: Ldl_recept_b
PF07645: EGF_CA
PF14670: FXa_inhibition

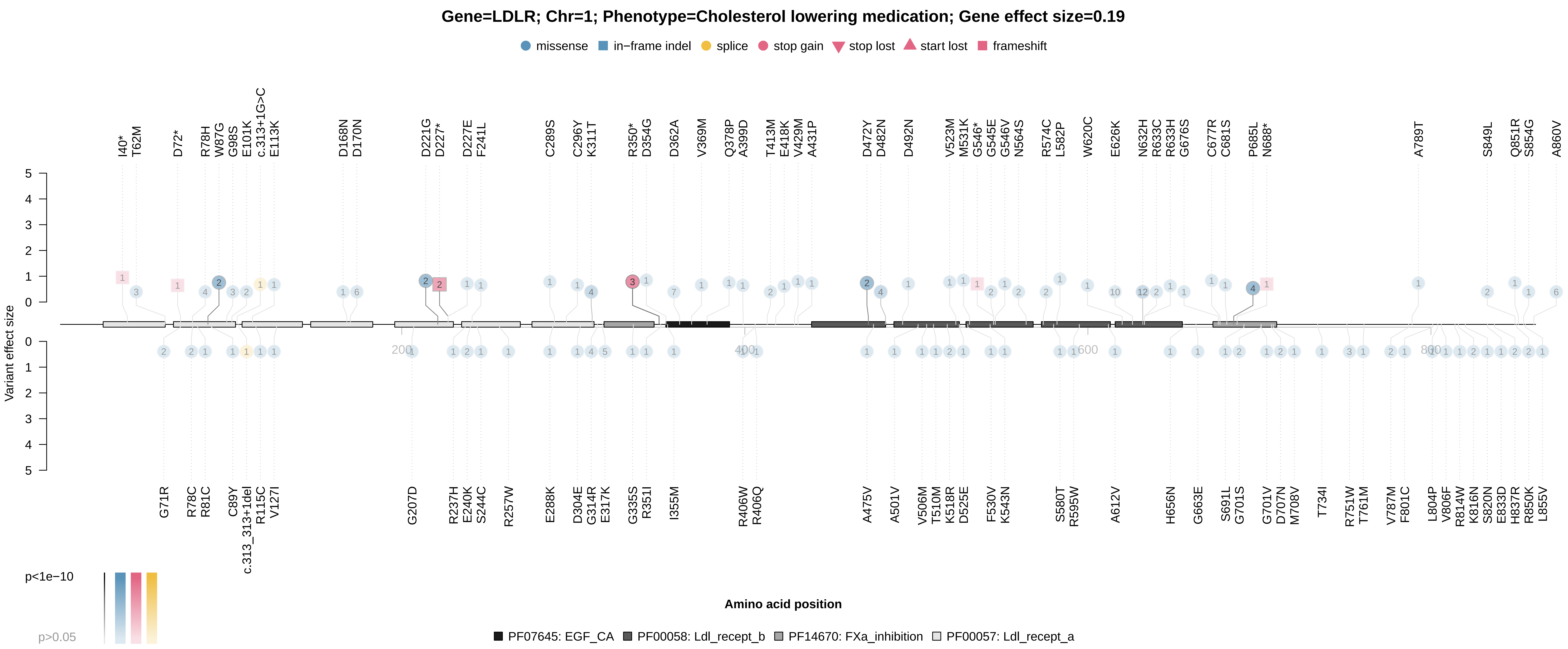

Gene=LDLR; Chr=1; Phenotype=Cholesterol lowering medication; Gene effect size=0.19
missense
in−frame indel
splice
stop gain
stop lost
start lost
frameshift
Variant effect size
Amino acid position
p<1e−10
p>0.05
PF07645: EGF_CA
PF00058: Ldl_recept_b
PF14670: FXa_inhibition
PF00057: Ldl_recept_a
I40* T62M D72* R78H W87G G98S E101K c.313+1G>C E113K D168N D170N D221G D227* D227E F241L C289S C296Y K311T R350* D354G D362A V369M Q378P A399D T413M E418K V429M A431P D472Y D482N D492N V523M M531K G546* G545E G546V N564S R574C L582P W620C E626K N632H R633C R633H G676S C677R C681S P685L N688* A789T S849L Q851R S854G A860V
G71R R78C R81C C89Y c.313_313+1del R115C V127I G207D R237H E240K S244C R257W E288K D304E G314R E317K G335S R351I I355M R406W R406Q A475V A501V V506M T510M K518R D525E F530V K543N S580T R595W A612V H656N G663E S691L G701S G701V D707N M708V T734I R751W T761M V787M F801C L804P V806F R814W K816N S820N E833D H837R R850K L855V

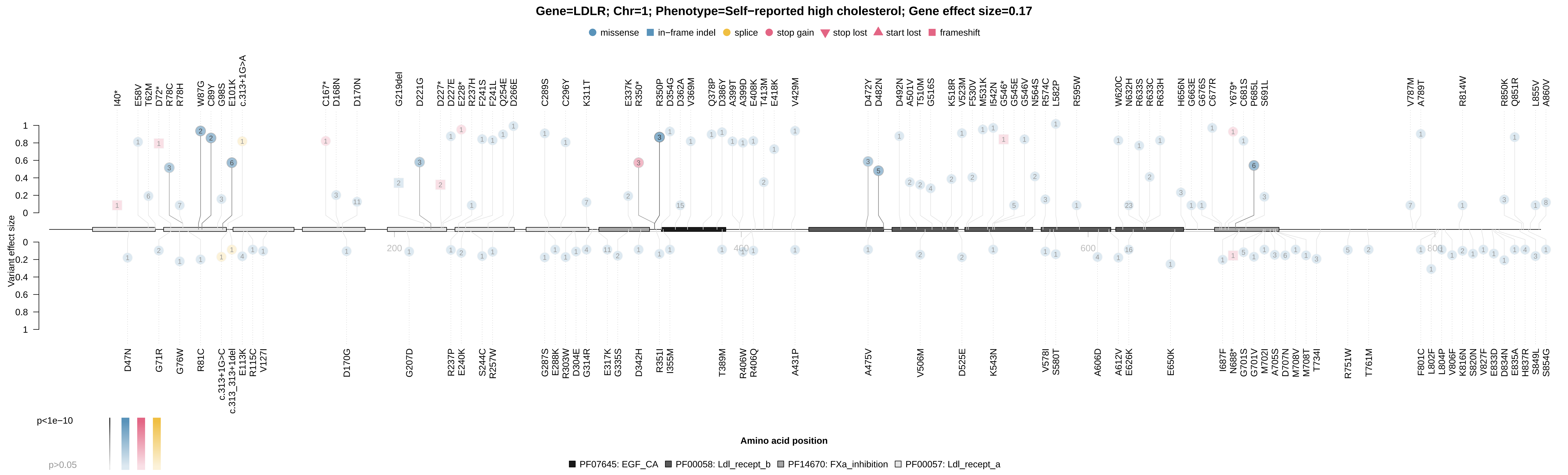

Gene=LDLR; Chr=1; Phenotype=Self−reported high cholesterol; Gene effect size=0.17
missense
in−frame indel
splice
stop gain
stop lost
start lost
frameshift
I40*
E58V
T62M
D72*
R78C
R78H
W87G
C89Y
G98S
E101K
c.313+1G>A
C167*
D168N
D170N
G219del
D221G
D227*
D227E
E228*
R237H
F241S
F241L
Q254E
D266E
C289S
C296Y
K311T
E337K
R350*
R350P
D354G
D362A
V369M
Q378P
D386Y
A399T
A399D
E408K
T413M
E418K
V429M
D472Y
D482N
D492N
A501V
T510M
G516S
K518R
V523M
F530V
M531K
I542N
G546*
G545E
G546V
N564S
R574C
L582P
R595W
W620C
N632H
R633S
R633C
R633H
H656N
G663E
G676S
C677R
Y679*
C681S
P685L
S691L
V787M
A789T
R814W
R850K
Q851R
L855V
A860V
Variant effect size
1
0.8
0.6
0.4
0.2
0
200
400
600
800
D47N
G71R
G76W
R81C
c.313+1G>C
c.313_313+1del
E113K
R115C
V127I
D170G
G207D
R237P
E240K
S244C
R257W
G287S
E288K
R303W
D304E
G314R
E317K
G335S
D342H
R351I
I355M
T389M
R406W
R406Q
A431P
A475V
V506M
D525E
K543N
V578I
S580T
A606D
A612V
E626K
E650K
I687F
N688*
G701S
G701V
M702I
A705S
D707N
M708V
M708T
T734I
R751W
T761M
F801C
L802F
L804P
V806F
K816N
S820N
V827F
E833D
D834N
E835A
H837R
S849L
S854G
p<1e−10
p>0.05
Amino acid position
PF07645: EGF_CA
PF00058: Ldl_recept_b
PF14670: FXa_inhibition
PF00057: Ldl_recept_a

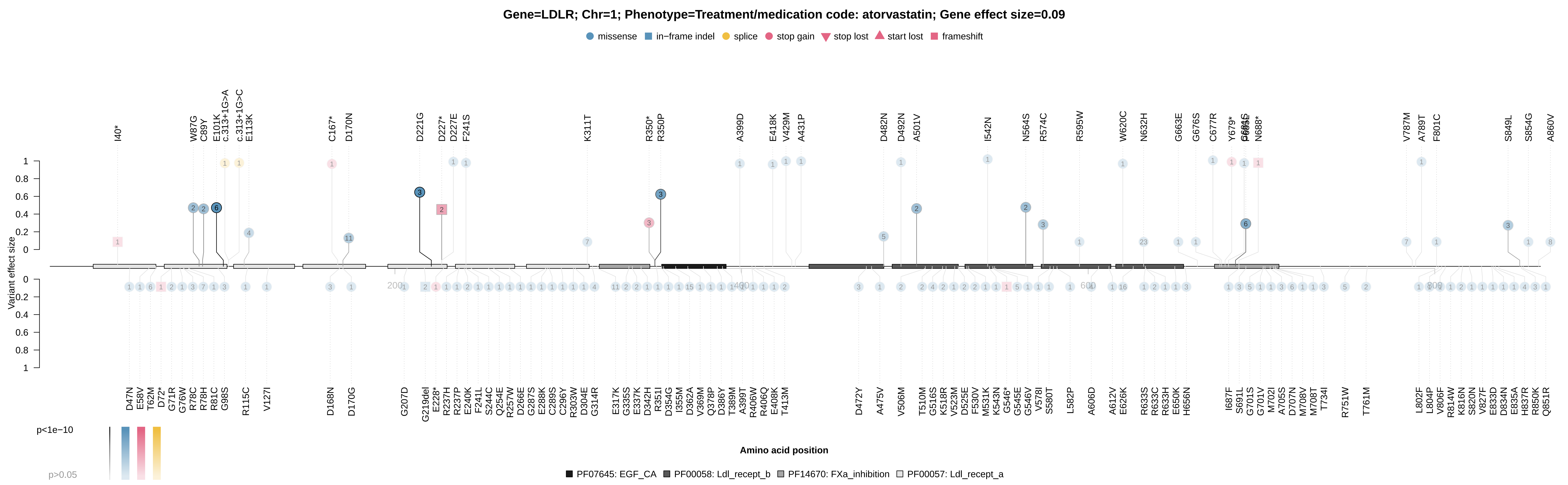

Gene=LDLR; Chr=1; Phenotype=Treatment/medication code: atorvastatin; Gene effect size=0.09
missense
in−frame indel
splice
stop gain
stop lost
start lost
frameshift
Variant effect size
Amino acid position
p<1e−10
p>0.05
PF07645: EGF_CA
PF00058: Ldl_recept_b
PF14670: FXa_inhibition
PF00057: Ldl_recept_a

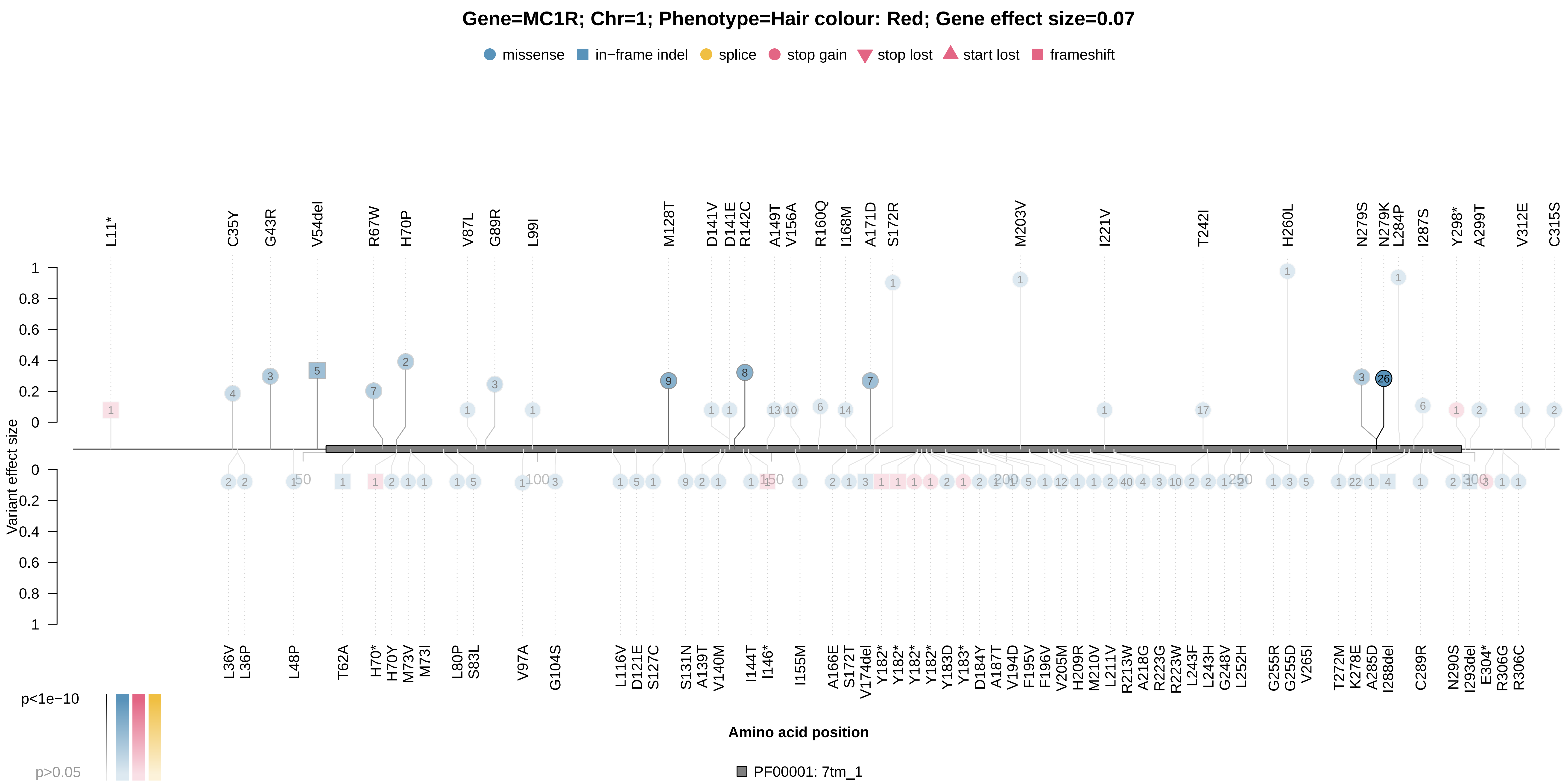
Gene=MC1R; Chr=1; Phenotype=Hair colour: Red; Gene effect size=0.07
missense
in−frame indel
splice
stop gain
stop lost
start lost
frameshift
L11*
C35Y
G43R
V54del
R67W
H70P
V87L
G89R
L99I
M128T
D141V
D141E
R142C
A149T
V156A
R160Q
I168M
A171D
S172R
M203V
I221V
T242I
H260L
N279S
N279K
L284P
I287S
Y298*
A299T
V312E
C315S
Variant effect size
L36V
L36P
L48P
T62A
H70*
H70Y
M73V
M73I
L80P
S83L
V97A
G104S
L116V
D121E
S127C
S131N
A139T
V140M
I144T
I146*
I155M
A166E
S172T
V174del
Y182*
Y182*
Y182*
Y182*
Y183D
Y183*
D184Y
A187T
V194D
F195V
F196V
V205M
H209R
M210V
L211V
R213W
A218G
R223G
R223W
L243F
L243H
G248V
L252H
G255R
G255D
V265I
T272M
K278E
A285D
I288del
C289R
N290S
I293del
E304*
R306G
R306C
p<1e−10
p>0.05
Amino acid position
PF00001: 7tm_1

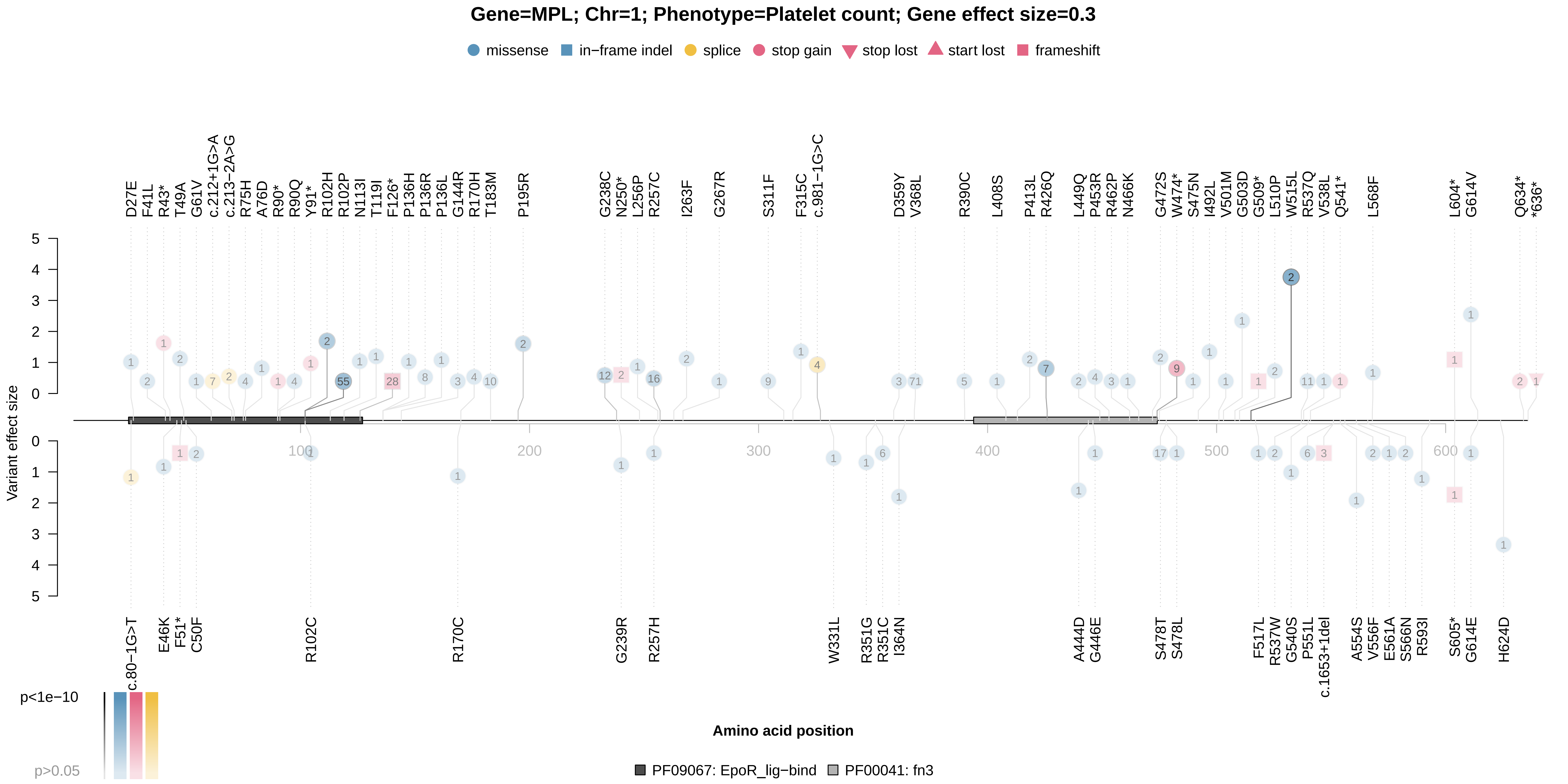
Gene=MPL; Chr=1; Phenotype=Platelet count; Gene effect size=0.3
missense
in−frame indel
splice
stop gain
stop lost
start lost
frameshift
Variant effect size
Amino acid position
p<1e−10
p>0.05
PF09067: EpoR_lig−bind
PF00041: fn3

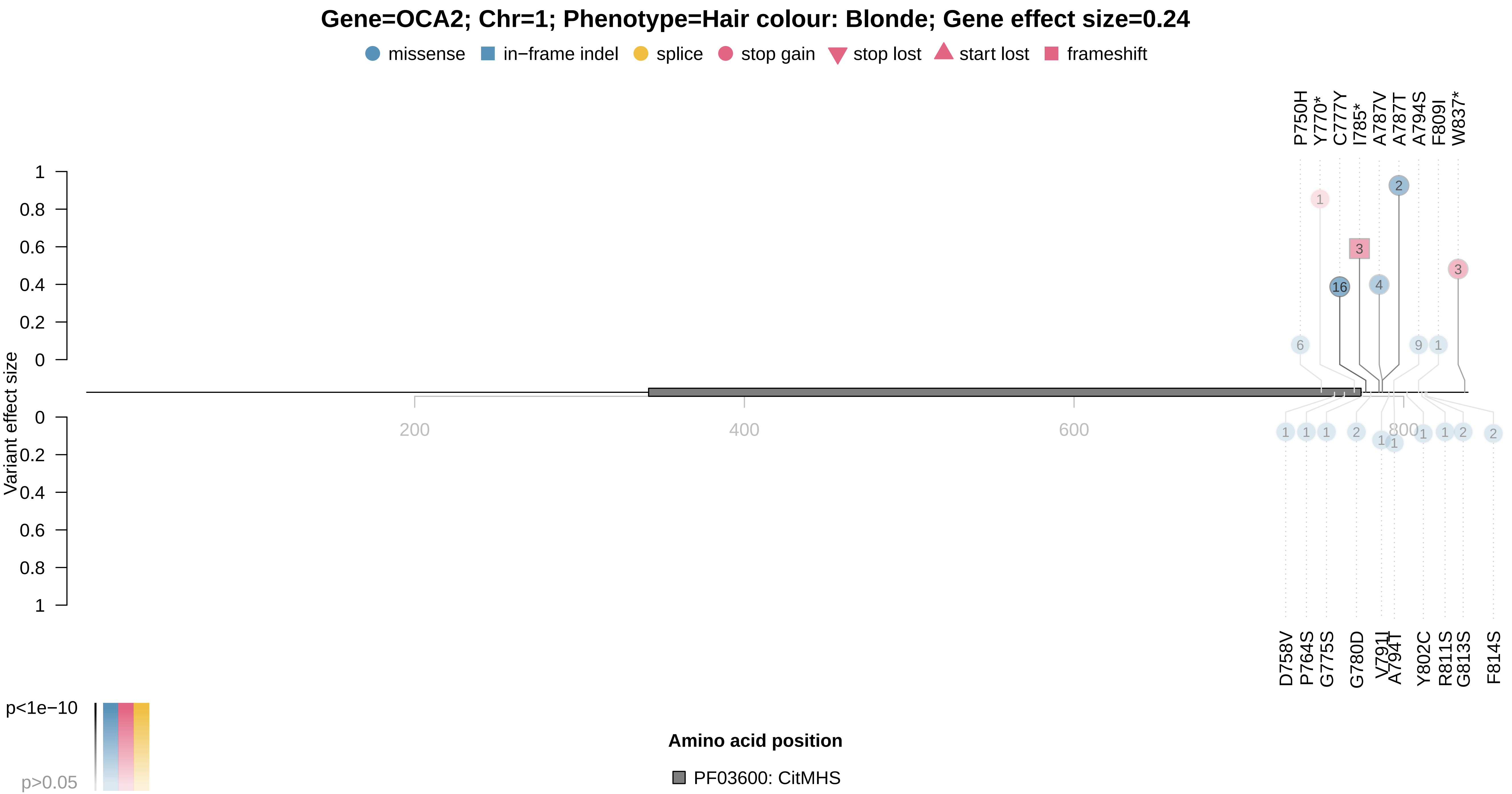
Gene=OCA2; Chr=1; Phenotype=Hair colour: Blonde; Gene effect size=0.24
missense
in−frame indel
splice
stop gain
stop lost
start lost
frameshift
P750H
Y770*
C777Y
I785*
A787V
A787T
A794S
F809I
W837*
Variant effect size
200
400
600
800
D758V
P764S
G775S
G780D
V791I
A794T
Y802C
R811S
G813S
F814S
p<1e−10
p>0.05
Amino acid position
PF03600: CitMHS

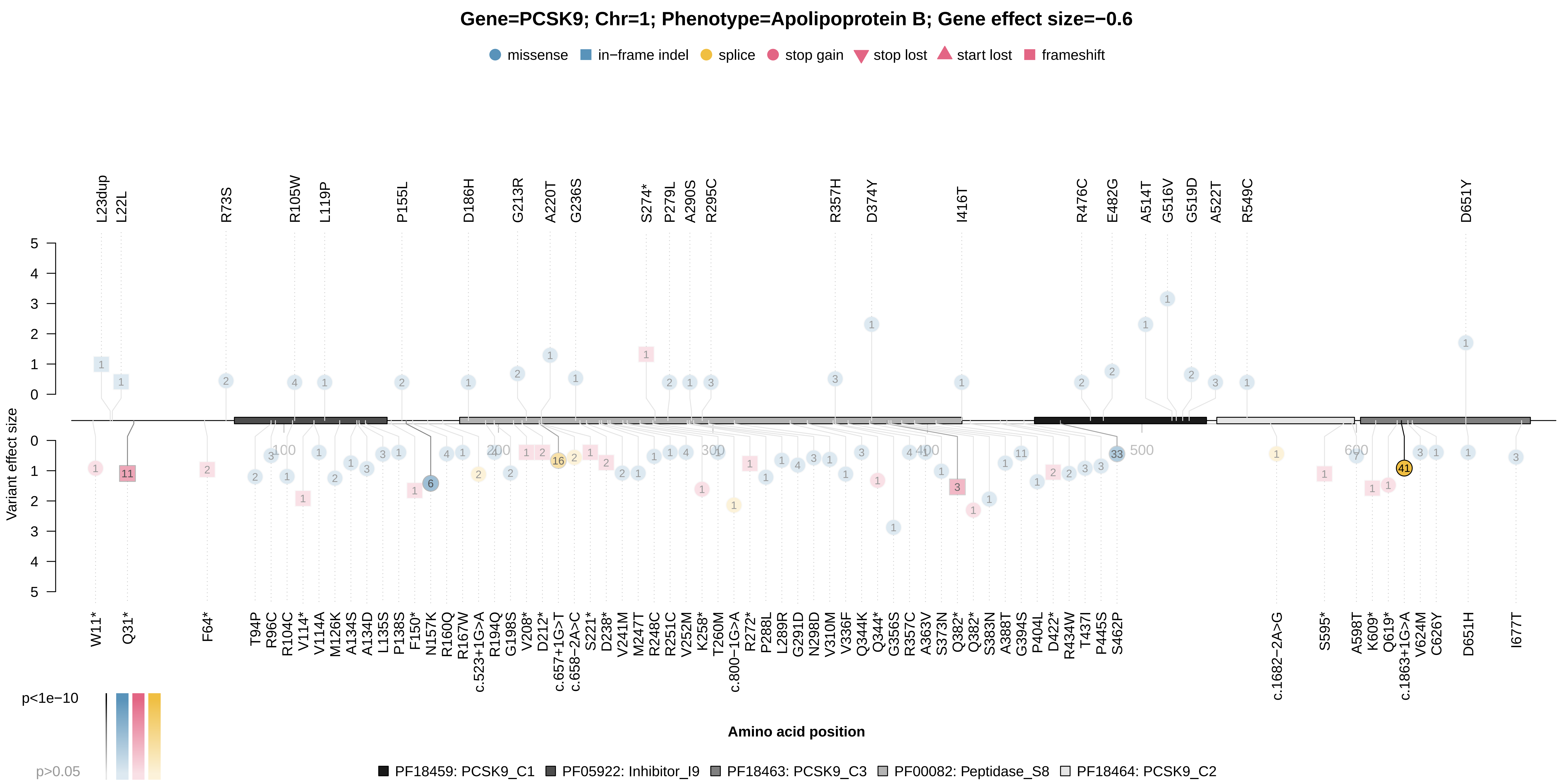
Gene=PCSK9; Chr=1; Phenotype=Apolipoprotein B; Gene effect size=−0.6
missense
in−frame indel
splice
stop gain
stop lost
start lost
frameshift
L23dup
L22L
R73S
R105W
L119P
P155L
D186H
G213R
A220T
G236S
S274*
P279L
A290S
R295C
R357H
D374Y
I416T
R476C
E482G
A514T
G516V
G519D
A522T
R549C
D651Y
Variant effect size
100
200
300
400
500
600
W11*
Q31*
F64*
T94P
R96C
R104C
V114*
V114A
M126K
A134S
A134D
L135S
P138S
F150*
N157K
R160Q
R167W
c.523+1G>A
R194Q
G198S
V208*
D212*
c.657+1G>T
c.658−2A>C
S221*
D238*
V241M
M247T
R248C
R251C
V252M
K258*
T260M
c.800−1G>A
R272*
P288L
L289R
G291D
N298D
V310M
V336F
Q344K
Q344*
G356S
R357C
A363V
S373N
Q382*
Q382*
S383N
A388T
G394S
P404L
D422*
R434W
T437I
P445S
S462P
c.1682−2A>G
S595*
A598T
K609*
Q619*
c.1863+1G>A
V624M
C626Y
D651H
I677T
p<1e−10
p>0.05
Amino acid position
PF18459: PCSK9_C1
PF05922: Inhibitor_I9
PF18463: PCSK9_C3
PF00082: Peptidase_S8
PF18464: PCSK9_C2

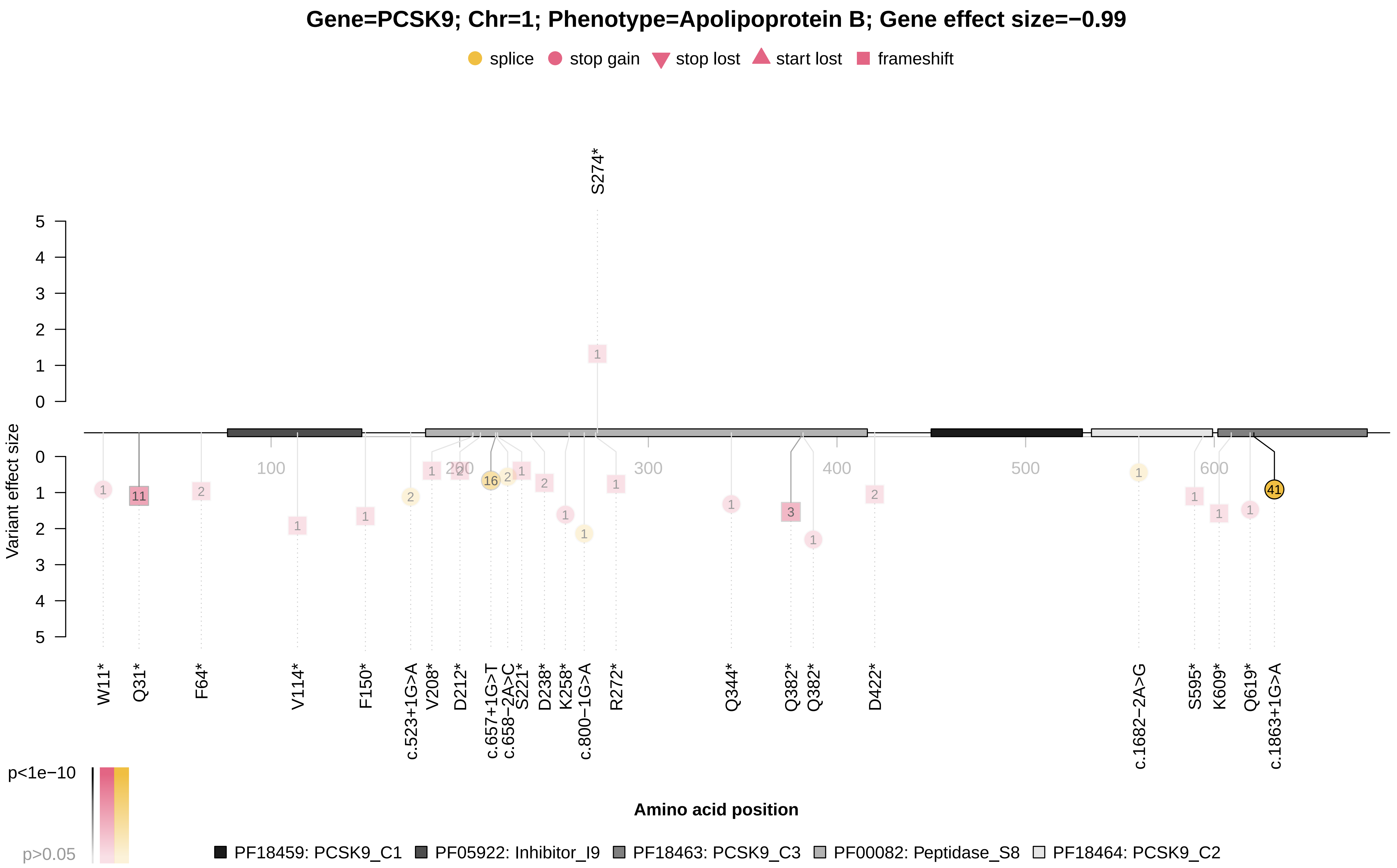

Gene=PCSK9; Chr=1; Phenotype=Apolipoprotein B; Gene effect size=−0.99
splice
stop gain
stop lost
start lost
frameshift
Variant effect size
Amino acid position
100
200
300
400
500
600
S274*
W11*
Q31*
F64*
V114*
F150*
c.523+1G>A
V208*
D212*
c.657+1G>T
c.658−2A>C
S221*
D238*
K258*
c.800−1G>A
R272*
Q344*
Q382*
Q382*
D422*
c.1682−2A>G
S595*
K609*
Q619*
c.1863+1G>A
p<1e−10
p>0.05
PF18459: PCSK9_C1
PF05922: Inhibitor_I9
PF18463: PCSK9_C3
PF00082: Peptidase_S8
PF18464: PCSK9_C2

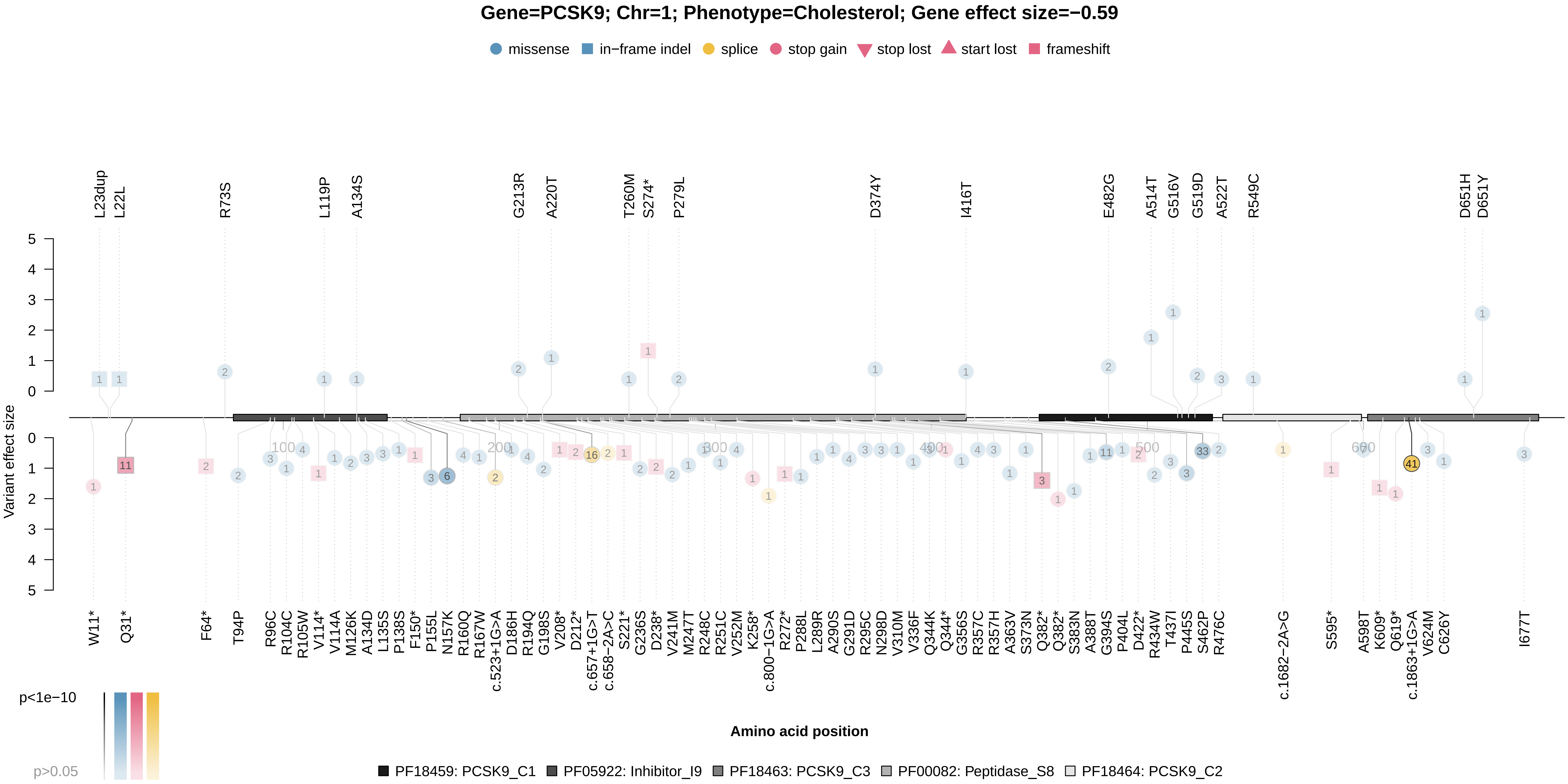
Gene=PCSK9; Chr=1; Phenotype=Cholesterol; Gene effect size=−0.59
missense
in−frame indel
splice
stop gain
stop lost
start lost
frameshift
L23dup
L22L
R73S
L119P
A134S
G213R
A220T
T260M
S274*
P279L
D374Y
I416T
E482G
A514T
G516V
G519D
A522T
R549C
D651H
D651Y
Variant effect size
W11*
Q31*
F64*
T94P
R96C
R104C
R105W
V114*
V114A
M126K
A134D
L135S
P138S
F150*
P155L
N157K
R160Q
R167W
c.523+1G>A
D186H
R194Q
G198S
V208*
D212*
c.657+1G>T
c.658−2A>C
S221*
G236S
D238*
V241M
M247T
R248C
R251C
V252M
K258*
c.800−1G>A
R272*
P288L
L289R
A290S
G291D
R295C
N298D
V310M
V336F
Q344K
Q344*
G356S
R357C
R357H
A363V
S373N
Q382*
Q382*
S383N
A388T
G394S
P404L
D422*
R434W
T437I
P445S
S462P
R476C
c.1682−2A>G
S595*
A598T
K609*
Q619*
c.1863+1G>A
V624M
C626Y
I677T
Amino acid position
p<1e−10
p>0.05
PF18459: PCSK9_C1
PF05922: Inhibitor_I9
PF18463: PCSK9_C3
PF00082: Peptidase_S8
PF18464: PCSK9_C2

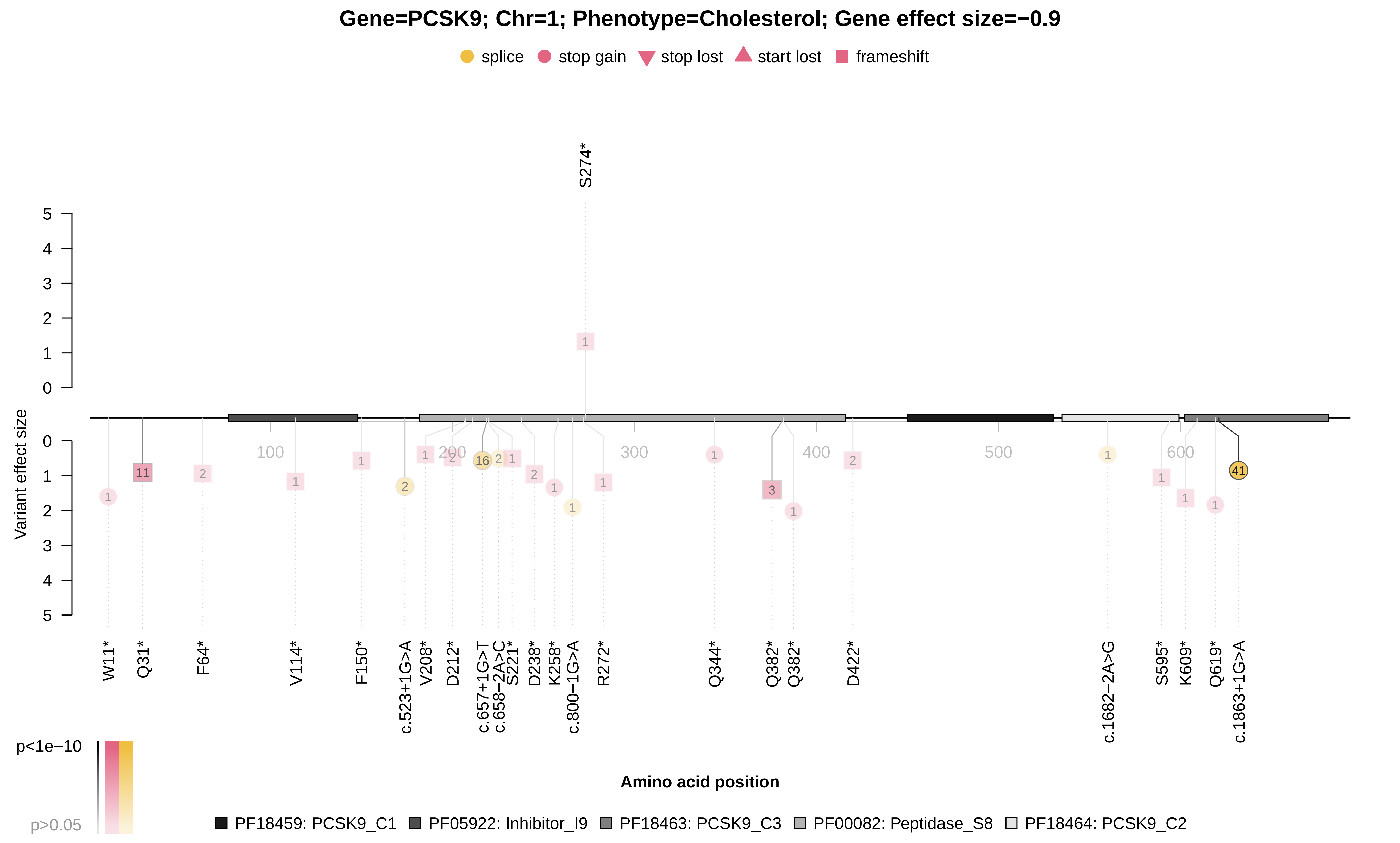

Gene=PCSK9; Chr=1; Phenotype=Cholesterol; Gene effect size=−0.9
splice
stop gain
stop lost
start lost
frameshift
S274*
Variant effect size
5
4
3
2
1
0
100
200
300
400
500
600
W11*
Q31*
F64*
V114*
F150*
c.523+1G>A
V208*
D212*
c.657+1G>T
c.658−2A>C
S221*
D238*
K258*
c.800−1G>A
R272*
Q344*
Q382*
Q382*
D422*
c.1682−2A>G
S595*
K609*
Q619*
c.1863+1G>A
p<1e−10
p>0.05
Amino acid position
PF18459: PCSK9_C1
PF05922: Inhibitor_I9
PF18463: PCSK9_C3
PF00082: Peptidase_S8
PF18464: PCSK9_C2

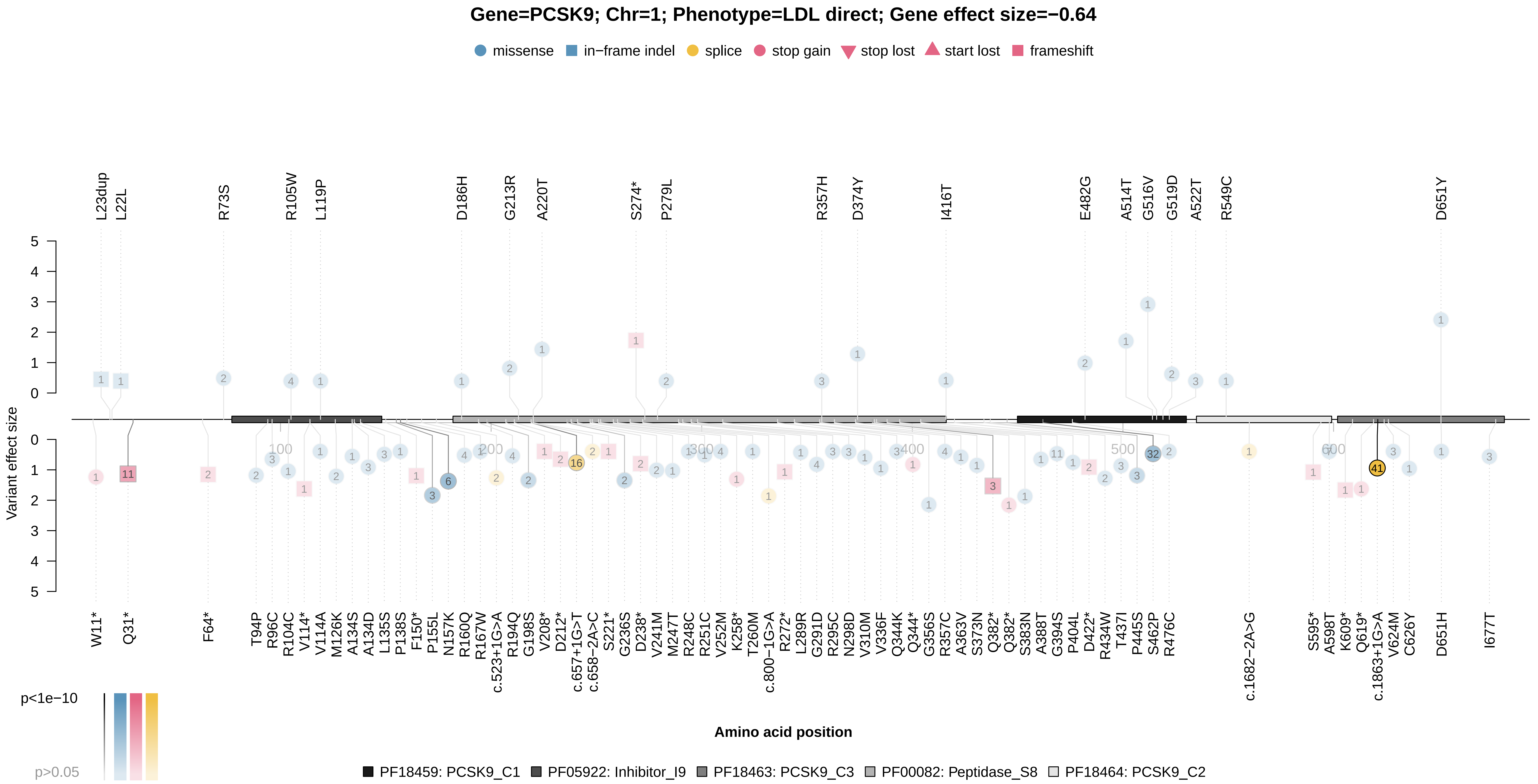
Gene=PCSK9; Chr=1; Phenotype=LDL direct; Gene effect size=−0.64
missense
in−frame indel
splice
stop gain
stop lost
start lost
frameshift
Variant effect size
Amino acid position
100
200
300
400
500
600
L23dup
L22L
R73S
R105W
L119P
D186H
G213R
A220T
S274*
P279L
R357H
D374Y
I416T
E482G
A514T
G516V
G519D
A522T
R549C
D651Y
W11*
Q31*
F64*
T94P
R96C
R104C
V114*
V114A
M126K
A134S
A134D
L135S
P138S
F150*
P155L
N157K
R160Q
R167W
c.523+1G>A
R194Q
G198S
V208*
D212*
c.657+1G>T
c.658−2A>C
S221*
G236S
D238*
V241M
M247T
R248C
R251C
V252M
K258*
T260M
c.800−1G>A
R272*
L289R
G291D
R295C
N298D
V310M
V336F
Q344K
Q344*
G356S
R357C
A363V
S373N
Q382*
Q382*
S383N
A388T
G394S
P404L
D422*
R434W
T437I
P445S
S462P
R476C
c.1682−2A>G
S595*
A598T
K609*
Q619*
c.1863+1G>A
V624M
C626Y
D651H
I677T
p<1e−10
p>0.05
PF18459: PCSK9_C1
PF05922: Inhibitor_I9
PF18463: PCSK9_C3
PF00082: Peptidase_S8
PF18464: PCSK9_C2

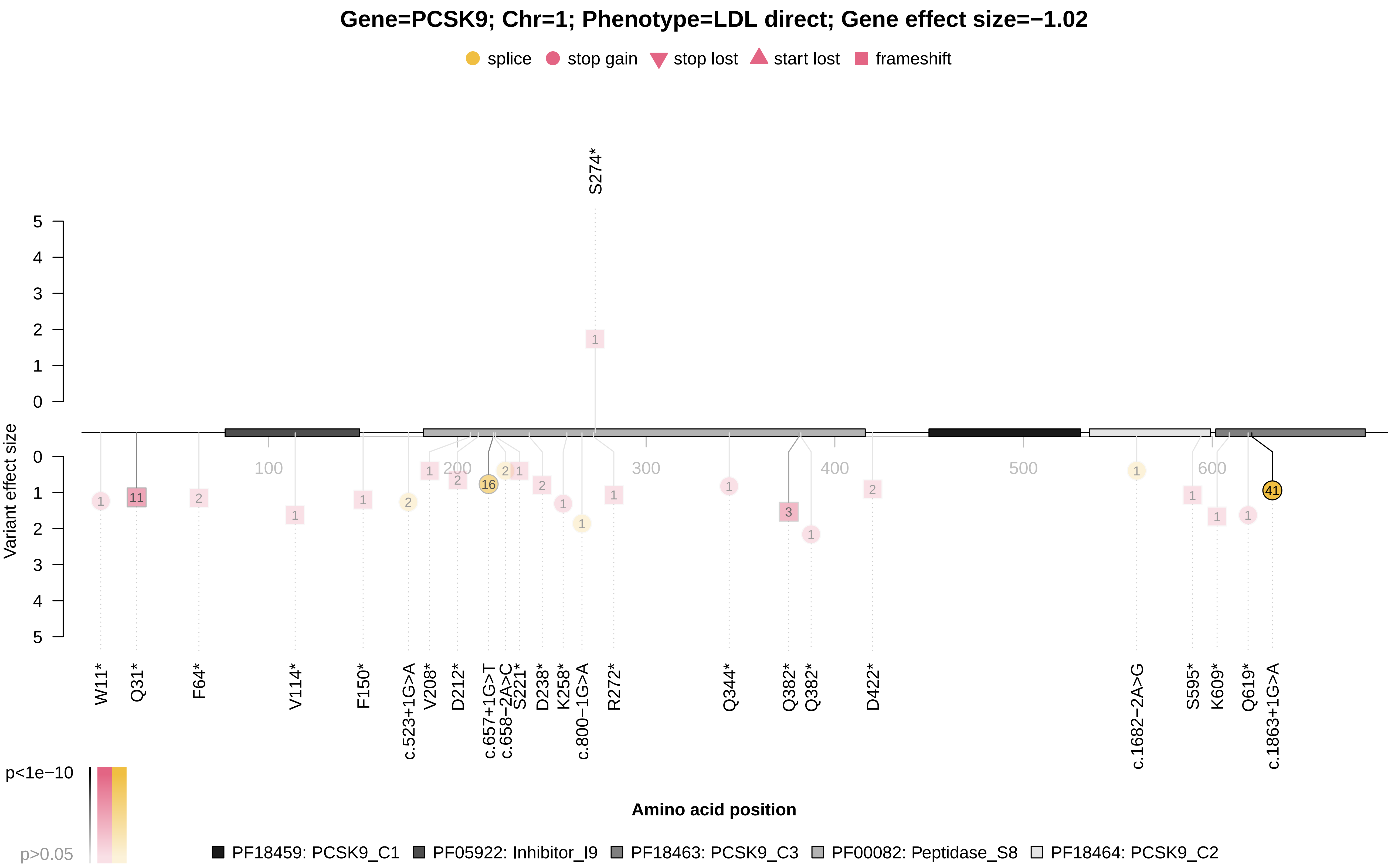

Gene=PCSK9; Chr=1; Phenotype=LDL direct; Gene effect size=−1.02
splice
stop gain
stop lost
start lost
frameshift
S274*
Variant effect size
W11*
Q31*
F64*
V114*
F150*
c.523+1G>A
V208*
D212*
c.657+1G>T
c.658−2A>C
S221*
D238*
K258*
c.800−1G>A
R272*
Q344*
Q382*
Q382*
D422*
c.1682−2A>G
S595*
K609*
Q619*
c.1863+1G>A
p<1e−10
p>0.05
Amino acid position
PF18459: PCSK9_C1
PF05922: Inhibitor_I9
PF18463: PCSK9_C3
PF00082: Peptidase_S8
PF18464: PCSK9_C2

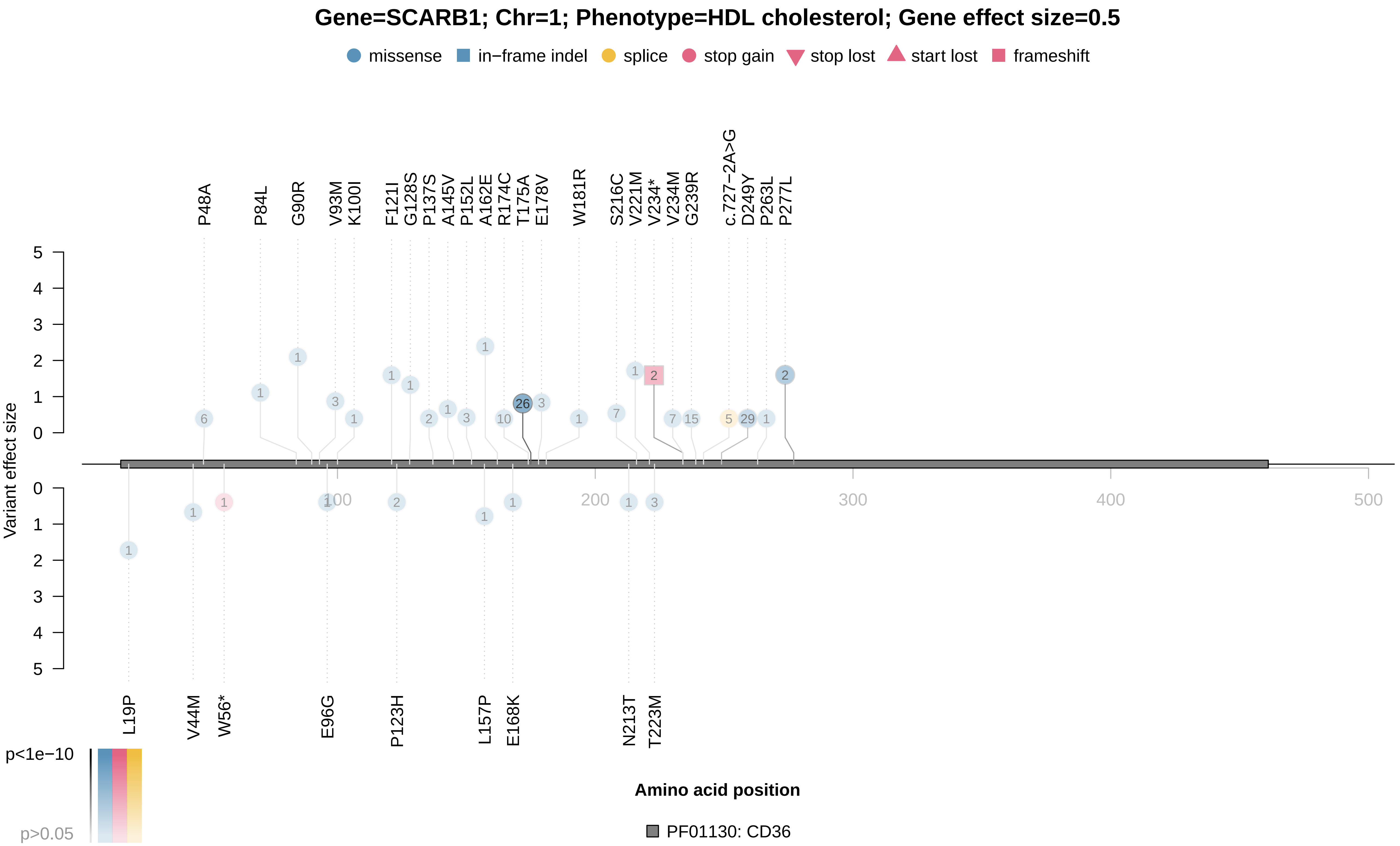

Gene=SCARB1; Chr=1; Phenotype=HDL cholesterol; Gene effect size=0.5
missense
in−frame indel
splice
stop gain
stop lost
start lost
frameshift
P48A
P84L
G90R
V93M
K100I
F121I
G128S
P137S
A145V
P152L
A162E
R174C
T175A
E178V
W181R
S216C
V221M
V234*
V234M
G239R
c.727−2A>G
D249Y
P263L
P277L
Variant effect size
L19P
V44M
W56*
E96G
P123H
L157P
E168K
N213T
T223M
p<1e−10
p>0.05
Amino acid position
PF01130: CD36

Gene=SEC23B; Chr=2; Phenotype=Red blood cell distribution width; Gene effect size=0.34

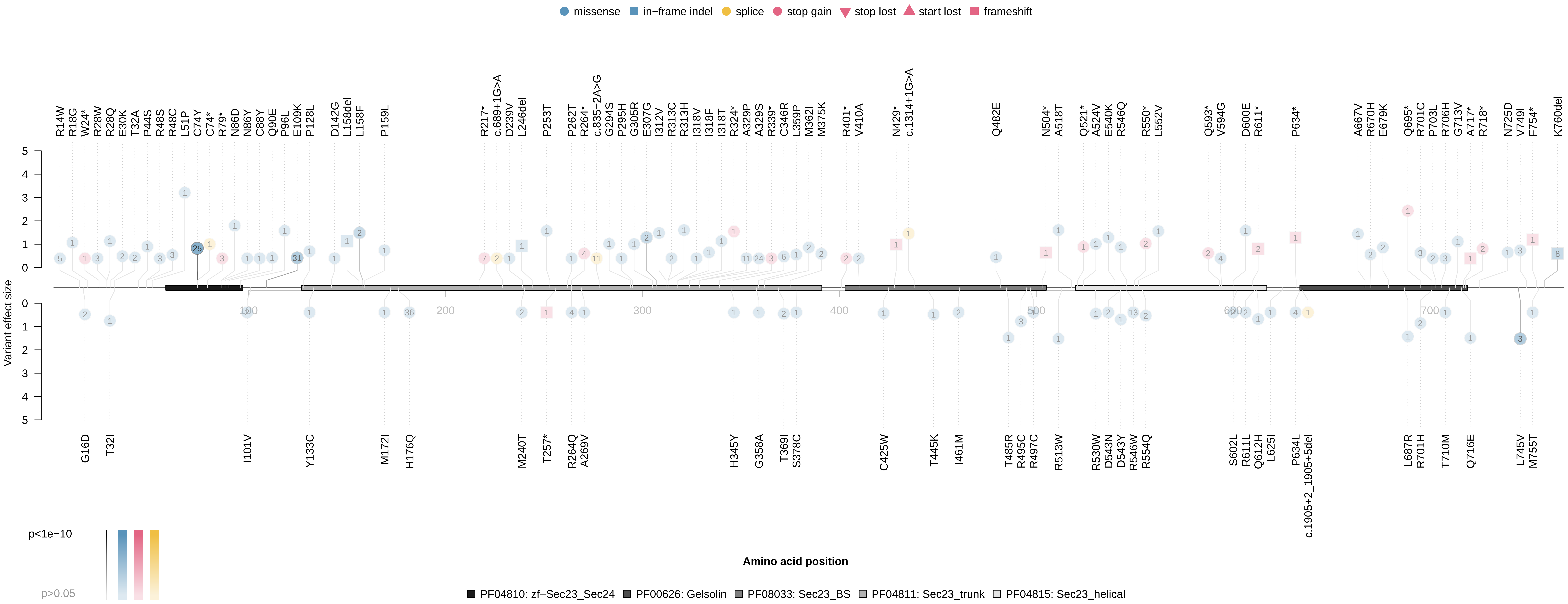

Gene=SHBG; Chr=1; Phenotype=SHBG; Gene effect size=−0.9

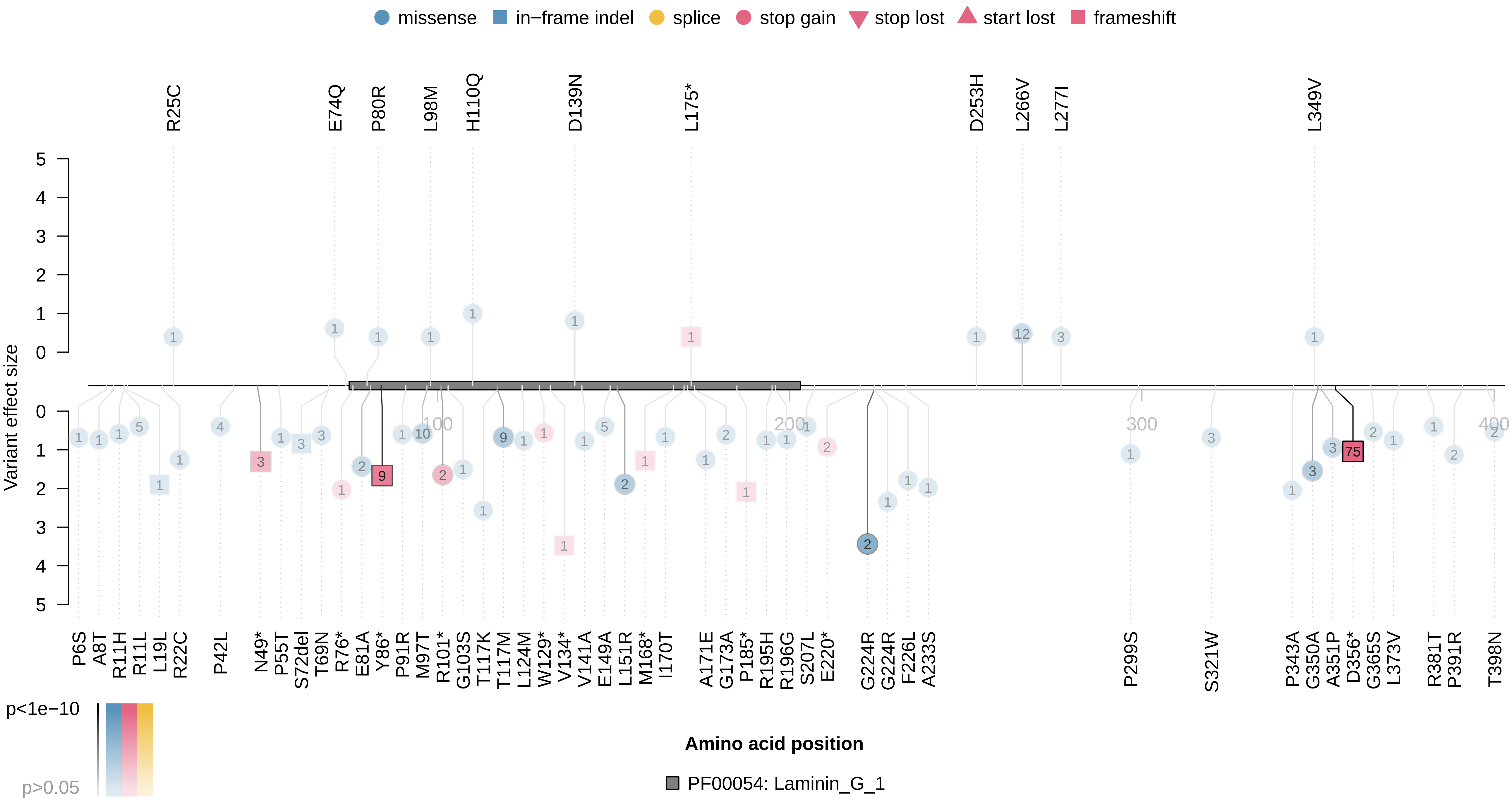

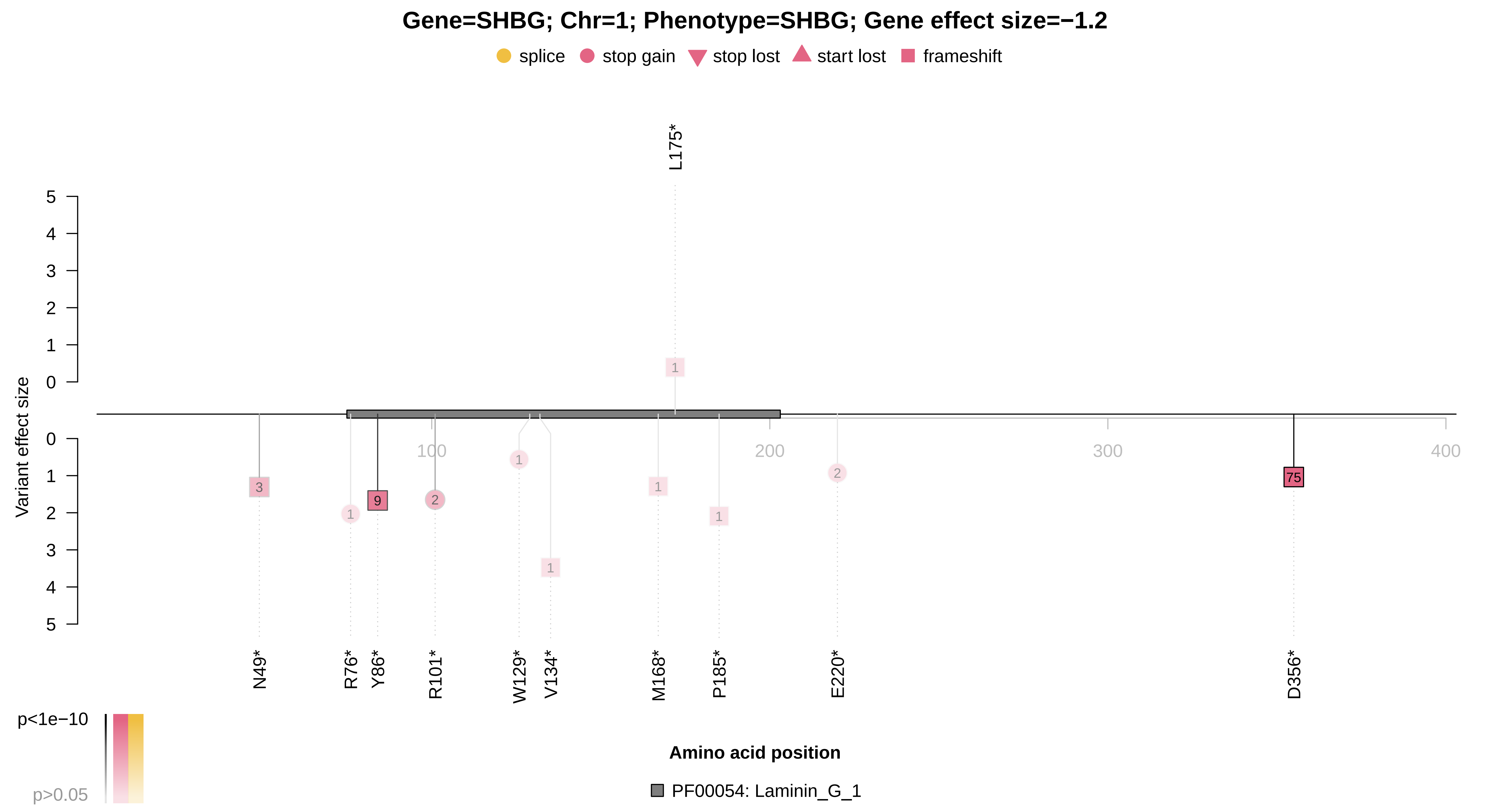

Gene=SHBG; Chr=1; Phenotype=SHBG; Gene effect size=−1.2
splice
stop gain
stop lost
start lost
frameshift
L175*
Variant effect size
0
1
2
3
4
5
100
200
300
400
3
1
9
2
1
1
1
1
2
75
N49*
R76*
Y86*
R101*
W129*
V134*
M168*
P185*
E220*
D356*
p<1e−10
p>0.05
Amino acid position
PF00054: Laminin_G_1

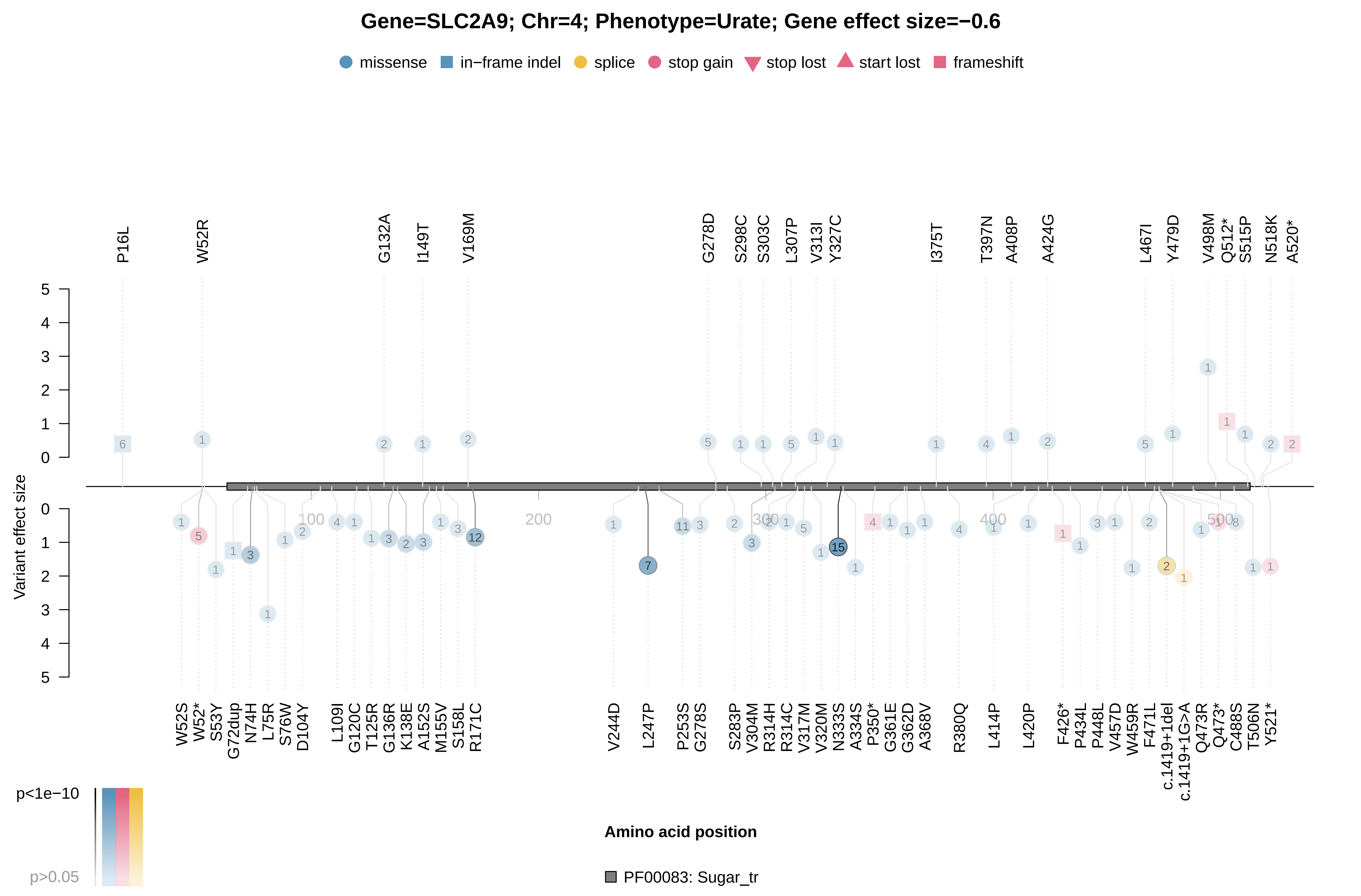
Gene=SLC2A9; Chr=4; Phenotype=Urate; Gene effect size=−0.6
missense
in−frame indel
splice
stop gain
stop lost
start lost
frameshift
P16L
W52R
G132A
I149T
V169M
G278D
S298C
S303C
L307P
V313I
Y327C
I375T
T397N
A408P
A424G
L467I
Y479D
V498M
Q512*
S515P
N518K
A520*
Variant effect size
100
200
300
400
500
W52S
W52*
S53Y
G72dup
N74H
L75R
S76W
D104Y
L109I
G120C
T125R
G136R
K138E
A152S
M155V
S158L
R171C
V244D
L247P
P253S
G278S
S283P
V304M
R314H
R314C
V317M
V320M
N333S
A334S
P350*
G361E
G362D
A368V
R380Q
L414P
L420P
F426*
P434L
P448L
V457D
W459R
F471L
c.1419+1del
c.1419+1G>A
Q473R
Q473*
C488S
T506N
Y521*
p<1e−10
p>0.05
Amino acid position
PF00083: Sugar_tr

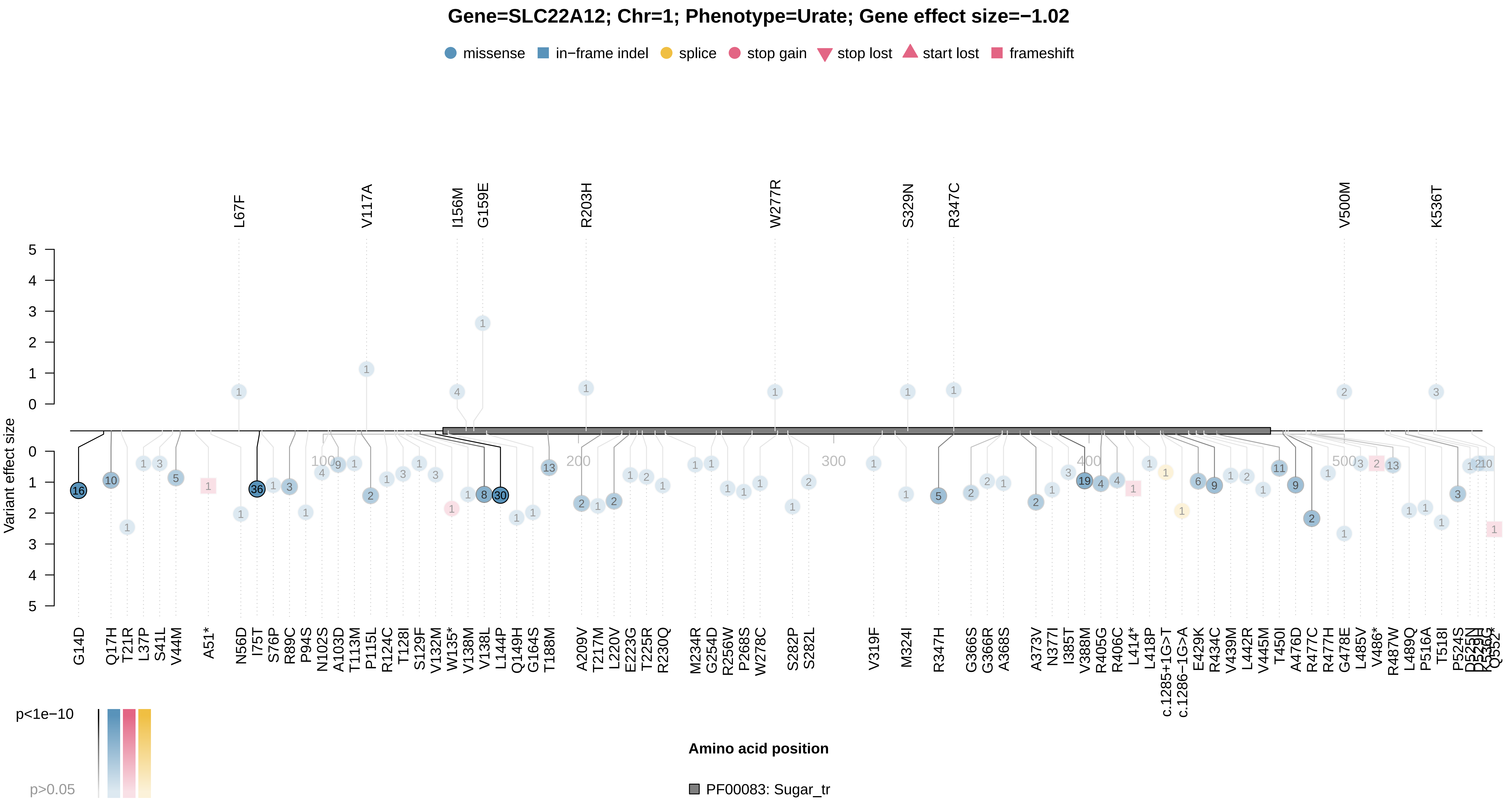

Gene=SLC22A12; Chr=1; Phenotype=Urate; Gene effect size=−1.02
missense
in−frame indel
splice
stop gain
stop lost
start lost
frameshift
Variant effect size
Amino acid position
PF00083: Sugar_tr
p<1e−10
p>0.05
L67F
V117A
I156M
G159E
R203H
W277R
S329N
R347C
V500M
K536T
G14D
Q17H
T21R
L37P
S41L
V44M
A51*
N56D
I75T
S76P
R89C
P94S
N102S
A103D
T113M
P115L
R124C
T128I
S129F
V132M
W135*
V138M
V138L
L144P
Q149H
G164S
T188M
A209V
T217M
L220V
E223G
T225R
R230Q
M234R
G254D
R256W
P268S
W278C
S282P
S282L
V319F
M324I
R347H
G366S
G366R
A368S
A373V
N377I
I385T
V388M
R405G
R406C
L414*
L418P
c.1285+1G>T
c.1286−1G>A
E429K
R434C
V439M
L442R
V445M
T450I
A476D
R477C
R477H
G478E
L485V
V486*
R487W
L489Q
P516A
T518I
P524S

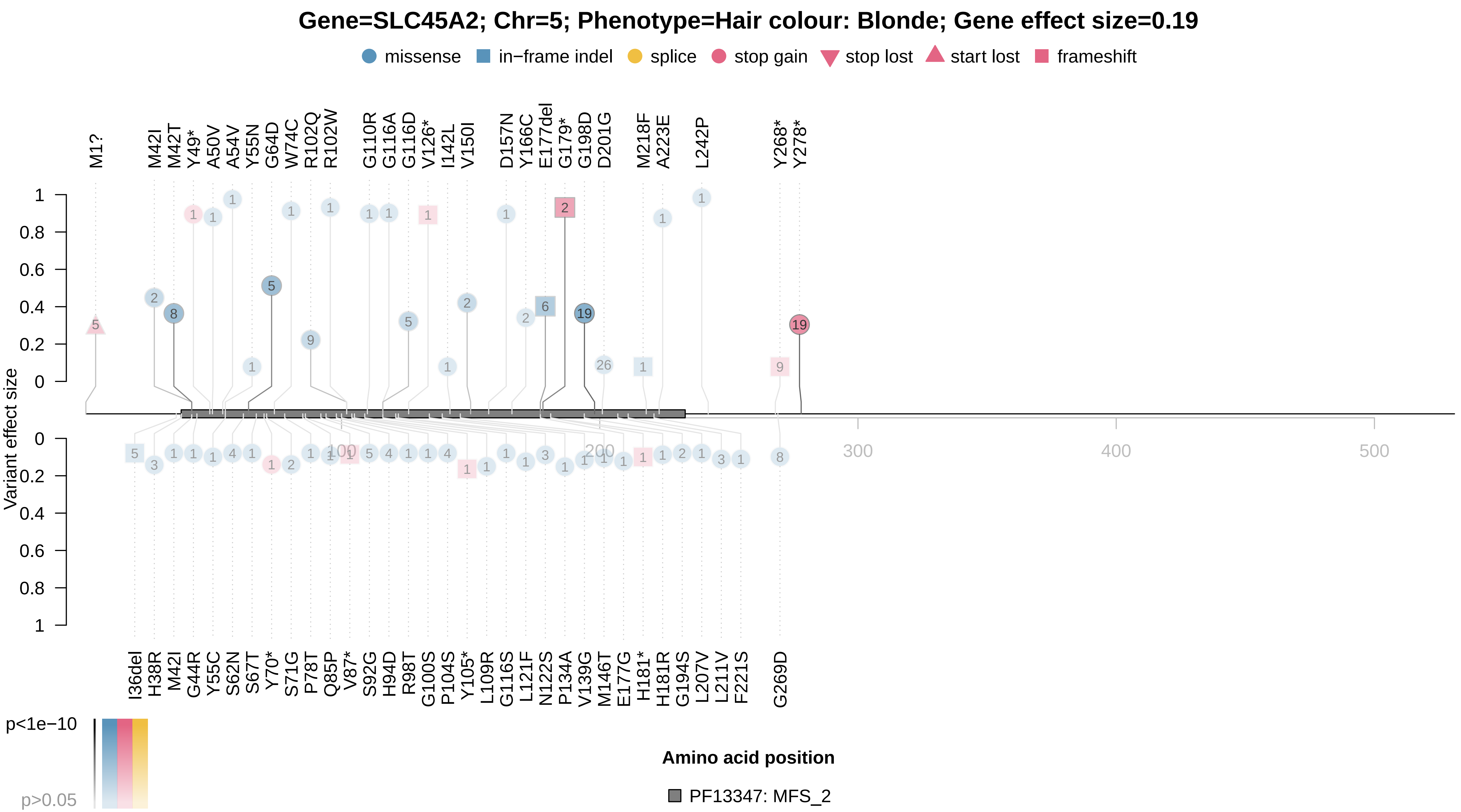
Gene=SLC45A2; Chr=5; Phenotype=Hair colour: Blonde; Gene effect size=0.19
missense
in−frame indel
splice
stop gain
stop lost
start lost
frameshift
M1?
M42I
M42T
Y49*
A50V
A54V
Y55N
G64D
W74C
R102Q
R102W
G110R
G116A
G116D
V126*
I142L
V150I
D157N
Y166C
E177del
G179*
G198D
D201G
M218F
A223E
L242P
Y268*
Y278*
Variant effect size
I36del
H38R
M42I
G44R
Y55C
S62N
S67T
Y70*
S71G
P78T
Q85P
V87*
S92G
H94D
R98T
G100S
P104S
Y105*
L109R
G116S
L121F
N122S
P134A
V139G
M146T
E177G
H181*
H181R
G194S
L207V
L211V
F221S
G269D
p<1e−10
p>0.05
Amino acid position
PF13347: MFS_2

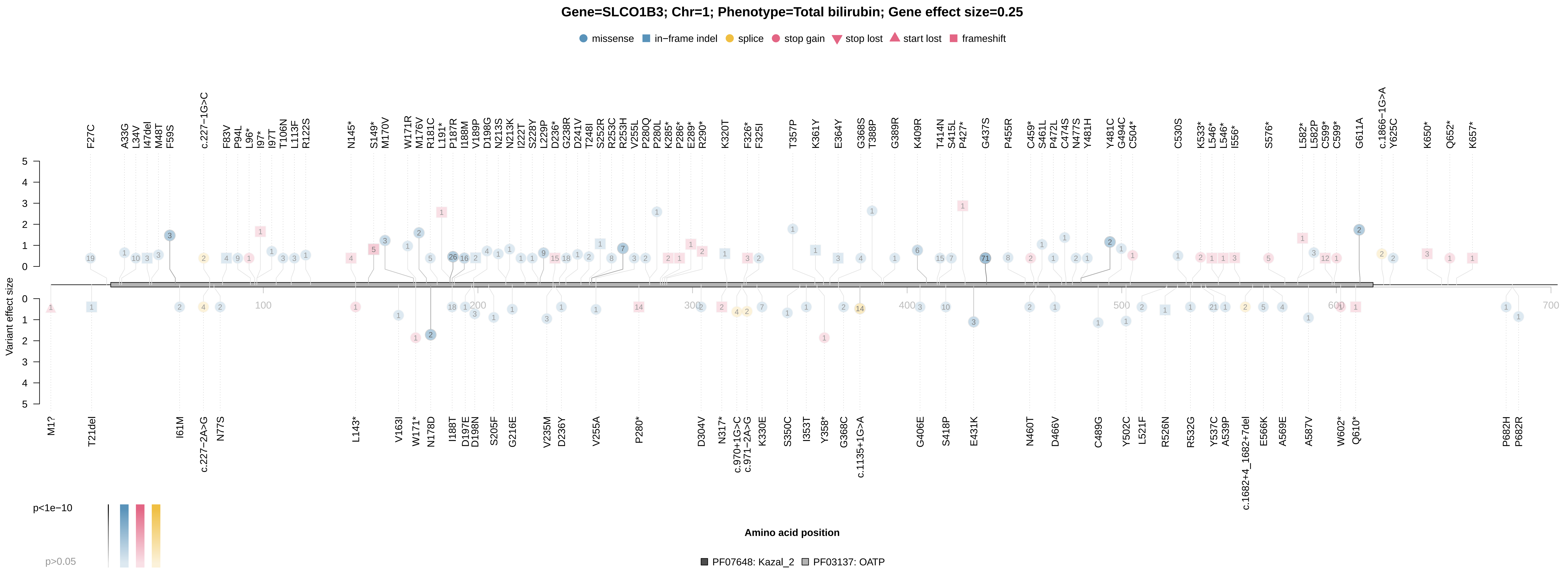

Gene=SLCO1B3; Chr=1; Phenotype=Total bilirubin; Gene effect size=0.25
missense
in−frame indel
splice
stop gain
stop lost
start lost
frameshift
F27C
A33G
L34V
I47del
M48T
F59S
c.227−1G>C
F83V
P94L
L96*
I97*
I97T
T106N
L113F
R122S
N145*
S149*
M170V
W171R
M176V
R181C
L191*
P187R
I188M
V189P
D198G
N213S
N213K
I222T
S228Y
L229P
D236*
G238R
D241V
T248I
S252R
R253C
R253H
V255L
P280Q
P280L
K285*
P286*
E289*
R290*
K320T
F326*
F325I
T357P
K361Y
E364Y
G368S
T388P
G389R
K409R
T414N
S415L
P427*
G437S
P455R
C459*
S461L
P472L
C474S
N477S
Y481H
Y481C
G494C
C504*
C530S
K533*
L546*
L546*
I556*
S576*
L582*
L582P
C599*
C599*
G611A
c.1866−1G>A
Y625C
K650*
Q652*
K657*
Variant effect size
100
200
300
400
500
600
700
M1?
T21del
I61M
c.227−2A>G
N77S
L143*
V163I
W171*
N178D
I188T
D197E
D198N
S205F
G216E
V235M
D236Y
V255A
P280*
D304V
N317*
c.970+1G>C
c.971−2A>G
K330E
S350C
I353T
Y358*
G368C
c.1135+1G>A
G406E
S418P
E431K
N460T
D466V
C489G
Y502C
L521F
R526N
R532G
Y537C
A539P
c.1682+4_1682+7del
E566K
A569E
A587V
W602*
Q610*
P682H
P682R
p<1e−10
p>0.05
Amino acid position
PF07648: Kazal_2
PF03137: OATP

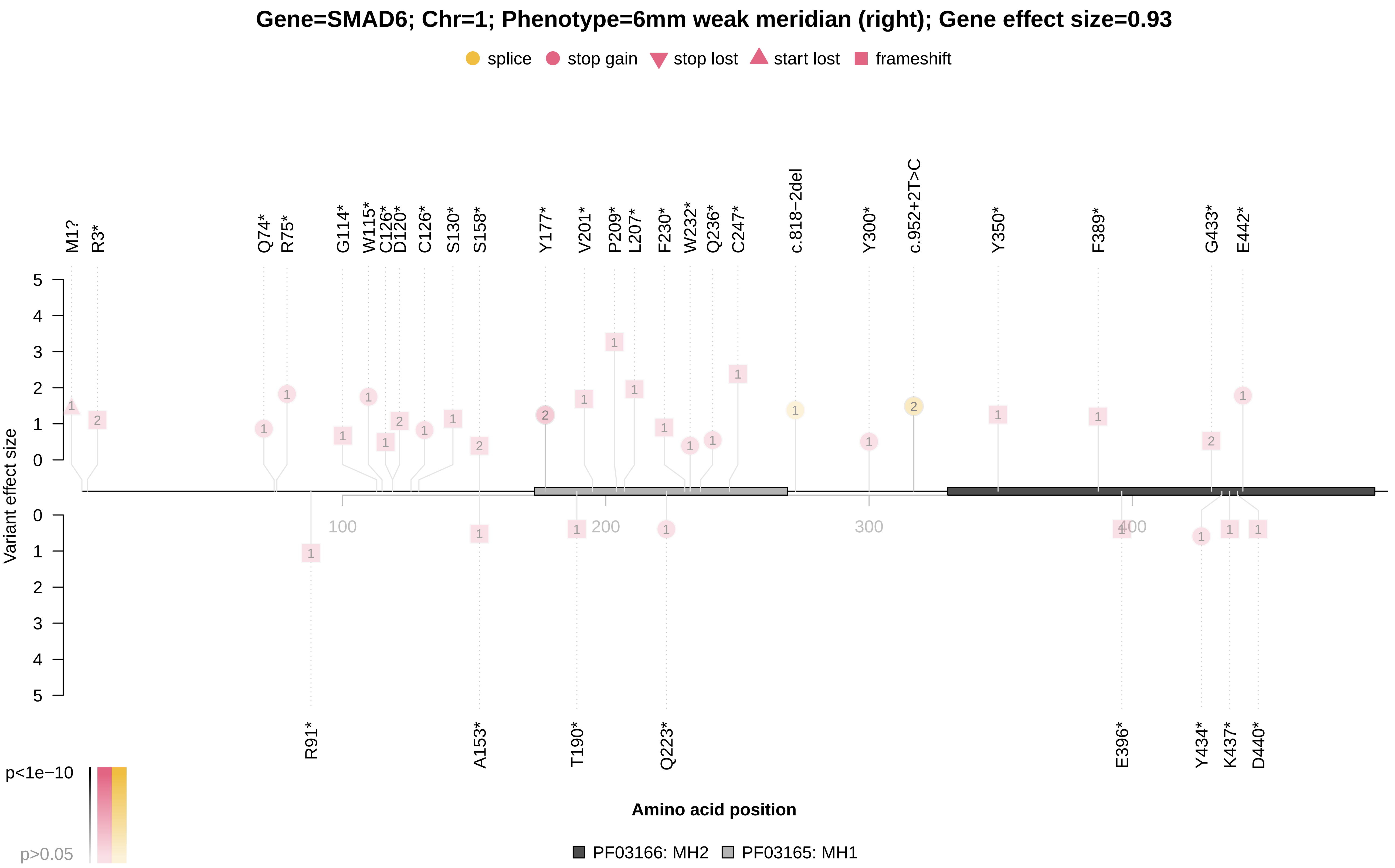

Gene=SMAD6; Chr=1; Phenotype=6mm weak meridian (right); Gene effect size=0.93
splice
stop gain
stop lost
start lost
frameshift
M1?
R3*
Q74*
R75*
G114*
W115*
C126*
D120*
C126*
S130*
S158*
Y177*
V201*
P209*
L207*
F230*
W232*
Q236*
C247*
c.818−2del
Y300*
c.952+2T>C
Y350*
F389*
G433*
E442*
R91*
A153*
T190*
Q223*
E396*
Y434*
K437*
D440*
Variant effect size
100
200
300
400
Amino acid position
p<1e−10
p>0.05
PF03166: MH2
PF03165: MH1

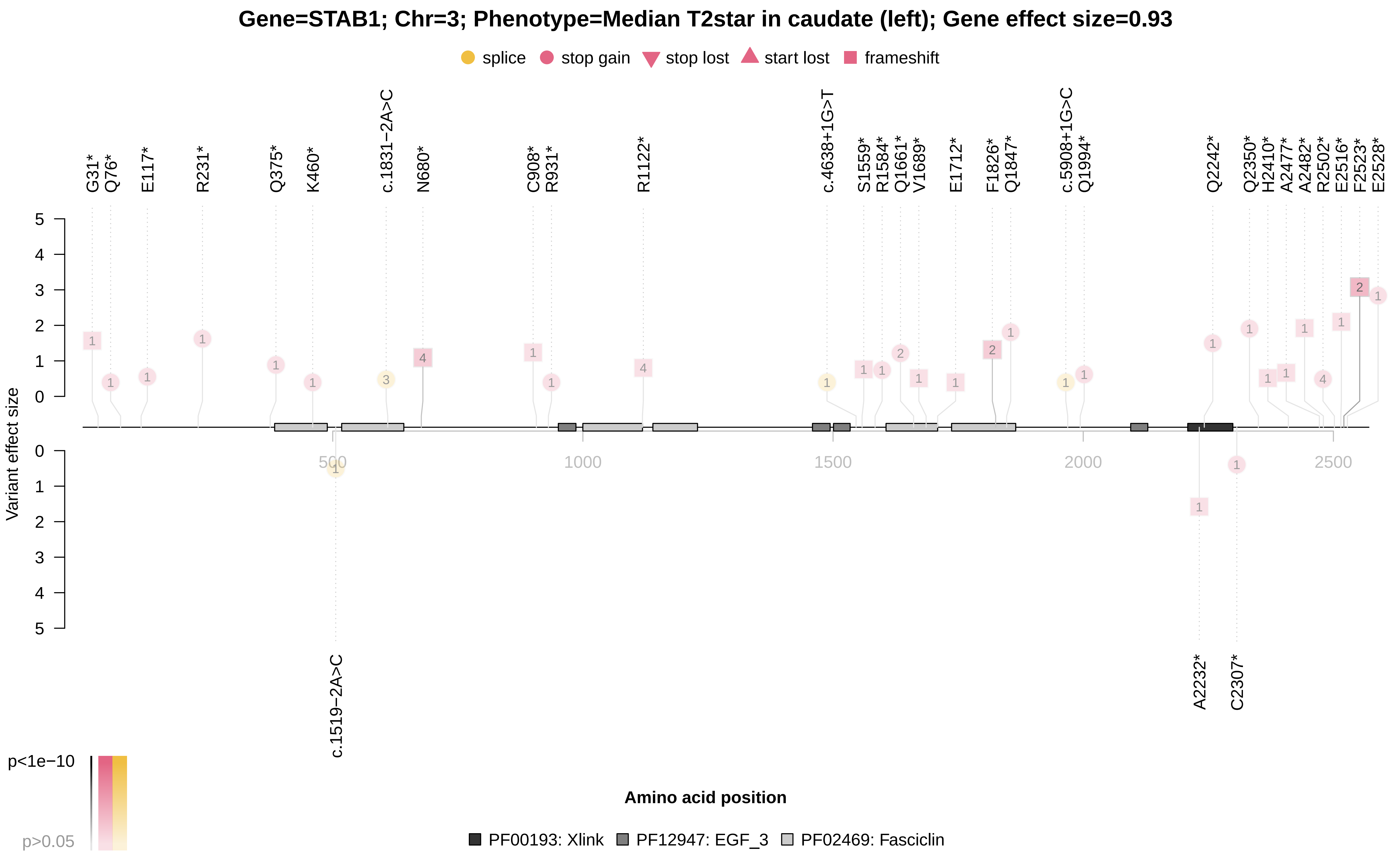

Gene=STAB1; Chr=3; Phenotype=Median T2star in caudate (left); Gene effect size=0.93
splice
stop gain
stop lost
start lost
frameshift
G31*
Q76*
E117*
R231*
Q375*
K460*
c.1831−2A>C
N680*
C908*
R931*
R1122*
c.4638+1G>T
S1559*
R1584*
Q1661*
V1689*
E1712*
F1826*
Q1847*
c.5908+1G>C
Q1994*
Q2242*
Q2350*
H2410*
A2477*
A2482*
R2502*
E2516*
F2523*
E2528*
Variant effect size
500
1000
1500
2000
2500
c.1519−2A>C
A2232*
C2307*
p<1e−10
p>0.05
Amino acid position
PF00193: Xlink
PF12947: EGF_3
PF02469: Fasciclin

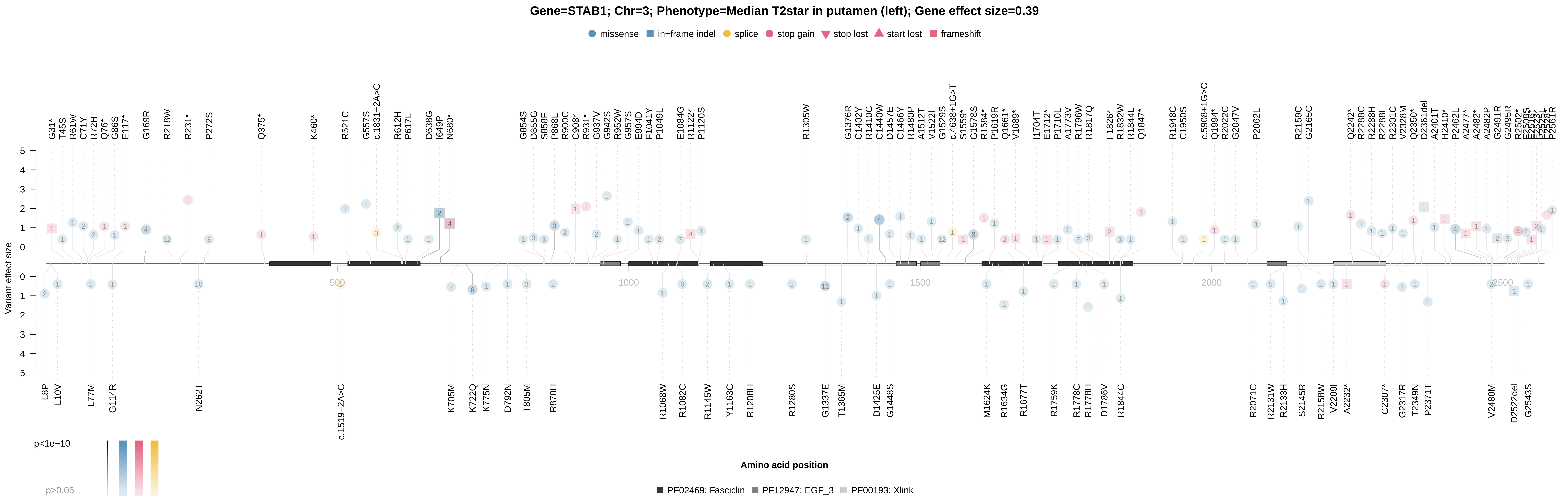
Gene=STAB1; Chr=3; Phenotype=Median T2star in putamen (left); Gene effect size=0.39
missense
in−frame indel
splice
stop gain
stop lost
start lost
frameshift
G31* T45S R61W C71Y R72H Q76* G86S E117* G169R R218W R231* P272S Q375* K460* R521C G557S c.1831−2A>C R612H P617L D638G I649P N680* G854S D855G S858F P868L R900C C908* R931* G937V G942S R952W G957S E994D F1041Y P1049L E1084G R1122* P1120S R1305W G1376R C1402Y R1410C C1440W D1457E C1466Y R1480P A1512T V1522I G1529S c.4638+1G>T S1559* G1578S R1584* P1619R Q1661* V1689* I1704T E1712* P1710L A1773V R1796W R1817Q F1826* R1832W R1844L Q1847* R1948C C1950S c.5908+1G>C Q1994* R2022C G2047V P2062L R2159C G2165C Q2242* R2288C R2288H R2288L R2301C V2328M Q2350* D2361del A2401T H2410* P2462L A2477* A2482* A2482P G2491R G2495R R2502*
Variant effect size
500
1000
1500
2000
2500
L8P L10V L77M G114R N262T c.1519−2A>C K705M K722Q K775N D792N T805M R870H R1068W R1082C R1145W Y1163C R1208H R1280S G1337E T1365M D1425E G1448S M1624K R1634G R1677T R1759K R1778C R1778H D1786V R1844C R2071C R2131W R2133H S2145R R2158W V2209I A2232* C2307* G2317R T2349N P2371T V2480M D2522del G2543S
p<1e−10
p>0.05
Amino acid position
PF02469: Fasciclin
PF12947: EGF_3
PF00193: Xlink

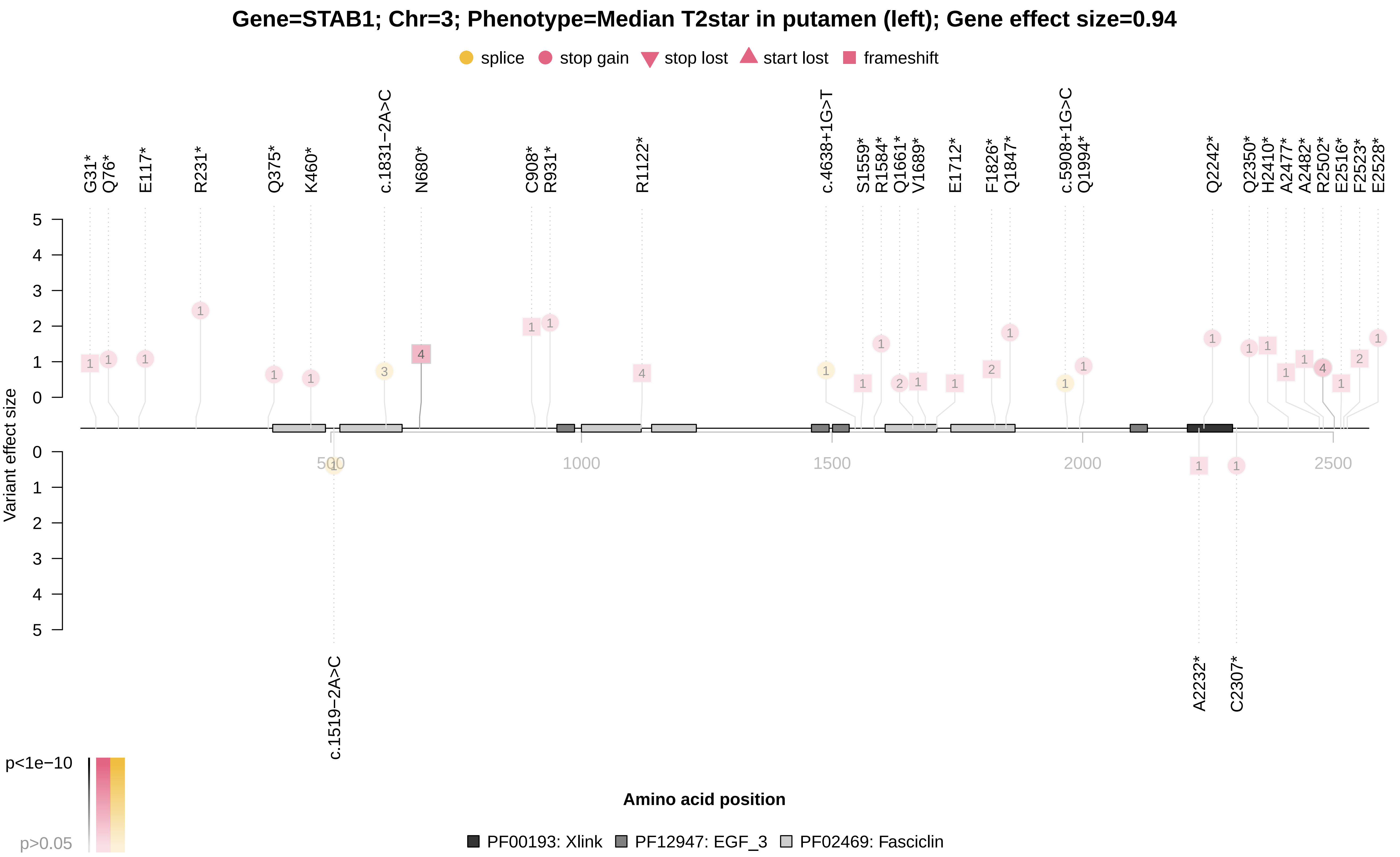

Gene=STAB1; Chr=3; Phenotype=Median T2star in putamen (left); Gene effect size=0.94
splice
stop gain
stop lost
start lost
frameshift
G31*
Q76*
E117*
R231*
Q375*
K460*
c.1831−2A>C
N680*
C908*
R931*
R1122*
c.4638+1G>T
S1559*
R1584*
Q1661*
V1689*
E1712*
F1826*
Q1847*
c.5908+1G>C
Q1994*
Q2242*
Q2350*
H2410*
A2477*
A2482*
R2502*
E2516*
F2523*
E2528*
Variant effect size
500
1000
1500
2000
2500
c.1519−2A>C
A2232*
C2307*
p<1e−10
p>0.05
Amino acid position
PF00193: Xlink
PF12947: EGF_3
PF02469: Fasciclin

Gene=STAB1; Chr=3; Phenotype=Median T2star in putamen (right); Gene effect size=0.44

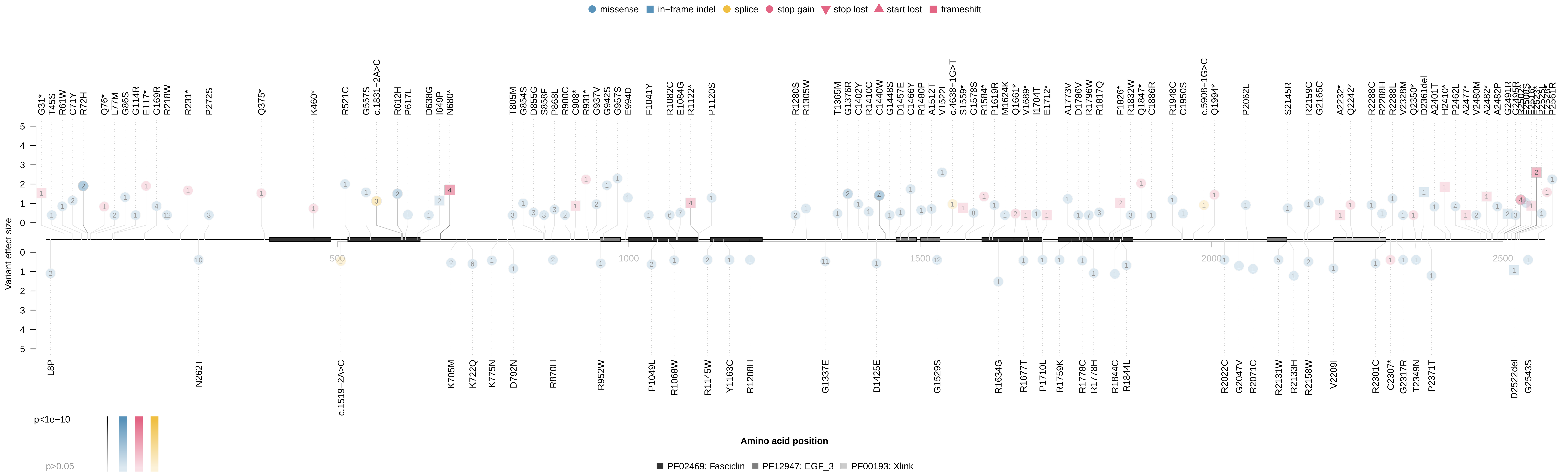

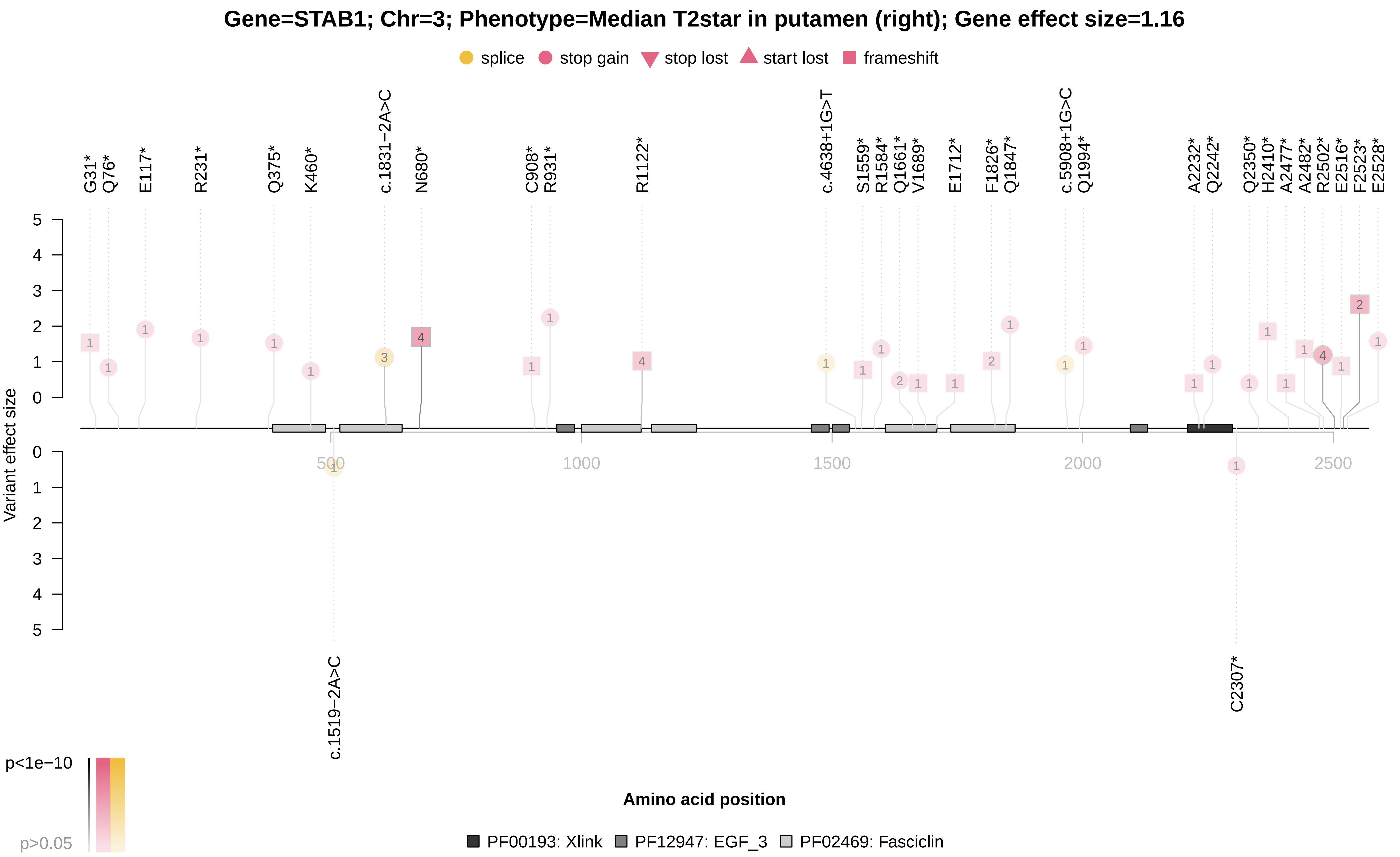

Gene=STAB1; Chr=3; Phenotype=Median T2star in putamen (right); Gene effect size=1.16
splice
stop gain
stop lost
start lost
frameshift
G31*
Q76*
E117*
R231*
Q375*
K460*
c.1831−2A>C
N680*
C908*
R931*
R1122*
c.4638+1G>T
S1559*
R1584*
Q1661*
V1689*
E1712*
F1826*
Q1847*
c.5908+1G>C
Q1994*
A2232*
Q2242*
Q2350*
H2410*
A2477*
A2482*
R2502*
E2516*
F2523*
E2528*
Variant effect size
500
1000
1500
2000
2500
c.1519−2A>C
C2307*
p<1e−10
p>0.05
Amino acid position
PF00193: Xlink
PF12947: EGF_3
PF02469: Fasciclin

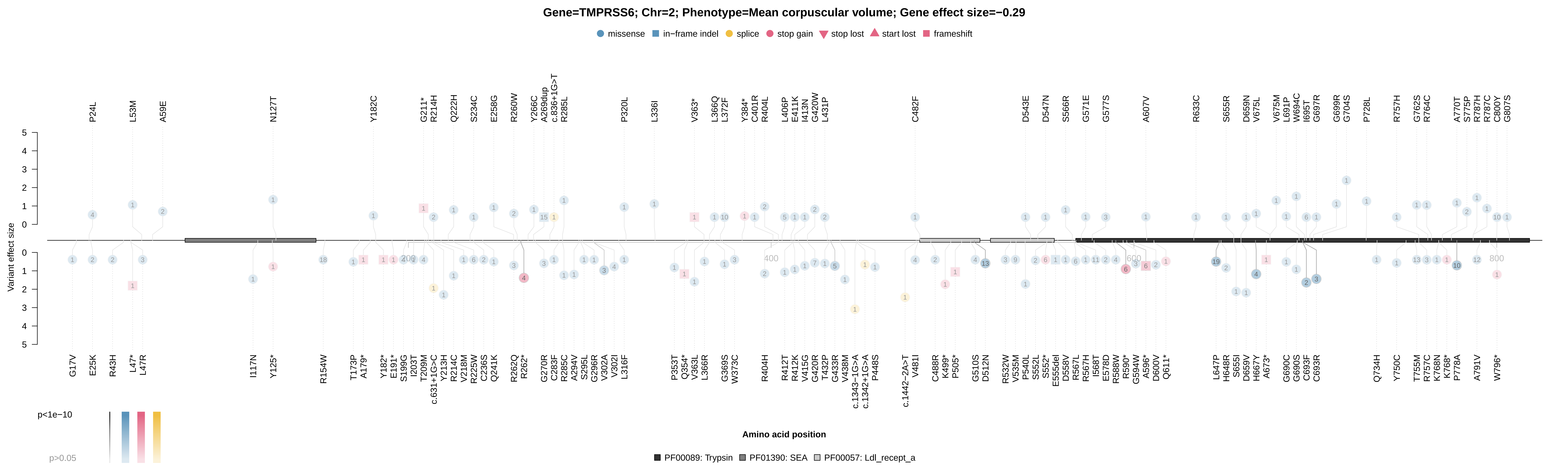

Gene=TMPRSS6; Chr=2; Phenotype=Mean corpuscular volume; Gene effect size=−0.29
missense
in−frame indel
splice
stop gain
stop lost
start lost
frameshift
P24L L53M A59E N127T Y182C G211* R214H Q222H S234C E258G R260W Y266C A269dup c.836+1G>T R285L P320L L336I V363* L366Q L372F Y384* C401R R404L L406P E411K I413N G420W L431P C482F D543E D547N S566R G571E G577S A607V R633C S655R D659N V675L V675M L691P W694C I695T G697R G699R G704S P728L R757H G762S R764C A770T S775P R787H R787C C800Y G807S
G17V E25K R43H L47* L47R I117N Y125* R154W T173P A179* Y182* E191* S199G I203T T209M c.631+1G>C Y213H R214C V218M R225W C236S Q241K R262Q R262* G270R C283F R285C A294V S295L G296R V302A V302I L316F P353T Q354* V363L L366R G369S W373C R404H R412T R412K V415G G420R T432P G433R V438M c.1343−1G>A c.1342+1G>A P448S c.1442−2A>T V481I C488R K499* P505* G510S D512N R532W V535M P540L S552L S552* E555del D558V R567L R567H I568T E578D R588W R590* G594W A596* D600V Q611* L647P H648R S655I D659V H667Y A673* G690C G690S C693F C693R Q734H Y750C T755M R757C K768N K768* P778A A791V W796*
Variant effect size
200
400
600
800
Amino acid position
p<1e−10
p>0.05
PF00089: Trypsin
PF01390: SEA
PF00057: Ldl_recept_a

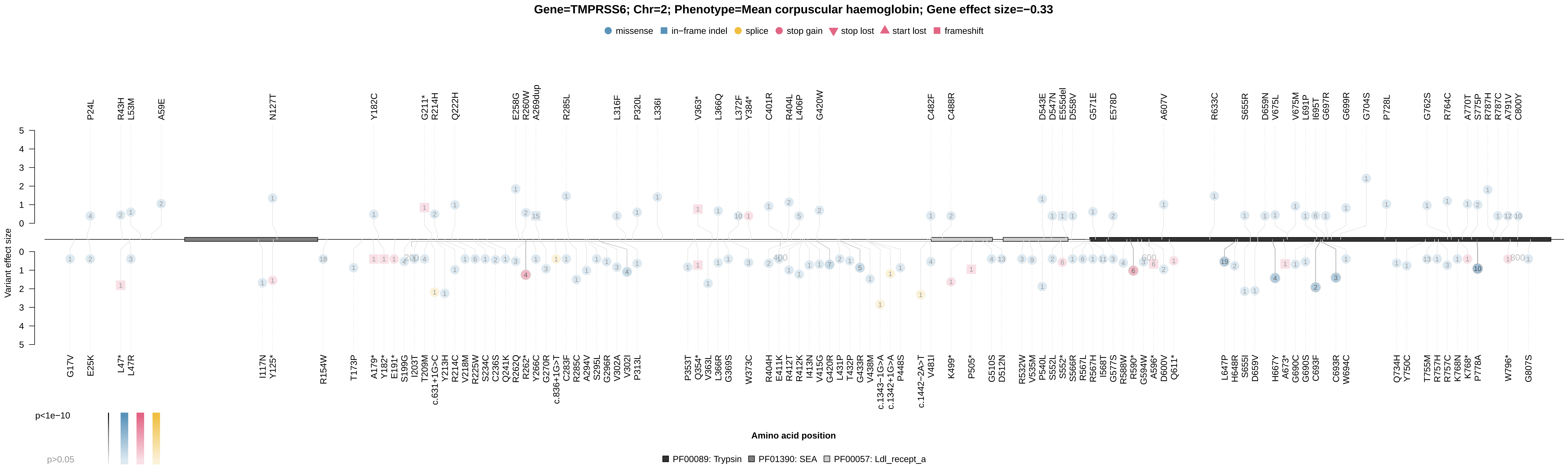

Gene=TMPRSS6; Chr=2; Phenotype=Mean corpuscular haemoglobin; Gene effect size=−0.33
missense
in−frame indel
splice
stop gain
stop lost
start lost
frameshift
P24L R43H L53M A59E N127T Y182C G211* R214H Q222H E258G R260W A269dup R285L L316F P320L L336I V363* L366Q L372F Y384* C401R R404L L406P G420W C482F C488R D543E D547N E555del D558V G571E E578D A607V R633C S655R D659N V675L V675M L691P I695T G697R G699R G704S P728L G762S R764C A770T S775P R787H R787C A791V C800Y
Variant effect size
200
400
600
800
G17V E25K L47* L47R I117N Y125* R154W T173P A179* Y182* E191* S199G I203T T209M c.631+1G>C Y213H R214C V218M R225W S234C C236S Q241K R262Q R262* Y266C G270R c.836+1G>T C283F R285C A294V S295L G296R V302A V302I P313L P353T Q354* V363L L366R G369S W373C R404H E411K R412T R412K I413N V415G G420R L431P T432P G433R V438M c.1343−1G>A c.1342+1G>A P448S c.1442−2A>T V481I K499* P505* G510S D512N R532W V535M P540L S552L S552* S566R R567L R567H I568T G577S R588W R590* G594W A596* D600V Q611* L647P H648R S655I D659V H667Y A673* G690C G690S C693F C693R W694C Q734H Y750C T755M R757H R757C K768N K768* P778A W796* G807S
p<1e−10
p>0.05
Amino acid position
PF00089: Trypsin
PF01390: SEA
PF00057: Ldl_recept_a

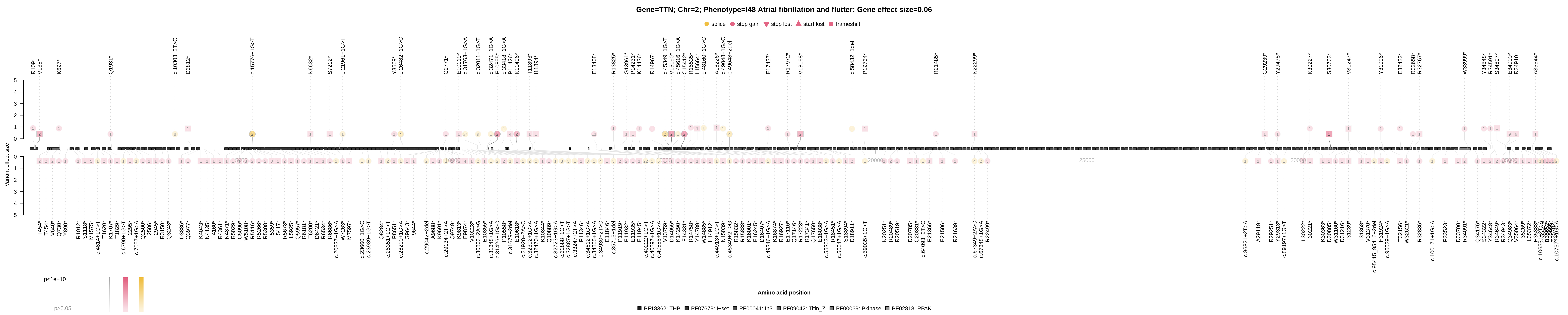

Gene=TTN; Chr=2; Phenotype=I48 Atrial fibrillation and flutter; Gene effect size=0.06
splice
stop gain
stop lost
start lost
frameshift
Variant effect size
Amino acid position
p<1e−10
p>0.05
PF18362: THB
PF07679: I-set
PF00041: fn3
PF09042: Titin_Z
PF00069: Pkinase
PF02818: PPAK

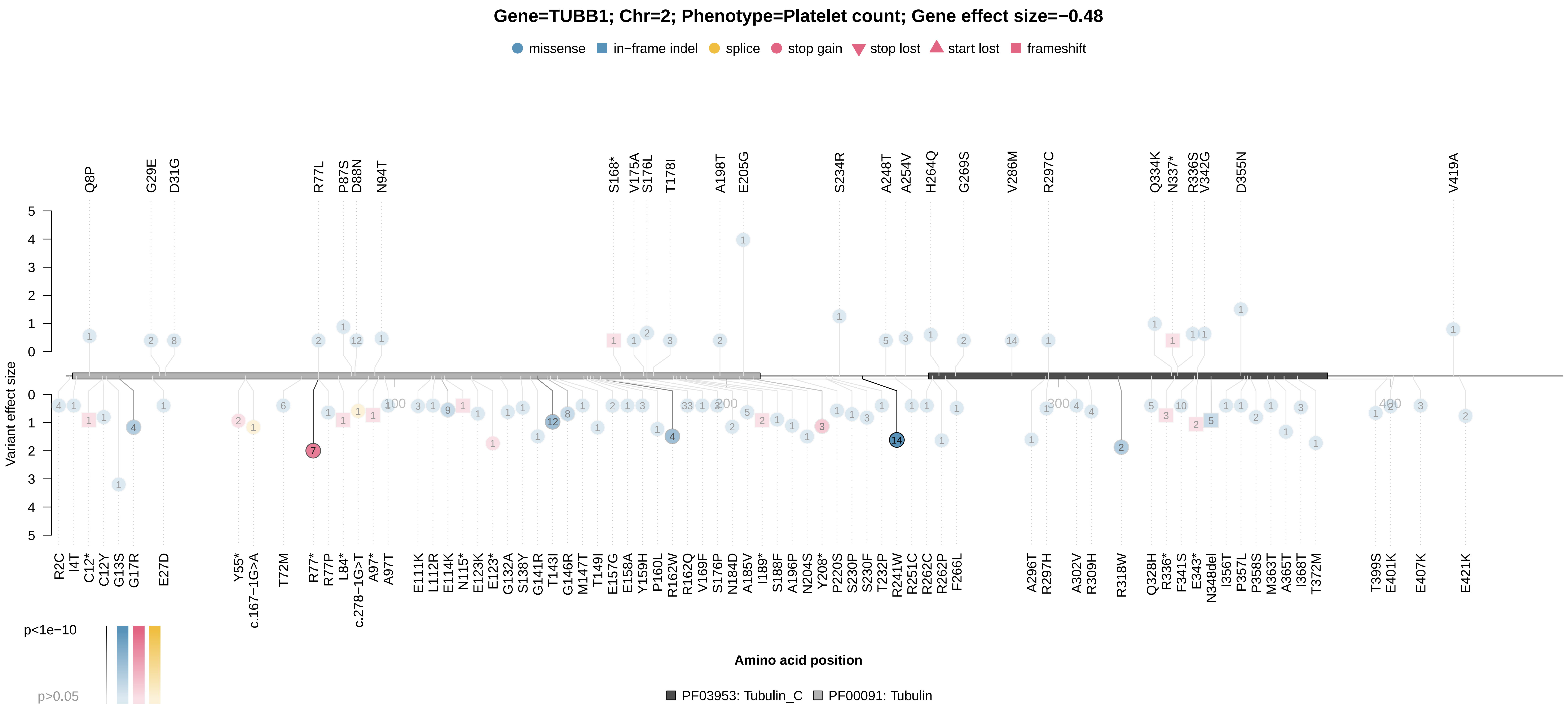

Gene=TUBB1; Chr=2; Phenotype=Platelet count; Gene effect size=−0.48
missense
in−frame indel
splice
stop gain
stop lost
start lost
frameshift
Variant effect size
Amino acid position
p<1e−10
p>0.05
PF03953: Tubulin_C
PF00091: Tubulin

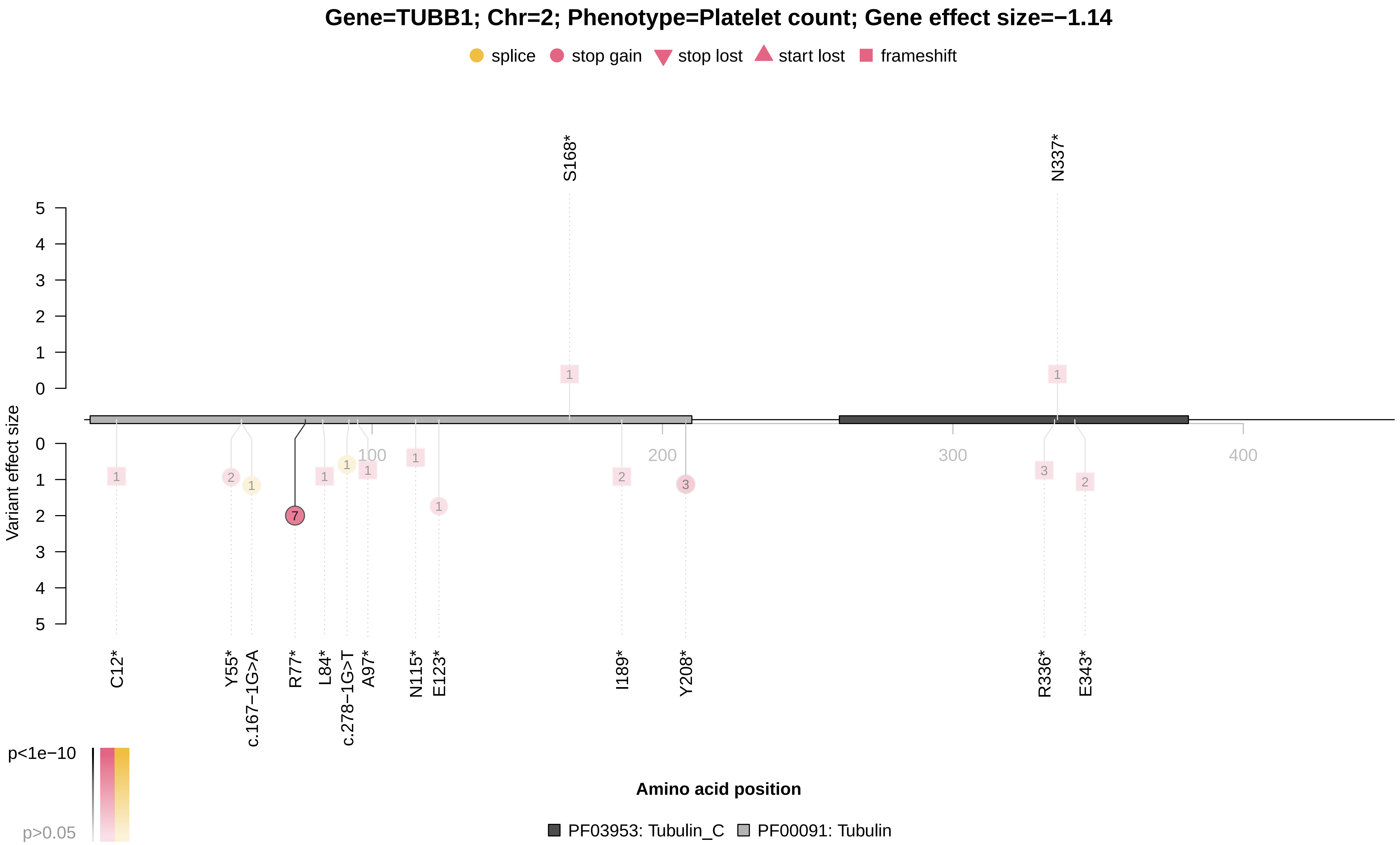

Gene=TUBB1; Chr=2; Phenotype=Platelet count; Gene effect size=−1.14
splice
stop gain
stop lost
start lost
frameshift
S168*
N337*
Variant effect size
C12*
Y55*
c.167−1G>A
R77*
L84*
c.278−1G>T
A97*
N115*
E123*
I189*
Y208*
R336*
E343*
100
200
300
400
p<1e−10
p>0.05
Amino acid position
PF03953: Tubulin_C
PF00091: Tubulin

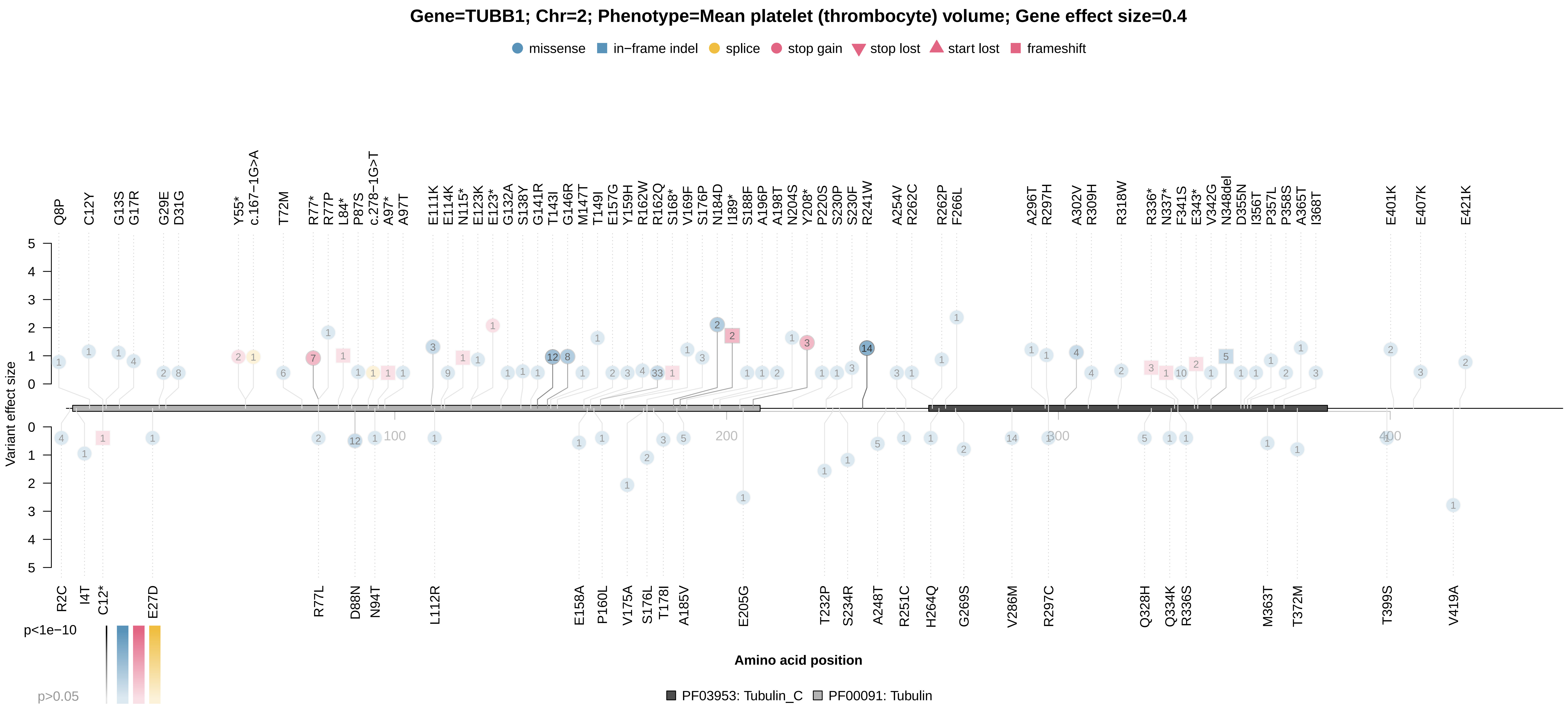
Gene=TUBB1; Chr=2; Phenotype=Mean platelet (thrombocyte) volume; Gene effect size=0.4
missense
in−frame indel
splice
stop gain
stop lost
start lost
frameshift
Q8P C12Y G13S G17R G29E D31G Y55* c.167−1G>A T72M R77* R77P L84* P87S c.278−1G>T A97* A97T E111K E114K N115* E123K E123* G132A S138Y G141R T143I G146R M147T T149I E157G Y159H R162W R162Q S168* V169F S176P N184D I189* S188F A196P A198T N204S Y208* P220S S230P S230F R241W A254V R262C R262P F266L A296T R297H A302V R309H R318W R336* N337* F341S E343* V342G N348del D355N I356T P357L P358S A365T I368T E401K E407K E421K
Variant effect size
0
1
2
3
4
5
100
200
300
400
R2C I4T C12* E27D R77L D88N N94T L112R E158A P160L V175A S176L T178I A185V E205G T232P S234R A248T R251C H264Q G269S V286M R297C Q328H Q334K R336S M363T T372M T399S V419A
p<1e−10
p>0.05
Amino acid position
PF03953: Tubulin_C
PF00091: Tubulin

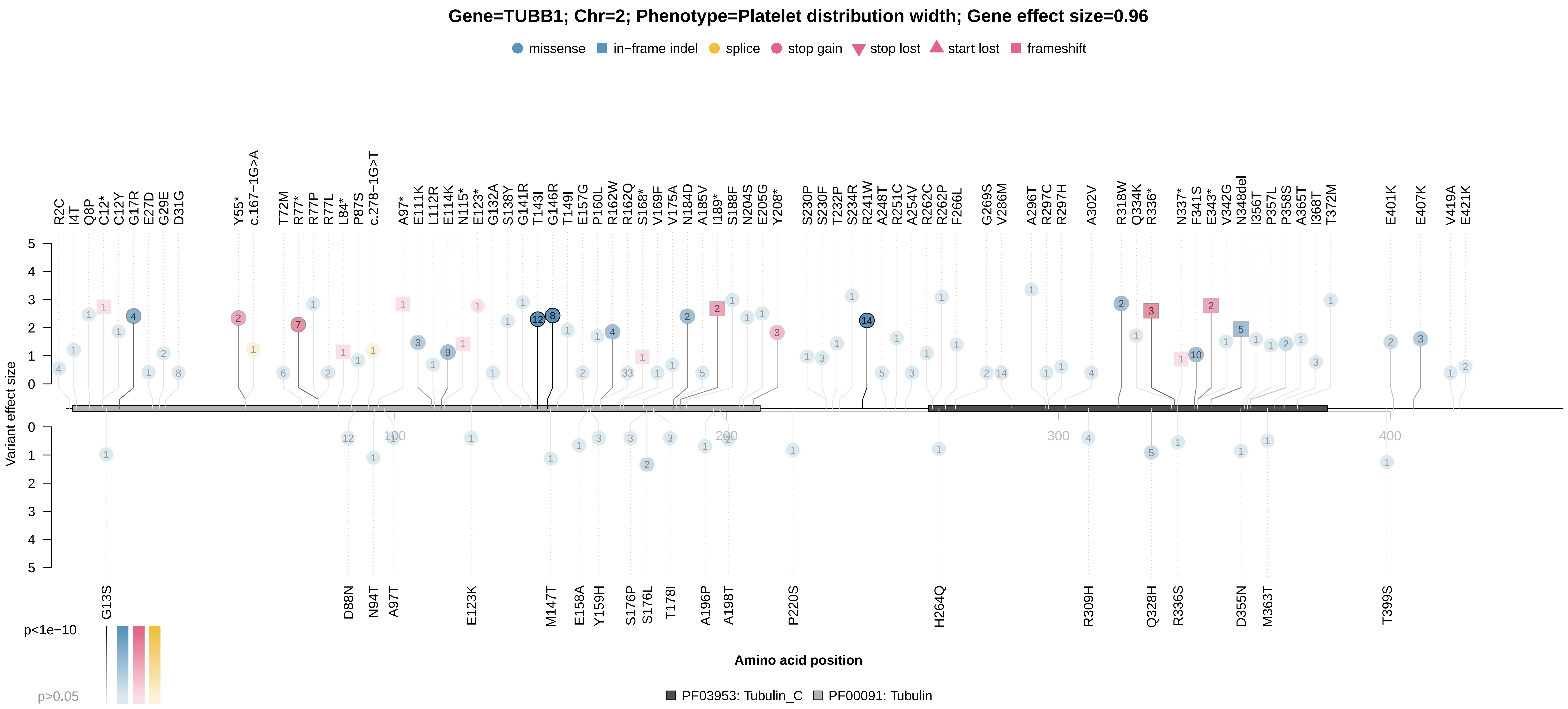

Gene=TUBB1; Chr=2; Phenotype=Platelet distribution width; Gene effect size=0.96
missense
in−frame indel
splice
stop gain
stop lost
start lost
frameshift
Variant effect size
Amino acid position
p<1e−10
p>0.05
PF03953: Tubulin_C
PF00091: Tubulin

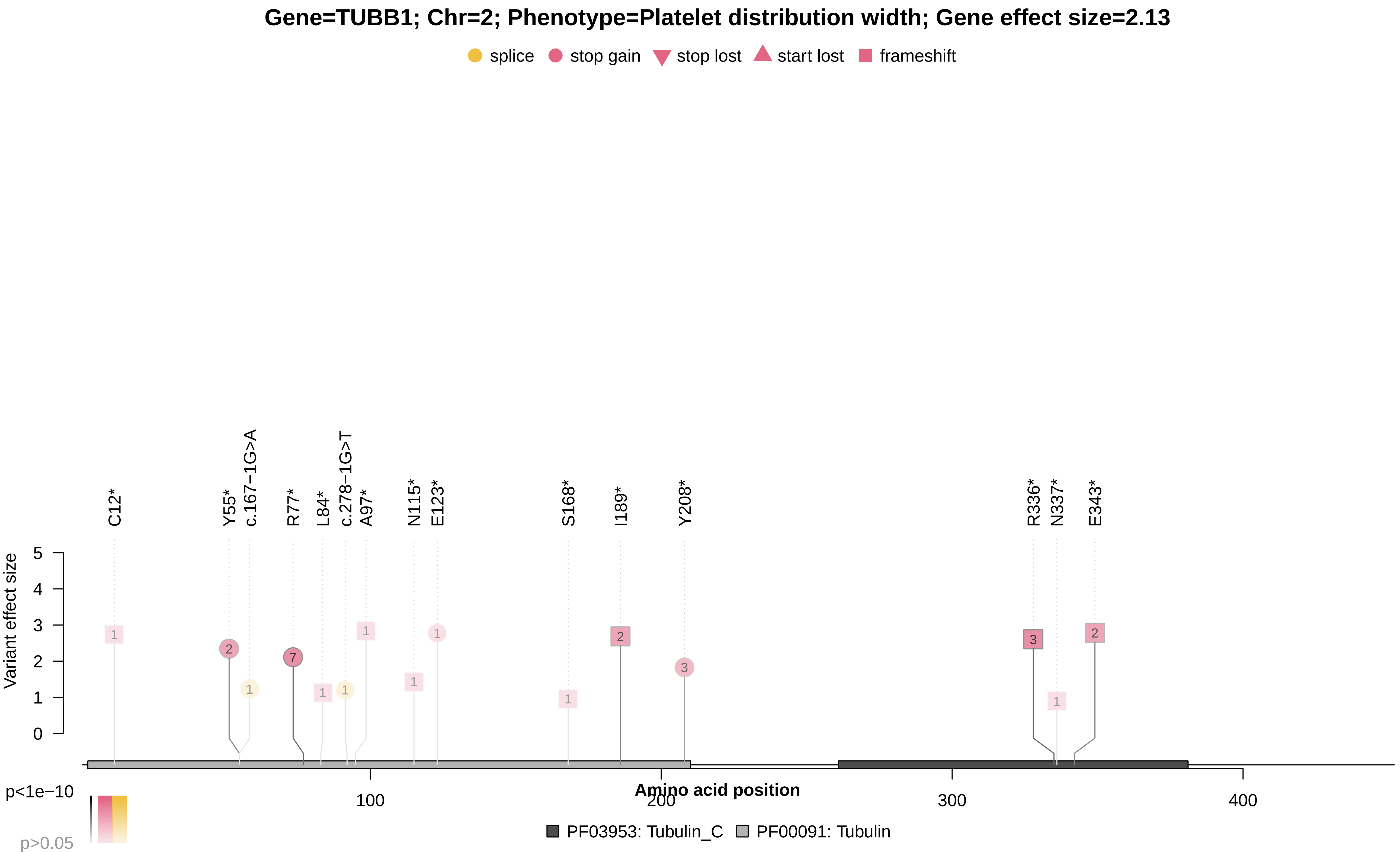

Gene=TUBB1; Chr=2; Phenotype=Platelet distribution width; Gene effect size=2.13
splice
stop gain
stop lost
start lost
frameshift
C12*
Y55*
c.167−1G>A
R77*
L84*
c.278−1G>T
A97*
N115*
E123*
S168*
I189*
Y208*
R336*
N337*
E343*
Variant effect size
5
4
3
2
1
0
Amino acid position
100
200
300
400
p<1e−10
p>0.05
PF03953: Tubulin_C
PF00091: Tubulin

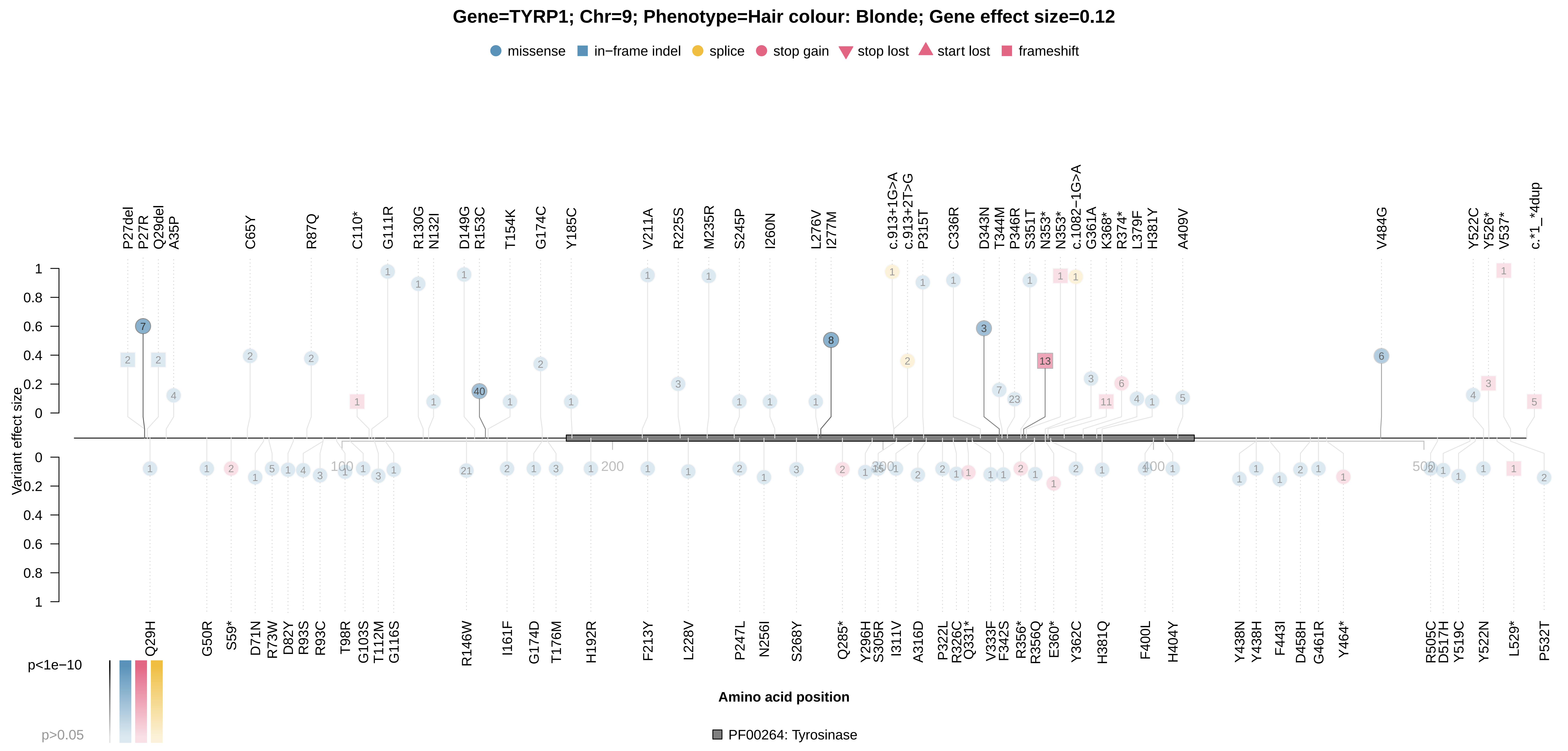

Gene=TYRP1; Chr=9; Phenotype=Hair colour: Blonde; Gene effect size=0.12
missense
in−frame indel
splice
stop gain
stop lost
start lost
frameshift
Variant effect size
Amino acid position
p<1e−10
p>0.05
PF00264: Tyrosinase

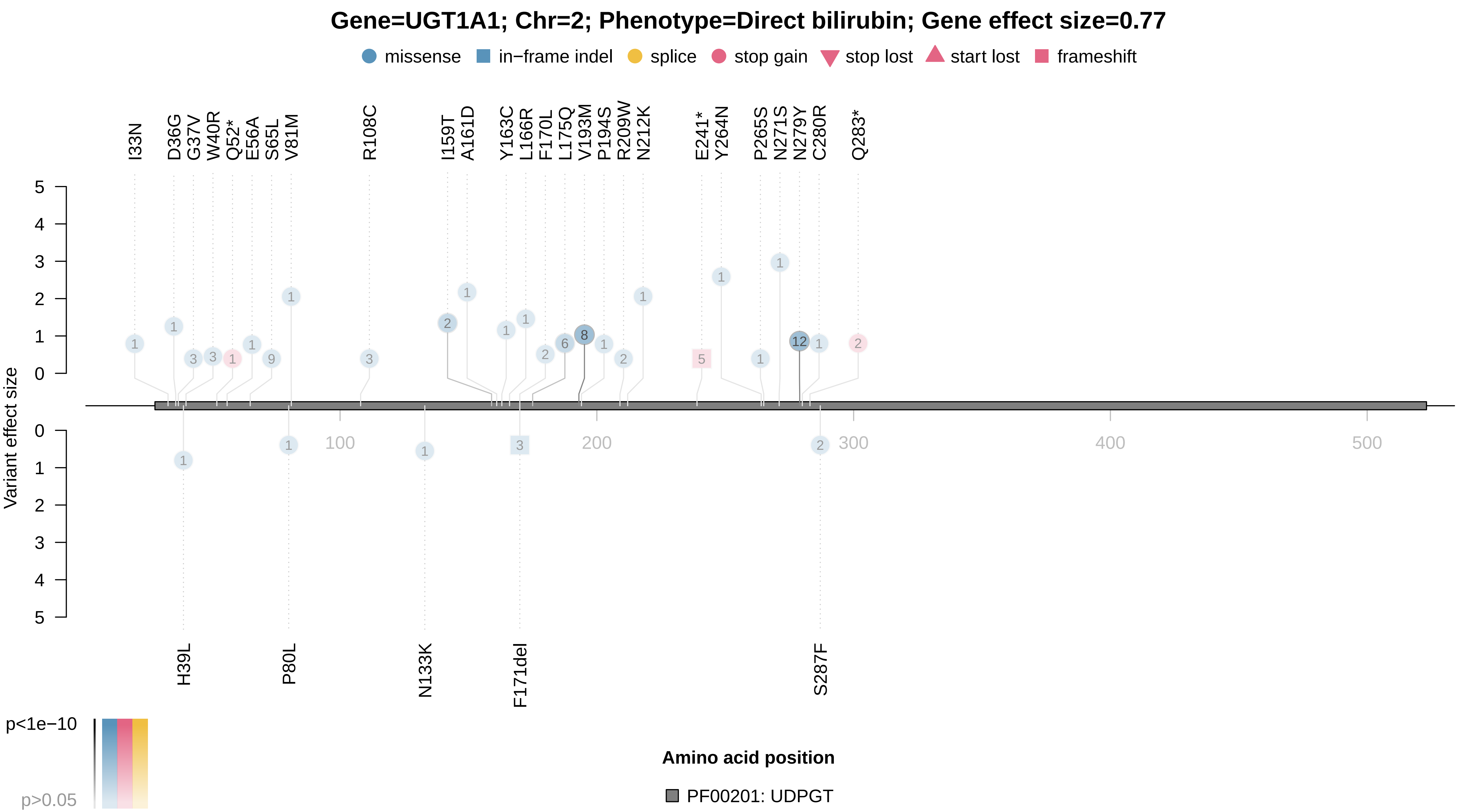

Gene=UGT1A1; Chr=2; Phenotype=Direct bilirubin; Gene effect size=0.77
missense
in−frame indel
splice
stop gain
stop lost
start lost
frameshift
I33N
D36G
G37V
W40R
Q52*
E56A
S65L
V81M
R108C
I159T
A161D
Y163C
L166R
F170L
L175Q
V193M
P194S
R209W
N212K
E241*
Y264N
P265S
N271S
N279Y
C280R
Q283*
H39L
P80L
N133K
F171del
S287F
Variant effect size
100
200
300
400
500
p<1e−10
p>0.05
Amino acid position
PF00201: UDPGT

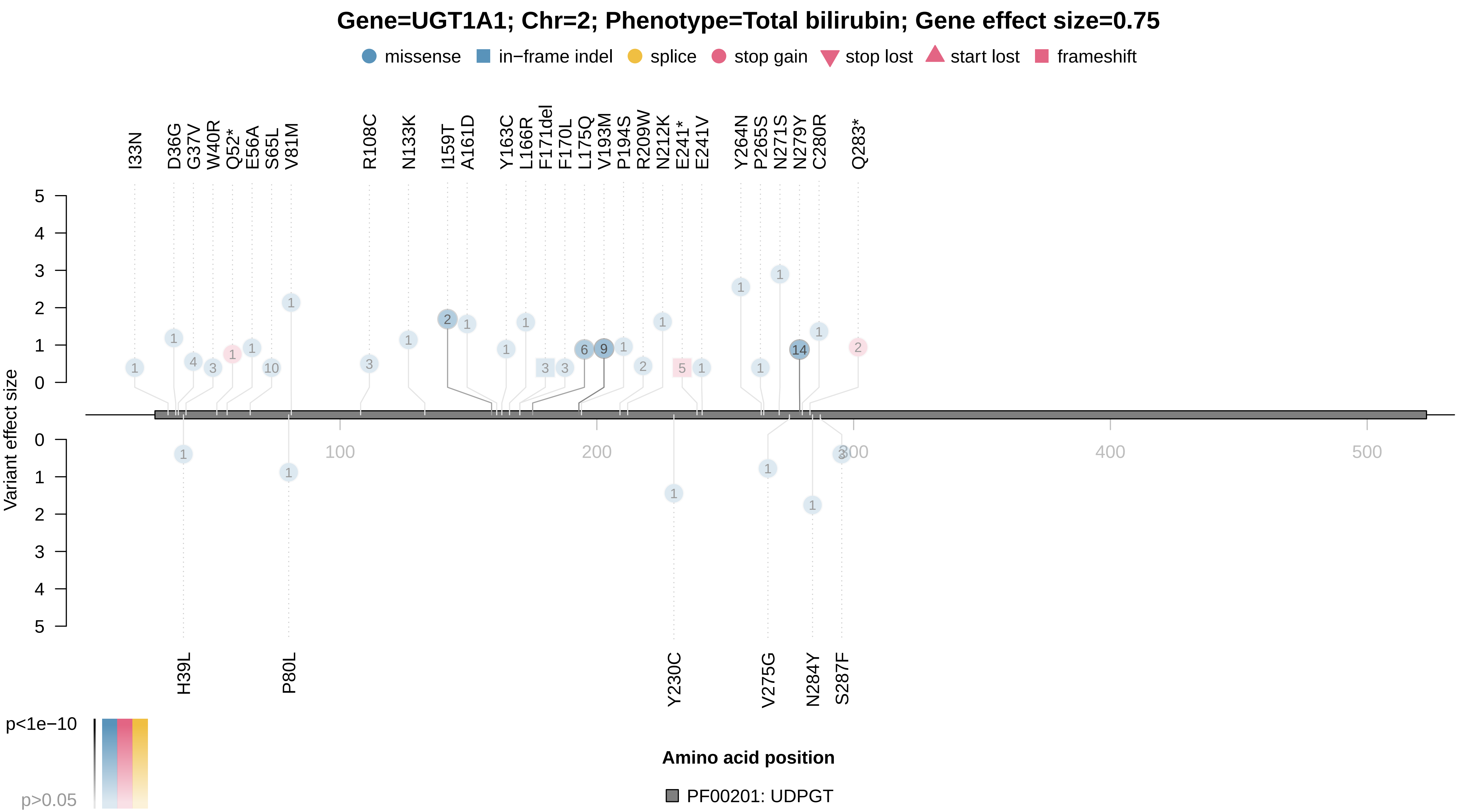

Gene=UGT1A1; Chr=2; Phenotype=Total bilirubin; Gene effect size=0.75
missense
in−frame indel
splice
stop gain
stop lost
start lost
frameshift
I33N
D36G
G37V
W40R
Q52*
E56A
S65L
V81M
R108C
N133K
I159T
A161D
Y163C
L166R
F171del
F170L
L175Q
V193M
P194S
R209W
N212K
E241*
E241V
Y264N
P265S
N271S
N279Y
C280R
Q283*
H39L
P80L
Y230C
V275G
N284Y
S287F
Variant effect size
Amino acid position
PF00201: UDPGT
p<1e−10
p>0.05

Supplementary Figure 3. Distribution of effects of rare variants on phenotypes in genes with statistically significant associations. Plots show the effect sizes of rare damaging variants on the phenotypes. The legend shows the gene, its associated phenotype, and the Effect Size (β). The effect size is computed from the gene-based collapsing model, in which individuals were coded as either having or not having a qualifying variant. A positive value indicates that variant carriers have, on average, higher values for the phenotype, while a negative value indicates that variant carriers have lower values. The amino acid positions are shown on the x-axis, with the PFAM domain highlighted. The y-axis displays the beta of each individual variant, with negative values shown below and positive values above the horizontal axis. Variants are indicated according to their consequence as shown and labeled according to their amino acid change or splice site variation. The number inside the circle is the number of people carrying that variant. Darker lines connecting the variants to the gene and darker-filled shapes indicate more significant p-values for the association. The cohort used is the UKB all ethnicities cohort.

Supplementary Table 1. Van Hout et al. comparison.

Gene	Phenotype	Van Hout p	Our p	N carriers	N expected case carriers	FET p	Replication?	Reason not replicated
TUBB1	Platelet distribution width	2.50E-23	2.50E-23	25			Yes	N/A
KALRN	Mean platelet thrombocyte volume	2.70E-23	3.90E-01	1			No	Lead Van Hout SNP MAF>0.1%
IQGAP2	Mean platelet thrombocyte volume	1.10E-19	7.80E-15	167			Yes	N/A
KLF1	Mean corpuscular haemoglobin	1.70E-16	5.00E-15	27			Yes	N/A
KLF1	Mean corpuscular volume	4.00E-14	6.90E-14	27			Yes	N/A
KLF1	Red blood cell erythrocyte distribution width	1.50E-13	1.50E-14	27			Yes	N/A
IL33	Eosinophill percentage	5.40E-12	7.90E-01	85			No	Lead Van Hout SNP MAF>0.1%
ASXL1	Red blood cell erythrocyte distribution width	2.40E-11	3.30E-06	126			No	Unclear, appears due to difference in variant calls used for indels
MLH1	Z85.0, Personal history of malignant neoplasm of digestive organs	3.50E-11	5.30E-26	6	0	3.64E-07	No	Too few expected carriers for regression, and Fisher's exact not significant
PKD1	N18, Chronic kidney disease (CKD)	2.90E-10	N/A	N/A	N/A	N/A	No	Outside of GIAB high confidence regions
IL33	Eosinophill count	3.30E-10	5.30E-01	85			No	Lead Van Hout SNP MAF>0.1%
COL6A1	Corneal resistance factor mean	3.60E-10	1.30E-05	10			No	Some variants outside of GIAB high confidence regions
HBB	Red blood cell erythrocyte count	1.70E-09	1.20E-01	1			No	Too few carriers in this population subset
TUBB1	Platelet count	2.10E-09	5.60E-10	25			No	Difference in p-value cutoff
ASXL1	Platelet distribution width	4.70E-09	8.00E-08	126			No	Unclear, appears due to difference in variant calls used for indels
GMPR	Mean corpuscular haemoglobin	1.10E-08	1.60E-02	7			No	Lead Van Hout SNP MAF>0.1%
TTN	I42, Cardiomyopathy	1.40E-08	3.90E-15	9	1	2.96E-06	No	Too few expected carriers for regression, and Fisher's exact not significant

MEPE	Heel bone mineral density	1.40E-08	3.30E-05	106			No	Unclear, appears due to difference in variant calls used for indels
COL6A1	Corneal hysteresis mean	2.10E-08	7.00E-05	10			No	Some variants outside of GIAB high confidence regions
TUBB1	Mean platelet thrombocyte volume	2.40E-08	2.10E-07	25			No	Small difference in p-value
PIEZO1	I83.9, Asymptomatic varicose veins of lower extremities	2.70E-08	2.30E-15	19	4	7.16E-06	No	Too few expected carriers for regression, and Fisher's exact not significant
CALR	D47, Other neoplasms of uncertain behavior of lymphoid, hematopoietic and related tissue	4.10E-08	7.60E-122	2	0	2.06E-05	No	Too few expected carriers for regression, and Fisher's exact not significant
HBB	Red blood cell erythrocyte distribution width	5.80E-08	1.80E-02	1			No	Too few carriers in this population subset
GP1BA	Mean platelet thrombocyte volume	6.40E-08	8.40E-09	88			No	Difference in p-value cutoff
CHEK2	Platelet crit	7.90E-08	7.60E-01	55			No	Lead Van Hout SNP MAF>0.1%

The p-values from Van Hout et al. are from an analysis of unrelated European ancestry individuals from the UKB cohort with a MAF cutoff of 1%. The p-values shown for our analysis are from the linear mixed model (LMM) analysis of the European UKB cohort, including relatives, with a 0.1% MAF cutoff and a restriction to Genome in a Bottle (GIAB) high-confidence regions. The number of carriers in our analysis is shown for quantitative traits, and for binary traits the number of case carriers and expected number of case carriers (given overall prevalence) is shown separately. Fisher's exact test (FET) is performed for binary traits as well.

Supplementary References

1. Rentzsch, P., Witten, D., Cooper, G. M., Shendure, J. & Kircher, M. CADD: predicting the deleteriousness of variants throughout the human genome. *Nucleic Acids Res.* **47**, D886–D894 (2019).
2. Kircher, M. *et al.* A general framework for estimating the relative pathogenicity of human genetic variants. *Nat. Genet.* **46**, 310–315 (2014).
3. Itan, Y. *et al.* The mutation significance cutoff: gene-level thresholds for variant predictions. *Nat. Methods* **13**, 109–110 (2016).
4. Lee, S. *et al.* Optimal unified approach for rare-variant association testing with application to small-sample case-control whole-exome sequencing studies. *Am. J. Hum. Genet.* **91**, 224–237 (2012).
5. Hail Team. Hail 0.2.21-f16fd64e0d77. https://github.com/hail-is/hail/releases/tag/0.2.21
6. Long, T. *et al.* Whole-genome sequencing identifies common-to-rare variants associated with human blood metabolites. *Nat. Genet.* **49**, 568–578 (2017).
7. Girard, S. L. *et al.* Mutation burden of rare variants in schizophrenia candidate genes. *PLoS One* **10**, e0128988 (2015).
