# Supplementary material for: Genome-wide rare variant analysis for thousands of phenotypes in over 70,000 exomes from two cohorts
Source: Nat Commun. 2020 Jan 28;11:542. doi: 10.1038/s41467-020-14288-y (PMC6987107; doi:10.1038/s41467-020-14288-y)
Supplement: Supplementary file 3 — Reporting Summary [file 41467_2020_14288_MOESM3_ESM.pdf]

nature research

Corresponding author(s): Elizabeth T. Cirulli

Last updated by author(s): Dec 16, 2019

Reporting Summary

Nature Research wishes to improve the reproducibility of the work that we publish. This form provides structure for consistency and transparency in reporting. For further information on Nature Research policies, see Authors & Referees and the Editorial Policy Checklist .

Statistics

For all statistical analyses, confirm that the following items are present in the figure legend, table legend, main text, or Methods section.

n/a	Confirmed	
☐	☒	The exact sample size (*n*) for each experimental group/condition, given as a discrete number and unit of measurement
☐	☒	A statement on whether measurements were taken from distinct samples or whether the same sample was measured repeatedly
☐	☒	The statistical test(s) used AND whether they are one- or two-sided<br>*Only common tests should be described solely by name; describe more complex techniques in the Methods section.*
☐	☒	A description of all covariates tested
☐	☒	A description of any assumptions or corrections, such as tests of normality and adjustment for multiple comparisons
☐	☒	A full description of the statistical parameters including central tendency (e.g. means) or other basic estimates (e.g. regression coefficient) AND variation (e.g. standard deviation) or associated estimates of uncertainty (e.g. confidence intervals)
☐	☒	For null hypothesis testing, the test statistic (e.g. *F*, *t*, *r*) with confidence intervals, effect sizes, degrees of freedom and *P* value noted<br>*Give P values as exact values whenever suitable.*
☒	☐	For Bayesian analysis, information on the choice of priors and Markov chain Monte Carlo settings
☒	☐	For hierarchical and complex designs, identification of the appropriate level for tests and full reporting of outcomes
☐	☒	Estimates of effect sizes (e.g. Cohen's *d*, Pearson's *r*), indicating how they were calculated

Our web collection on statistics for biologists contains articles on many of the points above.

Software and code

Policy information about availability of computer code

Data collection	UKB data are available to all researchers and collected as described by UKB. HNP phenotypes were collected from Epic/Clarity EHR data. Microsoft SQL Server was used as a backend database for record storage. SAS 9.4 M5 with SAS/ACCESS to SQL Server was used to perform ETL on these data in preparation for analysis, also in SAS using Base SAS and SAS/STAT.
Data analysis	We use Sentieon, Hail, BOLTLMM, PHESANT, and PLINK, all of which are available and have appropriate citations in the manuscript.

For manuscripts utilizing custom algorithms or software that are central to the research but not yet described in published literature, software must be made available to editors/reviewers. We strongly encourage code deposition in a community repository (e.g. GitHub). See the Nature Research guidelines for submitting code & software for further information.

Data

Policy information about availability of data

All manuscripts must include a data availability statement. This statement should provide the following information, where applicable:

- Accession codes, unique identifiers, or web links for publicly available datasets
- A list of figures that have associated raw data
- A description of any restrictions on data availability

UKB data are available for download (https://www.ukbiobank.ac.uk/). Analysis results are available for download at https://s3.amazonaws.com/helix-research-public/ukbb_exome_analysis_results/README.txt, and also browseable with an interactive web tool at https://ukb.research.helix.com. All summary statistics for significant associations are available for both UKB and HNP in Supplementary Data 2. Restrictions apply to the availability of the HNP data, which were used under license for the current study, and thus are not publicly available. The HNP data are available for qualified researchers upon reasonable request to Craig.Kugler@dri.edu and Joe.Grzymski@dri.edu and with permission of the Institute for Health Innovation and Helix. Researchers who would like to obtain the raw data related to this study will be presented with a data user agreement, which requires that the participants will not be re-identified and no data will be shared between researchers or uploaded onto public domains.

Field-specific reporting

Please select the one below that is the best fit for your research. If you are not sure, read the appropriate sections before making your selection.

- [x] Life sciences
- [ ] Behavioural & social sciences
- [ ] Ecological, evolutionary & environmental sciences

For a reference copy of the document with all sections, see nature.com/documents/nr-reporting-summary-flat.pdf

Life sciences study design

All studies must disclose on these points even when the disclosure is negative.

Sample size	Sample sizes were determined from the available data.
Data exclusions	Low-quality variants were excluded using GATK-suggested best practices. Genes with fewer than 10 people carrying qualifying variants in them were excluded from the LMM.
Replication	As detailed in the paper, in addition to a meta-analysis across the UKB and HNP, the same discovery analyses performed in the UKB cohort were performed in HNP data, and all signals for phenotypes measured in both cohorts either formally replicated or at least showed concordant directions of effect.
Randomization	Data were collected from population cohorts and sequenced in an order that was random with regard to their phenotypes.
Blinding	Blinding was not required in this study: large-scale data were collected from populations.

Reporting for specific materials, systems and methods

We require information from authors about some types of materials, experimental systems and methods used in many studies. Here, indicate whether each material, system or method listed is relevant to your study. If you are not sure if a list item applies to your research, read the appropriate section before selecting a response.

Materials & experimental systems

n/a	Involved in the study	
x		Antibodies
x		Eukaryotic cell lines
x		Palaeontology
x		Animals and other organisms
	x	Human research participants
x		Clinical data

Methods

n/a	Involved in the study	
x		ChIP-seq
x		Flow cytometry
x		MRI-based neuroimaging

Human research participants

Policy information about studies involving human research participants

Population characteristics	We used two cohorts in this analysis (Table 1). The first cohort was the set of sequenced UKB exomes (Table 1). The UKB participants are between the ages of 40 and 69, and each has been extensively phenotyped, including consenting to making their medical records available. As described previously, the exome-sequenced set of UKB samples is enriched for individuals with MRI data, enhanced baseline measurements, hospital episode statistics, and linked primary care records (described for Category 170 at http://biobank.ctsu.ox.ac.uk/crystal/label.cgi?id=170). Of the 49,960 exome-sequenced individuals, 55% are female, and 40,468 are genetically classified by the UKB as genetically of European ancestry (field 22006).<br><br>The second cohort included the exomes of 21,866 participants from the Healthy Nevada Project (Table 1). These are unselected patients from Northern Nevada (Renown Health, Reno, Nevada) who consented to research involving their electronic medical records15. The participants in this cohort are aged 18 to 89+, and 68% are female. We classified 17,238 of these as European ancestry using principal component analysis based on 184,445 representative common variants (see Methods).
Recruitment	HNP participants were unselected patients from the Renown Health system in Reno, Nevada.
Ethics oversight	The HNP study was reviewed and approved by the University of Nevada, Reno Institutional Review Board (IRB, project 956068-12), and all participants provided informed consent. This research has been conducted using the UK Biobank Resource under Application Number 40436.

Note that full information on the approval of the study protocol must also be provided in the manuscript.
