# Supplementary material for: Genome-wide rare variant analysis for thousands of phenotypes in over 70,000 exomes from two cohorts
Source: Nat Commun. 2020 Jan 28;11:542. doi: 10.1038/s41467-020-14288-y (PMC6987107; doi:10.1038/s41467-020-14288-y)
Supplement: Supplementary file 4 — Description of Additional Supplementary Files [file 41467_2020_14288_MOESM4_ESM.pdf]

Title: Supplementary Data 1.
Description: Phenotypes analyzed. Information provided about each phenotype includes the number of genes analyzed. For the quantitative traits, at least 5 carriers were required. For the binary traits, for the LMM models, at least 10 expected case carriers based on the overall prevalence was required. For the Fisher's exact tests, there was no required number of carriers, but here we only count the genes with at least 5 case carriers because no associations with fewer than 5 case carriers produced a statistically significant signal.

Title: Supplementary Data 2.
Description: Statistically significant associations. The results are provided for the 101 significant associations. The first columns indicate the type of analysis that led to the main significant association and the p-value for that association, with and without the lead variant removed. Later columns provide the broken down results for all analyses for that gene-phenotype combination.
